# Insights into the innate immunity of the Mediterranean mussel Mytilus galloprovincialis

**DOI:** 10.1186/1471-2164-12-69

**Published:** 2011-01-26

**Authors:** Paola Venier, Laura Varotto, Umberto Rosani, Caterina Millino, Barbara Celegato, Filippo Bernante, Gerolamo Lanfranchi, Beatriz Novoa, Philippe Roch, Antonio Figueras, Alberto Pallavicini

**Affiliations:** 1 Department of Biology, University of Padova, Via U. Bassi, 58/B, 35121, Padova, Italy; 2 C.R.I.B.I. Biotechnology Centre, University of Padova, Via U. Bassi, 58/B, 35121, Padova, Italy; 3 Instituto de Investigaciones Marinas, CSIC, C/Eduardo Cabello, 6, E-36208 Vigo, Spain; 4 Ecosystèmes Lagunaires UMR CNRS-University of Montpellier 2, cc093, place E. Bataillon, F-34095 Montpellier cedex 05, France; 5 Department of Life Sciences, University of Trieste, P.le Valmaura, 9, 34148 Trieste, Italy

## Abstract

**Background:**

Sessile bivalves of the genus *Mytilus *are suspension feeders relatively tolerant to a wide range of environmental changes, used as sentinels in ecotoxicological investigations and marketed worldwide as seafood. Mortality events caused by infective agents and parasites apparently occur less in mussels than in other bivalves but the molecular basis of such evidence is unknown. The arrangement of Mytibase, interactive catalogue of 7,112 transcripts of *M. galloprovincialis*, offered us the opportunity to look for gene sequences relevant to the host defences, in particular the innate immunity related genes.

**Results:**

We have explored and described the Mytibase sequence clusters and singletons having a putative role in recognition, intracellular signalling, and neutralization of potential pathogens in *M. galloprovincialis*. Automatically assisted searches of protein signatures and manually cured sequence analysis confirmed the molecular diversity of recognition/effector molecules such as the antimicrobial peptides and many carbohydrate binding proteins. Molecular motifs identifying complement C1q, C-type lectins and fibrinogen-like transcripts emerged as the most abundant in the Mytibase collection whereas, conversely, sequence motifs denoting the regulatory cytokine MIF and cytokine-related transcripts represent singular and unexpected findings. Using a cross-search strategy, 1,820 putatively immune-related sequences were selected to design oligonucleotide probes and define a species-specific Immunochip (DNA microarray). The Immunochip performance was tested with hemolymph RNAs from mussels injected with *Vibrio splendidus *at 3 and 48 hours post-treatment. A total of 143 and 262 differentially expressed genes exemplify the early and late hemocyte response of the *Vibrio*-challenged mussels, respectively, with AMP trends confirmed by qPCR and clear modulation of interrelated signalling pathways.

**Conclusions:**

The Mytibase collection is rich in gene transcripts modulated in response to antigenic stimuli and represents an interesting window for looking at the mussel immunome (transcriptomes mediating the mussel response to non-self or abnormal antigens). On this basis, we have defined a new microarray platform, a mussel Immunochip, as a flexible tool for the experimental validation of immune-candidate sequences, and tested its performance on *Vibrio*-activated mussel hemocytes. The microarray platform and related expression data can be regarded as a step forward in the study of the adaptive response of the *Mytilus *species to an evolving microbial world.

## Background

DNA sequencing, genomic and post-genomic techniques have made available long lists of partially described sequences and impose the construction of databases essential for mining very large data sets [1,2]. Whenever complete transcript sequences and gene structure information are not available, misidentification and erroneous annotation can easily occur. In fact, the greatest challenge in biology today is the precise delineation of genes and protein networks able to explain physiological and pathological phenotypes [3-5].

Besides well known model organisms, a number of invertebrate species differing in life cycles and adaptive strategies support the current understanding of the innate immunity, especially those living in fluctuating marine systems [6-9]. Filter-feeder bivalves such as mussels, oysters and clams typically harbour a community of commensal, opportunistic and pathogenic organisms composed of endoparasites such as *Mytilicola *and *Urastoma*, protozoans such as *Bonamia, Haplosporidium Marteilia, Perkinsus *spp., bacteria of the genus *Nocardia *and *Vibrio, Herpes *and enteric viruses. Microbial species take part in the biogeochemical cycles and some of them are expected to play a probiotic role in their typical hosts. The common rod-shaped *Vibrios *(> 60 Gram negative heterotrophic species) well exemplify associations ranging from mutualistic to pathogenic in aquatic animals [10-12]. *V. cholerae, V. parahaemolyticus, V. vulnificus *and other nine *Vibrio *species cause mild or severe syndromes in humans while other halophilic *Vibrios *occurring in brackish and marine habitats can greatly affect molluscs, crustaceans and fish (e.g. *V. tapetis, V. alginolyticus, V. splendidus, V. pectenicida, V. harvey, V. penaeicida, V. anguillarum*). Often triggered by environmental factors such as temperature, salinity or pollutants, elements of such microbiota may invade and colonize the host and eventually lead to disease outbreaks and mortality, especially in larvae, spat and juveniles of natural and farmed bivalves [13-15]. Compared to oyster and clams, no apparent mortality and fewer pathologies have been reported in mussels [16,17]. It is more likely that *Mytilus *spp. are a reservoir of infective agents for aquatic organisms and humans, since, for instance, they tolerate significant amounts of *V. alginolyticus, V. parahemolyticus *and other vibrios [18]. In fact, comparative and advanced understanding of the early-induced host responses may sustain and improve the aquaculture production in many coastal regions worldwide [17,19].

Immunocompetent mollusc cells, at least the circulating hemocytes, and a variety of molecular effectors provide a rapid and robust line of defence against potential pathogens. Once activated by the interaction between pathogen associated molecular patterns (PAMPs) and pathogen recognition receptors (PRR), such cells display chemotactic and chemokinetic reactions, participate in encapsulation and melanization, carry out phagocytic or lytic killing. These events are made possible by the concerted action of transmembrane and soluble lectins, Toll-like and virus sensing receptors, hydrolytic enzymes and proteolytic reaction cascades, short-lived cytotoxic by-products and antimicrobial peptides (AMP) [20-25]. According to morphological observations and flow cytometry, bivalve hemocytes are heterogeneous and very dynamic cells of 7-10 μm size which can be classified into large granulocytes (eosinophilic) most active in phagocytosis and ROS production, large hyalinocytes with intermediate activity, small non-phagocytic semigranular cells (basophilic) and the less abundant blast-like hyalinocytes [26-28]. As *Mytilus *hemocytes respond to interleukin 1 (IL 1), tumour necrosis factor (TNF) and to opioid peptides (the endogenous messengers between the nervous and the immune system) they may be part of an ancient monokine-like network [29,30]. Also relevant to the use of mussels as biosensors of coastal pollution [31] the interdependence of cell processes modulated by chemical contaminants and infective agents requires additional study [32,33].

The sequence data available for bivalve species are slowly but steadily growing, especially through EST collections [4,8,34]. A set of 1,714 cDNA probes of *M. galloprovincialis *was arranged to investigate the transcriptional signatures of pollutants [35] but more work has subsequently been devoted to EST sequencing, also using technologies which provide very large amounts of short reads more difficult to annotate [36]. A double set of 5' and 3' ESTs of *M. californianus*, 42,354 in total, was used to investigate the influence of the tidal cycle on mussel physiology [7]. As a result of laboratory treatments performed with environmental pollutants, bacterial antigens and viral-like polynucleotides, 18,788 high-quality ESTs of *M. galloprovincialis *are now organized in a structured collection of 7,112 transcript sequences [37], named Mytibase and including most of the ESTs publicly available for the Mediterranean mussel (19,575 ESTs at Oct 2010).

In the absence of genomic information, this knowledge base offered us the unique opportunity to outline the available mussel immunome and develop a new microarray platform. In the following sections we present the most relevant Mytibase clusters and singletons related to mussel immunity and the validation of a species-specific Immunochip with hemocyte samples of *Vibrio-*injected mussels.

## Results and Discussion

### Identification of immune-related Mytibase sequences

The Mytibase descriptions report BLAST similarity searches, structured Gene Ontology vocabulary (GO) and identifiable protein features of the Interpro database (IPR) [38]. The latter, in particular, supported the characterization of unknown or poorly predicted sequences, and integrated the meaning of a substantial fraction of the mussel transcripts.

Not surprisingly, the Mytibase sequences (MGCs) are often defined by multiple IPRs with the notable exception of 588 ESTs codifying the mussel AMP that could only be recognized by similarity to prototype sequences of mytilin, myticin, mytimycin and defensin. Table 1 illustrates in decreasing order of abundance the first 15 of 1753 redundant IPRs present in the MGCs and the known mussel AMP. The protein motifs represented in Mytibase point to cell processes which are not restricted to the immune system as only 15% of the total IPRs directly refer to immunity. Nevertheless, the abundance of transcripts identifying AMP precursors or including domains such as Complement C1q (IPR001073) and the related Tumour Necrosis Factor-like (IPR008983), C-type lectin (IPR001304) and Fibrinogen, alpha/beta/gamma chain, C-terminal globular subdomain (IPR002181/IPR014716) definitely confirm that the Mytibase EST collection is particularly rich in immuno-related transcripts. Conversely, about 41% of the listed IPRs are exclusive of single clusters and singletons, with uncommon and intriguing protein motifs exemplified by IPR001398 (macrophage migration inhibitory factor, 4 ESTs in 3 clusters) and IPR012916 (RED-like protein, 6 EST in 1 cluster). The IPRs mentioned are easily found in Mytibase as Interpro key words.

**Table 1:** The first 15 Identifiable Protein Features and 4 Anti Microbial Peptide precursors, listed according to the EST abundance in Mytibase

IPR or AMP	ESTs	Description
IPR008983*	581	Tumour necrosis factor-like
IPR001073*	430	Complement C1q protein
Mytilin	277	Antimicrobial peptide precursor
Myticin	267	Antimicrobial peptide precursor
IPR001304	246	C-type lectin
IPR002048	187	Calcium-binding EF-hand
IPR012677	178	Nucleotide-binding, alpha-beta plait
IPR002181**	164	Fibrinogen, alpha/beta/gamma chain, C-terminal globular
IPR014716**	159	Fibrinogen, alpha/beta/gamma chain, C-terminal globular, subdomain 1
IPR000504	146	RNA recognition motif, RNP-1
IPR011992	133	EF-Hand type
IPR002035	132	von Willebrand factor, type A
IPR001254	129	Peptidase S1 and S6, chymotrypsin/Hap
IPR009003	129	Peptidase, trypsin-like serine and cysteine
IPR001314	124	Peptidase S1A, chymotrypsin
IPR003582	119	Metridin-like ShK toxin
IPR004000	119	Actin/actin-like
...	...	...
Defensin	28	antimicrobial peptide precursor
Mytimycin	16	antimicrobial peptide precursor

*, **: the paired cases examplify domains with overlapping architecture. See more on the protein signatures at http://www.ebi.ac.uk/interpro.

Since the genome of *M. galloprovincialis *is not available and sequence data are still limited, we applied a multiple search strategy to identify in Mytibase a relevant set of immune-related sequences. A low-stringency tBLASTn search allowed the extraction of 309 mussel sequences related to immune system processes (GO:0002376) and 1,021 sequences similar to those indexed in the multispecies catalogue ImmunomeBase (download permitted by C. Ortutay et al., Finland). Searches based on key words and manual screening yielded an additional 973 inputs and supported the final selection of 1820 mussel sequences, which can be regarded as an operational set and the starting point for the progressive authentication of immune-related candidates by transcriptional analyses and gene studies. Additional file 1 describes the selected MGCs and updates their functional annotation whereas the following paragraphs illustrate by abundance and putative function the most relevant ones to the mussel immunity.

### Transcripts identifying antimicrobial peptides

**Figure 1:**
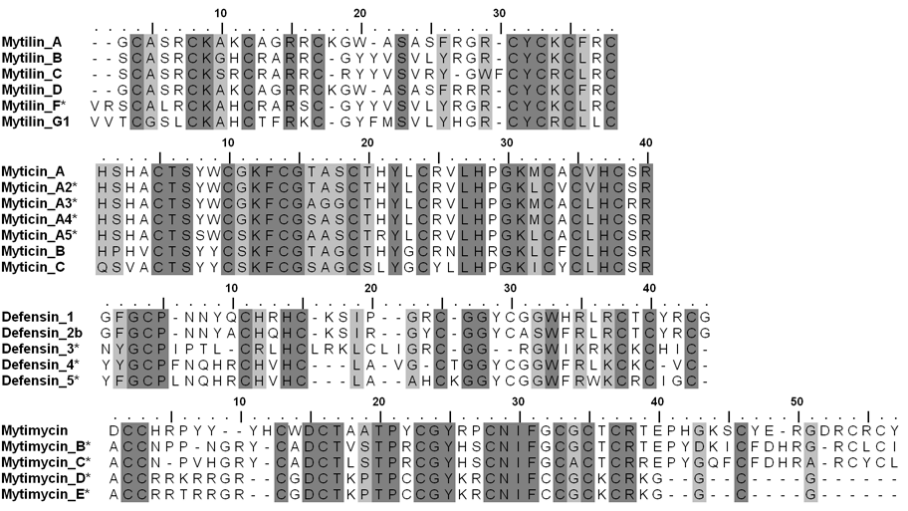
**Multiple alignment of the four AMP families identified in Mytibase**. According to Muscle alignment and BioEdit processing, each mussel AMP family includes new sequences indicated by an asterisk (*). Identical and similar (PAM250) residues are reported in dark and light grey, respectively.

Almost ubiquitous in the living species but highly diverse in structure and biological activity, host defence peptides interact with negatively charged cell membranes, lead to microbe killing and modulate both the innate and inducible antimicrobial responses in mammals [39]. Four groups of AMP are known in mussels: defensins, mytilins myticins and mytimycins [40,41]. The cationic and amphipatic structure of the mature peptides is stabilized by 4 intrachain disulphide bonds (6 in mytimycin) according to a unifying tridimensional motif [42]. Mytibase includes the full length precursor sequences of all the mussel AMP with some new variants: they are reported as mature peptide sequences in Figure 1.

Myticins are subdivided in A, B (96 aminoacid precursor) and the polymorphic type C (100 aminoacid precursor) [43]. Searching tBLASTn similarities to prototype sequences, we identified in Mytibase many precursors of myticin C (A7DWV6, 124 ESTs), myticin A (P82103, 88 ESTs) and myticin B (P82102, 55 ESTs). Robust non-synonymous SNPs analysis allowed us to split the sequence cluster of myticin A into 5 subgroups named A, A2, A3, A4 and A5, confirmed by 23, 38, 2, 21 and 4 sequence traces of high-quality, respectively (the latter three groups present indels in the 3'-UTR region).

Mytilin precursors are more heterogeneous in length ranging between 97 and 105 residues, and can be easily differentiated from the myticin precursors due to a different cysteine pattern. Similarly, we identified mytilin A (P81612, 5 ESTs), mytilin B (Q9Y0B1, 111 ESTs), mytilin C (Q5XWD7, 127 ESTs), mytilin D (B3VT96, 9 ESTs). We could also extend the sequence of Mytilin G1 (MGC00423, 14 ESTs) [41] and we propose MGC00659 (11 ESTs) as Mytilin F, namely a new mytilin component.

The defensin precursors identified in Mytibase are MGD1 (P80571, 20 ESTs), MGD2b (Q9U6U0, 5 ESTs) and three new sequences proposed as MGD3, MGD4, and MGD5. Due to the presence of a stop codon just after the 8th conserved cystein, defensins MGD3 and MGD4 are shorter than the others whereas MGD5 is the longest with 97 aminoacid residues.

Only one Mytibase EST (Mg_Nor01_39J03) corresponds to the mytimycin described in *M. edulis *(P81614) and four other sequences grouped from 4, 4, 4 and 3 ESTs may be regarded as new mytimycin variants. Curiously, two of these ESTs (Mg_Nor01_12C12, Mg_Nor01_39B01) display a long insertion in the 5' UTR and a signal peptide with maximal cleavage probabilities between positions 18-24 from ATG. cDNAs normalization was essential to reveal the rare mytimycin ESTs whereas the other more abundant AMP sequences can be easily and mainly attributed to hemocyte libraries prepared from immunostimulated Italian and Spanish mussels, without evidence of preferential geographical distribution (see in Mytibase the EST composition of Hae01-Hae05 and NOR01 libraries). All mussel AMP (27 clusters) and one hydramacin-like transcript (MGC2887) have been included in the Immunochip.

### Transcripts containing C1q and Tumour Necrosis Factor-like domains

The overlapping C1q (IPR001073) and TNF-like (IPR008983) domains have probably evolved by divergence from an ancient recognition molecule whose diversification could have started with urochordates and cephalocordates [44,45]. The large family of proteins with a C1q domain support many biological processes, from complement activation, modulatory immune functions, apoptotic cell clearance to coagulation, embryonic development and tissue homeostasis [46].

In mammals, the 18 polypeptide chains composing the complement subcomponent C1q are characterized by a short N-terminal region, a collagen-like Gly/Pro-rich tract and a C-terminal tulip-like structure of globular C1q domains (gC1q) also found in ficolins and other proteins. The C1q binding to immunoglobulins within immunocomplexes initiates the classic complement cascade and pathogen elimination. In the presence of Ca ions, the interaction of self and non-self ligands with charged gC1q residues causes gC1q reorientation and bending of the collagenous region. The activation signal is then transmitted to serine protease precursors (C1r, C1s) which, in turn, promote the proteolytic complement cascade and formation of a membrane attack complex [47]. Overall, the modularity and versatility of pattern recognition confirm the essential role of gC1q in both innate and acquired immune responses.

**Figure 2:**
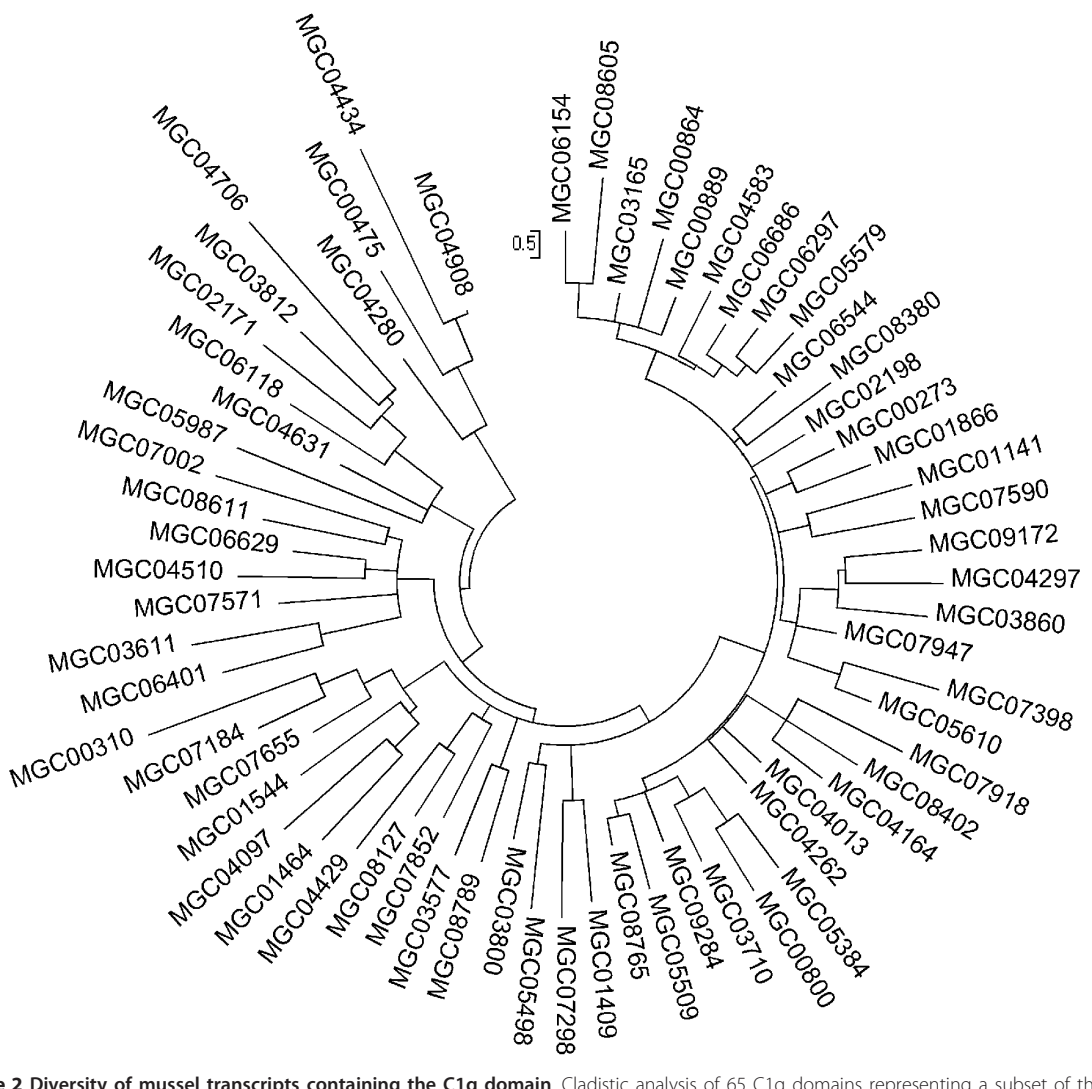
**Diversity of mussel transcripts containing the C1q domain**. Cladistic analysis of 65 C1q domains representing a subset of the 168 total sequences identified in Mytibase. This highly diversified non redundant subset was produced with NCBI BlastClust (-p F -L .5 -b T -S 40). The cladogram is based on the Neighbor-Joining method (1000 bootstrap replicates). The evolutionary distances were computed by using the JTT substitution matrix (number of amino acid substitutions for each of the 406 sites).

Several MGCs display sequence similarity to C1q, TNF, precerebellin, collagen and emilin proteins. Searching the TNF-like domain IPR008983 in Mytibase, we identified 146 transcripts, most of which are also characterized by the C1q domain IPR001073. Hidden Markov model analysis allowed the recognition of 22 additional C1q-containing sequences and the C1q motif was confirmed by manual validation in all 168 cases, without evidence of a true TNF domain. To illustrate their molecular diversity, a selection of the most divergent C1q-containing MGCs is reported in Figure 2. Many of them are similar to a sequence highly expressed in the mantle of the oyster *Pinctada fucata *[48] and some are very abundant, for instance MGC0284 with 99 out of 109 ESTs originating from hemocyte cDNAs [49]. In addition to the C-terminal globular domain, most of the predicted C1q proteins of *M. galloprovincialis *(163-293 aminoacid residues) have a short N-terminal signal peptide but lack central collagen-like repeats; hence, they should represent secreted gC1q receptor proteins expected to elicit chemotaxis and pathogen lysis via more ancient complement pathways [50]. The abundance and molecular diversity of the C1q-containing transcripts of *M. galloprovincialis *suggest pathogen-induced expansion of lectin-like PRR: the identification of related gene sequences will allow a comparison with the 32, 52 and 75 C1q gene models reported in *Homo sapiens, Danio rerio *and in the amphioxus *Brachiostoma floridae*, respectively [45,51]. The new microarray platform includes 162 of these mussel transcripts and also a few mussel transcripts similar to the complement component C3 (MGC07073 and MGC05748 display the alpha-2-macroglobulin complement component domain, IPR011626, and alpha-macroglobulin receptor binding domain, IPR009048, respectively) and a Membrane attack complex/perforin/C9 (IPR001862, MGC00636) expected to be involved in the pathogen lysis.

### Additional lectin-like and receptor-related transcripts

Protein-carbohydrate recognition is crucial to many cell processes and host-pathogen interactions. Lectins are membrane-associated and soluble proteins with specific carbohydrate recognition domains (CRD) which can either facilitate mutualistic interactions between host and microbiota or initiate innate and adaptive immune responses [52-54]. Acting as recognition receptors, lectins promote opsonization, phagocytosis and the activation of the complement system [25]. Structural and functional features distinguish eight to fifteen lectin groups largely related to immunity: C-type, S-type or glycan-binding galectins, I-type specific to sialic acids and glycoseaminoglycans also containing an Ig-like fold, pentraxins, fucolectins, fibrinogen-like lectins, ficolins, tachylectins and slug agglutinin, chitinase-like lectins, and orphan lectins. Transmembrane calnexins and soluble calreticulins support trafficking, sorting and maturation of glycoproteins whereas lectins localized in the plasma membrane or released into the extracellular matrix and body fluids mediate a broad range of processes including cell adhesion, cell signalling, pathogen recognition and endocytosis. Compared to more ancient lectins acting in the quality control of glycoproteins, extracellular lectins such as ficolins have evolved independently in the vertebrate and invertebrate lineages. The evolutionary radiation of these molecules emphasises the importance of the glycan code and lectin-ligand interactions in the immune responses and apoptotic cell clearance [55,56].

**Figure 3:**
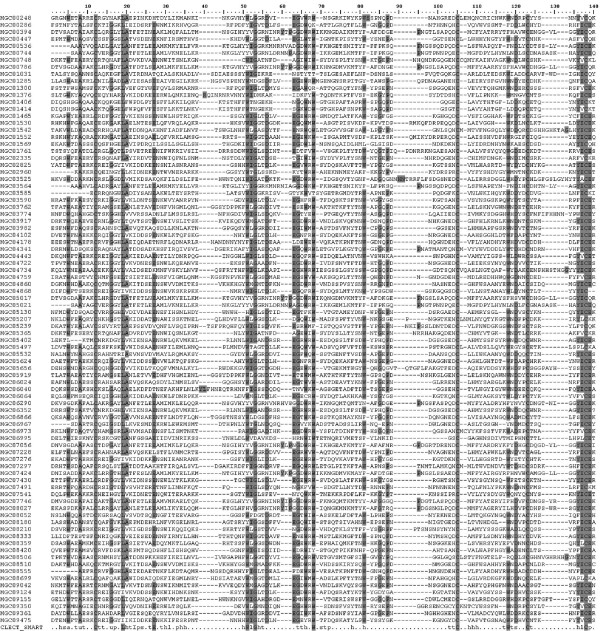
**Multiple alignment of 87 Mytibase sequences identified as C-lectins (IPR001304)**. The SMART consensus terms for the CLECT domain (SM00034) are shown at the bottom. Four cystein residues are entirely conserved (positions 12, 105, 123 and 138 in the multiple alignment). Other conserved (75%) residues are W5, A8, L19, W51, G53, G64, W66, W68, W118, D120, I137, C138 and E139. Positions with at least 50% of conservation are also shadowed.

Table 2 summarizes in decreasing abundance the lectin-like sequences identified in Mytibase by searching archetype lectin domains (IPRs). A total of 148 MGCs (429 ESTs) share the descriptive term 'lectin' as Interpro key-word. The most abundant and heterogeneous group refer to C-type lectins (IPR001304) originally named to reflect the importance of Ca+ in sugar binding. Many are similar to the nacre protein perlucin from *Haliotis laevigata *[57], while others remind of mammalian proteoglycans, type II receptors expressed particularly on macrophages and dendritic cells. For instance, among 9 MGCs the consensus MGC04167 is the most similar to the macrophage mannose receptor, protein involved in the glycoprotein endocytosis and antigen presentation, whereas 13 MGCs display similarity to the human DC-SIGN CD209 antigen [53]. Regardless of some conserved residues (e.g. cysteine and glycine) the remarkable sequence diversity of the C-type lectins expressed in mussels confirms them as candidate PRR (Figure 3). As a matter of fact, many of the *Caenorhabditis elegans *proteins containing a C-type lectin domain (278 genes) support pathogen-specific responses [58].

**Table 2:** Census of the Mytibase lectin-like transcripts (MGCs) by archetype IPRs

INTERPRO		MYTIBASE	
**ID**	**Description**	**MGCs**	**ESTs**

IPR001304	C-type lectin	104	246
IPR002181	Fibrinogen, alpha/beta/gamma chain, C-terminal globular	62	164
IPR013320	Concanavalin A-like lectin/glucanase, subgroup (related to galectins, S-type)	12	52
IPR006585	Fucolectin tachylectin-4 pentraxin-1 (F-type)	9	14
IPR013151	Immunoglobulin (I-type or siclecs)	7	9
IPR001223	Glycoside hydrolase, family 18, catalytic domain	4	53
IPR000922	D-galactoside/L-rhamnose binding SUEL lectin	4	17
IPR009169	Calreticulin	3	13
IPR001220	Legume lectin, beta chain	3	3
IPR009011	Mannose-6-phosphate receptor, binding (P-type)	2	4
IPR003990	Pancreatitis-associated protein	2	2
IPR008997	Ricin B-related lectin (R type)	1	13
IPR004043	LCCL	1	3
IPR005052	Legume-like lectin (L-type)	1	1

Each IPR can be referred to upstream- and downstream-related protein domains (e.g. IPR001304).

The second abundant lectin-like group recalls fibrinogen and fibronectin proteins and ficolins. Like the CRD of the mannose-binding lectins, the C-terminal fibrinogen-like domain (IPR002181) of ficolins has a bouquet-like structure which binds the carbohydrate residues of foreign and apoptotic cells (hence, pathogen opsonization, phagocytosis and clearance of dying cells) or in association with specific serine proteases (SP) initiates the proteolytic complement cascade and pathogen lysis [25,59]. Species-specific expansion of fibrinogen-related proteins (FREPs) has been reported in the snail *Biomphalaria glabrata *and the mosquito *Anopheles gambiae *[5,60]. In the crayfish *Pacifastacus leniusculus*, a protein containing the fibrinogen-like domain, but devoid of the hemagglutinating activity typical of vertebrate ficolins, acts as negative regulator of the prophenoloxidase system (proteolitic cascade similar to that of the complement system) and interferes with the transformation of quinone compounds to melanin [61].

Other MGCs point to galectins (IPR000922), I-type lectins (IPR013151) able to bind carbohydrate ligands via immunoglobulin-like domains, GH18 chitinase enzymes (IPR001223), L-type lectins (IPR005052) entailed in the intracellular protein sorting and P-type lectins (IPR009011), transmembrane proteins involved in the transport of lysosomal enzymes from the Golgi complex and the cell surface to lysosomes. For instance, chitinases are glycosyl hydrolases widely expressed from cnidarians to mammals, able to degrade the polysaccharide β-(1-4)-poly-N-acetyl D-glucosamine and confer protection against chitin-containing pathogens and parasites [62,63].

Mytibase is also rich in sequences with WD-40 repeats (IPR011046) and Leucin Rich Repeats (LRR, IPR001611). The modular organization of WD and LRR domains of vertebrate proteins sustains the diversity and plasticity of the apoptosome and inflammasome complexes in response to microbial products and metabolic stress, with the latter commonly signalled by ROS, nucleic acids, cathepsin and other molecules released by damaged cells [64,65]. In detail, the ligand binding to the carboxy-terminal LRR region of cytosolic receptors of the NOD-like family (NALPs/NLRs) can trigger receptor clustering, recruitment and activation of initiating caspases, release of IL-1R and IL18 citokines, inflammation and inflammatory cell death (pyroptosis).

Although many MGCs refer to nucleic acid binding proteins (IPR012677, IPR012340) or RNA/DNA binding helicases (IPR014021), further study is necessary to assign them an antiviral function typical of intracellular NOD-like and RIG-like helicase receptors or some membrane-bound TLRs [66]. With the possible exception of MGC02873, a Piwi-like singleton suggestive of silencing and regulative events in germ cells and hematopoietic stem cells, and putative RNA helicases of the DEAD-box family (IPR014014), we could not identify in Mytibase the core siRNA machinary Dcr-2, r2d2, AGO2 responsible for antiviral responses in *Drosophila *[67].

Keeping in mind the 222 and 72 TLR gene models identified in the genome of *Strongylocentrotus purpuratus *and *Branchiostoma floridae *[51,68], respectively, the occasional presence in Mytibase of TLR-related sequences (IPR000157, IPR004075) is disappointing. In fact, only MGC03952, MGC06978, MGC07535 and few other LRR-containing sequences display fragmentary similarity to human, fish and invertebrate TLR proteins. In the human TLRs, extracellular LRRs are arranged to recognize specific PAMPs whereas the intracellular Toll/Interleukin-1 receptor (TIR) domain activates downstream signalling pathways. According to a recent comparative overview, the identification of authentic invertebrate TLRs cannot rely on the sequence homology and requires functional studies [69].

Present in Mytibase are also putative Ig-like and MHC-related surface antigens (IPR013783, parent domain), sequences with a thyroglobulin domain (IPR000716) typical of Insulin-like Growth factor binding proteins and HLA class II invariant chain, and G-Protein-Coupled Receptors (IPR000276) involved in the transduction of various signals and accounting for about 3% of human genes [70].

Other MGCs are similar to von Willebrand Factor type C (IPR001007) found in plasma proteins promoting adhesion and thrombus formation at injured sites, Fasciclin-like proteins (IPR000782), Toll-InterLeukin Receptor (IPR000157), Speract/scavenger receptor (IPR001190), receptor-binding alpha macroglobulins (IPR009048), mannose-6-phosphate receptor (IPR009011) and TNF receptors (IPR001368: MGC05090, MGC06564).

### Transcripts involved in signalling and regulative networks

Not restricted to the innate immunity, cell signalling against fungal, bacterial and viral antigens occurs in insects through the Toll, Imd, Jak-STAT and P13K/Akt/TOR pathways. The first two are similar to the vertebrate TLR/IL and TNF signalling pathways, and interact with distinct NFkB factors to induce the expression of AMP and other molecules, whereas the inhibition of the nutrient signalling P13K/Akt/TOR can restrict viral replication by cell autophagy and reallocation of the resources from growth to immune defences [67].

Related to Toll/IL and TNF signalling are MGCs putatively identifying the LPS-induced TNF alpha factor or LITAF (IPR006629, 9 MGCs), TNF receptor associated factor TRAF (IPR008974, 4 MGCs), the adapter molecule MyD88 (MGC03566, MGC07770), Pellino which is known to associate with the kinase domain of the Pelle Ser/Thr kinase (MGC02650), NF_kB_inhibitor Cactus (MGC03934), a NFkB inhibitor-interacting Ras-like protein (MGC06766) and the transcription factor NFkB/Rel/Dorsal (IPR000451, IPR011539; MGC05614, MGC07242).

Definitely, many MGCs include the ankyrin repeat (IPR002110, 50 MGCs) typical of regulatory proteins but insufficient in itself to provide function recognition. Conversely, putative mussel kinases and phosphatases (e.g. IPR000340, IPR002290, IPR008343, IPR015731) support the existence of the mitogen-activated protein kinase (MAPK) signalling, whereas the EF-hand signature (IPR011992) and putative small G proteins (IPR003579, IPR003577, IPR005225) denote calcium regulated pathways. Putative zinc finger proteins (IPR000315, 49 MGCs), transcription factors bZIP-like (IPR004827), LIM-type (IPR001781), Jun-like (IPR002112), p53/RUNT-type (IPR012346) and repressors of transcription (IPR011991) reinforce the idea of multiple signalling pathways in mussels.

Interactions between protein kinase C, FAK and Src protein tyrosine kinases occur during the integrin-mediated spreading of *Lymnaea stagnalis *haemocytes [71] and robust intracellular signalling is essential to cytoskeleton remodelling, cell adhesion and migration of PAMP-activated haemocytes [26,72,73]. Although more than 60 MGCs contain a DNA-binding domain and some of them include the SH2 domain (IPR000980), there is no proof in Mytibase of a mussel JAK/STAT pathway, the main signalling system for a wide array of mammalian cytokines and growth factors. Nevertheless, the remarkable presence of a mussel Macrophage Migration Inhibitory Factor (3 MGCs), transcripts recalling Platelet-Derived Growth Factor (MGC01828, MGC07226), interferon-induced proteins (IPR009311, IPR004911), an interleukin enhancer binding factor (MGC05350), an interleukin-1-receptor-associated kinase (MGC00477) and G-protein coupled chemokine-like receptors, altogether evoke a regulatory humoral network able to reinforce mussel immunity. Unquestionably, Mytibase does not contain an IL17 homologue, found instead expressed in oyster hemocytes following bacterial stimulation [74].

Finally, MGCs denoting histone proteins (IPR005819, IPR009072), deacetylase (IPR000286, IPR003000) and acetyltransferase (IPR015418) enzymes confirm the importance of chromatin remodelling and histone modifications in the regulated transcription of effector genes.

### Transcripts related to oxidative stress and chaperon proteins

Scavenging and enzymatic activities protect the living cells from various stress factors, from endogenous reactive oxygen species (ROS) produced for instance by the mitochondrial respiratory chain to the oxidative burst consequent to pathogen recognition at the cell surface [75,76]. Partial or complete coding sequences of *M. galloprovincialis *super oxide dismutase (SOD), catalase, glutathione transferase, peroxisomal thiolase and polyamine oxidase have been reported [77-80]. In Mytibase, numerous MGCs putatively identify enzymes such as amine oxidases, dehydrogenases, peroxidases, mitochondrial oxidases and reductases. In addition to SOD (IPR001424, IPR001189, 4 MGCs) and glutathione peroxidases (IPR000889, 5 MGCs) many mussel sequences are featured by the thioredoxin fold/domain (IPR012336, IPR013766), typical of proteins regulating the redox state of cellular thiol groups such as the thioredoxin-like reductases (MGC09082).

Interestingly, more than 30 MGCs indicate heat shock proteins of different sizes (HSP 20, 40, 70 and 90 Kd) and related binding factors (MGC06041, MGC03865, MGC04512), mostly known to be modulated following immunostimulation [81-83].

### Transcripts identifying proteases, protease inhibitors and proteasome components

Proteases of various subfamilies and related inhibitors are essential in organism growth and development. Proteolytic reactions typically occur in the complement, coagulation and ProPO cascades, during apoptotic cell death, antimicrobial peptide synthesis and degradation of pathogen components within the lysosomal, cytosolic and extracellular compartments.

For instance, the insect clip-domain SP can act as cofactor or negatively regulate the melanization response, with a repertoire of 45 and 68 genes in *Drosophila melanogaster *and *Aedes aegypti*, respectively [5]. Cleavage of viral and host factors operated by granule-associated SP (granzymes) slows down viral replication and induces the apoptotic elimination of infected mammalian cells [70]. Caspases of the cysteine protease family also act in the proteolytic cascade of the apoptosis and, *via *NFkB signalling, regulate inflammatory responses in *Drosophila *[84].

Specific enzyme inhibitors are expected to modulate the same biological processes but also inhibit pathogen growth and invasive behaviour. In fact, trypsin and chymotrypsin inhibitor levels correlate with the plant resistance to pathogens, and in the basal metazoan *Hydra magnipapillata *the bactericidal activity of a kazal-type SP inhibitor possibly compensates the absence of migratory phagocytic cells [85,86].

In Mytibase, as much as 57 and 14 domains denote proteases/proteinases/peptidases and their inhibitors, respectively. Many MGCs indicate inherently secreted serine-type endopeptidases of the chymotrypsin/Hap family (IPR001254, 18 MGCs), SP inhibitors with Kazal-like repeats (IPR002350, 14 MGCs) or BIR repeats (IPR001370, 12 MGCs), with the latter belonging to the Inhibitor of Apoptosis (IAP) family [84]. Other MGCs point to cysteine caspase-like peptidases (IPR002398, 6 MGCs), astacin-like zinc metallopeptidases (IPR001506, 8 MGCs) and related inhibitors (I2, I8, I14/I15 hirudin/antistatin, I19, I29, I32, serpin and Tissue Inhibitors of MetalloProteases among others).

More than 60 MGCs denote ubiquitin, ubiquitin-related and proteasome-related components and give emphasis to intracellular processes oriented towards the pathogen elimination [87].

### Transcripts identifying lysozyme

Recognized in 1922 as an antibacterial molecule and abundant in various animal secretions, lyzozyme hydrolyzes 1,4-beta-linkages in peptidoglycan and chitodextrin structures. In flies and other invertebrates, lysozyme expression and activity increase after exposure to bacteria, and the species-specific gene number partly depends on the use of bacteria as food resource [5]. Up-regulation of the mussel lysozyme, with increased percentage of hemocytes expressing lysozyme mRNA, was observed at 2-3 days post-injection of *Vibrio anguillarum *or *Micrococcus lysodeikticus *[88] whereas maximum expression occurred after 3 hours in hemocytes immunostimulated *in vitro *[75]. In Mytibase, the 8 MGCs denoting lysozymes can mainly be classified in types C and G: among them, MGC02986 is similar to a C-type lysozyme described in insects but not yet reported in molluscs.

### Definition and validation of a *M. galloprovincialis *Immunochip

Owing to the continuous growth of the GenBank/UniProtKB/SwissProt databases, recurrent similarity searches and manual validation of the emerging similarities guided the progressive selection of 1,820 MGCs to be confirmed as components of the mussel immunome. Probes of 54-57 nucleotide length (plus 652 unrelated human probes) have been designed using the 3' end transcript region templates and spotted in four replicates to prepare a new DNA microarray platform, namely a *M. galloprovincialis *Immunochip.

Taking advantage of a large immunostimulation trial conducted *in vivo *on mussels from three different European regions [89] we selected and processed hemolymph samples collected at 3 and 48 hours after the injection of 10 million exponentially growing *Vibrio splendidus *cells into the adductor muscle. Total RNA was purified from two hemolymph pools (N = 10) per time point, and from paired saline-injected control mussels sampled at 3 and 48 h (N = 40, one unique reference pool). As the amplified Cy3/Cy5-labeled targets were competitively dye-swap tested on the mussel Immunochip, the reciprocal hybridizations of a target pair on quadruplicated probes yielded 8 fluorescence signals per probe (16 values per time point).

Looking at the total hybridization data set, 21.8% of the mussel probes gave significant fluorescence (median fluorescence values averaged per probe resulting above the background, i.e. average BG*2St.Dev.) with a range of 13.5-27.7% per individual array and average values of 17.2% and 26.4% lighted spots at 3 and 48 hours, respectively. These percentages reasonably relate to the number of differentially expressed genes (143 and 262, respectively) estimated by permutation from the absolute level and standard deviation of the replicates (SAM analysis, Table 3 and Additional files 2, 3 and 4). Soon after the immune stimulation, the over-expressed genes are consistently more numerous than the under-expressed (106 vs. 37 at 3 h), whereas later in time their proportion roughly equals (124 and 138 at 48 h). Converting the log_2 _(test/control) values by the relative fold change of expression, they range over two orders of magnitudes from +7.3 to -8.9 (at 3 h) and from + 7.6 to -9.6 (at 48 h).

**Table 3:** Total number of differentially expressed genes (over↑ under↓) in hemocytes sampled at 3 and 48 hours from mussels injected with live *V. splendidus*

Sampling time (hours post-injection)			Number of differentially expressed genes	FDR	Δ
					
	3		143 (106↑ 37↓)	1.30%	0.457
					
	48		262 (124↑ 138↓)	1.20%	0.534

False discovery rate (FDR) and delta value are also indicated (one class SAM, 16 replicates per time point).

**Figure 4:**
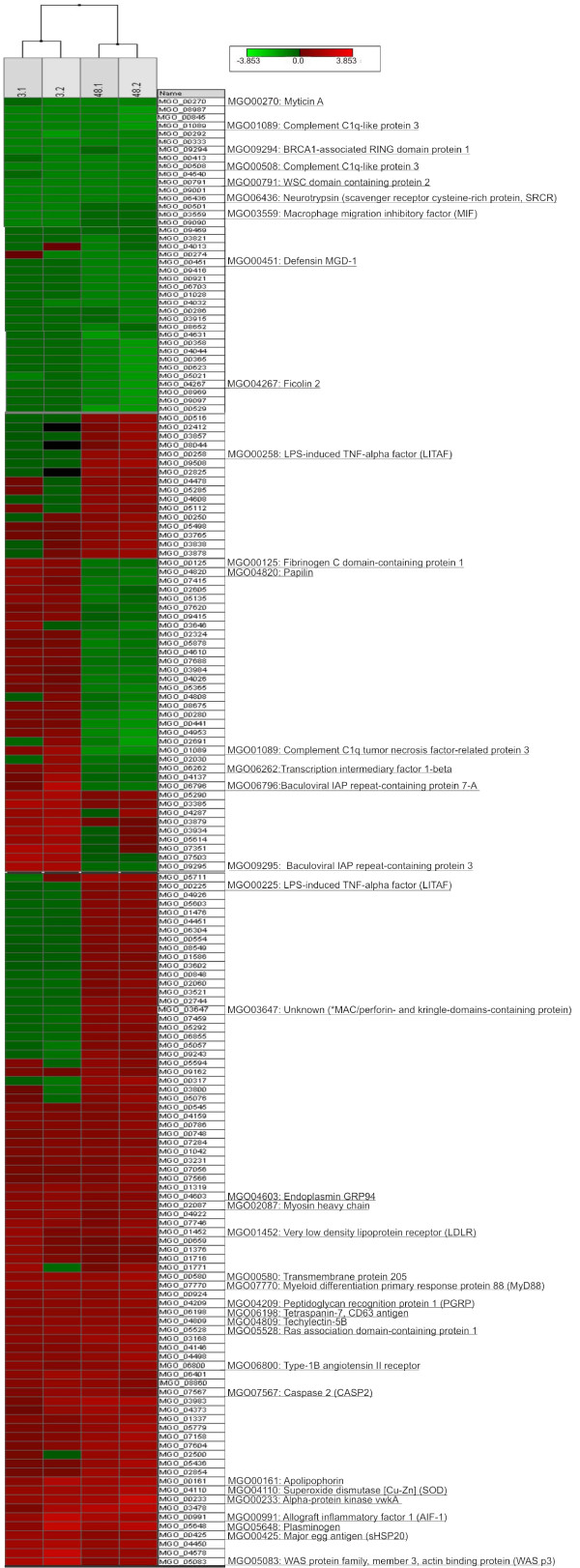
**Hierarchical clustering of the Immunochip profiles referring to 3 and 48 h post-injection of live *V. splendidus***. Two mussel groups with 8 replicates per time point have been analyzed (R-package software). Scale of the expression values and probe ID are indicated. For space reasons, only instructive parts of the clustered data are reported.

Hierarchical clustering of the Immunochip profiles clearly shows the resemblance between biological replicates (3.1 - 3.2 and 48.1 - 48.2), greater differences between the time points of 3 h and 48 h, and interesting discriminant signals such as those related to LITAF and IAP-like apoptosis inhibitors (Figure 4). The general AMP down-regulation detected in the hemocytes of *Vibrio*-injected mussels (for instance 4.7-6 fold down-regulation of defensin MGD1b at 3-48 h after challenge) confirms previous qPCR data [89]. Similarly, putative acute phase response proteins (apextrin) and the macrophage Migration Inhibitory Factor (MIF cytokine) were under-expressed. Conversely, probes pointing to Allograft inflammatory factor 1, SOD, small HSP20, plasminogen as well as various recognition receptors and molecules supporting intracellular signalling (e.g. MyD88) or cytoskeleton remodelling/motility (e.g. actin, myosin) were commonly up-regulated (Additional file 4). Compared to the early response, after 2 days we detected a significant expression of proteases and protease inhibitors, LITAF (TNF signalling) and sequences suggesting various cell functions (Additional file 3).

In general, no consistent trends could be defined for the C1q-like and lectin-like molecules. Due to their abundance and high sequence diversity, further study is necessary to understand their constitutive and PAMP-induced expression in mussel hemocytes.

**Figure 5:**
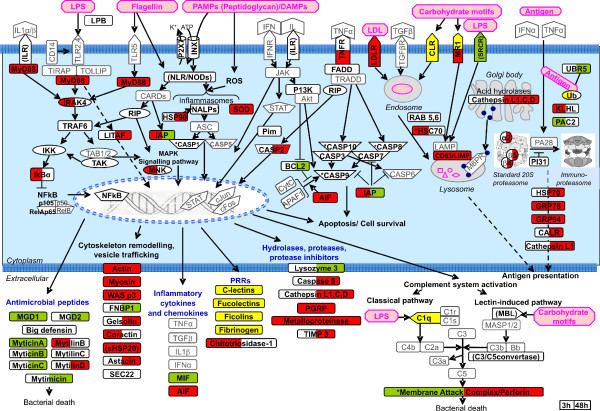
**Modelling the putative transcriptional hemocyte response to the *Vibrio *injection**. The model outlines PRR, interrelated signalling pathways and cell processes significantly modulated at 3 and 48 h post-treatment. Various bacterial PAMPs and DAMPs are indicated in pink. The gene functions represented on the Immunochip are indicated in bold according to sequence similarities/identities: framed bold characters on red, green and yellow fields indicate over-expression, under-expression and heterogeneous trends, respectively, of specific Immunochip probes at 3 h (left half) and 48 h (right half). Other gene functions not represented in Mytibase have been introduced to recall specific molecular pathways. Similarities resulting from InterproScan Analysis are reported in brackets whereas an asterisk indicates annotations based on manual inspection of relevant similarities. All the acronyms with related extended names are reported in the List of Abbreviations.

Based on the Immunochip hybridization data, the molecular pathways and gene functions mapping out the mussel hemocyte response to the *Vibrio *injection are modelled in Figure 5. Functionally similar to dendritic cells or macrophages, the mussel hemocytes display a pleiotropic response to the bacterial attack. Interacting with bacterial PAMPs, versatile and redundant recognition receptors undergo conformational changes, oligomerization or clustering. The subsequent activation of cross-talking signal transduction pathways adjusts the biochemical cell machinery towards the expression of specific gene sets and key effector molecules (in bold, on coloured field). Pathogen-induced oxidative burst and damage-associated molecular patterns (DAMPs) also sustain the inflammasome activation and intracellular signalling. Eventually, the endolysosomal and proteasome systems, secretory pathways and whole cell behaviour are recruited to achieve the pathogen killing.

### Overview of the mussel response to live *Vibrio splendidus*

The most ancient defences of the living organisms are based on neuropeptide and protein hormone receptors, receptor kinases and PRR (mainly TLRs, lectin-like, NLRs and RIG-like families) able to signal the danger and increase the expression of various inflammatory and effector molecules [90-92]. In view of the most recently sequenced invertebrate genomes, the pleiotropic innate immune responses could be described as a coordinated system of elements rapidly evolving and expanding the ability for pathogen sensing/targeting, and evolutionary conserved regulatory factors which finely adjust basic cell processes and direct the development and performance of the immune cells [5,51,93].

Ancient signalling pathways like those of MAPKs and NFkB are not exclusive of the immune responses and, not solved by standard sequence searching, the identification of invertebrate interleukine homologues (or molecules with functions analogous to the specialized signalling provided in vertebrates by interleukins and cytokines) makes new exploratory approaches necessary [9,74,94].

Although the hemolymph cells are fundamental in the mussel immunity, it is not clear if cells other than hemocytes contribute to the complex spectrum of rapid innate responses to potential pathogens. Consistent with a more general view linking immunity to metabolism and other body processes, typical immune genes and proteins should also be expressed in 'non-immune' cells, tissues and organs [2,41,95-97]. For instance, the expression of C1q/TNF-like molecules (MGC0284) has been detected in various tissues, with hemocytes showing the greatest levels, and throughout the development of *M. galloprovincialis *[49].

Similar to cells of the vertebrate monocyte/macrophage lineage, PAMP-activated immunocytes achieve pathogen elimination essentially through chemotaxis, phagocytosis, and cytotoxic processes [30]. In the Mediterranean mussel, agranular hemocytes are cells able to divide as they show replication-dependent chromosomal damage [98] whereas the heterogeneous and abundant granulocytes can be regarded as differentiated cells, mostly phagocytic and able to release antimicrobial peptides [41]. Accordingly, distinct hemocyte subpopulations appear to respond to potential pathogens with specific patterns of gene expression [88,99].

In addition to the host response, pathogen-related and physico-chemical factors are other main determinants of disease onset and mortality in aquacultured bivalves. The survival and niche occupation of *Vibrio *cells in changeable habitats (including the infected hosts) depend on the overall nutritional versatility of these bacteria, chemico-physical conditions for growth but also on the expression of hemagglutinins or lectins mediating the interaction with host cells and active secretions able to inhibit or disrupt the host defence reactions such as proteases, pore-forming hemolysins, ciliostatic and hemocyte-killer toxins [100-102]. As suggested for *V. harvey*, the modulation of signalling pathways essential to the antimicrobial immune response is an additional way to attack and escape the host response [103].

Testing the Immunochip performance with hemocytes sampled at 3 and 48 h from *Vibrio*-injected mussels revealed a general AMP downregulation, possibly related to the toxicity of live bacteria and contrasting the enhanced response to the stimulus obtained with heat-killed bacteria [43,75]. According to quantitative real time PCR assays performed on the hemolymph cells, the injection of control mussels with saline solution did not affect the expression of immune-relevant genes, namely mytilin B, myticin B, defensin, lysozyme and HSP70 [89]. The increase in transcriptional changes from 3 to 48 h and the slight prevalence of down-regulation signals at 48 h in the hemocytes of mussels injected with 10 million potentially infective *V.splendidus *cells mark an incoming functional decline. Indeed, a not negligible fraction of the *Vibrio*-injected mussels (22%) showed very slow or unapparent reactivity at 48 h (hence, considered close to death and discarded) whereas no mortality was observed at 3 h or in the control mussels injected with the saline solution only. As Spanish and French mussels injected in parallel with equal doses of live *Vibrio *cells in their respective locations did not show signs of distress [89] we suppose that season-related life history factors may underlie the overall reaction of these mussels to the injected bacteria. The delayed (48 h) over-expression of a number of proteases and stress proteins supports the functional hypothesis. Timing and complexity of the mussel immune response as well as the immunostimulation protocol (following shell notching, small mantle wounds may have attracted the hemocytes and induced their degranulation far from the adductor muscle) could also explain the progressive AMP down-regulation observed in the hemocytes of the *Vibrio*-challenged mussels. The HSPs (HSPs 20.6, 70 and 90 in particular) showed instead opposing expression trends with only a couple of probes for small HSPs down-regulated at 48 h post-challenge. These stress-inducible protein chaperons probably support pro-survival pathways but their multiple roles and complex expression patterns suggest further study [99,104]. In the same hemocyte samples, lectin-like and fibrinogen-like adhesion/recognition molecules showed heterogeneous expression trends whereas the frequent up-regulation of mussel genes relating to the cell shape and motility points to chemotactic and phagocytic hemocyte behaviour. The enhanced expression of LITAF and persistent MIF down-regulation in response to the injected bacteria encourage us to search regulatory mussel monokines with new immunostimulation trials and approaches other than DNA microarray testing.

The samples tested on the Immunochip exemplify only two temporal stages of the multi-step response to a reference dose of live *V. splendidus *cells. The observed transcriptional changes apparently mark the hemocyte activity against the *Vibrio *cells with a mounting inflammatory response (3 h) and a shift towards a more general stress condition (48 h). A previous equal treatment of *M. galloprovincialis *with live *V. splendidus*, caused a dramatic increase in living intra-hemocyte bacteria in less than an hour, suggestive of intense phagocytosis, and a subsequent gradual decrease with only a few viable bacteria at 24 h post-injection [105]. Recruited against active bacteria, the total counts of three distinct hemolymph cells (ialinocytes, small and large granulocytes) almost halved at 3 h post-injection and, after 48 h were still below the normal levels. Full understanding of the complex and dynamic response of *M. galloprovincialis *to the bacterial attack requires further study.

The great number of deep sea vent mussel transcripts made available during manuscript submission [106] and the launch of a new InterProScan Sequence Search interface (http://www.ebi.ac.uk/Tools/pfa/iprscan) will probably speed up the cross-species identification and validation of immune-related genes of marine bivalves. A partial comparison between Mytibase and the DeepSeaVent database (http://transcriptomics.biocant.pt:8080/deepSeaVent) rescued 5,261 annotated protein sequences expressed in both *M. galloprovincialis *and *Bathymodiolus azoricus *[106]. New BLASTN queries performed with the MGC transcript sequences significantly modulated at 3 and 48 h in the *Vibrio*-injected mussels against the 75,407 transcript sequences of *Bathymodiolus azoricus *confirmed the robustness of the Mytibase annotations (see Additional files 2 and 3). Such similarity searches also ascertained a not negligible sequence diversity of putative homologues (only 75 to 91% identical nucleotide positions in *M. galloprovincialis *and *B. azoricus *transcripts coupled by BLASTN e-value equal to 0.0) and the absence of typical AMP (the e-values obtained by querying the Mytibase AMP ranged from 0.97 to 5e-04). These findings, as well as previous comparative analysis of large EST sets from *M. californianus *and *M. galloprovincialis *[7,37], support the use of species-specific DNA microarrays.

## Conclusions

The great molecular diversification of pathogen binding molecules such as the insect Down syndrome cell adhesion molecule [107], snail FREPs [60], sea urchin TLRs [68] as well as the individual variant patterns reported for sea urchin 185/333 molecules [108]] and mussel myticins [43,109] emphasize the emerging complexity and divergent evolution of the invertebrate immune systems. Filter-feeding bivalves such as the *Mytilus *species commonly interact with a 'sea' of microscopic living forms, and can reveal interesting adaptations to co-evolving invaders and environmental changes. As many proteins involved in the immune responses also participate in basic cell processes, evolutionary adaptations differ between and within taxa and the *Mytilus *genomes are not yet available, the use of species-specific DNA microarrays represent a rational choice for studying transcriptional profiles and co-expression landscapes, and to validate many immune-related candidate molecules.

In fact, Mytibase includes almost all the domains featuring the innate PRR, i.e. C-type lectin and Ig-like domains, LRRs (and pyrin) domain, nucleotide-binding and Toll-Interleukin receptor domains, caspase recruitment and helicase domains [110], and reports abundance and diversity of the C1q/TNF-like, lectin-like and AMP mussel transcripts. Using the protein domains as instructive identifiers of sequence homology and other bioinformatics tools, we have designed 1,820 immune-candidate probes, organized them into a *M. galloprovincialis *Immunochip and tested this new DNA microarray with haemolymph samples exemplifying the early and late response to live *V. splendidus *cells. From one fifth to one fourth of the ImmunoChip probes gave significant fluorescence signals, respectively, and indicated both the modulation of various cell processes and a very specialized hemocyte transcriptome. Accordingly, the Immunochip could be confidently used to expand the validation of candidate probes on hemocytes and also in other mussel tissues. The putative relational map resulting from the Immunochip data certainly requires further study. In the meantime, a good number of Mytibase sequences relevant to the mussel immunity such as for instance the fibrinogen-like peptides are the object of new studies [111,112].

## Methods

### Identification of immune related mussel sequences in Mytibase

A multiple search strategy guided the extraction of putative immune-related sequences from Mytibase, the mussel transcript database [37]. We used 2,915 Gene Ontology (GO) sequences associated with UniProt Knowledgebase (UniProtKB) below the node GO:0002376-Immune system processes [113] and 4,216 sequences downloaded from the multispecies ImmunomeBase [2] to seek related mussel transcripts by tBLASTn similarity search (cut-off 1.0E-4). A working list of 1,233 keywords relating to mussels and innate immunity also supported the extraction of MytiBase sequences. Finally, BLAST similarities, gene ontologies and protein features reported in Mytibase were manually screened to confirm the core set of immune-related mussel transcripts.

### Descriptive analysis of selected sequence clusters

Selected immune sequence groups, mainly identified in Mytibase by textual search of Interpro domains [114] and/or BLAST similarity searches [115] were evaluated in more detail. The raw sequence traces identifying AMP and those containing the molecular signature of C-type lectin (IPR001304) and C1q (TNF-like) (IPR001073) were manually cleaned to perform multiple sequence alignment and compute phylogenetic trees by the Neighbour Joining with Bootstrap test. To multialign and validate the identification of AMP precursors and C1q domain containing sequences, we used different editors: Muscle [116], BioLign/BioEdit [117] and Jalview [118]. The C1q signature was confirmed by sequence homology search based on profile hidden Markov models (HMMER3) [119] whereas SignalP was used for prediction of signal peptide cleavage sites [120].

### Probe design and Immunochip preparation

One thousand and 820 oligonucleotide probes were designed with OligoArray 2.1 [121] on the selected MGCs according to the following requirements: 56.7 average length (54-57 range), 300 bases of distance between the oligo 5' end and transcript 3' end, 10-80% CG content, 70-92°C melting temperature with 65°C and 60°C as thresholds for cross-hybridization and hairpin formation, respectively. Additional 38 oligonucleotides (56-mers) with no virtual hybridization against the whole mussel EST collection were similarly designed using unrelated human sequences as templates. The designed probes were custom synthesized (Europe Services Invitrogen), arranged and deposited (BioRobotics MicroGrid II, Digilab, Genomic Solutions) on derivatized glass slides (MICROMAX Glass Slides: SuperChipTM I, Perkin-Elmer) at 50% relative humidity. The resulting species-specific Immunochip includes two equal arrays, each one organized in 16 subarrays and containing 4×1,820 mussel probes, 652 unrelated probes in multiple replicates and 112 alignment spots. Probe fixation on the slide was performed by UV cross linker (Stratagene) at a total power of 300 mJ. Slides were rinsed once in 1% SDS, 3× SSC for 1 min at room temperature, twice in distilled water for 5 min at room temperature, dried in laminar flux chamber and stored at room temperature under vacuum.

### Mussel challenge with *Vibrio splendidus*

Native mussels of commercial size (6.8 ± 0.7 cm lenght) from one outlet of the Venice lagoon (Italy, water temperature of 24-25°C) were acclimatized for one week in sea water collected at flood tide (32 psu, 22°C) and fed with *Isochrisis galbana*. Following careful shell notching, 0.1 ml of exponentially growing bacteria (10^7 ^Colony Forming Units of *V. splendidus *LGP32 in Trypsin-Casein-Soy medium) were injected into the posterior adductor muscle. One ml of hemolymph was withdrawn from individual mussels at 3 and 48 h post-injection and 10 hemolymph/group were pooled [89]. Hemolymph samples were similarly collected from paired control mussels injected with NaCl-enriched PBS (PBS-NaCl). Following centrifugation at 800x g, 4°C for 15 min, the pelleted hemocytes were re-suspended in 1 ml Trizol reagent (Invitrogen) and immediately stored at -80°C. Basically, two biological replicates per time point (3.1 and 3.2; 48.1 and 48.2) each one representing 10 individual hemolymphs were processed for hybridisation on the Immunochip in dye-swap combinations with a unique reference composed by all the hemolymphs sampled in parallel at 3 and 48 h from the control mussels (N = 40).

### RNA sample processing and microarray analysis

Total RNA from pooled hemolymph of treated and control mussels was extracted and additionally purified with high molar LiCl. RNA concentration and quality were ascertained by using the NanoDrop^® ^ND-1000UV spectrophotometer and Agilent 2100 Bioanalyzer (microcapillary electrophoresis on RNA 6000 Nano LabChips, Agilent Technologies). Equal amounts of 4 pooled hemolymph samples, representing 40 mussels injected with PBS-NaCl, were mixed to define one unique reference sample to be competitively hybridized on the Immunochip.

Hemolymph mRNA was linearly amplified from total RNA with the Message-Amp™ II aRNA Amplification kit (Ambion): 5-(3-aminoallyl)-UTP modified nucleotides were incorporated into the aRNA during the *in vitro *transcription reaction, then mono-functional NHS-esters of Cy3 or -Cy5 dyes (CyDye Post-Labeling Reactive Dye Pack, Amersham GE Healthcare) were resuspended in DMSO and covalently coupled to the aminoallyl-aRNA probes for 1 h at room temperature in the dark [122]. Following purification (Gene Elute PCR Clean-up kit, Sigma-Aldrich) and UV-quantification, 500 ng of both reference and test aaRNAs were combined and ethanol-precipitated. Cy3/Cy5-coupled samples were re-suspended in 18 μl of hybridization buffer (5x SSC, 50% formamide, 0.1% SDS), denaturated for 3 min at 70°C and competitively hybridised to the Immunochip for 24 h at 48°C in humidified dual-slide chamber (HybChamber, GeneMachines). Slides were first conditioned for 12 h at 48°C in a solution of 5x SSC, 100 ng/μl salmon sperm ssDNA, 5x Denhardt's solution and 0.1% SDS). Reference and test samples were then simultaneously hybridised in dye-swap crossed combinations on the 2 identical arrays of the same slide. The slides were sequentially washed at room temperature with mild shaking in buffer: 1x SSC, 0.2% SDS; 0.1x SSC, 0.2% SDS; 0.2x SSC (4 min each) and 0.1x SSC (3 min), with final drying by air flow.

### Microarray data analysis

Immunochip fluorescence signals were scanned using two lasers (633 nm and 543 nm) at 5 μm resolution with a GSI Lumonics LITE dual confocal laser scanner. Image processing and signal quantification were performed with the software ScanArray Express^® ^(PerkinElmer). Normalisation of the fluorescence signals was performed by using the total and LOWESS (Logfit) algorithm with MIDAS (MIcroarray Data Analysis System, http://www.tm4.org/midas.html) [123]. The log2 test/reference ratio of all the normalised fluorescence values was computed and the genes differentially expressed in the test sample *versus *control sample were identified by means of the Significance Analysis of Microarrays available from the Stanford University, CA (SAM software package v3.0, One-class analysis with 200 minimal permutations and FDR < 2%) [124,125]. Similarities among the Immunochip profiles were assessed by hierarchical clustering of the Pearson correlation similarity matrix (J-Express v2.1) [126].

## Mytibase home page and GEO accession numbers

The mussel knowledgebase is available following registration at http://mussel.cribi.unipd.it. The *Mytilus galloprovincialis *Immunochip (GPL10758) and related expression data (GSM575753, GSM575790, GSM577075-80) have been recorded at http://www.ncbi.nlm.nih.gov/geo.

## List of Abbreviations

**AIF**: Allograft Inflammatory Factor; **AKT**: RAC serine/threonine-protein kinase; **AMP**: Anti Microbial Peptides; **APAF1**: Apoptotic Peptidase Activating Factor 1; **ASC**: Apoptosis-associated Speck-like protein containing a CARD; **BCL2**: Baculoviral apoptosis regulator 2; **C1-5 **Complement component; **CALR**: Calreticulin; **CASP**: Caspase; **CD63/LIMP**: Tetraspanin-7 (lysosome membrane protein); **CLR**: C-type Lectin Receptor; **CLR**: C-type Lectin Receptor; **CRD**: Carbohydrate Recognition Domain; **DAMPs**: damage-associated molecular patterns; **EST**: Expressed Sequence Tag; **FADD**: FAS (TNFRSF)-associated via death domain; **FNBP1**: Formin-Binding Protein 1; **FREP**: Fibrinogen-Related Protein; **GO**: Gene Ontology; **GRP**: Glucose-Regulated Protein; **HSC70**: Heat Shock Cognate 70; **HSP**: Heat Shock Protein; **IAP**: Inhibitor of Apoptosis Protein; **IAP**: Inhibitor of Apoptosis Proteins; **Ig**: Immunoglobulin; **IKBα**: Inhibitor of nuclear factor Kappa-B kinase alpha; **IKK**: Inhibitor of nuclear factor Kappa-B Kinase complex; **IL**: InterLeukin; **INX**: Innexin; **IPR**: Identifiable PRotein feature based on the InterPro database; **IRAK4**: Interleukin Receptor-Associated Kinase 4; **JAK**: Janus kinase 1; **KLHL**: Kelch-like protein; **LAMP**: Lysosomal-Associated Membrane Protein; **LBP**: Lipopolysaccharide Binding Protein; **LDLR**: Low-Density Lipoprotein Receptor; **LITAF**: LPS-Induced TNF-Alpha Factor; **LPS**: LipoPolySaccharide; **LRR**: Leucine Rich Repeat; **M6PR**: Mannose 6 Phosphate Receptor; **MACPF**: Membrane Attack Complex/Perforin; **MAPKs**: Mitogen-Activated Protein Kinases; **MASP**: Mannan-binding lectin Serine Protease; **MBL**: Mannose Binding Lectin; **MGCs**: Mytibase consensuses or singletons (*M. galloprovincialis *transcripts); **MIF**: Migration Inhibitory Factor; **MNK**: MAP kinase-interacting serine/threonine-protein kinase; **MR1**: Mannose Receptor 1; **MyD88**: Myeloid Differentiation primary response gene 88; **NALP**: NATCH, LRR, and PYR containing proteins; **NCBI**: National Centre for Biotechnological Information; **NFkB**: Nuclear Factor of kappa light polypeptide gene enhancer in B-cells; **NLR**: NOD-Like Receptor; **NOD**: Nucleotide Binding Oligomerization Domain; **P13K**: Phosphatidylinositol-4,5-bisphosphate 3-Kinase; **P2X7**: ATP-gated ionotropic P2X purinoceptor subunit 7; **PA28**: Proteasome Activator subunit 28; **PAC2**: Proteasome Assembly Chaperone 2; **PAMP**: Pathogen Associated Molecular Pattern; **PGRP**: Peptidoglycan Recognition Protein; **PI31**: Proteasome Inhibitor PI31 subunit; **Pim**: proto-oncogene serine/threonine-protein kinase Pim; **ProPO**: ProPhenolOxidase; **PRR**: Pathogen Recognition Receptors; **RAB**: Ras-related gtp-Binding protein; **RIG**: Retinoic acid-Inducible Gene-I; **RIP**: Receptor-Interacting serine-threonine kinase; **RLR**: RIG-Like Receptor; **ROS**: Reactive Oxygen Species; **SEC22**: vesicle transport protein SEC22; **SOD**: SuperOxide Dismutase; **SP**: serine proteases; **SRCR**: Scavenger Receptor Cysteine-Rich protein precursor; **TAB**: TAK-binding protein; **TAK**: mitogen-activated protein kinase kinase; **TIMP3**: Tissue Inhibitors of MetalloProteinase 3; **TIR**: Toll/Interleukin-1 Receptor; **TIRAP**: Toll-Interleukin Receptor (TIR) domain containing Adaptor Protein; **TLR**: Toll-Like Receptor; **TNF**: Tumour Necrosis Factor; **TNFR**: Tumour Necrosis Factor Receptor; **TOLLIP**: TOLL-Interacting Protein; **TRADD**: TnfRsf-Associated via Death Domain; **TRAF6**: TNF receptor-associated factor 6; **Ub**: Ubiquitin; **UBR5**: Ubiquitin protein Ligase E3 (component n-recognin 5); **UniProtKB**: UniProt Knowledgebase; **α2**: proteasome subunit alpha type-2; **β4, β5**: Proteasome subunit beta type-4,-5.

## Authors' contributions

LV and UR performed RNA purification and processing, microarray experiments and data analysis, LV also supported the overall interpretation. FB (former Mytibase curator) designed the oligonucleotides and performed the AMP analysis. GL assured the equipment and supervised the microarray work (http://microcribi.cribi.unipd.it). BC and CM prepared the Immunochip slides using custom-synthesized oligonucleotides. AP guided the Mytibase annotations, carried out specific sequence analysis and supported manuscript development. FR, AF and BN provided valuable inputs for Immunochip definition and text improvements (AF partially screened the 1820 Mytibase transcripts). PV performed the mussel treatment, evaluated the Mytibase sequences for the Immunochip definition, drafted and wrote the manuscript. All authors read and approved the final manuscript.

## Supplementary Material

Additional file 1 **The 1820 putative immune-related sequences selected from Mytibase**. From left to right: ID and sequence data; first-hit similarities resulting from BLAST searches vs. UniProt/SW database; IPR domains from InterproScan analysis and GO terms; KEGG biochemical pathways and EC enzyme nomenclature based on BLAST similarity searches vs. annotated subsets of EMBL UniProtKB. Click here for file

Additional file 2 **Differentially expressed genes in mussel hemocytes at 3 h post-injection of live *V. splendidus***. Probe ID, sequence information and ordered expression values (log2 of normalized test/control values) are reported. Similarities resulting from InterproScan Analysis are reported in brackets (* annotation based on manual inspection of other relevant similarities) Click here for file

Additional file 3 **Differentially expressed genes in mussel hemocytes at 48 h post-injection of live *V. splendidus***. Probe ID, sequence information and ordered expression values (log2 of normalized test/control values) are reported. Similarities resulting from InterproScan Analysis are reported in brackets (* annotation based on manual inspection of other relevant similarities) Click here for file

Additional file 4 **Common differentially expressed genes in mussel hemocytes at 3 and 48 h post-injection of live *V. splendidus***. Probe ID, sequence information and ordered expression values (log2 of normalized test/control values). Similarities resulting from InterproScan Analysis are reported in brackets (* annotation based on manual inspection of other relevant similarities) Click here for file
