# Supplementary material for: Insights into the innate immunity of the Mediterranean mussel Mytilus galloprovincialis
Source: BMC Genomics. 2011 Jan 26;12:69. doi: 10.1186/1471-2164-12-69 (PMC3039611; doi:10.1186/1471-2164-12-69)
Supplement: Additional file 1 — The 1820 putative immune-related sequences selected from Mytibase. From left to right: ID and sequence data; first-hit similarities resulting from BLAST searches vs. UniProt/SW database; IPR domains from InterproScan analysis and GO terms; KEGG biochemical pathways and EC enzyme nomenclature based on BLAST similarity searches vs. annotated subsets of EMBL UniProtKB. [file 1471-2164-12-69-S1.PDF]

Additional file 1

Sequence information			Sequence similarity annotation					
**Mytibase ID**	**Clustered ESTs**	**EST lenght**	**BLAST X (or BLAST N)**	**InterPro signature**	**Gene Ontology**	**KEGG**	**EC nomenclature**
MGC00001	2	738	Perlucin	C-type lectin-like ; C_TYPE_LECTIN_1 ; C-TYPE LECTIN SUPERFAMILY MEMBER ; Lectin_C ; GALACTOSE-SPECIFIC C-TYPE LECTIN ; C_TYPE_LECTIN_2	GO:0009897_C_external side of plasma membrane; GO:0002925_P_positive regulation of humoral immune response mediated by circulating immunoglobulin;	K06468_04640_Hematopoietic cell lineage;	
MGC00008	1	747	Non-structural maintenance of chromosomes element 1 homolog	NON-SMC ELEMENT 1-RELATED ; SMC_Nse1 ; Cysteine-rich domain ; zf-RING-like ; coiled-coil			
MGC00011	22	902	Cysteine proteinase inhibitor 6	Cystatin/monellin ; CYSTATIN			
MGC00019	4	1036	6-phosphogluconolactonase	NagB/RpiA/CoA transferase-like ; pgl: 6-phosphogluconolactonase ; 6-PHOSPHOGLUCONOLACTONASE ; Glucosamine_iso	GO:0006098_P_pentose-phosphate shunt;	K01057_00030_Pentose phosphate pathway;	3.1.1.31_6-phosphogluconolactonase.;
MGC00022	1	745	RING finger protein 185	RING/U-box ; ZF_RING_1 ; zf-C3HC4 ; ZF_RING_2 ; RNF5			
MGC00023	2	765	Neurogenic locus notch homolog protein 1	EGF/Laminin ; NOTCH ; EGF-LIKE DOMAIN PROTEIN ; EGF ; EGF_3 ; Snake toxin-like ; EGF_1 ; EGF_2		K02599_04320_Dorso-ventral axis formation; K02599_04330_Notch signaling pathway;	
MGC00027	19	691	Caprin-2	C1q ; GLIACOLIN-RELATED ; CEREBELLIN-RELATED ; COMPLEMNTC1Q ; TNF-like ; C1Q		K03987_04610_Complement and coagulation cascades; K03987_05010_Alzheimers disease;	
MGC00028	6	735	Agrin	SPARC (OSTEONECTIN) ; Kazal_1 ; Kazal-type serine protease inhibitors ; coiled-coil	GO:0005605_C_basal lamina; GO:0043113_P_receptor clustering; GO:0005515_F_protein binding; GO:0005516_F_calmodulin binding;	K04661_04350_TGF-beta signaling pathway; K06254_04512_ECM-receptor interaction;	
MGC00041	2	927	Protein LITAF homolog	LIPOPOLYSACCHARIDE-INDUCED TRANSCRIPTION FACTOR REGULATING TUMOR NECROSIS FACTOR ALPHA			
MGC00045	1	744	Neuroglian	Immunoglobulin ; CELL ADHESION MOLECULE ; IMMUNOGLOBULIN DOMAIN SUPERFAMILY (SENSORY GUIDANCE PROTEIN) ; IG_LIKE ; ig		K06756_04514_Cell adhesion molecules (CAMs); K06757_04514_Cell adhesion molecules (CAMs);	2.7.10.1_Receptorprotein-tyrosinekinase.; 1.11.1.7_Peroxidase.;
MGC00054	8	1340	Meprin A subunit alpha	MAM DOMAIN-CONTAINING GLYCOSYLPHOSPHATIDYLINOSITOL ANCHOR PROTEIN 1 ; Galactose-binding domain-like ; Metalloproteases ('zincins'), catalytic domain ; APICAL ENDOSOMAL GLYCOPROTEIN PRECURSOR. ; Astacin ; ASTACIN ; MAM_2 ; MAM			3.4.24.21_Astacin.; 3.4.24.18_MeprinA.;
MGC00055	18	740	Pleiotrophin	PTN_MK_C ; Midkine ; SUBcoiled-coil			
MGC00060	31	2033	Chitotriosidase-1	Glycosidase; hydrolase	GO:0005615_C_extracellular space; GO:0008061_F_chitin binding; GO:0006032_P_chitin catabolic process; GO:0004568_F_chitinase activity; GO:0006955_P_immune response; GO:0009617_P_response to bacterium;	K01183_00530_Aminosugars metabolism;	3.2.1.14_Chitinase.;
MGC00062	2	337	Serine protease inhibitor Cvsi-2	Serine protease inhibitor			
MGC00066	1	723	Thyroglobulin	INSULIN-LIKE GROWTH FACTOR BINDING PROTEIN ; Thyroglobulin_1 ; Thyroglobulin type-1 domain ; INSULIN-LIKE GROWTH FACTOR BINDING PROTEIN 3 ; THYROGLOBULIN_1_2	GO:0005576_C_extracellular region; GO:0007165_P_signal transduction;	K10809_05320_Autoimmune thyroid disease;	
MGC00068	12	893	Multiple epidermal growth factor-like domains protein 6				

MGC00069	8	1331	Meprin A subunit alpha	MAM DOMAIN-CONTAINING GLYCOSYLPHOSPHATIDYLINOSITOL ANCHOR PROTEIN 1 ; Galactose-binding domain-like ; Metalloproteases ('zincins'), catalytic domain ; APICAL ENDOSOMAL GLYCOPROTEIN PRECURSOR. ; Astacin ; MAM_2 ; MAM			3.4.24.18_MeprinA.;
MGC00073	29	1107	Cathepsin L	Cysteine proteinases ; THIOL_PROTEASE_CYS ; Inhibitor_I29 ; Peptidase_C1 ; PAPAIN ; THIOL_PROTEASE_HIS ; Q8MNZ7_EEEEE_Q8MNZ7; ; CYSTEINE PROTEASE FAMILY C1-RELATED ; CATHEPSIN L ; THIOL_PROTEASE_ASN		K01365_04612_Antigen processing and presentation;	3.4.22.15_CathepsinL.;
MGC00079	1	757	Ankyrin repeat and sterile alpha motif domain-containing protein 1B	ANKYRIN ; Ank ; ANKYRIN REPEAT-CONTAINING ; ANK_REPEAT ; ANK_REP_REGION ; Ankyrin repeat		K06694_03050_Proteasome; K08803_05219_Bladder cancer;	2.7.11.1_Non-specificserine/threonineproteinkinase.; 2.4.2.30_NAD(+)ADP-ribosyltransferase.;
MGC00081	4	489	Unknown				
MGC00087	24	686	Myticin-A				
MGC00099	52	803	60S ribosomal protein L15	Ribosomal proteins L23 and L15e ; Ribosomal_L15e ; RIBOSOMAL_L15E ; RIBOSOMAL PROTEIN L15		K02877_03010_Ribosome;	
MGC00108	6	1151	Dolichyl-diphosphooligosaccharide--protein glycosyltransferase subunit 2	RIBOPHORIN II ; Ribophorin_II		K00730_00510_N-Glycan biosynthesis; K00730_01030_tba;	2.4.1.119_Dolichyl-diphosphooligosaccharide--proteinglycotransferase.;
MGC00125	1	464	Fibrinogen C domain-containing protein 1	FIBRINOGEN AND FIBRONECTIN ; Fibrinogen C-terminal domain-like ; Fibrinogen_C		K06252_04510_Focal adhesion; K06252_04512_ECM-receptor interaction;	
MGC00135	2	502	Prostasin	TRYPSIN_HIS ; SERINE PROTEASE-RELATED ; Trypsin ; TRYPSIN_SER ; SERINE PROTEASE-RELATED, INSECT ; TRYPSIN_DOM ; Trypsin-like serine proteases	GO:0005615_C_extracellular space; GO:0008236_F_serine-type peptidase activity; GO:0005576_C_extracellular region; GO:0005886_C_plasma membrane;		3.4.21.59_Tryptase.; 3.4.21.35_Tissuekallikrein.; 3.4.21.27_CoagulationfactorXIa.; 3.4.21.4_Trypsin.;
MGC00139	2	630	Unknown				
MGC00141	2	546	Calmodulin	EF_HAND_1 ; CALMODULIN ; Q40982_PEA_Q40982; ; EF_HAND_2 ; CALFLAGIN ; EF-hand ; CALCIUM BINDING PROTEIN ; efhand			2.7.11.1_Non-specificserine/threonineproteinkinase.;
MGC00145	80	1663	Elongation factor 1-alpha	EF-Tu/eEF-1alpha/eIF2-gamma C-terminal domain ; TRANSLATION FACTOR ; EF-1_alpha: translation elongation factor ; GTP_EFTU_D2 ; P-loop containing nucleoside triphosphate hydrolases ; ELONGATNFCT ; Translation proteins ; GTP_EFTU_D3 ; ELONGATION FACTOR 1-ALPHA (EF-1-ALPHA) ; EFACTOR_GTP ; GTP_EFTU			2.7.7.4_Sulfateadenylyltransferase.; 2.7.1.25_Adenylyl-sulfatekinase.;
MGC00153	75	861	60S ribosomal protein L7a	RIBOSOMALL7A ; L30e-like ; 60S RIBOSOMAL PROTEIN L7A ; RIBOSOMAL_L7AE ; RIBOSOMAL PROTEIN L7AE FAMILY MEMBER ; L7ARS6FAMILY ; Ribosomal_L7Ae		K02936_03010_Ribosome;	
MGC00156	2	515	Protocadherin gamma-A8	Cadherin-like ; CADHERIN_2 ; Cadherin ; CADHERIN-RELATED ; CADHERIN		K06797_04514_Cell adhesion molecules (CAMs);	
MGC00161	36	1197	Apolipophorins	EXTRACELLULAR MATRIX GLYCOPROTEIN RELATED ; VWD ; coiled-coil ; VWFD		K03900_04510_Focal adhesion; K03900_04512_ECM-receptor interaction; K03900_04610_Complement and coagulation cascades;	
MGC00167	13	622	Thymosin beta-4	Thymosin ; THYMOSIN_B4		K05764_04810_Regulation of actin cytoskeleton;	

MGC00175	25	1579	Tubulin beta chain	Tubulin_C ; Tubulin ; BETATUBULIN ; TUBULIN_B_AUTOREG ; TUBULIN BETA CHAIN ; Tubulin nucleotide-binding domain-like ; Tubulin C-terminal domain-like ; coiled-coil ; TUBULIN		K07375_04540_Gap junction;	
MGC00177	17	649	60S ribosomal protein L23a	Ribosomal proteins L23 and L15e ; Ribosomal_L23 ; RIBOSOMAL_L23 ; Ribosomal_L23eN ; 60S RIBOSOMAL PROTEIN L23A		K02893_03010_Ribosome;	
MGC00187	22	873	Fibrinolytic enzyme, isozyme C	TRYPSIN_HIS ; SERINE PROTEASE-RELATED ; Trypsin ; TRYPSIN_SER ; CHYMOTRYPSIN ; SERINE PROTEASE-RELATED, INSECT ; TRYPSIN_DOM ; Trypsin-like serine proteases			3.4.21.59_Tryptase.; 3.4.21.1_Chymotrypsin.; 3.4.21.4_Trypsin.;
MGC00189	8	1242	Cathepsin B	Cysteine proteinases ; CATHEPSIN B ; CATB_BOVIN_P07688; ; THIOL_PROTEASE_CYS ; Peptidase_C1 ; PAPAIN ; THIOL_PROTEASE_HIS ; CYSTEINE PROTEASE FAMILY C1-RELATED ; Propeptide_C1 ; THIOL_PROTEASE_ASN		K01363_04612_Antigen processing and presentation;	3.4.22.1_CathepsinB.;
MGC00197	1	658	Uncharacterized gene 48 protein				
MGC00210	4	762	SUMO-conjugating enzyme UBC9	UBIQUITIN_CONJUGAT_2 ; UBIQUITIN-CONJUGATING ENZYME E2 I ; UBIQUITIN-CONJUGATING ENZYME E2 ; UBC-like ; UQ_con ; O62622_DROME_O62622; ; UBIQUITIN_CONJUGAT_1		K10577_04120_Ubiquitin mediated proteolysis;	6.3.2.19_Ubiquitin--proteinligase.;
MGC00220	29	802	Soma ferritin	FERRITIN_LIKE ; FERRITIN_2 ; FERRITIN ; FERRITIN_1 ; Ferritin ; Ferritin-like ; Q7YW83_EEEEE_Q7YW83;		K00522_00860_Porphyrin and chlorophyll metabolism;	1.16.3.1_Ferroxidase.;
MGC00222	22	1090	Collagen alpha-2(XI) chain				
MGC00225	2	785	Lipopolysaccharide-induced tumor necrosis factor-alpha factor homolog	LIPOPOLYSACCHARIDE-INDUCED TRANSCRIPTION FACTOR REGULATING TUMOR NECROSIS FACTOR ALPHA			
MGC00227	126	554	Mytilin-B				
MGC00230	12	806	Putative gastrointestinal growth factor xP4	INTESTINAL TREFOIL FACTOR-RELATED ; Trefoil ; PTREFOIL ; P_TREFOIL ; TREFOIL FACTOR 1		K01187_00052_Galactose metabolism; K01187_00500_Starch and sucrose metabolism;	3.2.1.20_Alpha-glucosidase.;
MGC00233	2	803	Alpha-protein kinase vwkA	ALPHA KINASE/ELONGATION FACTOR 2 KINASE ; VWFA ; HEAT SHOCK 70 KDA PROTEIN 12 ; vWA-like ; VWA ; VWFADOMAIN			
MGC00234	20	710	Peptidyl-prolyl cis-trans isomerase	PEPTIDYL-PROLYL CIS-TRANS ISOMERASE F, PPIF ; CSA_PPIASE_1 ; Cyclophilin-like ; CSAPPISMRASE ; CYCLOPHILIN ; Pro_isomerase ; CSA_PPIASE_2			5.2.1.8_Peptidylprolylisomerase.;
MGC00238	1	585	Caprin-2	C1q ; GLIACOLIN-RELATED ; CEREBELLIN-RELATED ; COMPLEMNTC1Q ; TNF-like ; C1Q	GO:0005737_C_cytoplasm; GO:0005739_C_mitochondrion; GO:0030308_P_negative regulation of cell growth;	K03986_04610_Complement and coagulation cascades; K03986_05010_Alzheimers disease; K03987_04610_Complement and coagulation cascades; K03987_05010_Alzheimers disease;	
MGC00248	1	671	Perlucin	C-type lectin-like ; C_TYPE_LECTIN_1 ; C-TYPE LECTIN SUPERFAMILY MEMBER ; Lectin_C ; GALACTOSE-SPECIFIC C-TYPE LECTIN ; ANTIFREEZEII ; C_TYPE_LECTIN_2			
MGC00249	4	357	Unknown				

MGC00250	17	605	Caprin-2	C1q ; CEREBELLIN-RELATED ; COMPLEMNTC1Q ; TNF-like ; C1Q	GO:0007399_P_nervous system development; GO:0007268_P_synaptic transmission;		
MGC00258	3	792	Protein LITAF homolog	LIPOPOLYSACCHARIDE-INDUCED TRANSCRIPTION FACTOR REGULATING TUMOR NECROSIS FACTOR ALPHA			
MGC00262	6	970	SH3 domain-binding protein 2	SH3-BINDING ; PH ; SUBPH_DOMAIN ; PH domain-like	GO:0005070_F_SH3/SH2 adaptor activity; GO:0007165_P_signal transduction;	K07984_04650_Natural killer cell mediated cytotoxicity;	
MGC00270	70	755	Myticin-A				
MGC00272	2	806	Fibrinogen C domain-containing protein 1	FIBRINOGEN AND FIBRONECTIN ; Fibrinogen C-terminal domain-like ; Fibrinogen_C ; FIBRIN_AG_C_DOMAIN		K06252_04510_Focal adhesion; K06252_04512_ECM-receptor interaction;	
MGC00273	25	658	Heavy metal-binding protein HIP	C1q ; CEREBELLIN-RELATED ; COMPLEMNTC1Q ; TNF-like ; C1Q			
MGC00274	12	777	Ficolin-2	RCC1_2 ; FIBRINOGEN AND FIBRONECTIN ; Fibrinogen C-terminal domain-like ; Fibrinogen_C ; FIBRIN_AG_C_DOMAIN			
MGC00276	6	1245	Protein BTG1	B-CELL TRANSLOCATION GENE 1 BTG1 ; BTG_2 ; B-CELL TRANSLOCATION GENE ; BTG ; ANTIPRLFBTG1			
MGC00277	9	818	Cubilin	BONE MORPHOGENETIC PROTEIN 1 ; CUB ; DISCOIDIN, CUB, EGF, LAMININ , AND ZINC METALLOPROTEASE DOMAIN ; Spermadhesin, CUB domain			3.4.24.19_ProcollagenC-endopeptidase.; 3.4.21.120_Oviductin.; 3.4.24.21_Astacin.;
MGC00278	1	472	Probable G-protein coupled receptor 83	G_PROTEIN_RECEP_F1_2 ; G-PROTEIN COUPLED RECEPTOR ; G_PROTEIN_RECEP_F1_1 ; GPCRRHODOPSN ; 7tm_1 ; Family A G protein-coupled receptor-like	GO:0009582_P_detection of abiotic stimulus; GO:0004930_F_G-protein coupled receptor activity; GO:0005887_C_integral to plasma membrane; GO:0007186_P_G-protein coupled receptor protein signaling pathway;		
MGC00280	18	481	Defensin MGD-1 (Fragment)	Defensin_2 ; Scorpion toxin-like ; ARTHROPOD_DEFENSINS			
MGC00284	109	637	Unknown (C1Q, TNF-like)	C1Q, TNF-like			
MGC00286	9	528	Hepatic lectin	C-type lectin-like ; C-TYPE LECTIN SUPERFAMILY MEMBER ; Lectin_C ; GALACTOSE-SPECIFIC C-TYPE LECTIN ; C_TYPE_LECTIN_2		K06468_04640_Hematopoietic cell lineage;	
MGC00288	4	766	Metalloproteinase inhibitor 3	TIMP ; NTR ; TIMP-like ; METALLOPROTEASE INHIBITOR			
MGC00292	14	813	Unknown				
MGC00294	135	538	Mytilin-C				
MGC00295	1	699	Putative ferric-chelate reductase 1	SUBReeler ; REELIN			
MGC00297	29	785		Secreted; Repeat; glycoprotein			
MGC00298	1	397	Heat shock 70 kDa protein	HEAT SHOCK PROTEIN 70KDA ; HEAT SHOCK PROTEIN 70 (HSP70) ; Q8I0E9_DROME_Q8I0E9; ; HSP70_1 ; HSP70 ; Actin-like ATPase domain ; HEATSHOCK70		K03283_04010_MAPK signaling pathway; K03283_04612_Antigen processing and presentation;	
MGC00299	10	891	Unknown				
MGC00300	145	690	Myticin-C				
MGC00301	11	794	Stress-induced protein 1	SMALL HEAT-SHOCK PROTEIN (HSP20) FAMILY ; HEAT SHOCK PROTEIN HSP16 ; HSP20 ; HSP20-like chaperones	GO:0040010_P_positive regulation of growth rate; GO:0008340_P_determination of adult life span;	K04455_04010_MAPK signaling pathway; K04455_04370_VEGF signaling pathway;	
MGC00302	13	1508	Toxin CrTX-A	Ricin B-like lectins			

MGC00303	2	761	Serine/threonine-protein phosphatase 5	PPP5 ; TPR_1 ; TPR ; SERINE/THREONINE PROTEIN PHOSPHATASE ; PROTEIN PHOSPHATASE-5 ; TPR-like ; Metallo-dependent phosphatases ; TPR_REGION	GO:0005515_F_protein binding; GO:0005829_C_cytosol; GO:0019904_F_protein domain specific binding;	K04460_04010_MAPK signaling pathway;	3.1.3.16_Phosphoproteinphosphatase.;
MGC00304	28	839	Unknown				
MGC00305	2	791	Complement C1q tumor necrosis factor-related protein 3	C1q ; C1Q-RELATED FACTOR ; COLLAGEN ALPHA CHAIN ; COMPLEMNTC1Q ; TNF-like ; C1Q		K03986_04610_Complement and coagulation cascades; K03986_05010_Alzheimers disease;	
MGC00309	1	673	Endoplasmic reticulum-Golgi intermediate compartment protein 2	SEROLOGICALLY DEFINED BREAST CANCER ANTIGEN NY-BR-84-RELATED ; PTX1 PROTEIN ; DUF1692	GO:0005737_C_cytoplasm; GO:0005515_F_protein binding; GO:0005793_C_ER-Golgi intermediate compartment; GO:0006888_P_ER to Golgi vesicle-mediated transport;		
MGC00311	3	731	Heavy metal-binding protein HIP	C1q ; GPI-ANCHORED PROTEIN P137 ; COMPLEMNTC1Q ; TNF-like ; C1Q			
MGC00317	16	595	Mytilin-D				
MGC00318	34	1389	Actin-2	ACTINS_ACT_LIKE ; ACTINS_2 ; ACTIN ; Actin-like ATPase domain ; Actin ; ACTINS_1		K05692_04510_Focal adhesion; K05692_04520_Adherens junction; K05692_04530_Tight junction; K05692_04670_Leukocyte transendothelial migration; K05692_04810_Regulation of actin cytoskeleton;	2.7.10.2_Non-specificprotein-tyrosinekinase.;
MGC00319	1	754	Putative apoptosis inhibitor ORF87	BIR ; INHIBITOR OF APOPTOSIS ; Inhibitor of apoptosis (IAP) repeat ; INHIBITOR OF APOPTOSIS PROTEIN 1 AND 2, IAP1, IAP2 ; BIR_REPEAT_2		K04725_04120_Ubiquitin mediated proteolysis; K04725_04210_Apoptosis; K04725_04510_Focal adhesion; K04725_05222_Small cell lung cancer;	
MGC00322	16	782	Complement C1q tumor necrosis factor-related protein 4	C1q ; CEREBELLIN-RELATED ; COMPLEMNTC1Q ; TNF-like ; C1Q			
MGC00329	25	725	Proteasome subunit alpha type-7	N-terminal nucleophile aminohydrolases (Ntn hydrolases) ; Proteasome ; PROTEASOME SUBUNIT ALPHA TYPE 7 ; PROTEASOME SUBUNIT ALPHA/BETA ; PROTEASOME_A		K02731_03050_Proteasome;	3.4.25.1_Proteasomeendopeptidasecomplex.;
MGC00333	4	569	Collagen alpha-2(VIII) chain	C1q ; CEREBELLIN-RELATED ; SRP54 ; COMPLEMNTC1Q ; TNF-like ; C1Q			
MGC00336	11	1005					
MGC00346	40	728	Profilin	Profilin ; Profilin (actin-binding protein) ; PROFILIN		K05759_04810_Regulation of actin cytoskeleton;	
MGC00348	1	646	Fibrinogen-like protein A	FIBRINOGEN AND FIBRONECTIN ; Fibrinogen C-terminal domain-like ; Fibrinogen_C		K06252_04510_Focal adhesion; K06252_04512_ECM-receptor interaction;	
MGC00352	12	802	Fibrinogen C domain-containing protein 1	FIBRINOGEN AND FIBRONECTIN ; Fibrinogen C-terminal domain-like ; Fibrinogen_C ; FIBRIN_AG_C_DOMAIN		K06252_04510_Focal adhesion; K06252_04512_ECM-receptor interaction;	
MGC00357	2	796	Ribonuclease inhibitor	RNI-like ; NALP (NACHT, LEUCINE RICH REPEAT AND PYRIN DOMAIN CONTAINING)-RELATED			
MGC00358	17	484	Defensin MGD-2				
MGC00365	2	845	Complement C1q tumor necrosis factor-related protein 4	C1q ; CEREBELLIN-RELATED ; COMPLEMNTC1Q ; TNF-like ; C1Q			
MGC00370	3	650	Profilin	Profilin ; Profilin (actin-binding protein) ; PROFILIN		K05759_04810_Regulation of actin cytoskeleton;	
MGC00371	6	962	Fibrinogen C domain-containing protein 1	FIBRINOGEN AND FIBRONECTIN ; Fibrinogen C-terminal domain-like ; Fibrinogen_C ; FIBRIN_AG_C_DOMAIN			

MGC00372	4	726	Ubiquitin	UBIQUITIN (RIBOSOMAL PROTEIN L40) ; UBIQUITIN_2 ; Ubiquitin-like ; ubiquitin ; UBIQUITIN_1 ; UBIQUITIN		K02977_03010_Ribosome; K08770_03320_PPAR signaling pathway; K02927_03010_Ribosome; K04551_05020_tba;	
MGC00380	1	745	Rho-related GTP-binding protein RhoQ	P-loop containing nucleoside triphosphate hydrolases ; CELL DIVISION CONTROL PROTEIN 42 ; RASTRNSFRMNG ; RAS-RELATED GTPASE ; Ras	GO:0005515_F_protein binding; GO:0005886_C_plasma membrane; GO:0030036_P_actin cytoskeleton organization and biogenesis; GO:0008360_P_regulation of cell shape; GO:0003924_F_GTPase activity;		
MGC00384	1	811	WD repeat-containing protein 53	WD40 PROTEIN ; WD_REPEATS_2 ; GPROTEINBRPT ; WD40 REPEAT PROTEIN ; WD40 repeat-like ; WD_REPEATS_REGION ; WD40		K10599_04120_Ubiquitin mediated proteolysis; K08266_04150_mTOR signaling pathway; K01852_00100_Biosynthesis of steroids; K06666_04111_Cell cycle - yeast;	
MGC00385	2	820	Thyroglobulin	HLA CLASS II GAMMA CHAIN ; Thyroglobulin_1 ; HLA CLASS II HISTOCOMPATIBILITY ANTIGEN, GAMMA CHAIN ; Thyroglobulin type-1 domain ; THYROGLOBULIN_1_2		K10809_05320_Autoimmune thyroid disease;	
MGC00392	2	667	Heavy metal-binding protein HIP	C1q ; C1Q-RELATED FACTOR ; COLLAGEN ALPHA CHAIN ; TNF-like ; coiled-coil			
MGC00394	4	844	Unknown (C-type lectin domain)	C-type lectin domain			
MGC00397	2	527	Unknown				
MGC00398	5	815	Glucan endo-1,3-beta-glucosidase A1	Concanavalin A-like lectins/glucanases ; GRAM-NEGATIVE BACTERIA BINDING PROTEIN 1 ; SECRETED GLUCOSIDASE-RELATED ; Glyco_hydro_16			3.2.1.73_Licheninase.; 3.2.1.39_Glucanendo-1,3-beta-D-glucosidase.;
MGC00400	2	827	Protein fosB	bZIP_1 ; LEUZIPPRFOS ; A DNA-binding domain in eukaryotic transcription factors ; BZIP_BASIC ; BZIP ; FOS TRANSCRIPTION FACTOR-RELATED ; coiled-coil		K04379_04010_MAPK signaling pathway; K04379_04620_Toll-like receptor signaling pathway; K04379_04660_T cell receptor signaling pathway; K04379_04662_B cell receptor signaling pathway; K04379_05210_Colorectal cancer; K04502_04310_Wnt signaling pathway;	
MGC00402	2	781	Complement C1q tumor necrosis factor-related protein 4	C1q ; COLLAGEN ALPHA CHAIN ; TNF-like ; ATPASE_DELTA ; COMPLEMENT C1Q TUMOR NECROSIS FACTOR-RELATED PROTEIN 3			
MGC00405	2	1154	14-3-3-like protein 1	1433_CAEEL_P41932; ; 14-3-3 ; 1433ZETA ; coiled-coil ; 1433_1 ; 14-3-3 protein		K06630_04110_Cell cycle;	
MGC00409	21	664	Complement C1q-like protein 4	C1q ; GLIACOLIN-RELATED ; CEREBELLIN-RELATED ; TNF-like ; C1Q			
MGC00410	13	707	D-galactoside-specific lectin	G-PROTEIN COUPLED RECEPTOR ; Gal_Lectin ; SUEL_LECTIN ; gb def: Mus musculus 13 days embryo heart cDNA, RIKEN full-length enriched library, clon			3.2.1.23_Beta-galactosidase.;
MGC00411	21	684	40S ribosomal protein S19	Ribosomal_S19e ; RIBOSOMAL_S19E ; 'Winged helix' DNA-binding domain ; 40S RIBOSOMAL PROTEIN S19		K02966_03010_Ribosome;	
MGC00413	3	409	Unknown				
MGC00417	6	1172	High mobility group protein B2	HMG_box ; HMG-box ; SWI/SNF-RELATED CHROMATIN BINDING PROTEIN ; HMG_BOX_2 ; HIGHMOBLTY12		K10802_03410_Base excision repair;	
MGC00418	6	444	SCO-spondin	LDL receptor-like module ; LDLRA_2			

MGC00420	1	715	Unknown				
MGC00421	5	662	Myticin-A				
MGC00422	60	685	Myticin-B				
MGC00423	15	556	Mytilin-B				
MGC00425	9	835	Major egg antigen	ACRYSTALLIN ; HEAT SHOCK PROTEIN 30 ; SMALL HEAT-SHOCK PROTEIN (HSP20) FAMILY ; HSP20 ; HSP20-like chaperones	GO:0005515_F_protein binding;	K04455_04010_MAPK signaling pathway; K04455_04370_VEGF signaling pathway;	
MGC00431	5	475	Defensin MGD-1 (Fragment)	Defensin_2 ; Scorpion toxin-like ; GLYCO_HORMONE_BETA_1 ; ARTHROPOD_DEFENSINS			
MGC00441	17	846					
MGC00443	3	330	40S ribosomal protein S21	40S RIBOSOMAL PROTEIN S21E ; Ribosomal_S21e	GO:0043022_F_ribosome binding;	K02971_03010_Ribosome;	
MGC00444	2	705	Complement C1q tumor necrosis factor-related protein 3	C1q ; C1Q-RELATED FACTOR ; COLLAGEN ALPHA CHAIN ; COMPLEMNTC1Q ; TNF-like ; C1Q		K03987_04610_Complement and coagulation cascades; K03987_05010_Alzheimers disease;	
MGC00447	2	562	C-type lectin domain family 4 member D	REGENERATING GENE TYPE IV-RELATED ; C-type lectin-like ; C_TYPE_LECTIN_1 ; LITHOSTATHINE ; Lectin_C ; ANTIFREEZEII ; C_TYPE_LECTIN_2		K06468_04640_Hematopoietic cell lineage;	
MGC00449	1	505	Kelch-like protein 24	POZ domain ; MGMT ; KELCH-RELATED PROTEIN ; BTB			
MGC00451	2	456	Defensin MGD-1 (Fragment)	Defensin_2 ; Scorpion toxin-like ; ARTHROPOD_DEFENSINS			
MGC00452	9	855	Calreticulin	CALRETICULIN_1 ; Concanavalin A-like lectins/glucanases ; CALRETICULIN_REPEAT ; CALRETICULIN AND CALNEXIN ; CALRETICULIN ; Calreticulin ; CALRETICULIN_2		K08057_04612_Antigen processing and presentation;	
MGC00453	11	977	Actin, adductor muscle	ACTINS_ACT_LIKE ; ACTIN ; Actin-like ATPase domain ; Actin ; ACTINS_1		K05692_04510_Focal adhesion; K05692_04520_Adherens junction; K05692_04530_Tight junction; K05692_04670_Leukocyte transendothelial migration; K05692_04810_Regulation of actin cytoskeleton;	2.7.10.2_Non-specificprotein-tyrosinekinase.;
MGC00454	6	564	Complement C1q-like protein 3	C1q ; C1Q-RELATED FACTOR ; COLLAGEN ALPHA CHAIN ; COMPLEMNTC1Q ; TNF-like ; C1Q	GO:0005515_F_protein binding;		
MGC00455	5	813	Unknown				
MGC00463	2	723	Thyroglobulin	INSULIN-LIKE GROWTH FACTOR BINDING PROTEIN ; Thyroglobulin_1 ; Thyroglobulin type-1 domain ; INSULIN-LIKE GROWTH FACTOR BINDING PROTEIN 3 ; THYROGLOBULIN_1_2		K10809_05320_Autoimmune thyroid disease;	
MGC00464	12	1975	Heat shock protein HSP 90-alpha	HSP90 C-terminal domain (C-terminal part of Pfam 00183) ; HEAT SHOCK PROTEIN 90 ; Ribosomal protein S5 domain 2-like ; HSP90 ; ATPase domain of HSP90 chaperone/DNA topoisomerase II/histidine kinase ; coiled-coil		K04079_04612_Antigen processing and presentation; K04079_04914_Progesterone-mediated oocyte maturation; K04079_05215_Prostate cancer;	
MGC00470	11	858	Putative ATP synthase subunit f, mitochondrial	ATP SYNTHASE F CHAIN, MITOCHONDRIAL-RELATED		K02130_00190_Oxidative phosphorylation;	
MGC00475	10	906		C1q ; TNF-like			
MGC00476	34	1080	Nuclease-sensitive element-binding protein 1	COLD SHOCK DOMAIN CONTAINING PROTEINS ; COLD_SHOCK ; Nucleic acid-binding proteins ; COLDSHOCK ; Q8AXS2_ORYLA_Q8AXS2; ; Y BOX BINDING PROTEIN ; CSD		K06099_04530_Tight junction;	

MGC00477	3	1659	Interleukin-1 receptor-associated kinase 4	Protein kinase-like (PK-like) ; INTERLEUKIN-1 RECEPTOR-ASSOCIATED KINASE-4 ; PROTEIN_KINASE_ST ; PROTEIN_KINASE_ATP ; Q69FE1_HUMAN_Q69FE1; ; SERINE-THREONINE PROTEIN KINASE, PLANT-TYPE ; Pkinase ; DEATH domain ; PROTEIN_KINASE_DOM	GO:0005515_F_protein binding;	K00924_00562_Inositol phosphate metabolism; K00924_00632_Benzoate degradation via CoA ligation; K04733_04210_Apoptosis; K04733_04620_Toll-like receptor signaling pathway;	2.7.11.1_Non-specificserine/threonineproteinkinase.;
MGC00479	4	864	Calcyphosin-like protein	EF_HAND_1 ; CALMODULIN ; EF_HAND_2 ; CALL_CAEEL_P04630; ; EF-hand ; CALCYPHOSINE/TPP ; efhand			2.7.11.1_Non-specificserine/threonineproteinkinase.;
MGC00488	1	802	WD repeat-containing protein 89	WD_REPEATS_2 ; Q7Q405_EEEEE_Q7Q405; ; WD40 repeat-like ; WD_REPEATS_REGION ; WD40	GO:0005515_F_protein binding; GO:0005634_C_nucleus;	K06666_04111_Cell cycle - yeast;	2.7.11.1_Non-specificserine/threonineproteinkinase.; 2.3.1.48_Histoneacetyltransferase.;
MGC00500	13	1019	Calmodulin	EF_HAND_1 ; CALMODULIN ; EF_HAND_2 ; Q76LB7_STRIE_Q76LB7; ; EF-hand ; CALCIUM BINDING PROTEIN ; efhand			2.7.11.1_Non-specificserine/threonineproteinkinase.;
MGC00501	6	693	Integumentary mucin C.1 (Fragment)	INTESTINAL TREFOIL FACTOR-RELATED ; Trefoil ; PTREFOIL ; TREFOIL FACTOR 1			3.2.1.20_Alpha-glucosidase.;
MGC00505	1	612	Serine/threonine-protein kinase pim-3	Protein kinase-like (PK-like) ; PROTEIN_KINASE_ST ; PROTEIN_KINASE_ATP ; Pkinase ; PIM3_COTJA_Q9PU85; ; SERINE/THREONINE PROTEIN KINASE ; PROTEIN_KINASE_DOM		K08806_05221_Acute myeloid leukemia; K04702_04630_Jak-STAT signaling pathway; K04702_05221_Acute myeloid leukemia;	2.7.11.1_Non-specificserine/threonineproteinkinase.;
MGC00508	3	636	Complement C1q-like protein 3	C1q ; C1Q-RELATED FACTOR ; COLLAGEN ALPHA CHAIN ; TNF-like ; coiled-coil ; C1Q			
MGC00509	6	1183	Rho-related GTP-binding protein RhoQ	P-loop containing nucleoside triphosphate hydrolases ; CELL DIVISION CONTROL PROTEIN 42 ; RASTRNSFRMNG ; RAS-RELATED GTPASE ; small_GTP: small GTP-binding protein domain ; Ras ; SIGMA54_INTERACT_1			
MGC00510	7	725	Ficolin-1	FIBRINOGEN AND FIBRONECTIN ; Fibrinogen C-terminal domain-like ; Fibrinogen_C ; FIBRIN_AG_C_DOMAIN	GO:0007411_P_axon guidance; GO:0007155_P_cell adhesion;	K06252_04510_Focal adhesion; K06252_04512_ECM-receptor interaction;	
MGC00516	5	985	Galectin-9	Concanavalin A-like lectins/glucanases ; GALECTIN ; Gal-bind_lectin			3.1.1.5_Lysophospholipase.;
MGC00517	28	754	Heavy metal-binding protein HIP	C1q ; GPI-ANCHORED PROTEIN P137 ; COMPLEMNTC1Q ; TNF-like ; C1Q	GO:0005737_C_cytoplasm; GO:0005739_C_mitochondrion; GO:0030308_P_negative regulation of cell growth;		
MGC00519	1	683	Thyroglobulin	INSULIN-LIKE GROWTH FACTOR BINDING PROTEIN ; Thyroglobulin_1 ; Thyroglobulin type-1 domain ; INSULIN-LIKE GROWTH FACTOR BINDING PROTEIN 3 ; THYROGLOBULIN_1_2	GO:0005576_C_extracellular region; GO:0007165_P_signal transduction;	K10809_05320_Autoimmune thyroid disease;	
MGC00522	9	785	Complement C1q tumor necrosis factor-related protein 3	C1q ; GLIACOLIN-RELATED ; CEREBELLIN-RELATED ; COMPLEMNTC1Q ; TNF-like ; C1Q	GO:0005576_C_extracellular region;		
MGC00529	3	667	Skin secretory protein xP2	INTESTINAL TREFOIL FACTOR-RELATED ; Trefoil ; PTREFOIL ; TREFOIL FACTOR 1			
MGC00532	2	560	Thyroglobulin	INSULIN-LIKE GROWTH FACTOR BINDING PROTEIN ; Thyroglobulin_1 ; Thyroglobulin type-1 domain ; INSULIN-LIKE GROWTH FACTOR BINDING PROTEIN 3 ; THYROGLOBULIN_1_2		K10809_05320_Autoimmune thyroid disease;	

MGC00536	4	656	Macrophage mannose receptor 1	C-type lectin-like ; Lectin_C ; C_TYPE_LECTIN_2	GO:0005887_C_integral to plasma membrane; GO:0005886_C_plasma membrane; GO:0004872_F_receptor activity; GO:0005537_F_mannose binding; GO:0006898_P_receptor-mediated endocytosis;		
MGC00538	1	741		Profilin (actin-binding protein)			
MGC00542	2	454					
MGC00545	5	1070	Angiopoietin-related protein 6	FIBRINOGEN AND FIBRONECTIN ; Fibrinogen C-terminal domain-like ; Fibrinogen_C ; FIBRIN_AG_C_DOMAIN	GO:0030141_C_secretory granule;	K03903_04610_Complement and coagulation cascades; K06252_04510_Focal adhesion; K06252_04512_ECM-receptor interaction;	
MGC00551	1	335	Unknown				
MGC00554	3	739	Fucolectin-2	PAN_1 ; Galactose-binding domain-like ; F5_F8_type_C			
MGC00556	15	593	40S ribosomal protein S27a	Ribosomal_S27 ; UBIQUITIN_2 ; Ubiquitin-like ; ubiquitin ; UBIQUITIN_1 ; UBIQUITIN		K02977_03010_Ribosome; K08770_03320_PPAR signaling pathway; K02927_03010_Ribosome; K04551_05020_tba;	
MGC00564	28	1186	ADP,ATP carrier protein 1, mitochondrial	ADP,ATP CARRIER PROTEIN ; SOLCAR ; ADPTRNSLCASE ; MITOCARRIER ; MITOCHONDRIAL CARRIER PROTEIN RELATED ; Mitochondrial carrier ; Mito_carr		K05863_04020_Calcium signaling pathway;	
MGC00573	22	711	Lysozyme	DESTABILASE-RELATED ; Destabilase ; Lysozyme-like	GO:0003796_F_lysozyme activity; GO:0050829_P_defense response to Gram-negative bacterium; GO:0005576_C_extracellular region; GO:0050830_P_defense response to Gram-positive bacterium;		3.2.1.17_Lysozyme.;
MGC00580	2	679	Transmembrane protein 205	Transport; Transmembrane			
MGC00596	6	662	Complement C1q tumor necrosis factor-related protein 3	C1q ; GLIACOLIN-RELATED ; CEREBELLIN-RELATED ; TNF-like			
MGC00599	1	651	TNF receptor-associated factor 6	RING/U-box ; ZF_RING_1 ; zf-C3HC4 ; ZF_RING_2 ; TNF RECEPTOR-ASSOCIATED FACTOR 6 ; TNF RECEPTOR ASSOCIATED FACTOR			
MGC00603	1	577	ARF GTPase-activating protein GIT1	CENTAURIN/ARF ; ArfGap ; Pyk2-associated protein beta ARF-GAP domain ; ARFGAP ; REVINTRACTNG ; CENTAURIN/ARF-RELATED	GO:0008277_P_regulation of G-protein coupled receptor protein signaling pathway; GO:0005515_F_protein binding;	K05737_04810_Regulation of actin cytoskeleton; K05737_05120_Epithelial cell signaling in Helicobacter pylori infection;	
MGC00609	5	669	Low affinity immunoglobulin epsilon Fc receptor	C-type lectin-like ; C-TYPE LECTIN SUPERFAMILY MEMBER ; Lectin_C ; LOW AFFINITY IMMUNOGLOBULIN EPSILON FC RECEPTOR (CD23 ANTIGEN) ; ANTIFREEZEII ; C_TYPE_LECTIN_2	GO:0009897_C_external side of plasma membrane; GO:0002925_P_positive regulation of humoral immune response mediated by circulating immunoglobulin; GO:0007391_P_dorsal closure; GO:0005515_F_protein binding;	K06468_04640_Hematopoietic cell lineage;	
MGC00610	2	690	Ras-related protein Ral-A	P-loop containing nucleoside triphosphate hydrolases ; RASTRNSFRMNG ; RAS-RELATED GTPASE ; RAS-RELATED PROTEIN RAL ; small_GTP: small GTP-binding protein domain ; Ras	GO:0046329_P_negative regulation of JNK cascade; GO:0006935_P_chemotaxis; GO:0007165_P_signal transduction; GO:0005525_F_GTP binding;	K07835_05212_Pancreatic cancer; K07834_05212_Pancreatic cancer;	

MGC00616	3	956	Calreticulin	P-domain of calnexin/calreticulin ; Concanavalin A-like lectins/glucanases ; CALRETICULIN AND CALNEXIN ; CALRETICULIN ; ER_TARGET ; Calreticulin ; coiled-coil		K08057_04612_Antigen processing and presentation;	
MGC00617	1	543	Unknown (TNF-like)	TNF-like			
MGC00618	16	840	Proteasome subunit beta type-1	N-terminal nucleophile aminohydrolases (Ntn hydrolases) ; Proteasome ; PROTEASOME_B ; PROTEASOME SUBUNIT BETA TYPE 1 ; PROTEASOME SUBUNIT ALPHA/BETA		K02732_03050_Proteasome;	3.4.25.1_Proteasomeendopeptidasecomplex.;
MGC00623	2	340	Unknown				
MGC00625	5	688	Vesicle-associated membrane protein 4	SNARE PROTEINS ; VAMP-4 ; V_SNARE ; SYNAPTOBREVN ; coiled-coil ; Synaptobrevin	GO:0000139_C_Golgi membrane; GO:0005764_C_lysosome; GO:0005768_C_endosome;	K08513_04130_SNARE interactions in vesicular transport; K08510_04130_SNARE interactions in vesicular transport;	
MGC00630	3	722	Astacin	ZINC METALLOPROTEINASE NAS-RELATED ; DISCOIDIN, CUB, EGF, LAMININ , AND ZINC METALLOPROTEASE DOMAIN ; ZINC_PROTEASE ; Metalloproteases ('zincins'), catalytic domain ; Astacin ; ASTACIN			3.4.24.21_Astacin.;
MGC00636	1	683	Unknown	MACPF ; MAC_PERFORIN			
MGC00639	2	466	Defensin MGD-2	Scorpion toxin-like			
MGC00651	1	734	Probable nucleoporin Nup54	NUCLEOPORIN-LIKE PROTEIN 1 (NUPL2) ; NUCLEOPORIN-RELATED			
MGC00652	1	681	Histone-lysine N-methyltransferase MLL3	FYVE/PHD zinc finger ; ZF_DAG_PE_2 ; MYST-RELATED PROTEIN ; MYST-RELATED PROTEINS	GO:0006911_P_phagocytosis, engulfment; GO:0005515_F_protein binding;		2.1.1.43_Histone-lysineN-methyltransferase.;
MGC00659	12	483	Mytilin-B				
MGC00665	2	499	Ubiquitin	UBIQUITIN (RIBOSOMAL PROTEIN L40) ; UBIQUITIN_2 ; Ubiquitin-like ; ubiquitin ; UBIQUITIN_1 ; UBIQUITIN		K02977_03010_Ribosome; K08770_03320_PPAR signaling pathway; K02927_03010_Ribosome; K04551_05020_tba;	
MGC00667	1	623	Unknown (Cystine-knot cytokines)	Cystine-knot cytokines			
MGC00670	29	810	Heat shock protein HSP 90-alpha	HEAT SHOCK PROTEIN 90 ; HEATSHOCK90 ; HATPase_c ; HSP90 ; ATPase domain of HSP90 chaperone/DNA topoisomerase II/histidine kinase		K04079_04612_Antigen processing and presentation; K04079_04914_Progesterone-mediated oocyte maturation; K04079_05215_Prostate cancer;	
MGC00682	4	703	Allograft inflammatory factor 1-like	EF_HAND_1 ; EF_HAND_2 ; BRT1_RAT_P55007; ; ALLOGRAFT INFLAMMATORY FACTOR-1 ; EF-hand ; efhand			2.7.11.1_Non-specificserine/threonineproteinkinase.; 1.1.99.5_Glycerol-3-phosphatedehydrogenase.;
MGC00684	1	481	78 kDa glucose-regulated protein	HEAT SHOCK PROTEIN 70KDA ; HEAT SHOCK PROTEIN 70 (HSP70) ; HSP70 ; Actin-like ATPase domain ; coiled-coil	GO:0005783_C_endoplasmic reticulum; GO:0005515_F_protein binding; GO:0043022_F_ribosome binding; GO:0006983_P_ER overload response;	K09490_05060_tba;	
MGC00685	4	782	Cell division control protein 42 homolog	P-loop containing nucleoside triphosphate hydrolases ; CELL DIVISION CONTROL PROTEIN 42 ; RASTRNSFRMNG ; RAS-RELATED GTPASE ; small_GTP: small GTP-binding protein domain ; Ras	GO:0005515_F_protein binding;		

MGC00694	3	707	Mucin-2 (Fragments)	CTCK_1 ; GLYCO_HORMONE_BETA_1 ; CTCK_2	GO:0005576_C_extracellular region; GO:0042381_P_hemolymph coagulation; GO:0007599_P_hemostasis; GO:0035006_P_melanization defense response; GO:0042060_P_wound healing; GO:0042803_F_protein homodimerization activity;		
MGC00702	2	648	Ubiquitin	UBIQUITIN (RIBOSOMAL PROTEIN L40) ; UBIQUITIN_2 ; Ubiquitin-like ; ubiquitin ; UBIQUITIN_1 ; UBIQUITIN		K02977_03010_Ribosome; K08770_03320_PPAR signaling pathway; K02927_03010_Ribosome; K04551_05020_tba;	
MGC00709	3	568	Double-strand-break repair protein rad21 homolog	SCC1 / RAD21 FAMILY MEMBER ; Rad21_Rec8 ; COHESIN REC8	GO:0006302_P_double-strand break repair; GO:0007131_P_meiotic recombination; GO:0005515_F_protein binding; GO:0006310_P_DNA recombination;	K06670_04111_Cell cycle - yeast;	
MGC00729	1	632	Matrix metalloproteinase-28	PGBD-like ; PG_binding_1 ; MATRIXIN ; Metalloproteases ('zincins'), catalytic domain ; MATRIX METALLOPROTEINASE ; Peptidase_M10			3.4.24.12_Envelysin.; 3.4.24.34_Neutrophilcollagenase.; 3.4.24.23_Matrilysin.; 3.4.24.65_Macrophageelastase.;
MGC00738	1	722	Ras-related protein Rab-5C	P-loop containing nucleoside triphosphate hydrolases ; RASTRNSFRMNG ; RAS-RELATED GTPASE ; RAS-RELATED PROTEIN RAB-5 ; Ras ; SIGMA54_INTERACT_1			2.7.11.1_Non-specificserine/threonineproteinkinase.;
MGC00742	1	770	Putative mediator of RNA polymerase II transcription subunit 26				
MGC00744	5	594		C-type lectin-like			
MGC00747	1	715	Fibrinogen C domain-containing protein 1	FIBRINOGEN AND FIBRONECTIN ; Fibrinogen C-terminal domain-like ; Fibrinogen_C ; FIBRIN_AG_C_DOMAIN			
MGC00748	6	646	Brevican core protein	C-type lectin-like ; ASIALOGLYCOPROTEIN RECEPTOR ; C-TYPE LECTIN SUPERFAMILY MEMBER ; Lectin_C ; C_TYPE_LECTIN_2			
MGC00757	14	939		Scorpion toxin-like			
MGC00761	11	846	12 kDa FK506-binding protein	FKBP-like ; FK506 BINDING PROTEIN ; FK506-BINDING PROTEIN 1 ; FKBP_PPIASE ; FKBP_C			5.2.1.8_Peptidylprolylisomerase.;
MGC00773	2	437	Defensin MGD-2				
MGC00774	38	903	A-agglutinin anchorage subunit				
MGC00775	1	312		Antiviral defense, helicase, DEATH domain			
MGC00779	8	712	WSC domain-containing protein 2	WSC		K00771_00532_Chondroitin sulfate biosynthesis; K00771_01030_tba;	2.4.2.26_Proteinxylosyltransferase.;
MGC00786	10	667		C-type lectin-like			
MGC00789	1	603	Complement C1q tumor necrosis factor-related protein 3	C1q ; CEREBELLIN-RELATED ; COMPLEMNTC1Q ; TNF-like ; C1Q	GO:0005737_C_cytoplasm; GO:0005739_C_mitochondrion; GO:0030308_P_negative regulation of cell growth;	K03986_04610_Complement and coagulation cascades; K03986_05010_Alzheimers disease; K03987_04610_Complement and coagulation cascades; K03987_05010_Alzheimers disease;	
MGC00791	13	752	WSC domain-containing protein 2	WSC		K00771_00532_Chondroitin sulfate biosynthesis; K00771_01030_tba;	2.4.2.26_Proteinxylosyltransferase.;
MGC00800	2	717	Caprin-2	C1q ; TNF-like			
MGC00813	6	541					
MGC00840	1	686	Myticin-B				
MGC00845	4	793					

MGC00848	10	970	Proteasome subunit beta type-5	N-terminal nucleophile aminohydrolases (Ntn hydrolases) ; Proteasome ; PROTEASOME_B ; PROTEASOME ; PROTEASOME SUBUNIT BETA TYPE 5,8 ; PROTEASOME SUBUNIT ALPHA/BETA		K02737_03050_Proteasome; K02740_03050_Proteasome;	3.4.25.1_Proteasomeendopeptidasecomplex.;
MGC00857	3	1435	Suppressor of cytokine signaling 2	SUPPRESSOR OF CYTOKINE SIGNALING ; Q861R0_BOVIN_Q861R0; ; SOCS ; SH2DOMAIN ; SUPPRESSOR OF CYTOKINE SIGNALING-2 ; SOCS_box ; SH2 domain ; SH2		K04701_04630_Jak-STAT signaling pathway; K04695_04630_Jak-STAT signaling pathway; K04695_04910_Insulin signaling pathway; K04695_04930_Type II diabetes mellitus;	
MGC00860	2	407	Integumentary mucin C.1 (Fragment)	INTESTINAL TREFOIL FACTOR-RELATED ; Trefoil ; PTREFOIL ; TREFOIL FACTOR 1		K01187_00052_Galactose metabolism; K01187_00500_Starch and sucrose metabolism;	3.2.1.20_Alpha-glucosidase.;
MGC00864	6	858	Heavy metal-binding protein HIP	C1q ; CEREBELLIN-RELATED ; COMPLEMNTC1Q ; TNF-like ; coiled-coil ; C1Q	GO:0005576_C_extracellular region;		
MGC00865	6	1189	Galectin-4	Concanavalin A-like lectins/glucanases ; GALECTIN ; Gal-bind_lectin			3.1.1.5_Lysophospholipase.;
MGC00868	3	798		C1q ; TNF-like			
MGC00884	5	582	WSC domain-containing protein 2	WSC ; Hairpin loop containing domain-like		K00771_00532_Chondroitin sulfate biosynthesis; K00771_01030_tba;	2.4.2.26_Proteinxylosyltransferase.;
MGC00889	4	758	Caprin-2	C1q ; CEREBELLIN-RELATED ; COMPLEMNTC1Q ; TNF-like ; C1Q			
MGC00903	1	684	Beta-1,4-galactosyltransferase 2	Nucleotide-diphospho-sugar transferases ; Galactosyl_T_2 ; BETA-1,4-GALACTOSYLTRANSFERASE		K07966_00602_tba; K07966_01030_tba; K07966_01031_tba; K07967_00602_tba; K07967_01031_tba; K07969_00533_Keratan sulfate biosynthesis; K07969_00602_tba; K07969_01030_tba; K07969_01031_tba;	2.4.1.38_Beta-N-acetylglucosaminylglycopeptidebeta-1,4-galactosyltransferase.; 2.4.1.22_Lactosesynthase.; 2.4.1.90_N-acetyllactosaminesynthase.;
MGC00907	2	1212	Superoxide dismutase [Cu-Zn]	CU/ZN SUPEROXIDE DISMUTASE ; Sod_Cu ; CUZNDISMTASE ; SUPEROXIDE DISMUTASE [CU-ZN] ; SOD_CU_ZN_1 ; Q70GL0_MYTED_Q70GL0; ; Cu,Zn superoxide dismutase-like ; SOD_CU_ZN_2		K04565_05030_tba;	1.15.1.1_Superoxidedismutase.;
MGC00911	3	830	Uncharacterized serine-rich protein C215.13				
MGC00912	1	685	Somatostatin-like receptor F_48D10.1	G_PROTEIN_RECEP_F1_2 ; G-PROTEIN COUPLED RECEPTOR ; GPCRRHODOPSN ; 7tm_1 ; Family A G protein-coupled receptor-like		K04239_04080_Neuroactive ligand-receptor interaction;	
MGC00921	1	736	Hippocalcin-like protein 1	EF_HAND_1 ; RECOVERIN ; EF_HAND_2 ; NCAH_DROME_P42325; ; EF-hand ; CALCIUM BINDING PROTEINS ; efhand	GO:0005515_F_protein binding;	K08328_04740_Olfactory transduction;	1.6.3.1_NAD(P)Hoxidase.;
MGC00924	1	764	Delta and Notch-like epidermal growth factor-related receptor	EGF/Laminin ; EGF-LIKE DOMAIN PROTEIN ; EGF ; EGF_3 ; CRUMBS(D.MELANOGASTER) RELATED ; EGF_1 ; EGF_2		K02599_04320_Dorso-ventral axis formation; K02599_04330_Notch signaling pathway; K06052_04330_Notch signaling pathway;	
MGC00926	3	509	Mytilin-C				
MGC00934	8	887	Fibrinogen C domain-containing protein 1	FIBRINOGEN AND FIBRONECTIN ; Fibrinogen C-terminal domain-like ; Fibrinogen_C ; FIBRIN_AG_C_DOMAIN		K06252_04510_Focal adhesion; K06252_04512_ECM-receptor interaction;	
MGC00991	2	737	Allograft inflammatory factor 1-like	EF_HAND_1 ; EF_HAND_2 ; BRT1_RAT_P55007; ; ALLOGRAFT INFLAMMATORY FACTOR-1 ; EF-hand ; efhand	GO:0015629_C_actin cytoskeleton; GO:0005925_C_focal adhesion;		2.7.11.1_Non-specificserine/threonineproteinkinase.; 1.1.99.5_Glycerol-3-phosphatedehydrogenase.;
MGC01021	2	696	Myticin-C				

MGC01028	11	721	Collagen alpha-1(VIII) chain	C1q ; COLLAGEN ALPHA 1(VIII) CHAIN ; COLLAGEN ALPHA CHAIN ; COMPLEMNTC1Q ; TNF-like ; C1Q			
MGC01031	2	1047		C-type lectin-like			
MGC01042	3	1035	Allene oxide synthase-lipoxygenase protein	IG_MHC ; Lipoxygenase ; LIPOXYGENASE ; coiled-coil ; Lipoxigenase		K08021_00590_Arachidonic acid metabolism; K00461_00590_Arachidonic acid metabolism; K00461_00591_Linoleic acid metabolism; K00458_00590_Arachidonic acid metabolism;	1.13.11.31_Arachidonate12-lipoxygenase.; 4.2.1.92_Hydroperoxidedehydratase.; 1.13.11.40_Arachidonate8-lipoxygenase.; 1.13.11.34_Arachidonate5-lipoxygenase.;
MGC01070	1	703	Myticin-A				
MGC01080	1	688					
MGC01089	6	755	Complement C1q tumor necrosis factor-related protein 3	C1q ; GLIACOLIN-RELATED ; CEREBELLIN-RELATED ; COMPLEMNTC1Q ; TNF-like ; C1Q			
MGC01107	12	1328	Transcription factor AP-1	bZIP_1 ; Jun ; JUN TRANSCRIPTION FACTOR-RELATED ; A DNA-binding domain in eukaryotic transcription factors ; BZIP_BASIC ; LEUZIPPRJUN ; BZIP ; coiled-coil ; TRANSCRIPTION FACTOR C-JUN	GO:0005515_F_protein binding; GO:0046686_P_response to cadmium ion; GO:0051597_P_response to methylmercury;		
MGC01140	1	487	Myticin-A				
MGC01145	5	678	ATP synthase-coupling factor 6, mitochondrial	Mitochondrial ATP synthase coupling factor 6 (Pfam 05511) ; ATP SYNTHASE COUPLING FACTOR 6, MITOCHONDRIAL ; SUBATP-synt_F6		K02131_00190_Oxidative phosphorylation;	
MGC01166	1	801	Thyroglobulin	HLA CLASS II GAMMA CHAIN ; Thyroglobulin_1 ; HLA CLASS II HISTOCOMPATIBILITY ANTIGEN, GAMMA CHAIN ; Thyroglobulin type-1 domain ; THYROGLOBULIN_1_2		K10809_05320_Autoimmune thyroid disease;	
MGC01186	2	1396	Cyclic AMP-dependent transcription factor ATF-4	bZIP_1 ; ACTIVATING TRANSCRIPTION FACTOR (ATF) 4/5 ; BZIP_BASIC ; BZIP ; coiled-coil		K04374_04010_MAPK signaling pathway; K04374_04720_Long-term potentiation; K04374_04912_GnRH signaling pathway; K04374_05215_Prostate cancer;	
MGC01206	1	769	ATP synthase subunits region ORF 7				
MGC01219	5	415	Ubiquitin	UBIQUITIN (RIBOSOMAL PROTEIN L40) ; UBIQUITIN_2 ; Ubiquitin-like ; ubiquitin ; Ribosomal_L40e ; UBIQUITIN_1 ; UBIQUITIN		K02977_03010_Ribosome; K08770_03320_PPAR signaling pathway; K02927_03010_Ribosome; K04551_05020_tba;	
MGC01285	4	575	Perlucin	C-type lectin-like ; C_TYPE_LECTIN_1 ; C-TYPE LECTIN SUPERFAMILY MEMBER ; Lectin_C ; GALACTOSE-SPECIFIC C-TYPE LECTIN ; C_TYPE_LECTIN_2			
MGC01300	11	593	Perlucin	C-type lectin-like ; C_TYPE_LECTIN_1 ; C-TYPE LECTIN SUPERFAMILY MEMBER ; Lectin_C ; CD209 ANTIGEN (DENDRITIC CELL-SPECIFIC ICAM-3-GRABBING NONINTEGRIN 1) (DC-SIGN1) ; C_TYPE_LECTIN_2			
MGC01312	2	701	Fucolectin-5	Galactose-binding domain-like ; F5_F8_type_C			
MGC01316	3	368	C-type lectin domain family 4 member M	C-type lectin-like			
MGC01319	19	1445	Actin, adductor muscle	ACTINS_ACT_LIKE ; ACTINS_2 ; ACTIN ; Actin-like ATPase domain ; Actin ; ACTINS_1		K05692_04510_Focal adhesion; K05692_04520_Adherens junction; K05692_04530_Tight junction; K05692_04670_Leukocyte transendothelial migration; K05692_04810_Regulation of actin cytoskeleton;	2.7.10.2_Non-specificprotein-tyrosinekinase.;

MGC01326	4	764	CD209 antigen	C-type lectin-like ; C-TYPE LECTIN SUPERFAMILY MEMBER ; Lectin_C ; GALACTOSE-SPECIFIC C-TYPE LECTIN ; C_TYPE_LECTIN_2			
MGC01337	7	956	Heavy metal-binding protein HIP	C1q ; CEREBELLIN-RELATED ; COMPLEMNTC1Q ; TNF-like ; C1Q	GO:0030023_F_extracellular matrix constituent conferring elasticity; GO:0005515_F_protein binding; GO:0005576_C_extracellular region;		
MGC01354	9	699	Small nuclear ribonucleoprotein Sm D3	SMALL NUCLEAR RIBONUCLEOPROTEIN SM ; Sm-like ribonucleoproteins ; LSM ; SMALL NUCLEAR RIBONUCLEOPROTEIN SM D3			
MGC01371	4	539		EF_HAND_1 ; EF_HAND_2 ; EF-hand ; efhand			
MGC01376	1	509	Hepatic lectin	C-type lectin-like ; C_TYPE_LECTIN_1 ; Lectin_C ; C-TYPE LECTIN PROTEINS ; C_TYPE_LECTIN_2	GO:0004888_F_transmembrane receptor activity; GO:0005537_F_mannose binding; GO:0009986_C_cell surface;		
MGC01377	1	495	Tetratricopeptide repeat protein 25	TPR_1 ; TPR ; TPR-like ; TPR_REGION		K04460_04010_MAPK signaling pathway;	3.1.3.16_Phosphoproteinphosphatase.;
MGC01383	12	820	Mammalian ependymin-related protein 1				
MGC01385	10	1576	SCO-spondin	Serine proterase inhibitors ; TSP_1 ; VWFC_2 ; ADAMTS-RELATED PROTEASE (INVERTEBRATE) ; ADAMTS (A DISINTEGRIN AND METALLOPROTEASE WITH THROMBOSPONDIN MOTIFS) PROTEASE M12B-RELATED ; TSP-1 type 1 repeat ; EGF_2 ; TSP1REPEAT ; VWFC_1 ; TSP1 ; CTCK_2	GO:0005737_C_cytoplasm;	K06841_04360_Axon guidance;	3.4.24.14_ProcollagenN-endopeptidase.;
MGC01386	3	628	Complement C1q tumor necrosis factor-related protein 6	C1q ; COLLAGEN ALPHA CHAIN ; COMPLEMENT C1Q AND TUMOR NECROSIS FACTOR RELATED PROTEIN 6 ; COMPLEMNTC1Q ; TNF-like ; C1Q			
MGC01389	11	827					
MGC01402	5	932		AA_TRANSFER_CLASS_2			
MGC01403	1	446	Tolloid-like protein 2	CUB ; TOLLOID-RELATED ; DISCOIDIN, CUB, EGF, LAMININ , AND ZINC METALLOPROTEASE DOMAIN ; Spermadhesin, CUB domain			3.4.24.19_ProcollagenC-endopeptidase.; 3.4.24.21_Astacin.;
MGC01404	3	1352	Uncharacterized protein yfeX	Dyp_perox_fam: Dyp-type peroxidase fami ; Dyp_perox			
MGC01406	2	663	Lithostathine-1	C-type lectin-like ; C_TYPE_LECTIN_1 ; LITHOSTATHINE ; Lectin_C ; C_TYPE_LECTIN_2			
MGC01413	1	410	Testis-specific serine/threonine-protein kinase 4	Protein kinase-like (PK-like) ; PROTEIN_KINASE_ST ; Pkinase ; TESTIS-SPECIFIC SERINE/THREONINE KINASE 22C ; CALCIUM/CALMODULIN-DEPENDENT PROTEIN KINASE-RELATED ; Q6SA08_HUMAN_Q6SA08; ; PROTEIN_KINASE_DOM			2.7.11.1_Non-specificserine/threonineproteinkinase.;
MGC01414	2	632	Macrophage mannose receptor 1	C-type lectin-like ; C_TYPE_LECTIN_1 ; LITHOSTATHINE ; Lectin_C ; C_TYPE_LECTIN_2			
MGC01432	1	379	Angiotensin-converting enzyme (Fragment)	Peptidase_M2 ; ANGIOTENSIN-CONVERTING ENZYME ; ANGIOTENSIN-CONVERTING ENZYME (DIPEPTIDYL CARBOXYPEPTIDASE) ; Metalloproteases ('zincins'), catalytic domain ; PEPDIPTASEA	GO:0016021_C_integral to membrane; GO:0006508_P_proteolysis; GO:0004246_F_peptidyl-dipeptidase A activity;	K01283_04614_Renin-angiotensin system;	3.4.15.1_Peptidyl-dipeptidaseA.;
MGC01446	3	962					

MGC01449	1	536	Ras-related protein Rab-6A	P-loop containing nucleoside triphosphate hydrolases ; RASTRNSFRMNG ; RAS-RELATED GTPASE ; small_GTP: small GTP-binding protein domain ; RAS-RELATED PROTEIN RAB-6 ; Ras			2.7.11.1_Non-specificserine/threonineprotein kinase.;
MGC01450	1	153	Heat shock protein HSP 90-alpha	Heat shock protein 90			
MGC01452	1	538	Very low-density lipoprotein receptor	LDL receptor-like module ; LOW DENSITY LIPOPROTEIN RECEPTOR ; LDLRA_1 ; Ldl_recept_a ; LDLRECEPTOR ; LDLRA_2 ; LOW-DENSITY LIPOPROTEIN RECEPTOR (LDL)		K04550_05010_Alzheimers disease; K06255_04512_ECM-receptor interaction; K06233_04340_Hedgehog signaling pathway;	3.4.21.109_Matriptase.; 3.4.21.9_Enteropeptidase.;
MGC01455	24	830	Elongation factor 2	TRANSLATION FACTOR ; P-loop containing nucleoside triphosphate hydrolases ; ELONGATNFCT ; EFACTOR_GTP ; GTP_EFTU ; ELONGATION FACTOR 2			
MGC01465	4	706	Collectin-12	C-type lectin-like ; ASIALOGLYCOPROTEIN RECEPTOR ; C_TYPE_LECTIN_1 ; C-TYPE LECTIN SUPERFAMILY MEMBER ; Lectin_C ; C_TYPE_LECTIN_2			
MGC01466	2	943	Serine protease inhibitor dipetalogastin (Fragment)	Kazal_2 ; ORGANIC ANION TRANSPORTER ; KAZAL ; Kazal-type serine protease inhibitors ; ORGANIC ANION TRANSPORTER OATP		K06254_04512_ECM-receptor interaction;	
MGC01470	1	507	Glucose-6-phosphate 1-dehydrogenase X	G6P_DEHYDROGENASE ; Glyceraldehyde-3-phosphate dehydrogenase-like, C-terminal domain ; NAD(P)-binding Rossmann-fold domains ; G6PD_C ; G6PD_N ; GLUCOSE-6-PHOSPHATE 1-DEHYDROGENASE (G6PD) ; G6PDHDRGNASE		K00036_00030_Pentose phosphate pathway; K00036_00480_Glutathione metabolism;	1.1.1.49_Glucose-6-phosphatedehydrogenase.;
MGC01476	25	1011	Collagen alpha-1(I) chain	COLLAGEN ALPHA CHAIN ; Collagen ; Q6NTI5_BRARE_Q6NTI5; ; coiled-coil			
MGC01480	1	422		C-type lectin-like			
MGC01485	1	504	Kinectin	t-snare proteins ; coiled-coil	GO:0005783_C_endoplasmic reticulum; GO:0007018_P_microtubule-based movement; GO:0019894_F_kinesin binding;		
MGC01491	1	511	Ficolin-2	FIBRINOGEN AND FIBRONECTIN ; Fibrinogen C-terminal domain-like ; Fibrinogen_C ; FIBRIN_AG_C_DOMAIN ; FICOLIN			
MGC01524	1	461					
MGC01530	2	670	Collectin-12	C-type lectin-like ; ASIALOGLYCOPROTEIN RECEPTOR ; C_TYPE_LECTIN_1 ; C-TYPE LECTIN SUPERFAMILY MEMBER ; Lectin_C ; C_TYPE_LECTIN_2			
MGC01542	8	615	Hepatic lectin	REGENERATING GENE TYPE IV-RELATED ; C-type lectin-like ; LITHOSTATHINE ; Lectin_C ; C_TYPE_LECTIN_2			
MGC01544	3	552					
MGC01552	1	561	CD209 antigen-like protein E	C-type lectin-like ; ASIALOGLYCOPROTEIN RECEPTOR ; C-TYPE LECTIN SUPERFAMILY MEMBER ; Lectin_C ; C_TYPE_LECTIN_2			
MGC01555	18	616	Integrin beta-PS				
MGC01561	1	601		Galactose-binding domain-like			
MGC01569	1	525	Lectin BRA-3				

MGC01570	5	897	Ankyrin repeat domain-containing protein 45	ANK REPEAT-CONTAINING ; ANKYRIN ; Heat shock protein 70kD (HSP70), C-terminal subdomain ; Ank ; ANKYRIN REPEAT-CONTAINING ; HSP70 ; ANK_REPEAT ; ANK_REP_REGION ; Ankyrin repeat		K09490_05060_tba;	2.7.11.1_Non-specificserine/threonineprotеinkinase.;
MGC01577	4	744	26S proteasome non-ATPase regulatory subunit 10	ANKYRIN ; Ank ; ANKYRIN REPEAT-CONTAINING ; ANK_REPEAT ; ANK_REP_REGION ; 26S PROTEASOME SUBUNIT P28-RELATED ; Ankyrin repeat	GO:0000502_C_proteasome complex; GO:0005838_C_proteasome regulatory particle; GO:0005515_F_protein binding;	K06694_03050_Proteasome;	2.7.11.1_Non-specificserine/threonineproteinkinase.; 2.4.2.30_NAD(+)ADP-ribosyltransferase.; 2.1.1.43_Histone-lysineN-methyltransferase.;
MGC01582	1	595	Uncharacterized protein ybeT	SEL-1-LIKE PROTEIN, SEL-1L ; Sel1 ; HCP-like ; SEL-1-LIKE PROTEIN			
MGC01586	31	1092	Guanine nucleotide-binding protein subunit beta-2-like 1	WD_REPEATS_2 ; GPROTEINBRPT ; WD40 repeat-like ; Q8T6T3_SCHMA_Q8T6T3; ; RECEPTOR FOR ACTIVATED PROTEIN KINASE C (RACK1) ; WD_REPEATS_1 ; WD_REPEATS_REGION ; WD40	GO:0007205_P_activation of protein kinase C activity; GO:0005737_C_cytoplasm; GO:0043025_C_cell soma; GO:0005515_F_protein binding; GO:0005080_F_protein kinase C binding;	K01062_00565_Ether lipid metabolism;	2.7.11.1_Non-specificserine/threonineproteinkinase.; 2.4.1.37_Fucosylgalactoside3-alpha-galactosyltransferase.; 2.3.1.48_Histoneacetyltransferase.; 2.7.11.7_[Myosinheavy-chain]kinase.;
MGC01588	10	699	Mammalian ependymin-related protein 1				
MGC01604	4	1394	26S proteasome non-ATPase regulatory subunit 1	26S PROTEASOME REGULATORY SUBUNIT RPN2 ; 26S PROTEASOME REGULATORY SUBUNIT ; ARM repeat ; PC_rep ; coiled-coil		K03032_03050_Proteasome;	
MGC01609	13	1388	Low-density lipoprotein receptor-related protein 6	LOW DENSITY LIPOPROTEIN RECEPTOR ; LDLRB ; YWTD domain ; SRCR-like ; SRCR ; Ldl_recept_b ; SRCR_2 ; SPERACTRCPTR			
MGC01611	1	560	Collagen alpha-1(III) chain	COLLAGEN ALPHA CHAIN ; Collagen ; COLLAGEN ALPHA CHAIN, TYPE IV			
MGC01612	1	536	CD209 antigen-like protein E	C-type lectin-like ; C-TYPE LECTIN SUPERFAMILY MEMBER ; Lectin_C ; CD209 ANTIGEN (DENDRITIC CELL-SPECIFIC ICAM-3-GRABBING NONINTEGRIN 1) (DC-SIGN1) ; C_TYPE_LECTIN_2			
MGC01614	1	544	Calmodulin	EF_HAND_1 ; CALMODULIN ; EF_HAND_2 ; CALM_EUGGR_P11118; ; EF-hand ; efhand			2.7.11.1_Non-specificserine/threonineproteinkinase.;
MGC01624	10	1537	Protein NEL	ShK ; Fibronectin type I module ; VWFC_2 ; VWFC_1			
MGC01630	1	503		Galactose-binding domain-like			
MGC01632	1	405	Collagen alpha-3(IV) chain	Q8IWL2_HUMAN_Q8IWL2; ; COLLAGEN ALPHA CHAIN ; Collagen ; COLLAGEN ALPHA CHAIN, TYPE IV			
MGC01639	1	496	Spondin-1				
MGC01640	1	643	Kelch-like ECH-associated protein 1	Galactose oxidase, central domain ; KELCH-RELATED PROTEIN ; KELCH-RELATED ; Kelch_1		K10456_04120_Ubiquitin mediated proteolysis;	
MGC01643	2	656		CHITINASE ; BRAIN CHITINASE AND CHIA ; CBM_14 ; CHIT_BIND_II ; Invertebrate chitin-binding proteins			
MGC01646	10	897	Nidogen-1	HLA CLASS II GAMMA CHAIN ; Thyroglobulin_1 ; MHC CLASS II-ASSOCIATED INVARIANT CHAIN-RELATED ; Thyroglobulin type-1 domain ; THYROGLOBULIN_1_2			
MGC01648	14	1034	SH3 domain-containing protein C23A1.17	ShK ; Fibronectin type I module ; VWFC_2 ; CONNECTIVE TISSUE GROWTH FACTOR-RELATED ; VWFC_1			
MGC01652	6	1600	Trichohyalin				

MGC01657	1	287	Perlucin	C-type lectin-like ; C_TYPE_LECTIN_1 ; C-TYPE LECTIN SUPERFAMILY MEMBER ; Lectin_C ; GALACTOSE-SPECIFIC C-TYPE LECTIN ; C_TYPE_LECTIN_2			
MGC01658	9	661	Collagen alpha-1(XIV) chain	VWFA ; VON WILENBRAND FACTOR RELATED ; vWA-like ; VWA	GO:0005578_C_proteinaceous extracellular matrix;		
MGC01677	3	355		VWFA ; INTEGRIN ALPHA-RELATED ; VON WILENBRAND FACTOR RELATED ; vWA-like ; VWA			
MGC01681	1	198	Collagen alpha-5(VI) chain	VWFA ; INTEGRIN ALPHA-RELATED ; VON WILENBRAND FACTOR RELATED ; vWA-like ; VWA ; VWFADOMAIN		K06238_04510_Focal adhesion; K06238_04512_ECM-receptor interaction;	
MGC01686	2	315		DISCOIDIN, CUB, EGF, LAMININ , AND ZINC METALLOPROTEASE DOMAIN ; ZINC METALLOPROTEINASE ; Metalloproteases ('zincins'), catalytic domain ; Astacin			
MGC01688	1	237	Innexin-3	PANNEXIN ; Innexin ; INNEXIN			
MGC01692	1	269	Connective tissue growth factor	Fibronectin type I module ; VWC ; VWFC_2 ; CONNECTIVE TISSUE GROWTH FACTOR-RELATED			
MGC01700	4	695	Mammalian ependymin-related protein 1				
MGC01702	1	218		Immunoglobulin			
MGC01706	26	668	Cytochrome b-c1 complex subunit 7	UCR_14kD ; 14 kDa protein of cytochrome bc1 complex (Ubiquinol-cytochrome c reductase) ; UBIQUINOL-CYTOCHROME C REDUCTASE COMPLEX 14 KD PROTEIN		K00417_00190_Oxidative phosphorylation;	
MGC01716	2	750	Slit homolog 3 protein	LEUCINE-RICH TRANSMEMBRANE PROTEIN ; L domain-like ; LEUCINE-RICH TRANSMEMBRANE PROTEINS		K06838_04360_Axon guidance;	
MGC01720	2	559	ATP synthase subunit delta, mitochondrial	ATP-synt_DE_N ; ATP_synt_epsi: ATP synthase F1, epsilon ; Epsilon subunit of F1F0-ATP synthase C-terminal domain ; ATP SYNTHASE DELTA/EPSILON CHAIN ; coiled-coil ; ATP SYNTHASE DELTA CHAIN, MITOCHONDRIAL ; Epsilon subunit of F1F0-ATP synthase N-terminal domain	GO:0005743_C_mitochondrial inner membrane;	K02134_00190_Oxidative phosphorylation;	3.6.3.14_H(+)-transportingtwo-sectorATPase.;
MGC01733	1	230					
MGC01734	1	449	Keratin-associated protein 5-4				
MGC01735	18	1106	Zinc metalloproteinase nas-13	ZINC METALLOPROTEINASE-RELATED ; DISCOIDIN, CUB, EGF, LAMININ , AND ZINC METALLOPROTEASE DOMAIN ; ZINC_PROTEASE ; Metalloproteases ('zincins'), catalytic domain ; Astacin ; ASTACIN ; MAM_2 ; MAM			3.4.24.21_Astacin.; 3.4.24.18_MeprinA.;
MGC01739	1	434	Gamma-tubulin complex component 2	Spc97_Spc98 ; GAMMA-TUBULIN COMPLEX COMPONENT 2 (GCP-2) ; GAMMA TUBULIN COMPLEX PROTEIN	GO:0005881_C_cytoplasmic microtubule; GO:0005815_C_microtubule organizing center; GO:0007020_P_microtubule nucleation; GO:0006461_P_protein complex assembly;		
MGC01743	3	476	Beta-1,3-glucan-binding protein 1	Concanavalin A-like lectins/glucanases ; GRAM-NEGATIVE BACTERIA BINDING PROTEIN 1 ; SECRETED GLUCOSIDASE-RELATED			
MGC01746	4	1093	Sarcoplasmic calcium-binding protein	EF_HAND_1 ; EF_HAND_2 ; EF-hand			
MGC01752	2	429	von Willebrand factor D and EGF domain-containing protein				

MGC01754	1	354					
MGC01758	1	408	Ras-related protein M-Ras	RAS-RELATED PROTEIN RAP-1 AND 2 ; P-loop containing nucleoside triphosphate hydrolases ; RASTRNSFRMNG ; RAS-RELATED GTPASE ; small_GTP: small GTP-binding protein domain ; Ras		K07830_04010_MAPK signaling pathway; K07830_04530_Tight junction; K07830_04810_Regulation of actin cytoskeleton;	
MGC01761	1	429	Tetranectin-like protein	C-type lectin-like ; C_TYPE_LECTIN_1 ; C-TYPE LECTIN SUPERFAMILY MEMBER ; Lectin_C ; GALACTOSE-SPECIFIC C-TYPE LECTIN ; C_TYPE_LECTIN_2			
MGC01762	1	393	RWD domain-containing protein 4A				
MGC01766	1	306	Transmembrane emp24 domain-containing protein 7	COP-COATED VESICLE MEMBRANE PROTEIN P24 (EMP24/GP25L FAMILY) ; COPII-COATED VESICLE MEMBRANE PROTEIN			
MGC01771	2	399	Butyrate response factor 2	CCCH zinc finger ; CCCH ZINC FINGER/TIS11-RELATED ; zf-CCCH ; TIS11-RELATED			
MGC01775	6	1102	Plasminogen activator inhibitor 1 RNA-binding protein	HABP4_PAI-RBP1 ; PLASMINOGEN ACTIVATOR INHIBITOR 1 RNA-BINDING PROTEIN (PAI1 RNA-BINDING PROTEIN 1) ; HYALURONIC ACID-BINDING PROTEIN 4	GO:0003730_F_mRNA 3{prime}-UTR binding; GO:0005515_F_protein binding; GO:0043488_P_regulation of mRNA stability; GO:0016246_P_RNA interference; GO:0016442_C_RNA-induced silencing complex;		
MGC01777	1	488	G-protein coupled receptor GRL101	LRR_1 ; CHAOPTIN ; L domain-like ; LEURICHRPT ; LEUCINE-RICH TRANSMEMBRANE PROTEINS		K06838_04360_Axon guidance; K06839_04360_Axon guidance; K06850_04360_Axon guidance;	1.11.1.7_Peroxidase.;
MGC01787	2	399					
MGC01792	1	434	Collagen alpha-1(III) chain	COLLAGEN ALPHA CHAIN ; O77087_EEEEE_O77087; ; COLLAGEN ALPHA 2(IX) CHAIN			
MGC01800	1	471	Optineurin	coiled-coil ; L-aspartase-like			
MGC01801	2	992	Growth arrest and DNA damage-inducible protein GADD45 beta	L30e-like ; GADD45 RELATED ; Ribosomal_L7Ae		K04402_04010_MAPK signaling pathway; K04402_04110_Cell cycle; K04402_04115_p53 signaling pathway;	
MGC01802	8	694	Collagen alpha-6(VI) chain	VWFA ; INTEGRIN ALPHA-RELATED ; VON WILENBRAND FACTOR RELATED ; vWA-like ; VWA ; VWFADOMAIN	GO:0030199_P_collagen fibril organization; GO:0030020_F_extracellular matrix structural constituent conferring tensile strength; GO:0005595_C_collagen type XII; GO:0001501_P_skeletal development;	K06238_04510_Focal adhesion; K06238_04512_ECM-receptor interaction;	
MGC01805	1	186	Transmembrane emp24 domain-containing protein 7	EMP24_GP25L ; COP-COATED VESICLE MEMBRANE PROTEIN P24 (EMP24/GP25L FAMILY) ; COPII-COATED VESICLE MEMBRANE PROTEIN ; Supernatant protein factor (SPF), C-terminal domain ; GOLD			
MGC01807	1	186	Translin	Translin ; TRANSLIN AND TRANSLIN ASSOCIATED PROTEIN X ; TRANSLIN			
MGC01825	1	239	Death-associated protein kinase 1	MYOSIN LIGHT CHAIN KINASE-RELATED ; DEATH-ASSOCIATED PROTEIN KINASE ; DEATH domain		K08803_05219_Bladder cancer;	2.7.11.1_Non-specificserine/threonineproteinkinase.;

MGC01828	4	1114	Vascular endothelial growth factor D	Cystine-knot cytokines ; PDGF ; PDGF_2 ; PLATELET-DERIVED GROWTH FACTOR	GO:0005102_F_receptor binding; GO:0005737_C_cytoplasm; GO:0008201_F_heparin binding; GO:0030031_P_cell projection biogenesis; GO:0007298_P_border follicle cell migration; GO:0035099_P_hemocyte migration;		
MGC01833	1	208		heat shock protein 70			
MGC01835	1	334	Macrophage-expressed gene 1 protein				
MGC01845	1	259	Mitogen-activated protein kinase kinase kinase 15	MAPKK-RELATED SERINE/THREONINE PROTEIN KINASES ; MITOGEN ACTIVATED PROTEIN KINASE KINASE KINASE-RELATED	GO:0004702_F_receptor signaling protein serine/threonine kinase activity; GO:0006468_P_protein amino acid phosphorylation; GO:0008360_P_regulation of cell shape;	K04426_04010_MAPK signaling pathway; K04425_04010_MAPK signaling pathway;	2.7.11.25_Mitogen-activatedproteinkinasekinasekinase.;
MGC01854	3	1151	Ankyrin repeat domain-containing protein 57	ANK REPEAT-CONTAINING ; ANKYRIN ; Ank ; ANKYRIN REPEAT-CONTAINING ; ANK_REPEAT ; ANK_REP_REGION ; Ankyrin repeat		K06272_03320_PPAR signaling pathway; K06272_04510_Focal adhesion; K06272_05213_Endometrial cancer;	2.7.11.1_Non-specificserine/threonineproteinkinase.;
MGC01857	1	227	Collagen alpha-1(III) chain	COLLAGEN ALPHA CHAIN ; Collagen ; COLLAGEN ALPHA CHAIN, TYPE IV			
MGC01861	15	1921	Paramyosin	MYOSIN HEAVY CHAIN, SKELETAL MUSCLE OR CARDIAC MUSCLE ; MYOSIN ; coiled-coil ; Domain of the SRP/SRP receptor G-proteins ; Myosin_tail_1		K10352_04530_Tight junction;	
MGC01866	1	431		TNF-like ; coiled-coil			
MGC01877	1	271	Tenascin	FIBRINOGEN AND FIBRONECTIN ; Fibrinogen C-terminal domain-like ; Fibrinogen_C	GO:0007528_P_neuromuscular junction development; GO:0005576_C_extracellular region; GO:0005604_C_basement membrane;	K06252_04510_Focal adhesion; K06252_04512_ECM-receptor interaction;	
MGC01891	1	362	Set1/Ash2 histone methyltransferase complex subunit ASH2	TRITHORAX PROTEIN ASH2			
MGC01897	2	582	26S proteasome non-ATPase regulatory subunit 2	26S PROTEASOME REGULATORY SUBUNIT ; ARM repeat ; 26S PROTEASOME REGULATORY SUBUNIT RPN1 ; PC_rep ; coiled-coil		K03028_03050_Proteasome;	
MGC01898	1	383		HYR ; Complement control module/SCR domain			
MGC01906	2	810	Cysteine-rich secretory protein LCCL domain-containing 2	ShK ; SCP ; CYSTEINE-RICH SECRETORY PROTEIN (CRISP/SCP/TPX1)-RELATED ; V5TPXLIKE ; PR-1-like ; Q6UWH0_HUMAN_Q6UWH0; ; CRISP SUBFAMILY GLIOMA PATHOGENESIS-RELATED PROTEIN-RELATED			
MGC01907	2	842	Calsyntenin-1	Concanavalin A-like lectins/glucanases ; Cadherin-like ; CADHERIN_2 ; CALSYNTENIN			
MGC01909	1	435	Secretory carrier-associated membrane protein 1	SECRETORY CARRIER MEMBRANE PROTEIN ; SECRETORY CARRIER-ASSOCIATED MEMBRANE PROTEIN 1	GO:0030672_C_synaptic vesicle membrane; GO:0008021_C_synaptic vesicle; GO:0042589_C_zymogen granule membrane; GO:0006887_P_exocytosis;		

MGC01916	1	576	Cold shock domain-containing protein E1	UNR PROTEIN (N-RAS UPSTREAM GENE PROTEIN) ; COLD_SHOCK ; Nucleic acid-binding proteins ; CSD	GO:0005737_C_cytoplasm; GO:0003730_F_mRNA 3{prime}-UTR binding; GO:0017148_P_negative regulation of translation; GO:0005515_F_protein binding; GO:0003729_F_mRNA binding; GO:0009047_P_dosage compensation, by hyperactivation of X chromosome;		
MGC01920	6	1111	ATP-dependent RNA helicase DBP2	Q_MOTIF ; P-loop containing nucleoside triphosphate hydrolases ; HELICASE_ATP_BIND_1 ; ATP-DEPENDENT RNA HELICASE DBP3 (YEAST)-RELATED (DEAD BOX POLYPEPTIDE 5, 17, P68) ; DEAD BOX ATP-DEPENDENT RNA HELICASE ; DEAD ; DEAD_ATP_HELICASE	GO:0005515_F_protein binding; GO:0003712_F_transcription cofactor activity; GO:0005634_C_nucleus; GO:0045941_P_positive regulation of transcription;	K01509_00230_Purine metabolism; K01529_00500_Starch and sucrose metabolism; K01529_00790_Folate biosynthesis;	
MGC01925	1	640	60S ribosomal protein L21	Ribosomal_L21e ; Translation proteins SH3-like domain ; 60S RIBOSOMAL PROTEIN L21		K02889_03010_Ribosome;	
MGC01929	1	434	P2X purinoceptor 7	P2X7RECEPTOR ; P2X PURINOCEPTOR ; gb def: P2X7 purinoceptor	GO:0005886_C_plasma membrane; GO:0004931_F_ATP-gated cation channel activity; GO:0006812_P_cation transport;	K05220_04020_Calcium signaling pathway; K05220_04080_Neuroactive ligand-receptor interaction;	
MGC01966	16	770	Baculoviral IAP repeat-containing protein 7-B	BIR_REPEAT_1 ; BIR ; INHIBITOR OF APOPTOSIS ; Inhibitor of apoptosis (IAP) repeat ; INHIBITOR OF APOPTOSIS PROTEIN 1 AND 2, IAP1, IAP2 ; BIR_REPEAT_2		K04725_04120_Ubiquitin mediated proteolysis; K04725_04210_Apoptosis; K04725_04510_Focal adhesion; K04725_05222_Small cell lung cancer;	
MGC01970	1	491	Tumor necrosis factor receptor superfamily member 27	N6_MTASE			
MGC01978	1	563	Ficolin-2	FIBRINOGEN AND FIBRONECTIN ; Fibrinogen C-terminal domain-like ; Fibrinogen_C ; FIBRIN_AG_C_DOMAIN	GO:0005515_F_protein binding; GO:0007411_P_axon guidance; GO:0007155_P_cell adhesion;	K06252_04510_Focal adhesion; K06252_04512_ECM-receptor interaction;	
MGC01985	1	484	40S ribosomal protein S6	RIBOSOMAL_S6E ; Ribosomal_S6e ; 40S RIBOSOMAL PROTEIN S6		K02991_03010_Ribosome; K02991_04150_mTOR signaling pathway; K02991_04910_Insulin signaling pathway;	
MGC01992	1	409	Protein PCOTH	COLLAGEN ALPHA 1(X) CHAIN ; COLLAGEN ALPHA CHAIN ; Collagen ; CA1A_HUMAN_Q03692;			
MGC02011	1	589					
MGC02013	2	746	Collagen alpha-2(I) chain	CA25_HUMAN_P05997; ; 40S RIBOSOMAL PROTEIN S26 ; Ribosomal_S26e			
MGC02019	4	600	Mitochondrial import inner membrane translocase subunit TIM14	DnaJ ; Chaperone J-domain			
MGC02024	2	701	Spondin-1	TSP-1 type 1 repeat			
MGC02030	1	552	Aminopeptidase N	PROTEASE M1 ZINC METALLOPROTEASE ; AMINOPEPTIDASE N-RELATED			3.4.11.2_Membranealanylaminopeptidase.;
MGC02033	9	1286	Heterogeneous nuclear ribonucleoprotein R	RRM ; RRM_1 ; hnRNP-R-Q: hnRNP-R, Q splicing factor famil ; RNA-binding domain, RBD ; RNA-BINDING PROTEIN ; HETEROGENEOUS NUCLEAR RIBONUCLEOPROTEIN R	GO:0005515_F_protein binding;	K02965_03010_Ribosome; K03102_04320_Dorso-ventral axis formation;	5.2.1.8_Peptidylprolylisomerase.;
MGC02040	1	471	Probable G-protein coupled receptor 158				
MGC02047	1	589					
MGC02060	5	717	Uncharacterized protein yxiE	UNVRSLSTRESS ; Adenine nucleotide alpha hydrolases-like ; Usp			
MGC02064	1	494					

MGC02083	1	545	Plexin-A4	E set domains ; PLEXIN	GO:0017154_F_semaphorin receptor activity; GO:0005515_F_protein binding; GO:0016199_P_axon midline choice point recognition; GO:0007411_P_axon guidance; GO:0008045_P_motor axon guidance;	K06820_04360_Axon guidance;	
MGC02087	3	969	Myosin heavy chain, striated muscle	MYOSIN HEAVY CHAIN, SKELETAL MUSCLE OR CARDIAC MUSCLE ; Tubulin chaperone cofactor A ; Ribosomal protein L19 (L19e) ; MYOSIN ; Ribosomal_L19e ; Prefoldin ; coiled-coil ; Myosin_tail_1		K10352_04530_Tight junction;	
MGC02097	1	603	Viral protein TPX	Fibronectin type I module ; VWFC_2 ; VWFC_1			
MGC02106	10	1804	Collagen alpha-5(VI) chain	COCHLIN ; VWFA ; VON WILENBRAND FACTOR RELATED ; vWA-like ; VWA ; VWFADOMAIN		K06238_04510_Focal adhesion; K06238_04512_ECM-receptor interaction; K03900_04510_Focal adhesion; K03900_04512_ECM-receptor interaction; K03900_04610_Complement and coagulation cascades;	
MGC02114	1	276	Scavenger receptor cysteine-rich type 1 protein M130	GALECTIN-3 BINDING PROTEIN ; SRCR-like ; SRCR ; LYSYL OXIDASE-RELATED ; SRCR_2			
MGC02115	1	266					
MGC02119	4	1073	Transmembrane protease serine 3	TRYPSIN_HIS ; SERINE PROTEASE-RELATED ; Trypsin ; TRYPSIN_SER ; CHYMOTRYPSIN ; OVIDUCTIN ; TRYPSIN_DOM ; Trypsin-like serine proteases		K01324_04610_Complement and coagulation cascades;	3.4.21.34_Plasmakallikrein.; 3.4.21.106_Hepsin.;
MGC02120	5	1441	Alpha-amylase	Alpha-amylase_C ; Glycosyl hydrolase domain ; (Trans)glycosidases ; AMYLASE ; ALPHA-AMYLASE ; Alpha-amylase		K01176_00500_Starch and sucrose metabolism;	3.2.1.1_Alpha-amylase.;
MGC02128	9	961	Periostin	FAS1 ; PERIOSTIN-RELATED ; Fasciclin ; FAS1 domain ; TRANSFORMING GROWTH FACTOR-BETA INDUCED PROTEIN IG-H3			
MGC02136	2	802	Beta-1,3-glucan-binding protein 1	Concanavalin A-like lectins/glucanases ; GRAM-NEGATIVE BACTERIA BINDING PROTEIN 1 ; SECRETED GLUCOSIDASE-RELATED ; Glyco_hydro_16		K01238_00530_Aminosugars metabolism;	3.2.1.73_Licheninase.; 3.2.1.39_Glucanendo-1,3-beta-D-glucosidase.;
MGC02139	2	608	C-type lectin domain family 4 member M	C-type lectin-like ; C_TYPE_LECTIN_1 ; C-TYPE LECTIN SUPERFAMILY MEMBER ; Lectin_C ; CD209 ANTIGEN (DENDRITIC CELL-SPECIFIC ICAM-3-GRABBING NONINTEGRIN 1) (DC-SIGN1) ; ANTIFREEZEII ; C_TYPE_LECTIN_2			
MGC02140	1	410		CYSTATIN-RELATED ; Cystatin/monellin ; CYSTATIN FAMILY MEMBER			
MGC02143	4	1189	Collagen alpha-1(XII) chain	ShK ; VWFA ; INTEGRIN ALPHA-RELATED ; VON WILENBRAND FACTOR RELATED ; vWA-like ; VWA ; VWFADOMAIN	GO:0030199_P_collagen fibril organization; GO:0030020_F_extracellular matrix structural constituent conferring tensile strength; GO:0005595_C_collagen type XII; GO:0001501_P_skeletal development;		
MGC02148	4	792					

MGC02153	1	564	Lachesin	Immunoglobulin ; IG_LIKE ; ig ; V-set ; NEUROTRACTING/LSAMP/NEUROTRIMIN/OBCAM RELATED CELL ADHESION MOLECULE ; CPSASE_2 ; LACHESIN		K06775_04514_Cell adhesion molecules (CAMs);	2.7.11.1_Non-specificserine/threonineproteinkinase.; 2.7.10.1_Receptorprotein-tyrosinekinase.;
MGC02157	28	909	Chymotrypsin-like serine proteinase	TRYPSIN_HIS ; SERINE PROTEASE-RELATED ; Trypsin ; TRYPSIN_SER ; CHYMOTRYPSIN ; SERINE PROTEASE-RELATED, INSECT ; TRYPSIN_DOM ; Trypsin-like serine proteases		K01312_04080_Neuroactive ligand-receptor interaction;	3.4.21.1_Chymotrypsin.; 3.4.21.4_Trypsin.;
MGC02164	1	420	78 kDa glucose-regulated protein	Heat shock protein 70kD (HSP70), peptide-binding domain ; HEAT SHOCK PROTEIN 70KDA ; HEAT SHOCK PROTEIN 70 (HSP70) ; HSP70 ; Actin-like ATPase domain ; HSP70_3 ; HEATSHOCK70		K09490_05060_tba;	
MGC02169	16	880	Ovochymase-1	TRYPSIN_HIS ; SERINE PROTEASE-RELATED ; PTS_HPR_HIS ; Trypsin ; TRYPSIN_SER ; CHYMOTRYPSIN ; OVIDUCTIN ; TRYPSIN_DOM ; Trypsin-like serine proteases		K01323_04610_Complement and coagulation cascades;	3.4.21.34_Plasmakallikrein.; 3.4.21.9_Enteropeptidase.; 3.4.21.7_Plasmin.; 3.4.21.27_CoagulationfactorXIa.;
MGC02171	10	817	Complement C1q-like protein 4				
MGC02186	10	883	Chymotrypsin-like serine proteinase	TRYPSIN_HIS ; SERINE PROTEASE-RELATED ; Trypsin ; TRYPSIN_SER ; CHYMOTRYPSIN ; SERINE PROTEASE-RELATED, INSECT ; TRYPSIN_DOM ; Trypsin-like serine proteases		K01324_04610_Complement and coagulation cascades;	3.4.21.34_Plasmakallikrein.; 3.4.21.1_Chymotrypsin.;
MGC02194	1	368					
MGC02198	1	429	Complement C1q-like protein 4	C1q ; COLLAGEN ALPHA CHAIN ; COMPLEMNTC1Q ; gb def: Mus musculus adult male cecum cDNA, RIKEN full-length enriched library, clone:91 ; TNF-like ; C1Q		K03987_04610_Complement and coagulation cascades; K03987_05010_Alzheimers disease; K03988_04610_Complement and coagulation cascades; K03988_05010_Alzheimers disease;	
MGC02203	2	792	CD109 antigen	A2M_recep ; Alpha-macroglobulin receptor domain ; MACROGLOBULIN/COMPLEMENT		K03989_04610_Complement and coagulation cascades; K03910_04610_Complement and coagulation cascades; K03910_05010_Alzheimers disease;	
MGC02206	1	518	Transmembrane protease serine 2	SERINE PROTEASE-RELATED ; Trypsin ; SERINE PROTEASE-RELATED, INSECT ; TRYPSIN_DOM ; Trypsin-like serine proteases		K01334_04610_Complement and coagulation cascades;	3.4.21.46_ComplementfactorD.; 3.4.21.68_T-plasminogenactivator.; 3.4.21.9_Enteropeptidase.;
MGC02230	1	586	Methylosome protein 50	WD_REPEATS_2 ; METHYLOSOME PROTEIN 50 (MEP50 PROTEIN) ; GPROTEINBRPT ; ZINC_FINGER_C2H2_1 ; WD40 REPEAT FAMILY ; PEROXIDASE_1 ; WD40 repeat-like ; WD_REPEATS_1 ; WD_REPEATS_REGION ; WD40		K01062_00565_Ether lipid metabolism;	2.3.1.48_Histoneacetyltransferase.;
MGC02236	2	721	Collagen alpha-5(VI) chain	VWFA ; VON WILENBRAND FACTOR RELATED ; vWA-like ; VWA ; VWFADOMAIN		K06238_04510_Focal adhesion; K06238_04512_ECM-receptor interaction;	
MGC02247	2	768	Vimentin beta	coiled-coil			
MGC02249	1	295	Sorbin and SH3 domain-containing protein 2	DCAPL ; SH3-domain ; SH3 MULTI DOMAIN CONTAINING PROTEIN, ENDOPHILIN ; P67PHOX ; SH3 ; SH3DOMAIN ; SH3_1 ; Q8CHU0_MOUSE_Q8CHU0; ; SPECTRNALPHA		K06086_03320_PPAR signaling pathway; K06086_04520_Adherens junction; K06086_04910_Insulin signaling pathway;	
MGC02253	1	536	Calmodulin	EF_HAND_1 ; CALMODULIN ; EF_HAND_2 ; CALM_EUGGR_P11118; ; EF-hand ; CALCIUM BINDING PROTEIN ; efhand			2.7.11.1_Non-specificserine/threonineproteinkinase.;

MGC02260	7	1308	Collagen alpha-6(VI) chain	COCHLIN ; VWFA ; VON WILENBRAND FACTOR RELATED ; vWA-like ; VWA ; VWFADOMAIN		K06238_04510_Focal adhesion; K06238_04512_ECM-receptor interaction;	
MGC02264	2	582	Plasminogen	KRINGLE_1 ; SERINE PROTEASE-RELATED ; Kringle-like ; PLMN_PIG_P06867; ; PLASMINOGEN ; KRINGLE_2 ; KRINGLE ; Kringle		K01315_04080_Neuroactive ligand-receptor interaction; K01315_04610_Complement and coagulation cascades;	3.4.21.7_Plasmin.;
MGC02267	2	946	Heat shock cognate 71 kDa protein	Heat shock protein 70kD (HSP70), peptide-binding domain ; HEAT SHOCK PROTEIN 70KDA ; HEAT SHOCK PROTEIN 70 (HSP70) ; HSP70 ; Actin-like ATPase domain ; coiled-coil ; HSP70_3 ; HEATSHOCK70		K03283_04010_MAPK signaling pathway; K03283_04612_Antigen processing and presentation;	
MGC02276	9	763	Collagen alpha-1(XII) chain	VWFA ; VON WILENBRAND FACTOR RELATED ; vWA-like ; VWA ; VWFADOMAIN	GO:0030199_P_collagen fibril organization; GO:0030020_F_extracellular matrix structural constituent conferring tensile strength; GO:0005595_C_collagen type XII; GO:0001501_P_skeletal development;		3.4.21.43_Classical-complement-pathwayC3/C5convertase.;
MGC02287	13	994	Prohibitin	Band_7 ; PROHIBITIN	GO:0005515_F_protein binding; GO:0005739_C_mitochondrion; GO:0005743_C_mitochondrial inner membrane;		
MGC02318	1	693		Fibronectin type III			
MGC02324	1	521	DNA ligase 1	coiled-coil			
MGC02331	24	1020	Vegetative cell wall protein gp1	COLLAGEN ALPHA CHAIN ; Collagen ; COLLAGEN ALPHA-RELATED			
MGC02335	7	755	Macrophage mannose receptor 1	C-type lectin-like ; ASIALOGLYCOPROTEIN RECEPTOR ; C-TYPE LECTIN SUPERFAMILY MEMBER ; Lectin_C ; ANTIFREEZEII ; C_TYPE_LECTIN_2			
MGC02336	1	635	Collagen alpha-1(XXI) chain	VWFA ; VON WILENBRAND FACTOR RELATED ; vWA-like ; VWA			
MGC02341	6	1288	Tubulin beta chain	Tubulin_C ; Tubulin ; BETATUBULIN ; TUBULIN BETA CHAIN ; Tubulin nucleotide-binding domain-like ; TONB_DEPENDENT_REC_1 ; Tubulin C-terminal domain-like ; coiled-coil ; TUBULIN		K07375_04540_Gap junction;	
MGC02346	1	556	Kallikrein-13	SERINE PROTEASE-RELATED ; Trypsin ; CHYMOTRYPSIN ; SERINE PROTEASE-RELATED, INSECT ; TRYPSIN_DOM ; Trypsin-like serine proteases			3.4.21.9_Enteropeptidase.; 3.4.21.27_CoagulationfactorXIa.; 3.4.21.4_Trypsin.;
MGC02354	1	642	Activin receptor type-2A	Protein kinase-like (PK-like) ; Pkinase ; TRANSFORMING GROWTH FACTOR-BETA RECEPTOR TYPE I AND II ; Q8BRS2_MOUSE_Q8BRS2; ; ACTIVIN RECEPTOR TYPE II ; PROTEIN_KINASE_DOM	GO:0008544_P_epidermis development; GO:0051216_P_cartilage development; GO:0001755_P_neural crest cell migration; GO:0001501_P_skeletal development; GO:0030902_P_hindbrain development;	K04670_04060_Cytokine-cytokine receptor interaction; K04670_04350_TGF-beta signaling pathway;	2.7.11.30_Receptorproteinserine/threoninekinase.;
MGC02371	1	429					
MGC02373	1	537	Zinc finger CCCH domain-containing protein 13	coiled-coil			
MGC02376	1	476	40S ribosomal protein S19	Ribosomal_S19e ; 40S RIBOSOMAL PROTEIN S19		K02966_03010_Ribosome;	
MGC02384	1	451	Down syndrome cell adhesion molecule	Immunoglobulin ; IG_LIKE ; I-set ; TITIN	GO:0007399_P_nervous system development; GO:0005887_C_integral to plasma membrane; GO:0005624_C_membrane fraction; GO:0005886_C_plasma membrane; GO:0007155_P_cell adhesion;	K05456_04012_ErbB signaling pathway; K05098_04060_Cytokine-cytokine receptor interaction; K05098_04370_VEGF signaling pathway; K05098_04510_Focal adhesion;	2.7.11.1_Non-specificserine/threonineproteinkinase.; 2.7.10.1_Receptorprotein-tyrosinekinase.;

MGC02386	1	728	Aldehyde dehydrogenase, mitochondrial	ALDEHYDE DEHYDROGENASE-RELATED ; ALDEHYDE DEHYDROGENASE ; ALDH-like ; Aldedh	GO:0005739_C_mitochondrion;		1.2.1.3_Aldehydedehydrogenase(NAD(+)).;
MGC02412	1	631	Cysteine-rich motor neuron 1 protein	ANTISTASIN ; TNFR_NGFR_1 ; EXTRACELLULAR MATRIX GLYCOPROTEIN RELATED ; CYSTEINE-RICH MOTOR NEURON 1 ; Antistasin ; Leech antihemostatic proteins	GO:0001568_P_blood vessel development; GO:0001756_P_somitogenesis; GO:0048570_P_notochord morphogenesis;		
MGC02414	2	1079	Ankyrin repeat and SAM domain-containing protein 6	ANK_REP_REGION ; Ankyrin repeat			
MGC02419	1	649	Galectin-4	Concanavalin A-like lectins/glucanases ; GALECTIN ; Gal-bind_lectin			3.1.1.5_Lysophospholipase.;
MGC02425	17	930	Chitotriosidase-1	CHITINASE ; Chitinase insertion domain ; BRAIN CHITINASE AND CHIA ; (Trans)glycosidases ; CHIT_BIND_II ; Glyco_hydro_18 ; Invertebrate chitin-binding proteins	GO:0005615_C_extracellular space; GO:0008061_F_chitin binding; GO:0006032_P_chitin catabolic process; GO:0004568_F_chitinase activity; GO:0006955_P_immune response; GO:0009617_P_response to bacterium;	K01183_00530_Aminosugars metabolism;	3.2.1.14_Chitinase.;
MGC02428	1	540	Immediate-early protein	coiled-coil			
MGC02429	1	702	FACT complex subunit SSRP1	HMG_box ; HMG-box ; SWI/SNF-RELATED CHROMATIN BINDING PROTEIN ; HMG_BOX_2 ; HIGHMOBLTY12			
MGC02452	1	589	Muscle M-line assembly protein unc-89	Immunoglobulin ; IG_LIKE ; I-set ; MYOMESIN ; TITIN	GO:0007498_P_mesoderm development; GO:0006468_P_protein amino acid phosphorylation; GO:0004674_F_protein serine/threonine kinase activity;	K00907_04020_Calcium signaling pathway; K00907_04510_Focal adhesion; K00907_04810_Regulation of actin cytoskeleton; K06759_04514_Cell adhesion molecules (CAMs);	2.7.11.1_Non-specificserine/threonineproteinkinase.; 2.7.11.18_[Myosinlight-chain]kinase.;
MGC02454	1	642	Collagen alpha-1(V) chain	Q8MW53_MYTGA_Q8MW53; ; COLLAGEN ALPHA CHAIN ; Collagen			
MGC02455	2	908	Collagen alpha-1(V) chain	Ribosomal protein S7 ; Collagen ; Ribosomal_S7 ; 40S RIBOSOMAL PROTEIN S5 ; Q86G64_DERVA_Q86G64; ; O44367_MYTED_O44367; ; RIBOSOMAL PROTEIN S7		K02989_03010_Ribosome;	
MGC02456	13	1192	Cathepsin L1	Cysteine proteinases ; Q86GJ2_HYDAT_Q86GJ2; ; THIOL_PROTEASE_CYS ; Inhibitor_I29 ; Peptidase_C1 ; PAPAIN ; THIOL_PROTEASE_HIS ; CYSTEINE PROTEASE FAMILY C1-RELATED ; CATHEPSIN L ; THIOL_PROTEASE_ASN		K01365_04612_Antigen processing and presentation;	3.4.22.15_CathepsinL.;
MGC02457	2	817	PDZ and LIM domain protein 4	PDZ ; LIM DOMAIN CONTAINING PROTEIN ; PDZ domain-like	GO:0007498_P_mesoderm development;	K05631_04530_Tight junction; K05631_05050_tba; K05629_04530_Tight junction; K05629_05050_tba;	
MGC02462	1	653	Uncharacterized protein DDB_G0271670				
MGC02465	1	702	Ferritin, middle subunit	FERRITIN_LIKE ; FERRITIN ; Q7YZR8_CRAGI_Q7YZR8; ; Ferritin ; Ferritin-like		K00522_00860_Porphyrin and chlorophyll metabolism;	1.16.3.1_Ferroxidase.;
MGC02476	1	485	Stress-associated endoplasmic reticulum protein 2	RAMP4 ; STRESS ASSOCIATED ENDOPLASMIC RETICULUM PROTEIN (SERP1/RAMP4)			
MGC02498	1	207	Adhesive plaque matrix protein 2	EGF/Laminin ; NOTCH ; EGF-LIKE DOMAIN PROTEIN ; EGF ; EGF_3 ; EGF_1 ; EGF_2		K06051_04330_Notch signaling pathway; K06052_04330_Notch signaling pathway;	
MGC02500	1	413	Whey acidic protein	WHEY ACDIC PROTEIN (WAP) ; Antistasin ; Leech antihemostatic proteins ; WAP ; 4DISULPHCORE ; Elafin-like			

MGC02504	1	331	Phosphatidylinositol-binding clathrin assembly protein LAP	ANTH ; GAT-like domain ; CLATHRIN ASSEMBLY PROTEIN ; PHOSPHATIDYLINOSITOL-BINDING CLATHRIN ASSEMBLY PROTEIN			
MGC02505	2	561	Complement C1q tumor necrosis factor-related protein 6	C1q ; COLLAGEN ALPHA CHAIN ; COMPLEMENT C1Q AND TUMOR NECROSIS FACTOR RELATED PROTEIN 6 ; TNF-like			
MGC02510	2	655	Homeobox protein ARX	Homeobox ; HOMEOBOX PROTEIN ; HOMEOBOX_1 ; HOMEOBOX_2 ; OTP_DROME_P56672; ; HOMEOBOX PROTEIN ARISTALESS-RELATED ; Homeodomain-like ; HTHREPRESSR ; HOMEOBOX			
MGC02559	2	1070	Collagen alpha-1(III) chain	MRCO_MESAU_Q9WUB9; ; COLLAGEN ALPHA CHAIN ; Collagen ; COLLAGEN ALPHA CHAIN, TYPE IV			
MGC02581	1	555	GTP-binding nuclear protein Ran	GTP-BINDING NUCLEAR PROTEIN RAN ; P-loop containing nucleoside triphosphate hydrolases ; RASTRNSFRMNG ; RAS-RELATED GTPASE ; RAN ; small_GTP: small GTP-binding protein domain ; GTPRANTC4 ; Ras			2.7.11.1_Non-specificserine/threonineprotein kinase.;
MGC02586	1	724	Cell division protein kinase 5	Protein kinase-like (PK-like) ; PROTEIN_KINASE_ST ; CDK5 ; Pkinase ; CDC2, MAP KINASE-RELATED ; Q6V5R4_EMENI_Q6V5R4; ; PROTEIN_KINASE_DOM		K02090_04360_Axon guidance;	2.7.11.22_Cyclin-dependentkinase.;
MGC02596	1	441	Peptidyl-prolyl cis-trans isomerase CYP19-3	PEPTIDYL-PROLYL CIS-TRANS ISOMERASE F, PPIF ; CSA_PPIASE_1 ; Cyclophilin-like ; CSAPPISMRASE ; CYCLOPHILIN ; Pro_isomerase ; CSA_PPIASE_2			5.2.1.8_Peptidylprolylisomerase.;
MGC02600	1	498					
MGC02605	1	689	Protein still life, isoforms C/SIF type 2	STILL LIFE PROTEIN, SIF (T-LYMPHOMA INVASION AND METASTASIS INDUCING PROTEIN) (TIAM PROTEIN) ; RHO GUANINE EXCHANGE FACTOR-RELATED ; PH domain-like		K05731_04810_Regulation of actin cytoskeleton;	
MGC02622	1	643	Collagen alpha-1(IV) chain	COLLAGEN ALPHA CHAIN ; Collagen ; COLLAGEN ALPHA CHAIN, TYPE IV ; Q810J9_MOUSE_Q810J9;		K06237_04510_Focal adhesion; K06237_04512_ECM-receptor interaction; K06237_05222_Small cell lung cancer;	
MGC02643	1	620	Ubiquitin	UBIQUITIN (RIBOSOMAL PROTEIN L40) ; UBIQUITIN_2 ; Ubiquitin-like ; ubiquitin ; UBIQUITIN_1 ; UBIQUITIN		K02977_03010_Ribosome; K08770_03320_PPAR signaling pathway; K02927_03010_Ribosome; K04551_05020_tba;	
MGC02650	1	453	Protein pellino	RING/U-box ; Pellino ; SUBFAMILY NOT NAMED	GO:0016020_C_membrane; GO:0005515_F_protein binding; GO:0019207_F_kinase regulator activity; GO:0005829_C_cytosol ; GO:0008063_P_Toll signaling pathway; GO:0043234_C_protein complex; GO:0006955_P_immune response;		
MGC02668	1	422		Immunoglobulin ; IG_LIKE			
MGC02678	7	1204	Voltage-dependent anion-selective channel protein 2	VOLTAGE-DEPENDENT ANION-SELECTIVE CHANNEL ; EUKARYTPORIN ; Porin_3 ; VOLTAGE-DEPENDENT ANION-SELECTIVE CHANNEL (PORIN PROTEIN) (DMVDAC)		K05862_04020_Calcium signaling pathway;	

MGC02691	1	649	Collagen alpha-1(V) chain	COLLAGEN ALPHA CHAIN ; Collagen ; COLLAGEN ALPHA CHAIN, TYPE IV			
MGC02701	1	728	Phytanoyl-CoA dioxygenase domain-containing protein 1 homolog	PhyH ; PHYTANOYL-COA DIOXYGENASE DOMAIN CONTAINING 1			
MGC02703	1	633	Techylectin-5B	FIBRINOGEN AND FIBRONECTIN ; Fibrinogen C-terminal domain-like ; Fibrinogen_C ; FIBRIN_AG_C_DOMAIN ; FICOLIN	GO:0016337_P_cell-cell adhesion; GO:0005529_F_sugar binding;		
MGC02711	1	512	Annulin	E set domains ; Transglut_N ; PROTEIN-GLUTAMINE GAMMA-GLUTAMYLTRANSFERASE ; COAGULATION FACTOR XIII A CHAIN	GO:0007596_P_blood coagulation; GO:0005576_C_extracellular region; GO:0003810_F_protein-glutamine gamma-glutamyltransferase activity;	K03917_04610_Complement and coagulation cascades;	2.3.2.13_Protein-glutaminegamma-glutamyltransferase.;
MGC02728	1	486	Protein CBFA2T1	TAFH ; ETO/MGT8-RELATED 2 ; ETO/MTG8/NERVY ; ETOFAMILY	GO:0008283_P_cell proliferation;	K03129_03022_Basal transcription factors; K10053_05221_Acute myeloid leukemia;	
MGC02730	1	415	Collectin-12	Q7Z5L5_HUMAN_Q7Z5L5; ; COLLAGEN ALPHA CHAIN ; Collagen ; COLLAGEN ALPHA-RELATED			
MGC02732	1	512	Tumor protein 63	TUMOR PROTEIN P73 ; CELLULAR TUMOR ANTIGEN P53-RELATED ; P53_tetramer ; P53 ; p53-like transcription factors ; p53 tetramerization domain ; P53SUPPRESSR			
MGC02733	1	643		HSP20-like chaperones			
MGC02741	1	419	Desert hedgehog protein	SONIC HEDGEHOG ; HEDGEHOG			
MGC02744	3	685	Proteasome subunit alpha type-2	N-terminal nucleophile aminohydrolases (Ntn hydrolases) ; Proteasome ; PROTEASOME SUBUNIT ALPHA/BETA ; PROTEASOME_A ; PROTEASOME SUBUNIT ALPHA TYPE 2		K02726_03050_Proteasome;	3.4.25.1_Proteasomeendopeptidasecomplex.;
MGC02773	1	602	Proteasome subunit alpha type-5	N-terminal nucleophile aminohydrolases (Ntn hydrolases) ; Proteasome ; PROTEASOME SUBUNIT ALPHA TYPE 5 ; PROTEASOME SUBUNIT ALPHA/BETA		K02729_03050_Proteasome;	3.4.25.1_Proteasomeendopeptidasecomplex.;
MGC02794	1	411	Collagen alpha-2(VIII) chain	C1q ; GLIACOLIN-RELATED ; CEREBELLIN-RELATED ; COMPLEMNTC1Q ; TNF-like ; C1Q		K03986_04610_Complement and coagulation cascades; K03986_05010_Alzheimers disease; K03987_04610_Complement and coagulation cascades; K03987_05010_Alzheimers disease;	
MGC02800	1	571	Myeloid leukemia factor 2				
MGC02813	1	555	Sushi, nidogen and EGF-like domain-containing protein 1	EGF/Laminin ; EGF_3 ; ASX_HYDROXYL ; SUSHI ; COMPLEMENT COMPONENT-RELATED SUSHI DOMAIN-CONTAINING ; CUB AND SUSHI MULTIPLE DOMAINS PROTEIN ; EGF_1 ; Complement control module/SCR domain ; EGF_2 ; Sushi			
MGC02825	2	661	Macrophage mannose receptor 1	C-type lectin-like ; C_TYPE_LECTIN_1 ; C-TYPE LECTIN SUPERFAMILY MEMBER ; Lectin_C ; GALACTOSE-SPECIFIC C-TYPE LECTIN ; coiled-coil ; C_TYPE_LECTIN_2			
MGC02833	3	697	Heavy metal-binding protein HIP	COMPLEMENT C1Q TUMOR NECROSIS FACTOR-RELATED PROTEIN 4 ; C1q ; COLLAGEN ALPHA CHAIN ; COMPLEMNTC1Q ; TNF-like ; coiled-coil ; C1Q	GO:0030023_F_extracellular matrix constituent conferring elasticity; GO:0005515_F_protein binding; GO:0005576_C_extracellular region;	K03986_04610_Complement and coagulation cascades; K03986_05010_Alzheimers disease;	

MGC02853	1	608	Cytochrome b-c1 complex subunit 7	UCR_14kD ; 14 kDa protein of cytochrome bc1 complex (Ubiquinol-cytochrome c reductase) ; UBIQUINOL-CYTOCHROME C REDUCTASE COMPLEX 14 KD PROTEIN		K00417_00190_Oxidative phosphorylation;	
MGC02854	4	951	Suppressor of tumorigenicity 14 protein	Serine proterase inhibitors ; LDL receptor-like module ; LDLRA_1 ; LDLRA_2 ; coiled-coil			
MGC02855	1	562	Protein O-mannosyl-transferase 2	MIR domain (Pfam 02815) ; DOLICHYL-PHOSPHATE-MANNOSE--PROTEIN MANNOSYLTRANSFERASE ; MIR	GO:0005783_C_endoplasmic reticulum; GO:0030259_P_lipid glycosylation; GO:0005515_F_protein binding; GO:0004169_F_dolichyl-phosphate-mannose-protein mannosyltransferase activity; GO:0007517_P_muscle development;		2.4.1.109_Dolichyl-phosphate-mannose-proteinmannosyltransferase.;
MGC02856	1	539	Nebulin	SH3-domain ; Q8N8M3_HUMAN_Q8N8M3; ; SH3 ; SH3DOMAIN ; SH3_1 ; NEBULIN	GO:0030832_P_regulation of actin filament length; GO:0008307_F_structural constituent of muscle;		
MGC02872	2	1112	Nucleoprotein TPR	NUCLEOPROTEIN TPR-RELATED ; coiled-coil	GO:0005635_C_nuclear envelope;	K09291_05216_Thyroid cancer;	
MGC02873	1	632	Piwi-like protein 1	PIWIL1 ; PIWI ; PIWI-RELATED ; Ribonuclease H-like ; Piwi	GO:0005737_C_cytoplasm; GO:0005515_F_protein binding; GO:0007283_P_spermatogenesis; GO:0003727_F_single-stranded RNA binding;	K02156_04320_Dorso-ventral axis formation;	
MGC02880	1	532	60S ribosomal protein L7a	RIBOSOMALL7A ; L30e-like ; 60S RIBOSOMAL PROTEIN L7A ; RIBOSOMAL_L7AE ; RIBOSOMAL PROTEIN L7AE FAMILY MEMBER ; L7ARS6FAMILY ; Ribosomal_L7Ae		K02936_03010_Ribosome;	
MGC02882	1	649	Fibulin-2	EGF/Laminin ; EGF-LIKE DOMAIN PROTEIN ; EGF ; EGF_3 ; ASX_HYDROXYL ; EGF_2 ; ANAPHYLATOXIN_2 ; EGF_CA	GO:0005515_F_protein binding;		
MGC02887	1	573	Neuromacin-like protein	Scorpion toxin-like			
MGC02899	2	1068	Fibrinogen C domain-containing protein 1	FIBRINOGEN AND FIBRONECTIN ; Fibrinogen C-terminal domain-like ; Fibrinogen_C ; FIBRIN_AG_C_DOMAIN	GO:0007411_P_axon guidance; GO:0007155_P_cell adhesion;	K06252_04510_Focal adhesion; K06252_04512_ECM-receptor interaction;	
MGC02902	1	676					
MGC02918	1	726	CUE domain-containing protein 1	UBA-like ; CUE ; CUE DOMAIN CONTAINING PROTEIN 1			
MGC02931	1	699	Probable ATP-dependent RNA helicase DDX53	DEAD (ASP-GLU-ALA-ASP) BOX POLYPEPTIDE 43 ; P-loop containing nucleoside triphosphate hydrolases ; HELICASE_ATP_BIND_1 ; DEAD BOX ATP-DEPENDENT RNA HELICASE ; DEAD		K01529_00500_Starch and sucrose metabolism; K01529_00790_Folate biosynthesis;	
MGC02960	2	380	E-selectin	C-type lectin-like ; C_TYPE_LECTIN_1 ; Lectin_C ; C-TYPE LECTIN PROTEINS ; C_TYPE_LECTIN_2			
MGC02970	6	887	Fibrinogen C domain-containing protein 1	FIBRINOGEN AND FIBRONECTIN ; Fibrinogen C-terminal domain-like ; Fibrinogen_C ; FIBRIN_AG_C_DOMAIN		K06252_04510_Focal adhesion; K06252_04512_ECM-receptor interaction;	
MGC02986	2	472	Lysozyme	Lys ; LACTALBUMIN_LYSOZYME ; LYZLACT ; LYSOZYME C ; Lysozyme-like			3.2.1.17_Lysozyme.;
MGC02989	2	477	Collagen alpha-1(III) chain	Q8MW53_MYTGA_Q8MW53; ; COLLAGEN ALPHA CHAIN ; Collagen ; COLLAGEN ALPHA CHAIN, TYPE IV			
MGC03002	2	587					
MGC03021	6	764					

MGC03024	1	662					
MGC03039	1	398	Serine/threonine-protein kinase SIK2	UBA-like ; CALCIUM/CALMODULIN-DEPENDENT PROTEIN KINASE-RELATED ; UBA ; SERINE/THREONINE-PROTEIN KINASE SNF1-LIKE KINASE 1,2			2.7.11.1_Non-specificserine/threonineprotein kinase.;
MGC03064	17	644	Cerebellin-2	C1q ; CEREBELLIN-RELATED ; COMPLEMNTC1Q ; TNF-like ; C1Q			
MGC03065	2	577	Ubiquitin	UBIQUITIN (RIBOSOMAL PROTEIN L40) ; UBIQUITIN_2 ; Ubiquitin-like ; ubiquitin ; UBIQUITIN_1 ; UBIQUITIN		K02977_03010_Ribosome; K08770_03320_PPAR signaling pathway; K02927_03010_Ribosome; K04551_05020_tba;	
MGC03068	1	705	Whey acidic protein	WHEY ACDIC PROTEIN (WAP) ; 4_DISULFIDE_CORE ; WAP ; 4DISULPHCORE ; Elafin-like			
MGC03114	1	388		TNF-like			
MGC03116	1	468	Proliferating cell nuclear antigen 2	DNA clamp ; PROLIFERATING CELL NUCLEAR ANTIGEN ; PCNACYCLIN ; PCNA_1 ; PCNA_N		K04802_03030_DNA replication; K04802_03410_Base excision repair; K04802_03420_Nucleotide excision repair; K04802_03430_Mismatch repair; K04802_04110_Cell cycle;	
MGC03165	4	1001	Caprin-2	C1q ; GLIACOLIN-RELATED ; CEREBELLIN-RELATED ; COMPLEMNTC1Q ; TNF-like ; C1Q	GO:0009617_P_response to bacterium; GO:0006950_P_response to stress; GO:0005634_C_nucleus; GO:0005737_C_cytoplasm; GO:0005739_C_mitochondrion; GO:0030308_P_negative regulation of cell growth;		
MGC03168	4	1342	14-3-3-like protein 2	O96945_GEOCY_O96945; ; 14-3-3 ; 1433ZETA ; coiled-coil ; 1433_1 ; 14-3-3 protein	GO:0005515_F_protein binding; GO:0006605_P_protein targeting; GO:0005739_C_mitochondrion; GO:0005634_C_nucleus; GO:0019904_F_protein domain specific binding;	K06630_04110_Cell cycle;	
MGC03192	1	535					
MGC03195	2	1012	Krueppel-like factor 7	Q7Z3H8_HUMAN_Q7Z3H8; ; ZINC_FINGER_C2H2_1 ; C2H2 and C2HC zinc fingers ; zf-C2H2 ; KRUPPEL-LIKE FACTOR 4 (EPITHELIAL ZINC-FINGER PROTEIN EZF) ; KRUEPPEL C2H2-TYPE ZINC-FINGER ; ZINC_FINGER_C2H2_2	GO:0005515_F_protein binding;		
MGC03210	10	1273	Cysteine-rich motor neuron 1 protein	CYR61/CCN1 ; ShK ; Fibronectin type I module ; VWC ; VWFC_2 ; CONNECTIVE TISSUE GROWTH FACTOR-RELATED ; VWFC_1			
MGC03226	3	977	Ficolin-2	FIBRINOGEN AND FIBRONECTIN ; Fibrinogen C-terminal domain-like ; Fibrinogen_C		K06252_04510_Focal adhesion; K06252_04512_ECM-receptor interaction;	
MGC03227	2	618	Serine protease inhibitor dipetalogastin (Fragment)	Kazal_2 ; KAZAL ; SECRETORY TRYPSIN INHIBITOR ; SERINE PROTEASE INHIBITOR ; Kazal-type serine protease inhibitors			
MGC03231	1	525					
MGC03246	5	573	ATP-dependent RNA helicase DDX42	DEAD (ASP-GLU-ALA-ASP) BOX POLYPEPTIDE 42 ; P-loop containing nucleoside triphosphate hydrolases ; DEAD BOX ATP-DEPENDENT RNA HELICASE		K01529_00500_Starch and sucrose metabolism; K01529_00790_Folate biosynthesis;	
MGC03313	2	583	Deleted in malignant brain tumors 1 protein	CUB ; Spermadhesin, CUB domain ; LYSYL OXIDASE-RELATED ; DELETED IN MALIGNANT BRAIN TUMORS 1			3.4.24.21_Astacin.;
MGC03318	3	821					

MGC03364	1	679	Protein phosphatase 1 regulatory inhibitor subunit 16B	ANKYRIN ; Ank ; ANKYRIN REPEAT-CONTAINING ; ANK_REPEAT ; PROTEIN PHOSPHATASE 1 REGULATORY INHIBITOR SUBUNIT 16A ; ANK_REP_REGION ; coiled-coil ; Ankyrin repeat	GO:0005515_F_protein binding; GO:0005829_C_cytosol;	K06270_04510_Focal adhesion; K06270_04720_Long-term potentiation; K06270_04810_Regulation of actin cytoskeleton;	
MGC03366	1	580	Leucine-rich repeat-containing protein 6	LEUCINE RICH REPEAT FAMILY ; coiled-coil ; HSP20-like chaperones ; TESTIS SPECIFIC LEUCINE RICH REPEAT PROTEIN			
MGC03380	1	629		coiled-coil			
MGC03385	1	569	Integrin beta-1-B	EGF/Laminin ; INTEGRIN_BETA ; EGF_1 ; EGF_2 ; PA2C_AGKRH_Q9PVE9; ; INTEGRIN BETA SUBUNIT			
MGC03425	1	572	THO complex subunit 5 homolog A	FMS INTERACTING PROTEIN			
MGC03427	1	439					
MGC03433	1	475	Heat shock protein 83 (Fragment)	HEAT SHOCK PROTEIN 90 ; Ribosomal protein S5 domain 2-like ; HSP90		K04079_04612_Antigen processing and presentation; K04079_04914_Progesterone-mediated oocyte maturation; K04079_05215_Prostate cancer;	
MGC03442	1	538	Interaptin	coiled-coil			
MGC03448	1	511		Fibronectin type III ; FN3			
MGC03461	1	329	ATP-binding cassette sub-family F member 3	ATP-BINDING CASSETTE, SUB-FAMILY F (GCN20), MEMBER 3 ; P-loop containing nucleoside triphosphate hydrolases ; ATP-BINDING TRANSPORT PROTEIN-RELATED			
MGC03472	1	392					
MGC03478	5	985	Ependymin-2				
MGC03479	1	658	Multiple C2 and transmembrane domain-containing protein 1	MCTP-RELATED ; C2DOMAIN ; C2 domain (Calcium/lipid-binding domain, CaLB) ; C2	GO:0019722_P_calcium-mediated signaling; GO:0005544_F_calcium-dependent phospholipid binding; GO:0016021_C_integral to membrane; GO:0005624_C_membrane fraction; GO:0005509_F_calcium ion binding;		2.7.11.13_ProteinkinaseC.;
MGC03493	1	501	Chymotrypsinogen A	SERINE PROTEASE-RELATED ; Trypsin ; TRYPSIN_SER ; SERINE PROTEASE-RELATED, INSECT ; coiled-coil ; TRYPSIN_DOM ; Trypsin-like serine proteases		K01324_04610_Complement and coagulation cascades; K01312_04080_Neuroactive ligand-receptor interaction;	3.4.21.34_Plasmakallikrein.; 3.4.21.1_Chymotrypsin.; 3.4.21.27_CoagulationfactorXIa.; 3.4.21.4_Trypsin.;
MGC03496	1	459					
MGC03499	1	385	Basic proline-rich protein	FIBRINOGEN AND FIBRONECTIN ; Collagen ; Q8MW54_MYTGA_Q8MW54; ; FICOLIN			
MGC03521	3	1051	Uncharacterized protein ZC395.10	TELOMERASE-BINDING PROTEIN P23 (HSP90 CO-CHAPERONE) ; CS ; HSP20-like chaperones			5.3.99.3_Prostaglandin-Esynthase.;
MGC03523	5	810	Peritrophin-1	CBM_14 ; CHIT_BIND_II ; CHITIN BINDING PERITROPHIN-A ; Invertebrate chitin-binding proteins			
MGC03525	6	850	Collectin-12	C-type lectin-like ; C-TYPE LECTIN SUPERFAMILY MEMBER ; Lectin_C ; CD209 ANTIGEN (DENDRITIC CELL-SPECIFIC ICAM-3-GRABBING NONINTEGRIN 1) (DC-SIGN1) ; C_TYPE_LECTIN_2			
MGC03541	5	685		ARM repeat			
MGC03549	1	803	Ankyrin-1	ANKYRIN ; Ank ; ANKYRIN REPEAT-CONTAINING ; ANK_REPEAT ; ANK_REP_REGION ; Ankyrin repeat		K02599_04320_Dorso-ventral axis formation; K02599_04330_Notch signaling pathway;	2.4.2.30_NAD(+)ADP-ribosyltransferase.; 2.7.11.1_Non-specificserine/threonineproteinkinase.;

MGC03550	1	805	Proteasome assembly chaperone 2	TUMOR NECROSIS FACTOR SUPERFAMILY, MEMBER 5-INDUCED PROTEIN 1 (CLAST3)	GO:0043248_P_proteasome assembly; GO:0005515_F_protein binding; GO:0005634_C_nucleus;		
MGC03551	2	855	von Willebrand factor A domain-containing protein 2	EGF/Laminin ; EGF ; EGF_3 ; VWFA ; VON WILENBRAND FACTOR RELATED ; vWA-like ; EGF_1 ; VWA ; EGF_2 ; VWFADOMAIN	GO:0005578_C_proteinaceous extracellular matrix;		3.4.21.47_Alternative-complement-pathwayC3/C5convertase.; 3.4.21.22_Coagulationfactor IXa.;
MGC03554	1	753	Tripartite motif-containing protein 56	RING/U-box ; TRIM56 PROTEIN ; ZF_RING_1 ; zf-C3HC4 ; B-box zinc-binding domain ; ZF_RING_2 ; ZF_BBOX ; zf-B_box ; BBOXZNFINGER ; RING FINGER-CONTAINING PROTEIN-RELATED			
MGC03557	11	887	Baculoviral IAP repeat-containing protein 7	BIR_REPEAT_1 ; BIR ; INHIBITOR OF APOPTOSIS ; Inhibitor of apoptosis (IAP) repeat ; INHIBITOR OF APOPTOSIS PROTEIN 1 AND 2, IAP1, IAP2 ; BIR_REPEAT_2		K04725_04120_Ubiquitin mediated proteolysis; K04725_04210_Apoptosis; K04725_04510_Focal adhesion; K04725_05222_Small cell lung cancer;	
MGC03559	2	724	Macrophage migration inhibitory factor	MACROPHAGE MIGRATION INHIBITORY FACTOR RELATED ; Tautomerase/MIF ; MIF		K07253_00350_Tyrosine metabolism; K07253_00360_Phenylalanine metabolism;	5.3.2.1_Phenylpyruvatetautomerase.;
MGC03564	10	686	Macrophage mannose receptor 1				
MGC03565	8	1230	Putative per-hexamer repeat protein 5	ShK ; Fibronectin type I module ; VWFC_2 ; VWFC_1			
MGC03566	10	1212	Myeloid differentiation primary response protein MyD88	TIR ; Toll/Interleukin receptor TIR domain ; Death ; DEATH domain ; DEATH_DOMAIN ; MYD88		K04729_04210_Apoptosis; K04729_04620_Toll-like receptor signaling pathway;	
MGC03577	2	813	Complement C1q-like protein 3	C1Q-like			
MGC03583	7	772	Coactosin-like protein	CORTACTIN ; Actin depolymerizing proteins ; DREBRIN-RELATED ; Cofilin_ADF			
MGC03585	1	568	Macrophage mannose receptor 1				
MGC03589	1	801	Metalloproteinase inhibitor 2	TIMP-like ; METALLOPROTEASE INHIBITOR			
MGC03590	17	749	Aggrecan core protein	C-type lectin-like ; C_TYPE_LECTIN_1 ; C-TYPE LECTIN SUPERFAMILY MEMBER ; Lectin_C ; GALACTOSE-SPECIFIC C-TYPE LECTIN ; C_TYPE_LECTIN_2			
MGC03600	2	826	D-galactoside-specific lectin	Gal_Lectin ; SUEL_LECTIN			
MGC03602	5	1340	Gelsolin-like protein 1	Actin depolymerizing proteins ; Gelsolin ; VILLIN ; GELSOLIN ; coiled-coil		K05768_04810_Regulation of actin cytoskeleton;	
MGC03605	1	760		Lysozyme-like			
MGC03611	1	835		C1q ; TNF-like			
MGC03617	1	566	Myb-like protein X				
MGC03620	1	752	60 kDa heat shock protein, mitochondrial	GroEL apical domain-like ; GroEL equatorial domain-like ; CHAPERONIN-60KDA, CH60 ; Cpn60_TCP1 ; CHAPERONIN ; CHAPERONIN60		K04077_04940_Type I diabetes mellitus; K04077_05060_tba;	
MGC03629	1	695	Thyroglobulin	HLA CLASS II GAMMA CHAIN ; Thyroglobulin_1 ; HLA CLASS II HISTOCOMPATIBILITY ANTIGEN, GAMMA CHAIN ; Thyroglobulin type-1 domain ; THYROGLOBULIN_1_2		K10809_05320_Autoimmune thyroid disease;	
MGC03639	2	600	Complement C1q-like protein 3	C1q ; COLLAGEN ALPHA 1(VIII) CHAIN ; COLLAGEN ALPHA CHAIN ; COMPLEMNTC1Q ; TNF-like ; PROKAR_LIPOPROTEIN ; C1Q	GO:0005576_C_extracellular region;		
MGC03646	1	611					
MGC03647	1	813		PAN_1 ; PAN ; Hairpin loop containing domain-like			

MGC03656	5	670	Ankyrin repeat domain-containing protein 49	ANKYRIN ; Ank ; FETAL GLOBIN-INDUCING FACTOR ; ANKYRIN REPEAT-CONTAINING ; ANK_REPEAT ; ANK_REP_REGION ; Ankyrin repeat	GO:0005515_F_protein binding; GO:0048675_P_axon extension;	K06694_03050_Proteasome;	2.7.11.1_Non-specificserine/threonineproteinkinase.; 2.4.2.30_NAD(+)ADP-ribosyltransferase.;
MGC03657	2	758	Proteasome subunit beta type-2	N-terminal nucleophile aminohydrolases (Ntn hydrolases) ; Proteasome ; PROTEASOME_B ; PROTEASOME SUBUNIT BETA TYPE 2 ; PROTEASOME SUBUNIT ALPHA/BETA		K02734_03050_Proteasome;	3.4.25.1_Proteasomeendopeptidasecomplex.;
MGC03658	2	587	Ras-like GTP-binding protein RHO	P-loop containing nucleoside triphosphate hydrolases ; RASTRNSFRMNG ; RAS-RELATED GTPASE ; small_GTP: small GTP-binding protein domain ; Ras ; GTPASE_RHO			2.7.11.1_Non-specificserine/threonineproteinkinase.;
MGC03662	1	753					
MGC03666	7	716	Echotoxin-2	Anemone pore-forming cytolysin			
MGC03669	1	607	Ras-related protein R-Ras2	P-loop containing nucleoside triphosphate hydrolases ; RASTRNSFRMNG ; RAS-RELATED GTPASE ; small_GTP: small GTP-binding protein domain ; Ras ; GTP-BINDING PROTEIN RIT		K07830_04010_MAPK signaling pathway; K07830_04530_Tight junction; K07830_04810_Regulation of actin cytoskeleton;	
MGC03682	6	1021	Eukaryotic translation initiation factor 4E-binding protein 1	EUKARYOTIC TRANSLATION INITIATION FACTOR 4E-BINDING PROTEIN ; eIF_4EBP		K07205_04012_ErbB signaling pathway; K07205_04150_mTOR signaling pathway; K07205_04910_Insulin signaling pathway; K07205_05221_Acute myeloid leukemia;	
MGC03685	1	478		Immunoglobulin ; CELL ADHESION MOLECULE ; IMMUNOGLOBULIN DOMAIN SUPERFAMILY (SENSORY GUIDANCE PROTEIN) ; I-set			
MGC03686	1	541	Myticin-B				
MGC03689	1	773	Complement C1q tumor necrosis factor-related protein 6	C1q ; COLLAGEN ALPHA CHAIN ; COMPLEMENT C1Q AND TUMOR NECROSIS FACTOR RELATED PROTEIN 6 ; COMPLEMNTC1Q ; TNF-like ; coiled-coil ; PROKAR_LIPOPROTEIN ; C1Q		K03987_04610_Complement and coagulation cascades; K03987_05010_Alzheimers disease;	
MGC03691	1	789	Kelch-like protein 28	POZ domain			
MGC03693	9	816	Adipocyte enhancer-binding protein 1	LACTADHERIN/MFG-E8 ; DISCOIDIN, CUB, EGF, LAMININ , AND ZINC METALLOPROTEASE DOMAIN ; FA58C_3 ; Galactose-binding domain-like ; F5_F8_type_C		K03902_04610_Complement and coagulation cascades;	
MGC03696	4	702	Macrophage asialoglycoprotein-binding protein 1	C-type lectin-like ; ASIALOGLYCOPROTEIN RECEPTOR ; C-TYPE LECTIN SUPERFAMILY MEMBER ; Lectin_C ; C_TYPE_LECTIN_2	GO:0009792_P_embryonic development ending in birth or egg hatching;		
MGC03698	1	712	Thyroglobulin	INSULIN-LIKE GROWTH FACTOR BINDING PROTEIN ; Thyroglobulin_1 ; Thyroglobulin type-1 domain ; INSULIN-LIKE GROWTH FACTOR BINDING PROTEIN 3 ; THYROGLOBULIN_1_2		K10809_05320_Autoimmune thyroid disease;	
MGC03702	1	293	Collagen-like protein 7	COLLAGEN ALPHA CHAIN ; Collagen ; COLLAGEN ALPHA 2(IV) CHAIN, INSECT			
MGC03707	15	1094	Mucin-2	Invertebrate chitin-binding proteins			
MGC03709	1	718		C_TYPE_LECTIN_1			
MGC03710	2	760		C1q ; GLIACOLIN-RELATED ; CEREBELLIN-RELATED ; TNF-like			
MGC03713	3	821	Structural maintenance of chromosomes protein 4	coiled-coil			

MGC03716	1	679	Inositol monophosphatase 1	INOSITOL MONOPHOSPHATASE ; Carbohydrate phosphatase ; MYO INOSITOL MONOPHOSPHATASE ; MYOP_MOUSE_O55023; ; Inositol_P ; IMP_1 ; INOSPHPHTASE	GO:0006020_P_inositol metabolic process;	K01092_00521_Streptomycin biosynthesis; K01092_00562_Inositol phosphate metabolism; K01092_04070_Phosphatidylinositol signaling system;	3.1.3.25_Inositol-phosphatephosphatase.;
MGC03719	1	768	Probable ATP-dependent RNA helicase DDX10	ATP-DEPENDENT RNA HELICASE DDX10 (DEAD-BOX PROTEIN 10) ; DEAD BOX ATP-DEPENDENT RNA HELICASE ; coiled-coil	GO:0003724_F_RNA helicase activity;	K01529_00500_Starch and sucrose metabolism; K01529_00790_Folate biosynthesis;	
MGC03726	9	759	Mammalian ependymin-related protein 1				
MGC03727	3	761		E set domains			
MGC03735	11	777	Proteasome subunit beta type-6	N-terminal nucleophile aminohydrolases (Ntn hydrolases) ; Proteasome ; PROTEASOME_B ; PROTEASOME ; PROTEASOME SUBUNIT BETA TYPE 6,9 ; PROTEASOME SUBUNIT ALPHA/BETA		K02738_03050_Proteasome;	3.4.25.1_Proteasomeendopeptidasecomplex.;
MGC03736	13	1019	Periostin	FAS1 ; PERIOSTIN-RELATED ; PERIOSTIN (PN) (OSTEOBLAST-SPECIFIC FACTOR 2) (OSF-2) ; Fasciclin ; FAS1 domain	GO:0008201_F_heparin binding; GO:0007155_P_cell adhesion; GO:0030198_P_extracellular matrix organization and biogenesis; GO:0009888_P_tissue development; GO:0005578_C_proteinaceous extracellular matrix; GO:0001501_P_skeletal development;		
MGC03741	8	710		PTN_MK_C ; Midkine ; SUBcoiled-coil			
MGC03745	6	743	Complement C1q-like protein 2	C1q ; CEREBELLIN-RELATED ; TNF-like ; coiled-coil ; C1Q			
MGC03747	1	767	Apoptosis regulator Bcl-2	BCL2_FAMILY ; Bcl-2 inhibitors of programmed cell death ; Bcl-2 ; BCL-2 RELATED			
MGC03753	1	698	Fucolectin-6	Galactose-binding domain-like ; F5_F8_type_C			
MGC03756	2	932	Purine nucleoside phosphorylase	Purine-nucleoside phosphorylase ; PURINE NUCLEOSIDE PHOSPHORYLASE ; METHYLTHIOADENOSINE/PURINE NUCLEOSIDE PHOSPHORYLASE ; PNPH: purine nucleoside phosphorylase ; Mtap_PNP ; Purine and uridine phosphorylases ; PNPH-PUNA-XAPA: inosine guanosine and		K03815_00230_Purine metabolism; K03783_00230_Purine metabolism; K03783_00240_Pyrimidine metabolism; K03783_00760_Nicotinate and nicotinamide metabolism;	2.4.2.1_Purine-nucleosidephosphorylase.;
MGC03762	2	610	Perlucin	C-type lectin-like ; C_TYPE_LECTIN_1 ; C-TYPE LECTIN SUPERFAMILY MEMBER ; Lectin_C ; GALACTOSE-SPECIFIC C-TYPE LECTIN ; EGF_2 ; C_TYPE_LECTIN_2			
MGC03765	4	732	Calmodulin	EF_HAND_1 ; CALMODULIN ; EF_HAND_2 ; EF-hand ; CALM_EPIAK_Q7T3T2; ; CALCIUM BINDING PROTEIN ; efhand			2.7.11.1_Non-specificserine/threonineproteinkinase.;
MGC03770	3	668	Calmodulin	EF_HAND_1 ; CALMODULIN ; EF_HAND_2 ; Q76LB7_STRIE_Q76LB7; ; EF-hand ; CALCIUM BINDING PROTEIN ; efhand			2.7.11.1_Non-specificserine/threonineproteinkinase.;
MGC03774	6	648	Neurocan core protein	C-type lectin-like ; C-TYPE LECTIN SUPERFAMILY MEMBER ; Lectin_C ; GALACTOSE-SPECIFIC C-TYPE LECTIN ; C_TYPE_LECTIN_2			

MGC03780	1	689	WD repeat-containing protein 61	WD_REPEATS_2 ; Q8BVQ0_MOUSE_Q8BVQ0; ; GPROTEINBRPT ; SUBWD40 repeat-like ; WD_REPEATS_REGION ; WD40	GO:0005515_F_protein binding;	K01062_00565_Ether lipid metabolism;	2.7.11.1_Non-specificserine/threonineproteinkinase.; 2.4.1.37_Fucosylgalactoside3-alpha-galactosyltransferase.; 2.7.11.7_[Myosinheavy-chain]kinase.;
MGC03785	2	800	Uncharacterized protein ywbO	DSBA ; Thioredoxin-like			
MGC03787	9	754	Lysozyme 3	DESTABILASE-RELATED ; Destabilase ; Lysozyme-like	GO:0003796_F_lysozyme activity; GO:0050829_P_defense response to Gram-negative bacterium; GO:0005576_C_extracellular region; GO:0050830_P_defense response to Gram-positive bacterium;		3.2.1.17_Lysozyme.;
MGC03790	1	810	Ras-related protein Rab-32B	P-loop containing nucleoside triphosphate hydrolases ; RAS-RELATED PROTEIN RAB-32 AND 38 ; RASTRNSFRMNG ; RAS-RELATED GTPASE ; small_GTP: small GTP-binding protein domain ; Ras			
MGC03791	5	834	Calcyclin-binding protein	CALICYLIN BINDING PROTEIN ; CS ; SGS ; coiled-coil ; Siah-Interact_N ; HSP20-like chaperones	GO:0005515_F_protein binding;	K04507_04310_Wnt signaling pathway;	
MGC03794	1	768	Growth arrest and DNA damage-inducible protein GADD45 alpha				
MGC03799	2	753	Caprin-2	C1q ; GLIACOLIN-RELATED ; CEREBELLIN-RELATED ; COMPLEMNTC1Q ; TNF-like ; C1Q	GO:0005737_C_cytoplasm; GO:0005739_C_mitochondrion; GO:0030308_P_negative regulation of cell growth;		
MGC03800	3	640		TNF-like			
MGC03812	2	896	Cerebellin-2				
MGC03819	3	624	Calmodulin	EF_HAND_1 ; CALMODULIN ; EF_HAND_2 ; CALFLAGIN ; CALM_CHLRE_P04352; ; EF-hand ; coiled-coil ; CALCIUM BINDING PROTEIN ; efhand			2.7.11.1_Non-specificserine/threonineproteinkinase.;
MGC03821	1	780	Uncharacterized protein PFB0145c	coiled-coil			
MGC03823	4	561		EF_HAND_1 ; EF_HAND_2 ; EF-hand ; efhand			
MGC03825	1	796	NADH dehydrogenase [ubiquinone] iron-sulfur protein 3, mitochondrial	COMPLEX1_30K ; NUGM_HUMAN_O75489; ; Complex1_30kDa ; NADH-UBIQUINONE OXIDOREDUCTASE-RELATED ; NuoC_fam: NADH (or F420H2) dehydrogenase, su ; NADH-UBIQUINONE OXIDOREDUCTASE MITOCHONDRIAL PRECURSOR		K03936_00190_Oxidative phosphorylation;	1.6.99.3_NADHdehydrogenase.; 1.6.5.3_NADHdehydrogenase(ubiquinone).;
MGC03826	1	755	Ficolin-1	FIBRINOGEN AND FIBRONECTIN ; Fibrinogen C-terminal domain-like ; Fibrinogen_C		K08767_03320_PPAR signaling pathway;	
MGC03836	2	645	Cerebellin-1	C1q ; CEREBELLIN-RELATED ; COMPLEMNTC1Q ; TNF-like ; C1Q	GO:0007399_P_nervous system development; GO:0007268_P_synaptic transmission;		
MGC03838	13	822	Metalloproteinase inhibitor 3				
MGC03844	3	1226	mRNA-capping enzyme	mRNA_cap_C ; MRNA CAPPING ENZYME ; mRNA_cap_enzyme ; Nucleic acid-binding proteins ; DNA ligase/mRNA capping enzyme, catalytic domain			2.7.7.50_mRNAguanylyltransferase.; 3.1.3.33_Polynucleotide5{prime}-phosphatase.;
MGC03848	3	1091	Collagen alpha-1(XII) chain	ShK ; VWFA ; INTEGRIN ALPHA-RELATED ; VON WILENBRAND FACTOR RELATED ; vWA-like ; VWA ; VWFADOMAIN			

MGC03852	3	1038	Tubulin beta-1 chain	Tubulin_C ; Tubulin ; BETATUBULIN ; TUBULIN BETA CHAIN ; Tubulin nucleotide-binding domain-like ; Tubulin C-terminal domain-like ; TUBULIN	GO:0045298_C_tubulin complex; GO:0005874_C_microtubule; GO:0016203_P_muscle attachment; GO:0005525_F_GTP binding; GO:0007017_P_microtubule-based process; GO:0005200_F_structural constituent of cytoskeleton;	K07375_04540_Gap junction;	
MGC03857	4	1160					
MGC03859	1	504	Cyclic AMP-dependent transcription factor ATF-3	TRANSCRIPTION FACTOR HY5-LIKE (HY5 HOMOLOG) ; bZIP_2 ; A DNA-binding domain in eukaryotic transcription factors ; BZIP ; coiled-coil ; X-BOX TRANSCRIPTION FACTOR-RELATED			
MGC03860	8	902	Complement C1q-like protein 4	C1q ; GLIACOLIN-RELATED ; CEREBELLIN-RELATED ; COMPLEMNTC1Q ; TNF-like ; coiled-coil ; C1Q			
MGC03869	4	744	Lysozyme g	SLT ; LYSOZYMEG ; Lysozyme g ; Lysozyme-like			3.2.1.17_Lysozyme.;
MGC03870	1	593	Myosin-2 heavy chain	Spectrin repeat ; STRUCTURAL MAINTENANCE OF CHROMOSOMES SMC FAMILY MEMBER ; tRNA-binding arm ; coiled-coil			
MGC03878	4	724	Putative tyrosinase-like protein tyr-3	EGF/Laminin ; ShK			
MGC03879	3	1427	Tribbles homolog 1	Protein kinase-like (PK-like) ; Pkinase ; SER/THR PROTEIN KINASE-TRB1 ; SER/THR PROTEIN KINASE-TRB ; Q76D11_XENLA_Q76D11; ; PROTEIN_KINASE_DOM			2.7.11.1_Non-specificserine/threonineproteinkinase.;
MGC03882	12	778	Metalloproteinase inhibitor 3				
MGC03887	1	601	Low-density lipoprotein receptor-related protein 6	LOW DENSITY LIPOPROTEIN RECEPTOR ; LDLRB ; YWTD domain ; Ldl_recept_b			
MGC03894	4	833	Anamorsin homolog	SUBS-adenosyl-L-methionine-dependent methyltransferases ; DUF689	GO:0000003_P_reproduction; GO:0005737_C_cytoplasm; GO:0005515_F_protein binding; GO:0040010_P_positive regulation of growth rate; GO:0009792_P_embryonic development ending in birth or egg hatching; GO:0030097_P_hemopoiesis; GO:0006916_P_anti-apoptosis;		
MGC03898	3	985					
MGC03900	4	804	Muscle LIM protein Mlp84B	CRP1/CSRP1/CRIP1 ; LIM ; LIM DOMAIN CONTAINING PROTEIN ; LIM_DOMAIN_2 ; Glucocorticoid receptor-like (DNA-binding domain) ; LIM_DOMAIN_1 ; Q6SA71_BOMMO_Q6SA71; coiled-coil			
MGC03904	1	505					
MGC03908	1	284	Peptidyl-prolyl cis-trans isomerase-like 3	Cyclophilin-like ; CSAPPISMRASE ; CYCLOPHILIN-10 ; CYCLOPHILIN ; Pro_isomerase ; CSA_PPIASE_2			5.2.1.8_Peptidylprolylisomerase.;
MGC03915	2	654	Platelet endothelial aggregation receptor 1	EGF-LIKE DOMAIN PROTEIN ; omega toxin-like ; 4DISULPHCORE ; EGF_1 ; EGF_2 ; MULTIPLE EGF-LIKE-DOMAIN PROTEIN	GO:0005515_F_protein binding; GO:0040010_P_positive regulation of growth rate;		
MGC03917	3	654	Neurocan core protein	C-type lectin-like ; ASIALOGLYCOPROTEIN RECEPTOR ; C_TYPE_LECTIN_1 ; C-TYPE LECTIN SUPERFAMILY MEMBER ; Lectin_C ; ANTIFREEZEII ; C_TYPE_LECTIN_2			

MGC03918	2	489	Heavy metal-binding protein HIP	C1q ; CEREBELLIN-RELATED ; COMPLEMNTC1Q ; TNF-like ; C1Q		K03986_04610_Complement and coagulation cascades; K03986_05010_Alzheimers disease;	
MGC03930	1	732	Serine protease inhibitor dipetalogastin (Fragment)	Kazal_2 ; TESTICAN 3 ; KAZAL ; TESTICAN ; Kazal-type serine protease inhibitors ; Q6H3D0_TRIST_Q6H3D0;		K06254_04512_ECM-receptor interaction;	
MGC03934	2	1422	NF-kappa-B inhibitor alpha	ANKYRIN ; Ank ; ANKYRIN REPEAT-CONTAINING ; DEVELOPMENTAL PROTEIN CACTUS ; ANK_REPEAT ; ANK_REP_REGION ; Ankyrin repeat			2.7.11.1_Non-specificserine/threonineproteinkinase.;
MGC03943	7	958	Neurogenic locus notch homolog protein 2	CBM_14 ; CHIT_BIND_II ; Invertebrate chitin-binding proteins			
MGC03947	1	703	Lysozyme 1	N-acetylmuramoyl-L-alanine amidase-like	GO:0003796_F_lysozyme activity; GO:0050829_P_defense response to Gram-negative bacterium; GO:0005576_C_extracellular region; GO:0050830_P_defense response to Gram-positive bacterium;		3.2.1.17_Lysozyme.;
MGC03949	1	641	Guanine nucleotide-binding protein G(I)/G(S)/G(O) subunit gamma-12	GUANINE NUCLEOTIDE-BINDING PROTEIN GAMMA SUBUNIT ; G-gamma ; Transducin (heterotrimeric G protein), gamma chain ; G_PROTEIN_GAMMA ; GPROTEING	GO:0007165_P_signal transduction;	K04347_04010_MAPK signaling pathway; K04347_04810_Regulation of actin cytoskeleton;	
MGC03951	4	772	Sarcoplasmic calcium-binding protein	EF_HAND_2 ; EF-hand ; efhand			
MGC03952	1	656	Protein toll	TIR ; Toll/Interleukin receptor TIR domain ; TOLL ; INTRLKN1R1F ; LEUCINE-RICH TRANSMEMBRANE PROTEINS		K10160_04620_Toll-like receptor signaling pathway; K10159_04620_Toll-like receptor signaling pathway;	
MGC03954	4	1390	Apolipophorins	MUCIN ; Lipovitellin-phosvitin complex; beta-sheet shell regions ; DUF1943			
MGC03960	7	737	Chymotrypsin-like elastase family member 1	TRYPSIN_HIS ; SERINE PROTEASE-RELATED ; Trypsin ; TRYPSIN_SER ; CHYMOTRYPSIN ; SERINE PROTEASE-RELATED, INSECT ; TRYPSIN_DOM ; Trypsin-like serine proteases			3.4.21.36_Pancreaticelastase.; 3.4.21.1_Chymotrypsin.;
MGC03967	1	538	Serine/threonine-protein phosphatase 6 regulatory ankyrin repeat subunit C	ANKYRIN ; Ank ; ANKYRIN REPEAT-CONTAINING ; ANK_REPEAT ; ANK_REP_REGION ; TANKYRASE ; Ankyrin repeat			2.7.11.1_Non-specificserine/threonineproteinkinase.; 2.4.2.30_NAD(+)ADP-ribosyltransferase.; 2.1.1.43_Histone-lysineN-methyltransferase.;
MGC03972	2	1206	Gastric intrinsic factor	TRANSCOBALAMIN ; Terpenoid cyclases/Protein prenyltransferases			
MGC03975	1	785	Putative L-amino-acid oxidase yobN	AMINE OXIDASE ; Amino_oxidase ; FAD-linked reductases, C-terminal domain ; BACILLUS AMINE OXIDASE ; FAD/NAD(P)-binding domain			1.4.3.2_L-amino-acidoxidase.;
MGC03979	2	863	Trichohyalin	coiled-coil			
MGC03982	1	505	Perlucin	C-type lectin-like ; C_TYPE_LECTIN_1 ; C-TYPE LECTIN SUPERFAMILY MEMBER ; Lectin_C ; GALACTOSE-SPECIFIC C-TYPE LECTIN ; ANTIFREEZEII ; C_TYPE_LECTIN_2			
MGC03983	5	1102	Peptidyl-prolyl cis-trans isomerase B	Cyclophilin-like ; CSAPPISMRASE ; CYCLOPHILIN ; Pro_isomerase ; CSA_PPIASE_2			5.2.1.8_Peptidylprolylisomerase.;

MGC03984	3	1094	Collagen alpha-1(XII) chain	VWFA ; VON WILENBRAND FACTOR RELATED ; vWA-like ; VWA ; VWFADOMAIN	GO:0030199_P_collagen fibril organization; GO:0030020_F_extracellular matrix structural constituent conferring tensile strength; GO:0005595_C_collagen type XII; GO:0005578_C_proteinaceous extracellular matrix; GO:0001501_P_skeletal development;	K06462_04810_Regulation of actin cytoskeleton; K06524_04810_Regulation of actin cytoskeleton; K06594_04810_Regulation of actin cytoskeleton;	
MGC03985	1	773					
MGC03990	2	882	Uncharacterized protein ZK643.6	ShK			
MGC03994	4	802	Transforming growth factor-beta-induced protein ig-h3	FAS1 ; Fasciclin ; FAS1 domain			
MGC03996	1	746	Tctex1 domain-containing protein 4	AXONEMAL DYNEIN LIGHT CHAIN ; Tctex-1 ; T-COMPLEX-ASSOCIATED-TESTIS-EXPRESSED 1/ DYNEIN LIGHT CHAIN	GO:0016020_C_membrane; GO:0005515_F_protein binding; GO:0009434_C_microtubule-based flagellum; GO:0005868_C_cytoplasmic dynein complex; GO:0007018_P_microtubule-based movement;		
MGC03997	2	663	Disabled homolog 2-interacting protein	PH ; RAS GTPASE ACTIVATING PROTEIN-RELATED ; RAS GTPASE-ACTIVATING PROTEINS ; PH_DOMAIN ; PH domain-like		K04352_04010_MAPK signaling pathway; K04352_04360_Axon guidance; K04352_05040_tba;	
MGC04007	1	763	Heat shock protein 83	HEAT SHOCK PROTEIN 90 ; Ribosomal protein S5 domain 2-like ; HSP90 ; coiled-coil	GO:0030235_F_nitric-oxide synthase regulator activity; GO:0005515_F_protein binding; GO:0030911_F_TPR domain binding; GO:0045429_P_positive regulation of nitric oxide biosynthetic process;	K04079_04612_Antigen processing and presentation; K04079_04914_Progesterone-mediated oocyte maturation; K04079_05215_Prostate cancer;	
MGC04010	1	719					
MGC04013	1	714	C1q-related factor	C1q ; GPI-ANCHORED PROTEIN P137 ; COMPLEMNTC1Q ; TNF-like ; coiled-coil ; C1Q		K03986_04610_Complement and coagulation cascades; K03986_05010_Alzheimers disease;	
MGC04021	4	910	Aggrecan core protein	EGF/Laminin ; C-type lectin-like ; EGF ; EGF_3 ; Lectin_C ; AGGRECAN/VERSICAN PROTEOGLYCAN ; EGF_1 ; EGF_2 ; coiled-coil ; ANTIFREEZEII ; C_TYPE_LECTIN_2			
MGC04022	8	738	Proteasome subunit alpha type-6	N-terminal nucleophile aminohydrolases (Ntn hydrolases) ; Proteasome ; PROTEASOME SUBUNIT ALPHA TYPE 6 ; PROTEASOME SUBUNIT ALPHA/BETA ; PROTEASOME_A	GO:0005515_F_protein binding; GO:0003723_F_RNA binding; GO:0006511_P_ubiquitin-dependent protein catabolic process; GO:0004175_F_endopeptidase activity; GO:0005839_C_proteasome core complex;	K02730_03050_Proteasome;	3.4.25.1_Proteasomeendopeptidasecomplex.;
MGC04026	2	638		TNF-like			
MGC04028	1	761		Snake toxin-like			
MGC04032	4	705	Collagen alpha-1(XII) chain	VWFA ; VON WILENBRAND FACTOR RELATED ; vWA-like ; VWA ; VWFADOMAIN	GO:0030199_P_collagen fibril organization; GO:0030020_F_extracellular matrix structural constituent conferring tensile strength; GO:0005595_C_collagen type XII; GO:0001501_P_skeletal development;	K06238_04510_Focal adhesion; K06238_04512_ECM-receptor interaction;	3.4.21.43_Classical-complement-pathwayC3/C5convertase.;
MGC04036	2	653	Oxidation resistance protein 1	NUCLEOLAR PROTEIN 7/ESTROGEN RECEPTOR COACTIVATOR-RELATED ; LysM ; LysM domain ; NUCLEOLAR PROTEIN C7B	GO:0005730_C_nucleolus;		

MGC04037	2	762	Complement C1q tumor necrosis factor-related protein 2	C1q ; COLLAGEN ALPHA CHAIN ; COMPLEMNTC1Q ; TNF-like ; coiled-coil ; C1Q	GO:0005515_F_protein binding; GO:0007155_P_cell adhesion;	K03987_04610_Complement and coagulation cascades; K03987_05010_Alzheimers disease;	
MGC04039	1	672	CCAAT/enhancer-binding protein epsilon	CCAAT/ENHANCER BINDING PROTEIN ; bZIP_2 ; BZIP ; coiled-coil			
MGC04043	1	524	D-galactoside-specific lectin	Gal_Lectin ; SUEL_LECTIN			
MGC04044	3	852					
MGC04046	1	741					
MGC04049	5	741	Metalloproteinase inhibitor 3	TIMP-like ; METALLOPROTEASE INHIBITOR			
MGC04056	9	813	Putative tyrosinase-like protein tyr-3	ShK			
MGC04063	3	962	Yolk ferritin	FERRITIN_LIKE ; FERRITIN ; Ferritin ; Ferritin-like ; FRIY_LYMST_P42578;		K00522_00860_Porphyrin and chlorophyll metabolism;	1.16.3.1_Ferroxidase.;
MGC04064	3	775	Eukaryotic translation initiation factor 3 subunit M	COP9 SIGNALOSOME COMPLEX SUBUNIT 7/DENDRITIC CELL PROTEIN GA17 ; DENDRITIC CELL PROTEIN GA17	GO:0005515_F_protein binding;		
MGC04067	13	1352	Alcohol dehydrogenase class-3 chain H	ALCOHOL DEHYDROGENASE ; ADH_N ; ALCOHOL DEHYDROGENASE RELATED ; NAD(P)-binding Rossmann-fold domains ; ADH_ZINC ; adh_III_F_hyde: S-(hydroxymethyl)gluta ; TONB_DEPENDENT_REC_1 ; ADH_zinc_N ; GroES-like		K00121_00680_Methane metabolism;	1.1.1.1_Alcoholdehydrogenase.; 1.1.1.284_S-(hydroxymethyl)glutathionedehydrogenase.;
MGC04069	1	600	Ras-related C3 botulinum toxin substrate 2	P-loop containing nucleoside triphosphate hydrolases ; RASTRNSFRMNG ; RAS-RELATED PROTEIN RAC ; RAS-RELATED GTPASE ; small_GTP: small GTP-binding protein domain ; Ras			
MGC04073	2	662	Transcription factor MafB				
MGC04074	2	776	Low-density lipoprotein receptor-related protein 5				
MGC04076	1	769					
MGC04079	2	826	Gamma-secretase subunit Aph-1	Aph-1 ; GAMMA-SECRETASE SUBUNIT APH-1	GO:0005887_C_integral to plasma membrane; GO:0007219_P_Notch signaling pathway; GO:0004175_F_endopeptidase activity;	K06172_04330_Notch signaling pathway; K06172_05010_Alzheimers disease;	
MGC04082	8	1363	Neurogenic locus notch homolog protein 3	EGF/Laminin ; EGF-LIKE DOMAIN PROTEIN ; EGF ; EGF_3 ; ASX_HYDROXYL ; CRUMBS(D.MELANOGASTER) RELATED ; EGF_1 ; EGF_2		K02599_04320_Dorso-ventral axis formation; K02599_04330_Notch signaling pathway;	3.4.21.22_Coagulationfactor IXa.;
MGC04088	1	589	SH3 domain-binding glutamic acid-rich-like protein 3	SH3 DOMAIN-BINDING GLUTAMIC ACID-RICH-LIKE PROTEIN ; Thioredoxin-like ; SH3BGR			
MGC04100	1	707	97 kDa heat shock protein	HEAT SHOCK PROTEIN 70 (HSP70)-4 ; HEAT SHOCK PROTEIN 70KDA ; Heat shock protein 70kD (HSP70), C-terminal subdomain ; HSP70 ; coiled-coil			
MGC04102	5	629	C-type lectin domain family 4 member E	C-type lectin-like ; C_TYPE_LECTIN_1 ; C-TYPE LECTIN SUPERFAMILY MEMBER ; Lectin_C ; GALACTOSE-SPECIFIC C-TYPE LECTIN ; coiled-coil ; C_TYPE_LECTIN_2			
MGC04105	15	1148	Beta-1,3-glucan-binding protein 1	Concanavalin A-like lectins/glucanases ; GRAM-NEGATIVE BACTERIA BINDING PROTEIN 1 ; SECRETED GLUCOSIDASE-RELATED ; Glyco_hydro_16		K01238_00530_Aminosugars metabolism;	3.2.1.73_Licheninase.; 3.2.1.39_Glucanendo-1,3-beta-D-glucosidase.;
MGC04107	1	762	Thyroglobulin	INSULIN-LIKE GROWTH FACTOR BINDING PROTEIN ; Thyroglobulin_1 ; Thyroglobulin type-1 domain ; INSULIN-LIKE GROWTH FACTOR BINDING PROTEIN 3 ; THYROGLOBULIN_1_2		K10809_05320_Autoimmune thyroid disease;	

MGC04110	6	1216	Superoxide dismutase [Cu-Zn]	CU/ZN SUPEROXIDE DISMUTASE ; Sod_Cu ; CUZNDISMTASE ; SUPEROXIDE DISMUTASE [CU-ZN] ; SOD_CU_ZN_1 ; Q7YXM6_APILI_Q7YXM6; ; Cu,Zn superoxide dismutase-like	GO:0006979_P_response to oxidative stress; GO:0004784_F_superoxide dismutase activity; GO:0005829_C_cytosol; GO:0005634_C_nucleus;	K04565_05030_tba;	1.15.1.1_Superoxidedismutase.;
MGC04114	4	767	Probable splicing factor, arginine/serine-rich 7	coiled-coil			
MGC04118	1	825	Electroneutral sodium bicarbonate exchanger 1	ANION EXCHANGE PROTEIN ; SODIUM BICARBONATE COTRANSPORTER (NBC2, 3) ; HCO3_cotransp	GO:0006821_P_chloride transport; GO:0015701_P_bicarbonate transport; GO:0016021_C_integral to membrane;		
MGC04119	10	1355	Rootletin	CENTROSOMAL PROTEIN 2 ; coiled-coil ; CILIARY ROOTLET COILED-COIL, ROOTLETIN	GO:0005198_F_structural molecule activity; GO:0051297_P_centrosome organization and biogenesis; GO:0035253_C_ciliary rootlet; GO:0019894_F_kinesin binding; GO:0005814_C_centriole;		
MGC04120	1	607	Dedicator of cytokinesis protein 11	DOCK-9, 10, 11 ; DEDICATOR OF CYTOKINESIS (DOCK)	GO:0005089_F_Rho guanyl-nucleotide exchange factor activity; GO:0017048_F_Rho GTPase binding;		
MGC04122	9	657	Ganglioside GM2 activator	Ganglioside M2 (gm2) activator			
MGC04123	1	734	Ubiquitin-associated and SH3 domain-containing protein B	LigT-like ; UBA-like ; SUBUBA	GO:0005515_F_protein binding;		3.1.2.15_Ubiquitinthiolesterase.;
MGC04124	3	794	Hemicentin-1	Immunoglobulin ; IG_LIKE ; I-set ; LCCL domain ; ig ; LCCL ; NEUROTRACTING/LSAMP/NEUROTRIMIN/OBCAM RELATED CELL ADHESION MOLECULE	GO:0005604_C_basement membrane; GO:0008104_P_protein localization; GO:0005576_C_extracellular region; GO:0005938_C_cell cortex; GO:0030054_C_cell junction; GO:0005515_F_protein binding; GO:0005578_C_proteinaceous extracellular matrix;	K06550_04360_Axon guidance; K06550_04514_Cell adhesion molecules (CAMs); K06255_04512_ECM-receptor interaction;	2.7.11.1_Non-specificserine/threonineproteinkinase.; 1.11.1.7_Peroxidase.;
MGC04133	1	828	Repetitive proline-rich cell wall protein 2				
MGC04135	3	1162	Src substrate cortactin	CORTACTIN ; HS1_rep ; SRC SUBSTRATE CORTACTIN	GO:0005737_C_cytoplasm; GO:0030027_C_lamellipodium; GO:0005938_C_cell cortex; GO:0005515_F_protein binding; GO:0006898_P_receptor-mediated endocytosis;	K06106_04530_Tight junction;	
MGC04136	6	1033	Collagen alpha-1(XII) chain	VWFA ; VON WILENBRAND FACTOR RELATED ; TSP-1 type 1 repeat ; vWA-like ; VWA ; VWFADOMAIN	GO:0030199_P_collagen fibril organization; GO:0030020_F_extracellular matrix structural constituent conferring tensile strength; GO:0001501_P_skeletal development; GO:0005595_C_collagen type XII;		
MGC04137	1	811	Thyrotropin-releasing hormone-degrading ectoenzyme	PROTEASE M1 ZINC METALLOPROTEASE ; AMINOPEPTIDASE N-RELATED ; Metalloproteases ('zincins'), catalytic domain ; Peptidase_M1			3.4.11.2_Membranealanylaminopeptidase.; 3.4.19.6_Pyroglutamyl-peptidaseII.;
MGC04139	1	806					

MGC04143	10	921	Neurogenic locus Notch protein	EGF/Laminin ; EGF-LIKE DOMAIN PROTEIN ; EGF ; EGF_3 ; ASX_HYDROXYL ; Cadherin-like ; FA58C_3 ; CRUMBS(D.MELANOGASTER) RELATED ; EGF_1 ; Galactose-binding domain-like ; He_PIG ; EGF_CA ; F5_F8_type_C	GO:0001957_P_intramembranous ossification; GO:0001501_P_skeletal development;	K02599_04320_Dorso-ventral axis formation; K02599_04330_Notch signaling pathway;	
MGC04146	2	680	Calmodulin	EF_HAND_1 ; CALMODULIN ; EF_HAND_2 ; Q76LB7_STRIE_Q76LB7; ; EF-hand ; CALCIUM BINDING PROTEIN ; efhand			2.7.11.1_Non-specificserine/threonineproteinkinase.;
MGC04151	1	750	Zinc metalloproteinase dpy-31	ZINC METALLOPROTEINASE NAS-RELATED ; DISCOIDIN, CUB, EGF, LAMININ , AND ZINC METALLOPROTEASE DOMAIN ; Metalloproteases ('zincins'), catalytic domain ; Astacin ; ASTACIN	GO:0009792_P_embryonic development ending in birth or egg hatching;		3.4.24.21_Astacin.;
MGC04157	2	701	Ectonucleoside triphosphate diphosphohydrolase 5	ADENOSINE/GUANOSINE DIPHOSPHATASE ; GDA1_CD39 ; ADENOSINE DIPHOSPHATASE		K01511_00230_Purine metabolism; K01511_00240_Pyrimidine metabolism;	3.6.1.5_Apyrase.; 3.6.1.42_Guanosine-diphosphatase.; 3.6.1.6_Nucleoside-diphosphatase.;
MGC04159	3	1072	Endoplasmin	HSP90 C-terminal domain (C-terminal part of Pfam 00183) ; HEAT SHOCK PROTEIN 90 ; Ribosomal protein S5 domain 2-like ; ER_TARGET ; HSP90 ; ENDOPLASMIN		K09487_05215_Prostate cancer;	
MGC04160	6	1258	Glutathione S-transferase P	GST_C ; GST_N ; GSTRNSFRASEP ; Glutathione S-transferase (GST), C-terminal domain ; Thioredoxin-like ; GLUTATHIONE S-TRANSFERASE ; GLUTATHIONE S-TRANSFERASE CLASS PI		K00799_00480_Glutathione metabolism; K00799_00980_Metabolism of xenobiotics by cytochrome P450;	2.5.1.18_Glutathionetransferase.;
MGC04164	4	693		C1q ; GLIACOLIN-RELATED ; CEREBELLIN-RELATED ; TNF-like ; coiled-coil ; C1Q			
MGC04165	3	684		C1q ; GLIACOLIN-RELATED ; CEREBELLIN-RELATED ; TNF-like		K03987_04610_Complement and coagulation cascades; K03987_05010_Alzheimers disease;	
MGC04167	9	940	Macrophage mannose receptor 1	C-type lectin-like ; C-TYPE LECTIN SUPERFAMILY MEMBER ; Lectin_C ; GALACTOSE-SPECIFIC C-TYPE LECTIN ; C_TYPE_LECTIN_2			
MGC04169	17	869	Trypsin alpha	TRYPSIN_HIS ; SERINE PROTEASE-RELATED ; Trypsin ; TRYPSIN_SER ; CHYMOTRYPSIN ; SERINE PROTEASE-RELATED, INSECT ; TRYPSIN_DOM ; Trypsin-like serine proteases		K01312_04080_Neuroactive ligand-receptor interaction;	3.4.21.4_Trypsin.;
MGC04170	4	1000	Collagen alpha-1(XXII) chain	VWFA ; VON WILENBRAND FACTOR RELATED ; vWA-like ; VWA ; VWFADOMAIN	GO:0030199_P_collagen fibril organization; GO:0030020_F_extracellular matrix structural constituent conferring tensile strength; GO:0001501_P_skeletal development; GO:0005595_C_collagen type XII;		
MGC04172	5	838	Proteasome inhibitor PI31 subunit	Homodimeric domain of signal transducing histidine kinase ; PROTEASOME INHIBITOR ; PI31_Prot_Reg	GO:0006511_P_ubiquitin-dependent protein catabolic process; GO:0005515_F_protein binding; GO:0005839_C_proteasome core complex; GO:0008539_F_proteasome inhibitor activity;	K06700_03050_Proteasome;	
MGC04173	1	848	Fibropellin-1	EGF/Laminin ; FN1_1 ; EGF-LIKE DOMAIN PROTEIN ; EGF ; EGF_3 ; ASX_HYDROXYL ; CRUMBS(D.MELANOGASTER) RELATED ; EGF_1 ; EGF_2 ; EGFBLOOD		K02599_04320_Dorso-ventral axis formation; K02599_04330_Notch signaling pathway;	2.7.10.1_Receptorprotein-tyrosinekinase.;

MGC04174	2	813	Receptor-interacting serine/threonine-protein kinase 2	Protein kinase-like (PK-like) ; PROTEIN_KINASE_ST ; PROTEIN_KINASE_ATP ; Pkinase ; RECEPTOR-INTERACTING SERINE-THREONINE KINASE 2, 3 ; Q8N2G9_HUMAN_Q8N2G9; ; SERINE-THREONINE PROTEIN KINASE ; PROTEIN_KINASE_DOM		K04424_04010_MAPK signaling pathway; K04424_04530_Tight junction; K02861_04210_Apoptosis; K02861_04620_Toll-like receptor signaling pathway;	2.7.11.1_Non-specificserine/threonineproteinkinase.;
MGC04175	5	838	Hsc70-interacting protein	TPR_1 ; TPR ; HSC70-INTERACTING PROTEIN ; TPR-like ; TPR REPEAT CONTAINING PROTEIN ; TPR_REGION			3.1.3.16_Phosphoproteinphosphatase.;
MGC04176	1	836	Tctex1 domain-containing protein 1-A	AXONEMAL DYNEIN LIGHT CHAIN ; Tctex-1 ; T-COMPLEX-ASSOCIATED-TESTIS-EXPRESSED 1/ DYNEIN LIGHT CHAIN	GO:0016020_C_membrane; GO:0005868_C_cytoplasmic dynein complex; GO:0005515_F_protein binding; GO:0009434_C_microtubule-based flagellum; GO:0007018_P_microtubule-based movement;		
MGC04178	1	560	Perlucin	REGENERATING GENE TYPE IV-RELATED ; C-type lectin-like ; C_TYPE_LECTIN_1 ; LITHOSTATHINE ; Lectin_C ; C_TYPE_LECTIN_2			
MGC04181	27	801	Peroxiredoxin-6	PEROXIREDOXIN ; AhpC-TSA ; PEROXIREDOXIN 6 RELATED ; Thioredoxin-like			1.11.1.7_Peroxidase.; 1.11.1.15_Peroxiredoxin.;
MGC04185	3	829					
MGC04196	1	628	Deleted in malignant brain tumors 1 protein	ShK ; Insulin-like ; SRCR-like ; SRCR ; CD5 ANTIGEN-LIKE PRECURSOR (APOPTOSIS INHIBITOR EXPRESSED BY MACROPHAGES) ; LYSYL OXIDASE-RELATED ; SRCR_2 ; SPERACTRCPTR			
MGC04197	1	738		TONB_DEPENDENT_REC_1			
MGC04198	2	551	Serine protease inhibitor dipetalogastin (Fragment)	Kazal_2 ; KAZAL ; SECRETORY TRYPSIN INHIBITOR ; SERINE PROTEASE INHIBITOR ; Kazal-type serine protease inhibitors		K06254_04512_ECM-receptor interaction;	
MGC04202	21	724	Lysozyme g	SLT ; LYSOZYMEG ; Lysozyme g ; Lysozyme-like			3.2.1.17_Lysozyme.;
MGC04204	4	857	Asialoglycoprotein receptor 1				
MGC04209	4	727	Peptidoglycan recognition protein 1	PEPTIDOGLYCAN RECOGNITION PROTEIN SB2, SC2 ; Amidase_2 ; PEPTIDOGLYCAN RECOGNITION PROTEIN ; N-acetylmuramoyl-L-alanine amidase-like		K01446_00550_Peptidoglycan biosynthesis;	3.5.1.28_N-acetylmuramoyl-L-alanineamidase.;
MGC04211	1	615	28 kDa heat- and acid-stable phosphoprotein	28 KDA HEAT- AND ACID-STABLE PHOSPHOPROTEIN (PDGF-ASSOCIATED PROTEIN) ; coiled-coil			
MGC04217	1	739	Protein lin-7 homolog C	PDZ ; LIN-7-LIKE PROTEINS ; L27 ; PDZ domain-like	GO:0007269_P_neurotransmitter secretion; GO:0005886_C_plasma membrane; GO:0005515_F_protein binding;		3.1.3.48_Protein-tyrosine-phosphatase.;
MGC04218	1	860	Spliceosome RNA helicase BAT1	Q_MOTIF ; P-loop containing nucleoside triphosphate hydrolases ; HELICASE_ATP_BIND_1 ; DEAD (ASP-GLU-ALA-ASP) BOX POLYPEPTIDE 39 AND P47 ; DEAD BOX ATP-DEPENDENT RNA HELICASE ; DEAD ; coiled-coil		K01529_00500_Starch and sucrose metabolism; K01529_00790_Folate biosynthesis; K01509_00230_Purine metabolism;	
MGC04224	19	835	Polycomb group protein Pc	Chromo ; CHROMO_1 ; CHROMOBOX PROTEIN ; CHROMO_2 ; Chromo domain-like			2.3.1.48_Histoneacetyltransferase.;

MGC04225	3	1180	MAM domain-containing glycosylphosphatidylinositol anchor protein 1	MAM DOMAIN-CONTAINING GLYCOSYLPHOSPHATIDYLINOSITOL ANCHOR PROTEIN 1 ; APICAL ENDOSOMAL GLYCOPROTEIN PRECURSOR. ; MAM_2 ; MAM		K06724_04360_Axon guidance;	3.4.21.9_Enteropeptidase.;
MGC04228	2	832	Heat shock protein 90	HEAT SHOCK PROTEIN 90 ; Ribosomal protein S5 domain 2-like ; HSP90	GO:0005737_C_cytoplasm; GO:0042803_F_protein homodimerization activity; GO:0005515_F_protein binding;	K04079_04612_Antigen processing and presentation; K04079_04914_Progesterone-mediated oocyte maturation; K04079_05215_Prostate cancer;	
MGC04229	1	729	Protein ERGIC-53	Lectin_leg-like ; Concanavalin A-like lectins/glucanases ; ERGIC-53 PROTEIN (MANNOSE-BINDING LECTIN 1)-RELATED ; VESICULAR MANNOSE-BINDING LECTIN	GO:0005515_F_protein binding;		
MGC04235	1	698	Lysyl oxidase homolog 2	SCAVENGER RECEPTOR-RELATED ; SRCR-like ; SRCR ; LYSYL OXIDASE-RELATED ; SRCR_2 ; SPERACTRCPTR			
MGC04246	17	720		Invertebrate chitin-binding proteins			
MGC04247	2	692	WSC domain-containing protein 2	WSC		K00771_00532_Chondroitin sulfate biosynthesis; K00771_01030_tba;	2.4.2.26_Proteinxylosyltransferase.;
MGC04249	1	868	Peptidoglycan recognition protein 3	Amidase_2 ; PEPTIDOGLYCAN RECOGNITION PROTEIN ; N-acetylmuramoyl-L-alanine amidase-like ; PEPTIDOGLYCAN RECOGNITION PROTEIN 4		K01446_00550_Peptidoglycan biosynthesis;	3.5.1.28_N-acetylmuramoyl-L-alanineamidase.;
MGC04253	10	737	Calmodulin	EF_HAND_1 ; CALMODULIN ; CALM_PAXIN_Q8X187; ; EF_HAND_2 ; EF-hand ; CALCIUM BINDING PROTEIN ; efhand			2.7.11.1_Non-specificserine/threonineproteinkinase.;
MGC04255	1	726	Lipopolysaccharide-induced tumor necrosis factor-alpha factor homolog	LIPOPOLYSACCHARIDE-INDUCED TRANSCRIPTION FACTOR REGULATING TUMOR NECROSIS FACTOR ALPHA			
MGC04259	2	820	Nudix hydrolase 8	NUDIX HYDROLASE RELATED ; GFGPROTEIN ; coiled-coil ; NUDIX ; NUDIXFAMILY ; Nudix			3.6.1.13_ADP-ribosediphosphatase.; 3.6.1.22_NAD(+)diphosphatase.;
MGC04260	6	855	Putative epidermal cell surface receptor	ShK ; Fibronectin type I module ; VWFC_1			
MGC04262	2	842	Complement C1q-like protein 4	C1q ; GLIACOLIN-RELATED ; CEREBELLIN-RELATED ; TNF-like ; coiled-coil ; C1Q			
MGC04266	1	842	Inversin	INVERSIN ; ANKYRIN ; Ank ; ANKYRIN REPEAT-CONTAINING ; P-loop containing nucleoside triphosphate hydrolases ; ANK_REPEAT ; IQ ; ANK_REP_REGION ; coiled-coil ; Ankyrin repeat	GO:0010171_P_body morphogenesis; GO:0001822_P_kidney development;	K08803_05219_Bladder cancer;	2.4.2.30_NAD(+)ADP-ribosyltransferase.; 2.7.11.1_Non-specificserine/threonineproteinkinase.;
MGC04267	3	752	Ficolin-2	FIBRINOGEN AND FIBRONECTIN ; Fibrinogen C-terminal domain-like ; Fibrinogen_C ; FIBRIN_AG_C_DOMAIN	GO:0008228_P_opsonization; GO:0005529_F_sugar binding; GO:0005509_F_calcium ion binding; GO:0003823_F_antigen binding;		
MGC04270	1	8001	Ficolin-2	FIBRINOGEN AND FIBRONECTIN ; Fibrinogen C-terminal domain-like ; Fibrinogen_C ; FIBRIN_AG_C_DOMAIN			
MGC04273	4	1179	Sporozoite surface protein 2				
MGC04275	1	597		DEATH domain ; coiled-coil			
MGC04280	10	770	Complement C1q-like protein 4	coiled-coil			
MGC04286	2	707					
MGC04287	1	719	Noggin-2	Noggin ; Cystine-knot cytokines ; BONE MORPHOGENETIC PROTEIN ANTAGONIST NOGGIN	GO:0030514_P_negative regulation of BMP signaling pathway;	K04658_04350_TGF-beta signaling pathway;	
MGC04295	2	728					

MGC04297	12	761	Complement C1q tumor necrosis factor-related protein 3	TNF-like ; coiled-coil			
MGC04299	2	793	Proteasome subunit beta type-7	N-terminal nucleophile aminohydrolases (Ntn hydrolases) ; Proteasome ; PROTEASOME_B ; PROTEASOME ; PROTEASOME SUBUNIT BETA TYPE 7,10 ; PROTEASOME SUBUNIT ALPHA/BETA		K02739_03050_Proteasome;	3.4.25.1_Proteasomeendopeptidasecomplex.;
MGC04301	3	1051	Actin, cytoplasmic 1	ACTINS_ACT_LIKE ; ACTIN ; Actin-like ATPase domain ; Actin ; ACTINS_1		K05692_04510_Focal adhesion; K05692_04520_Adherens junction; K05692_04530_Tight junction; K05692_04670_Leukocyte transendothelial migration; K05692_04810_Regulation of actin cytoskeleton;	2.7.10.2_Non-specificprotein-tyrosinekinase.;
MGC04308	3	769	Ankyrin repeat and sterile alpha motif domain-containing protein 1B	ANKYRIN ; Ank ; ANKYRIN REPEAT-CONTAINING ; ANK_REPEAT ; ANK_REP_REGION ; HSPC200 ; Ankyrin repeat	GO:0005515_F_protein binding;	K08803_05219_Bladder cancer;	2.7.11.1_Non-specificserine/threonineproteinkinase.;
MGC04314	1	814		Immunoglobulin			
MGC04315	11	1180	78 kDa glucose-regulated protein	HSP70_2 ; HEAT SHOCK PROTEIN 70KDA ; HEAT SHOCK PROTEIN 70 (HSP70) ; Q8I0E9_DROME_Q8I0E9; ; HSP70_1 ; HSP70 ; Actin-like ATPase domain ; coiled-coil ; HEATSHOCK70		K09490_05060_tba;	
MGC04318	22	1051	Cubilin				
MGC04326	1	819	Heat shock 70 kDa protein 12B	HSP70_1 ; Actin-like ATPase domain			
MGC04329	7	740	Collagen alpha-1(XII) chain	VWFA ; VON WILENBRAND FACTOR RELATED ; vWA-like ; VWA ; VWFADOMAIN			
MGC04330	1	701	14-3-3 protein epsilon	14-3-3 ; 1433ZETA ; Q8BPH1_MOUSE_Q8BPH1; ; 14-3-3 protein		K06630_04110_Cell cycle;	
MGC04337	1	847	Leucine-rich repeat-containing protein 61	LRR_1 ; Outer arm dynein light chain 1 ; LEUCINE RICH REPEAT FAMILY ; LEURICHRPT			
MGC04338	1	851	C-X-C chemokine receptor type 1	G_PROTEIN_RECEP_F1_2 ; G-PROTEIN COUPLED RECEPTOR ; GPCRRHODOPSN ; 7tm_1 ; Family A G protein-coupled receptor-like			
MGC04340	20	1545	SWI/SNF complex subunit SMARCC2	SWI/SNF COMPLEX-RELATED ; SWI/SNF-RELATED MATRIX-ASSOCIATED ACTIN-DEPENDENT REGULATOR OF CHROMATIN SUBFAMILY C MEMBER 2 (SMARCC2) ; SWIRM ; Myb_DNA-binding ; Homeodomain-like ; coiled-coil ; MYB_3			
MGC04341	2	627	C-type lectin domain family 4 member E	C-type lectin-like ; C-TYPE LECTIN SUPERFAMILY MEMBER ; Lectin_C ; CD209 ANTIGEN (DENDRITIC CELL-SPECIFIC ICAM-3-GRABBING NONINTEGRIN 1) (DC-SIGN1) ; ANTIFREEZEII ; C_TYPE_LECTIN_2	GO:0005529_F_sugar binding;		
MGC04350	7	805	Estradiol 17-beta-dehydrogenase 12	17-BETA HYDROXYSTEROID DEHYDROGENASE ; adh_short ; SHORT-CHAIN DEHYDROGENASES/REDUCTASE FAMILY MEMBER ; SDRFAMILY ; GDHRDH ; NAD(P)-binding Rossmann-fold domains		K00044_00150_Androgen and estrogen metabolism; K10251_01040_Biosynthesis of unsaturated fatty acids;	1.1.1.62_Estradiol17-beta-dehydrogenase.;
MGC04355	5	1398	Alpha-protein kinase vwkA	ALPHA KINASE/ELONGATION FACTOR 2 KINASE ; VWFA ; HEAT SHOCK 70 KDA PROTEIN 12 ; vWA-like ; VWA ; VWFADOMAIN			

MGC04365	1	788	Myopalladin	Immunoglobulin ; IG_LIKE ; I-set ; TITIN		K00907_04020_Calcium signaling pathway; K00907_04510_Focal adhesion; K00907_04810_Regulation of actin cytoskeleton; K05098_04060_Cytokine-cytokine receptor interaction; K05098_04370_VEGF signaling pathway; K05098_04510_Focal adhesion;	2.7.11.1_Non-specificserine/threonineproteinkinase.; 2.7.10.1_Receptorprotein-tyrosinekinase.; 2.7.11.18_[Myosinlight-chain]kinase.;
MGC04371	1	763	Spore wall protein 1	ShK			
MGC04373	1	640	Actin cytoskeleton-regulatory complex protein pan1	SAM/Pointed domain			
MGC04381	18	917	Periostin	FAS1 ; PERIOSTIN (PN) (OSTEOBLAST-SPECIFIC FACTOR 2) (OSF-2) ; Fasciclin ; PERIOSTIN-RELATED ; FAS1 domain			
MGC04382	10	811	Centaurin-gamma-1A	CENTAURIN/ARF ; Miro ; P-loop containing nucleoside triphosphate hydrolases ; RASTRNSFRMNG ; CENTAURIN/ARF-RELATED	GO:0007264_P_small GTPase mediated signal transduction; GO:0005515_F_protein binding; GO:0005634_C_nucleus;	K07836_04010_MAPK signaling pathway; K07836_04510_Focal adhesion; K07836_04670_Leukocyte transendothelial migration; K07836_04720_Long-term potentiation; K07836_05211_Renal cell carcinoma;	
MGC04383	7	741	Ganglioside GM2 activator	Ganglioside M2 (gm2) activator			
MGC04388	4	1001	Neurogenic locus notch homolog protein 2	EGF/Laminin ; EGF-LIKE DOMAIN PROTEIN ; EGF ; EGF_3 ; ASX_HYDROXYL ; CRUMBS(D.MELANOGASTER) RELATED ; EGF_1 ; EGF_2 ; EGFBLOOD		K02599_04320_Dorso-ventral axis formation; K02599_04330_Notch signaling pathway;	2.7.10.1_Receptorprotein-tyrosinekinase.;
MGC04391	6	1026	Meiosis-specific nuclear structural protein 1	MEIOSIS-SPECIFIC NUCLEAR STRUCTURAL PROTEIN 1 ; coiled-coil	GO:0005882_C_intermediate filament; GO:0005635_C_nuclear envelope;		
MGC04392	1	689	RNA-binding protein 12	ShK			
MGC04402	3	661	Complement C1q tumor necrosis factor-related protein 3	C1q ; CEREBELLIN-RELATED ; COMPLEMNTC1Q ; TNF-like ; C1Q			
MGC04408	3	688	Putative per-hexamer repeat protein 5	ShK ; Fibronectin type I module ; VWFC_2 ; CONNECTIVE TISSUE GROWTH FACTOR-RELATED ; VWFC_1			
MGC04410	1	753	WD repeat-containing protein KIAA1875	WD_REPEATS_2 ; GBLP_SCHPO_Q10281; ; GPROTEINBRPT ; WD40 repeat-like ; F-BOX AND WD40 DOMAIN PROTEIN ; WD_REPEATS_1 ; WD_REPEATS_REGION ; WD40		K00924_00562_Inositol phosphate metabolism; K00924_00632_Benzoate degradation via CoA ligation; K03130_03022_Basal transcription factors; K06666_04111_Cell cycle - yeast;	2.7.11.1_Non-specificserine/threonineproteinkinase.; 2.7.11.7_[Myosinheavy-chain]kinase.;
MGC04412	1	785	Scavenger receptor cysteine-rich type 1 protein M130	SRCR_1 ; SRCR-like ; SRCR ; CD5 ANTIGEN-LIKE PRECURSOR (APOPTOSIS INHIBITOR EXPRESSED BY MACROPHAGES) ; LYSYL OXIDASE-RELATED ; SRCR_2 ; SPERACTRCPTR			
MGC04422	4	695	Scavenger receptor class F member 2	EGF/Laminin ; EGF-LIKE DOMAIN PROTEIN ; LAMININ EGF-LIKE DOMAIN ; EGF_1 ; EGF_2			2.7.10.1_Receptorprotein-tyrosinekinase.;
MGC04428	1	801	RING finger protein 213	RING/U-box ; ZF_RING_2			
MGC04429	2	1015	Heavy metal-binding protein HIP	C1q ; CEREBELLIN-RELATED ; COMPLEMNTC1Q ; TNF-like			
MGC04434	1	677					
MGC04435	5	752		Snake toxin-like ; UPAR_LY6			

MGC04439	5	767	Tenascin-R	FIBRINOGEN AND FIBRONECTIN ; Fibrinogen C-terminal domain-like ; Fibrinogen_C ; FIBRIN_AG_C_DOMAIN	GO:0046625_F_sphingolipid binding; GO:0007155_P_cell adhesion; GO:0030198_P_extracellular matrix organization and biogenesis; GO:0005515_F_protein binding; GO:0007411_P_axon guidance; GO:0045121_C_membrane raft; GO:0009986_C_cell surface;	K06252_04510_Focal adhesion; K06252_04512_ECM-receptor interaction;		
MGC04441	2	759	Chromodomain-helicase-DNA-binding protein 1	HELICASE_CTER ; Helicase_C ; P-loop containing nucleoside triphosphate hydrolases ; CHROMODOMAIN HELICASE DNA BINDING PROTEIN ; ATP-DEPENDENT HELICASE SMARCA (SWI/SNF-RELATED MATRIX-ASSOCIATED ACTIN-DEPENDENT REGULATOR OF CHROMATIN A)-RELATED				
MGC04443	2	538	Macrophage mannose receptor 1	C-type lectin-like ; C_TYPE_LECTIN_1 ; C-TYPE LECTIN SUPERFAMILY MEMBER ; Lectin_C ; GALACTOSE-SPECIFIC C-TYPE LECTIN ; C_TYPE_LECTIN_2				
MGC04449	2	682	Heat shock 70 kDa protein 12A	ALPHA KINASE/ELONGATION FACTOR 2 KINASE ; Actin-like ATPase domain ; HEAT SHOCK 70 KDA PROTEIN 12	GO:0048514_P_blood vessel morphogenesis; GO:0002040_P_sprouting angiogenesis;			
MGC04450	2	961	ETS homologous factor	ETS ; ETSDOMAIN ; EPITHELIUM SPECIFIC ETS FACTOR 3, ESE3 ; 'Winged helix' DNA-binding domain ; ETS_DOMAIN_3 ; Ets				
MGC04451	4	814	Collagen alpha-1(XII) chain	VWFA ; VON WILENBRAND FACTOR RELATED ; vWA-like ; VWA ; VWFADOMAIN				
MGC04454	1	725						
MGC04455	2	945	Mediator of RNA polymerase II transcription subunit 20	UBIQUITIN SPECIFIC PROTEASE HOMOLOG 49 ; TATA_RF		K00960_03020_RNA polymerase;		
MGC04458	1	696	Baculoviral IAP repeat-containing protein 3					
MGC04464	1	629	Trichohyalin	CALPONIN HOMOLOGY (CH) DOMAIN CONTAINING, CHDC/LRCH ; LEUCINE-RICH REPEAT-CONTAINING PROTEIN ; coiled-coil				
MGC04466	1	761	Prostaglandin E2 receptor EP4 subtype	G_PROTEIN_RECEP_F1_2 ; PROSTANOIDR ; PROSTAGLANDIN RECEPTOR ; G_PROTEIN_RECEP_F1_1 ; GPCRRHODOPSN ; 7tm_1 ; Family A G protein-coupled receptor-like		K04261_04080_Neuroactive ligand-receptor interaction; K04259_04080_Neuroactive ligand-receptor interaction;		
MGC04467	6	789	Vitelline membrane outer layer protein 1	VOMI ; Vitelline membrane outer protein-I (VMO-I) ; VITELLINE MEMBRANE OUTER LAYER PROTEIN I-RELATED				
MGC04472	1	780	60 kDa heat shock protein, mitochondrial	GroEL apical domain-like ; GroEL equatorial domain-like ; CHAPERONIN-60KDA, CH60 ; Cpn60_TCP1 ; TCOMPLEXTCP1 ; CHAPERONINS_CPN60 ; coiled-coil ; CHAPERONIN ; CHAPERONIN60		K04077_04940_Type I diabetes mellitus; K04077_05060_tba;		

MGC04473	2	584	Dual oxidase	OXIDASE/PEROXIDASE ; Heme-dependent peroxidases ; PEROXIDASE_3 ; An_peroxidase	GO:0051591_P_response to cAMP; GO:0004601_F_peroxidase activity; GO:0019221_P_cytokine and chemokine mediated signaling pathway; GO:0042335_P_cuticle development; GO:0042742_P_defense response to bacterium;	K00430_00360_Phenylalanine metabolism; K00430_00680_Methane metabolism; K00430_00940_Phenylpropanoid biosynthesis;	1.6.3.1_NAD(P)Hoxidase.;
MGC04475	2	593	DnaJ homolog subfamily C member 2	DNAJ/HSP40 ; DNAJ_2 ; DnaJ ; DNAJ_1 ; Chaperone J-domain ; ZUOTIN RELATED FACTOR 4	GO:0006457_P_protein folding; GO:0051082_F_unfolded protein binding; GO:0005634_C_nucleus;		
MGC04478	3	1264		Chitin_bind_3			
MGC04479	1	747	Plasminogen	KRINGLE_1 ; SERINE PROTEASE-RELATED ; Kringle-like ; PLMN_PIG_P06867; ; PLASMINOGEN ; KRINGLE_2 ; KRINGLE ; Kringle		K01315_04080_Neuroactive ligand-receptor interaction; K01315_04610_Complement and coagulation cascades;	3.4.21.7_Plasmin.;
MGC04489	2	679	CD209 antigen-like protein D	C-type lectin-like ; C-TYPE LECTIN SUPERFAMILY MEMBER ; Lectin_C ; CD209 ANTIGEN (DENDRITIC CELL-SPECIFIC ICAM-3-GRABBING NONINTEGRIN 1) (DC-SIGN1) ; coiled-coil ; C_TYPE_LECTIN_2	GO:0042803_F_protein homodimerization activity; GO:0005537_F_mannose binding;		
MGC04498	1	601	B(0,+)-type amino acid transporter 1	CATIONIC AMINO ACID TRANSPORTER SLC7A11-RELATED ; AMINO ACID TRANSPORTER			
MGC04501	4	794	Fucolectin-6				
MGC04506	4	899	Ficolin-1	FIBRINOGEN AND FIBRONECTIN ; Fibrinogen C-terminal domain-like ; Fibrinogen_C		K06252_04510_Focal adhesion; K06252_04512_ECM-receptor interaction;	
MGC04510	2	756					
MGC04511	1	760	Serine/threonine-protein kinase 16	Protein kinase-like (PK-like) ; PROTEIN_KINASE_ST ; Pkinase ; Q6GLG5_XENTR_Q6GLG5; ; SERINE/THREONINE KINASE 16 (STK16) ; SERINE/THREONINE PROTEIN KINASE ; PROTEIN_KINASE_DOM			2.7.11.1_Non-specificserine/threonineproteinkinase.;
MGC04512	2	1156	Hsp90 co-chaperone Cdc37	Hsp90 co-chaperone CDC37 ; CDC37_C ; CDC37-RELATED ; CDC37_N ; coiled-coil ; CDC37_M	GO:0005737_C_cytoplasm; GO:0006605_P_protein targeting; GO:0051082_F_unfolded protein binding; GO:0000079_P_regulation of cyclin-dependent protein kinase activity; GO:0005515_F_protein binding;		
MGC04514	4	735	Aggrecan core protein	C-type lectin-like ; C_TYPE_LECTIN_1 ; C-TYPE LECTIN SUPERFAMILY MEMBER ; Lectin_C ; GALACTOSE-SPECIFIC C-TYPE LECTIN ; C_TYPE_LECTIN_2			
MGC04517	8	741	Complement C1q tumor necrosis factor-related protein 3	C1q ; C1Q-RELATED FACTOR ; COLLAGEN ALPHA CHAIN ; COMPLEMNTC1Q ; TNF-like ; C1Q	GO:0005576_C_extracellular region;		
MGC04518	17	850	Fibrinogen C domain-containing protein 1	FIBRINOGEN AND FIBRONECTIN ; Fibrinogen C-terminal domain-like ; Fibrinogen_C ; FIBRIN_AG_C_DOMAIN			
MGC04524	2	807	Protein outspread	PH ; PH_DOMAIN ; PLECKSTRIN HOMOLOGY (PH) DOMAIN-CONTAINING PROTEIN ; PH domain-like ; RHO INTERACTING PROTEIN 3 (RIP3)	GO:0005515_F_protein binding;		
MGC04529	1	601	Complement C1q tumor necrosis factor-related protein 6	TNF-like			
MGC04534	1	881	Kynurenine formamidase	Putative cyclase ; Cyclase			

MGC04536	3	813	Calmodulin	EF_HAND_1 ; CALMODULIN ; EF_HAND_2 ; CALM_HALOK_Q95NI4; ; EF-hand ; CALCIUM BINDING PROTEIN ; efhand			2.7.11.1_Non-specificserine/threonineprotein kinase.;
MGC04538	2	775	Coronin-1B	WD_REPEATS_2 ; CORONIN ; DUF1899 ; GPROTEINBRPT ; Q6P7R0_RAT_Q6P7R0; ; WD40 repeat-like ; WD_REPEATS_1 ; WD_REPEATS_REGION ; WD40	GO:0005515_F_protein binding;	K01852_00100_Biosynthesis of steroids; K01062_00565_Ether lipid metabolism;	2.7.11.1_Non-specificserine/threonineproteinkinase.; 2.3.1.48_Histoneacetyltransferase.;
MGC04540	1	766	Complement C1q-like protein 4	C1q ; GLIACOLIN-RELATED ; CEREBELLIN-RELATED ; COMPLEMNTC1Q ; TNF-like ; coiled-coil ; C1Q			
MGC04547	1	713	Annexin A11	ANXD_HUMAN_P27216; ; Annexin ; ANNEXIN			
MGC04562	8	780					
MGC04568	1	779	5'-tyrosyl-DNA phosphodiesterase	UBA-like ; Exo_endo_phos ; DNase I-like	GO:0007166_P_cell surface receptor linked signal transduction; GO:0007369_P_gastrulation; GO:0007368_P_determination of left/right symmetry; GO:0003714_F_transcription corepressor activity;		
MGC04573	6	751	Angiopoietin-4	FIBRINOGEN AND FIBRONECTIN ; Fibrinogen C-terminal domain-like ; Fibrinogen_C ; coiled-coil	GO:0030297_F_transmembrane receptor protein tyrosine kinase activator activity; GO:0006979_P_response to oxidative stress;		
MGC04576	1	690	C-type lectin domain family 4 member F	C-type lectin-like ; FRAS1 RELATED EXTRACELLULAR MATRIX PROTEIN 1 ; C-TYPE LECTIN SUPERFAMILY MEMBER ; Lectin_C ; coiled-coil ; ANTIFREEZEII ; C_TYPE_LECTIN_2			
MGC04577	4	754	Short-chain collagen C4 (Fragment)				
MGC04578	10	620	Heavy metal-binding protein HIP	C1q ; CEREBELLIN-RELATED ; COMPLEMNTC1Q ; TNF-like ; C1Q			
MGC04582	1	712	Arrestin domain-containing protein 3	ARRESTIN DOMAIN CONTAINING PROTEIN ; E set domains ; Arrestin_N ; Arrestin_C	GO:0042802_F_identical protein binding; GO:0005515_F_protein binding;		
MGC04583	1	698	Collagen alpha-2(VIII) chain	C1q ; COLLAGEN ALPHA 1(VIII) CHAIN ; COLLAGEN ALPHA CHAIN ; COMPLEMNTC1Q ; TNF-like ; coiled-coil ; C1Q			
MGC04587	1	603					
MGC04590	1	377		C1q ; C1Q-RELATED FACTOR ; COLLAGEN ALPHA CHAIN ; TNF-like ; coiled-coil			
MGC04594	1	539	Rho-related protein racA	P-loop containing nucleoside triphosphate hydrolases ; RASTRNSFRMNG ; RAS-RELATED PROTEIN RAC ; RAS-RELATED GTPASE ; small_GTP: small GTP-binding protein domain ; Ras ; SIGMA54_INTERACT_1			
MGC04603	5	765	Endoplasmin	HEAT SHOCK PROTEIN 90 ; HEATSHOCK90 ; HATPase_c ; HSP90 ; ATPase domain of HSP90 chaperone/DNA topoisomerase II/histidine kinase ; ENDOPLASMIN		K09487_05215_Prostate cancer;	
MGC04607	3	1078	Calmodulin	EF_HAND_1 ; CALMODULIN ; EF_HAND_2 ; CALF_NAEGR_P53440; ; EF-hand ; CALCIUM BINDING PROTEIN ; efhand			2.7.11.1_Non-specificserine/threonineproteinkinase.;
MGC04608	1	694	Collagen alpha-1(XII) chain	VWFA ; INTEGRIN ALPHA-RELATED ; VON WILENBRAND FACTOR RELATED ; vWA-like ; VWA ; VWFADOMAIN			

MGC04609	1	698	IgGFc-binding protein				
MGC04610	3	818	Fibrinogen-like protein A	FIBRINOGEN AND FIBRONECTIN ; Fibrinogen C-terminal domain-like ; Fibrinogen_C			
MGC04615	1	651	Scavenger receptor cysteine-rich type 1 protein M130	PII_GLNB_CTER ; SRCR_1 ; SRCR-like ; SRCR ; LYSYL OXIDASE-RELATED ; SRCR_2 ; SPERACTRCPTR ; DELETED IN MALIGNANT BRAIN TUMORS 1			
MGC04616	12	946	PHD and RING finger domain-containing protein 1	FYVE/PHD zinc finger ; JUMONJI/ARID DOMAIN-CONTAINING PROTEIN 1 ; ZF_PHD_2 ; RING/U-box ; JUMONJI DOMAIN CONTAINING PROTEIN ; ZF_RING_1 ; ZF_PHD_1 ; zf-C3HC4 ; ZF_RING_2 ; PHD ; coiled-coil			2.1.1.43_Histone-lysineN-methyltransferase.; 2.3.1.48_Histoneacetyltransferase.;
MGC04620	1	589	Extensin-2				
MGC04623	1	618	Thyroglobulin	INSULIN-LIKE GROWTH FACTOR BINDING PROTEIN ; Thyroglobulin_1 ; Thyroglobulin type-1 domain ; INSULIN-LIKE GROWTH FACTOR BINDING PROTEIN 3 ; THYROGLOBULIN_1_2	GO:0007165_P_signal transduction; GO:0005576_C_extracellular region;	K10809_05320_Autoimmune thyroid disease;	
MGC04625	1	660	Fucolectin-4	Galactose-binding domain-like ; F5_F8_type_C			
MGC04631	10	724					
MGC04638	4	633	Complement C1q-like protein 3	C1q ; C1Q-RELATED FACTOR ; COLLAGEN ALPHA CHAIN ; COMPLEMNTC1Q ; TNF-like ; C1Q	GO:0005515_F_protein binding;		
MGC04640	5	818	Peritrophin-1	CBM_14 ; CHIT_BIND_II ; Invertebrate chitin-binding proteins			
MGC04645	2	754	ATP-dependent RNA helicase DDX54	DDX54 PROTEIN ; DBP10CT ; DEAD BOX ATP-DEPENDENT RNA HELICASE		K01529_00500_Starch and sucrose metabolism; K01529_00790_Folate biosynthesis;	
MGC04646	2	768	Plasma kallikrein	SERINE PROTEASE-RELATED ; Trypsin ; CHYMOTRYPSIN ; OVIDUCTIN ; TRYPSIN_DOM ; Trypsin-like serine proteases		K01324_04610_Complement and coagulation cascades;	3.4.21.34_Plasmakallikrein.; 3.4.21.4_Trypsin.;
MGC04647	1	711	Fibrinogen C domain-containing protein 1-B	FIBRINOGEN AND FIBRONECTIN ; Fibrinogen C-terminal domain-like ; Fibrinogen_C		K06252_04510_Focal adhesion; K06252_04512_ECM-receptor interaction;	
MGC04653	2	774	C3 and PZP-like alpha-2-macroglobulin domain-containing protein 8	C-type lectin-like ; C-TYPE LECTIN PROTEINS	GO:0005515_F_protein binding;		
MGC04660	1	806	Serine/threonine-protein kinase PAK 2	CRIB ; Wiscott-Aldrich syndrome protein, WASP, C-terminal domain ; PBD ; WISKOTT-ALDRICH SYNDROME PROTEIN			2.7.11.1_Non-specificserine/threonineproteinkinase.;
MGC04662	7	1384		ZINC METALLOPROTEINASE NAS-RELATED ; DISCOIDIN, CUB, EGF, LAMININ , AND ZINC METALLOPROTEASE DOMAIN ; Metalloproteases ('zincins'), catalytic domain ; Astacin			
MGC04668	3	1047	Neurogenic locus Notch protein	EGF/Laminin ; t-snare proteins ; EGF-LIKE DOMAIN PROTEIN ; EGF ; EGF_3 ; CRUMBS(D.MELANOGASTER) RELATED ; EGF_1 ; EGF_2 ; coiled-coil		K06051_04330_Notch signaling pathway; K02599_04320_Dorso-ventral axis formation; K02599_04330_Notch signaling pathway;	
MGC04677	1	804	Rhamnose-binding lectin	G-PROTEIN COUPLED RECEPTOR ; Gal_Lectin ; SUEL_LECTIN ; LATROPHILIN ; gb def: Mus musculus 13 days embryo heart cDNA, RIKEN full-length enriched library, clon			

MGC04681	6	990	Peptidase inhibitor 16	ShK ; Sea anemone toxin k ; SCP ; CYSTEINE-RICH SECRETORY PROTEIN (CRISP/SCP/TPX1)-RELATED ; V5TPXLIKE ; PR-1-like ; Q6UWH0_HUMAN_Q6UWH0; ; CRISP SUBFAMILY GLIOMA PATHOGENESIS-RELATED PROTEIN-RELATED			
MGC04686	1	785	ATP-dependent RNA helicase DDX54	HELICASE_CTER ; Helicase_C ; DEAD-BOX PROTEIN 54 ; P-loop containing nucleoside triphosphate hydrolases ; DEAD BOX ATP-DEPENDENT RNA HELICASE ; coiled-coil		K01529_00500_Starch and sucrose metabolism; K01529_00790_Folate biosynthesis;	
MGC04687	1	750	Neuromedin-U receptor 1	Family A G protein-coupled receptor-like			
MGC04688	3	1259	von Willebrand factor A domain-containing protein 5A	VON WILLEBRAND FACTOR, TYPE A DOMAIN CONTAINING ; VWFA ; BREAST CANCER SUPPRESSOR CANDIDATE 1,BCSC-1 ; VIT ; vWA-like ; VWA		K10798_03410_Base excision repair;	2.4.2.30_NAD(+)ADP-ribosyltransferase.;
MGC04695	9	1281		coiled-coil			
MGC04696	3	704	Uncharacterized protein KIAA1751 homolog	coiled-coil			
MGC04698	1	791	Alpha- and gamma-adaptin-binding protein p34		GO:0005737_C_cytoplasm;		
MGC04706	1	710	Complement C1q tumor necrosis factor-related protein 2	C1q ; COLLAGEN ALPHA CHAIN ; COMPLEMNTC1Q ; COMPLEMENT-C1Q TUMOR NECROSIS FACTOR-RELATED ; TNF-like ; C1Q			
MGC04707	2	910	Ovochymase-1	SERINE PROTEASE-RELATED ; PTS_HPR_HIS ; Trypsin ; CHYMOTRYPSIN ; SERINE PROTEASE-RELATED, INSECT ; TRYPSIN_DOM ; Trypsin-like serine proteases	GO:0005515_F_protein binding; GO:0006508_P_proteolysis; GO:0004295_F_trypsin activity;	K01324_04610_Complement and coagulation cascades; K01312_04080_Neuroactive ligand-receptor interaction; K01315_04080_Neuroactive ligand-receptor interaction; K01315_04610_Complement and coagulation cascades;	3.4.21.34_Plasmakallikrein.; 3.4.21.4_Trypsin.;
MGC04713	9	768	Peptidoglycan-recognition protein SC2	PEPTIDOGLYCAN RECOGNITION PROTEIN SB2, SC2 ; Amidase_2 ; PEPTIDOGLYCAN RECOGNITION PROTEIN ; N-acetylmuramoyl-L-alanine amidase-like		K01446_00550_Peptidoglycan biosynthesis;	3.5.1.28_N-acetylmuramoyl-L-alanineamidase.;
MGC04714	2	996	Fibrinogen-like protein A	FIBRINOGEN AND FIBRONECTIN ; Fibrinogen C-terminal domain-like ; Fibrinogen_C		K06252_04510_Focal adhesion; K06252_04512_ECM-receptor interaction;	
MGC04716	1	783					
MGC04717	1	755		Immunoglobulin			
MGC04718	4	1234	Peptidyl-prolyl cis-trans isomerase G	PEPTIDYL-PROLYL CIS-TRANS ISOMERASE G, PPIG ; Cyclophilin-like ; CSAPPISMRASE ; CYCLOPHILIN ; coiled-coil ; Pro_isomerase ; CSA_PPIASE_2	GO:0005654_C_nucleoplasm; GO:0008380_P_RNA splicing; GO:0005634_C_nucleus; GO:0016018_F_cyclosporin A binding;	K05864_04020_Calcium signaling pathway;	5.2.1.8_Peptidylprolylisomerase.;
MGC04720	2	985	Equistatin	HLA CLASS II GAMMA CHAIN ; Thyroglobulin_1 ; Thyroglobulin type-1 domain ; THYROGLOBULIN_1_1 ; THYROGLOBULIN_1_2		K10809_05320_Autoimmune thyroid disease;	
MGC04722	3	804	Perlucin	REGENERATING GENE TYPE IV-RELATED ; C-type lectin-like ; C_TYPE_LECTIN_1 ; LITHOSTATHINE ; Lectin_C ; ANTIFREEZEII ; C_TYPE_LECTIN_2	GO:0030246_F_carbohydrate binding;		
MGC04725	3	547	Complement C1q tumor necrosis factor-related protein 3	C1q ; C1Q-RELATED FACTOR ; COLLAGEN ALPHA CHAIN ; COMPLEMNTC1Q ; TNF-like ; C1Q		K03987_04610_Complement and coagulation cascades; K03987_05010_Alzheimers disease;	
MGC04729	1	695	Complement C1q-like protein 4	C1q ; GPI-ANCHORED PROTEIN P137 ; COMPLEMNTC1Q ; TNF-like ; coiled-coil ; C1Q			

MGC04732	2	688	Immunoglobulin superfamily member 10	L domain-like ; LEURICHRPT ; LEUCINE-RICH TRANSMEMBRANE PROTEINS	GO:0001503_P_ossification; GO:0042552_P_myelination; GO:0042551_P_neuron maturation;	K06838_04360_Axon guidance; K06839_04360_Axon guidance; K06850_04360_Axon guidance;	2.7.10.1_Receptorprotein-tyrosinekinase.; 1.11.1.7_Peroxidase.;
MGC04734	2	587	C-type lectin domain family 10 member A	C-type lectin-like ; C-TYPE LECTIN SUPERFAMILY MEMBER ; Lectin_C ; CD209 ANTIGEN (DENDRITIC CELL-SPECIFIC ICAM-3-GRABBING NONINTEGRIN 1) (DC-SIGN1) ; ANTIFREEZEII ; C_TYPE_LECTIN_2	GO:0005537_F_mannose binding;		
MGC04740	3	1310	Collagen alpha-4(VI) chain	Q6CAY0_EEEEE_Q6CAY0; ; VWFA ; VON WILENBRAND FACTOR RELATED ; vWA-like ; VWA ; VWFADOMAIN		K06238_04510_Focal adhesion; K06238_04512_ECM-receptor interaction;	
MGC04752	3	596	Neurogenic locus notch homolog protein 2	EGF/Laminin ; NOTCH ; EGF-LIKE DOMAIN PROTEIN ; EGF ; EGF_3 ; ASX_HYDROXYL ; EGF_1 ; EGF_2 ; EGF_CA		K02599_04320_Dorso-ventral axis formation; K02599_04330_Notch signaling pathway;	
MGC04759	5	610	Perlucin	C-type lectin-like ; C_TYPE_LECTIN_1 ; C-TYPE LECTIN SUPERFAMILY MEMBER ; Lectin_C ; GALACTOSE-SPECIFIC C-TYPE LECTIN ; C_TYPE_LECTIN_2			
MGC04761	1	731	Pre-mRNA-processing factor 19	PRP19/PSO4 HOMOLOG (NUCLEAR MATRIX PROTEIN 200) ; WD_REPEATS_2 ; PRE-MRNA SPLICING FACTOR PRP19-RELATED ; GPROTEINBRPT ; WD40 repeat-like ; Q6GLG2_XENTR_Q6GLG2; ; WD_REPEATS_1 ; WD_REPEATS_REGION ; WD40		K10599_04120_Ubiquitin mediated proteolysis;	2.7.11.1_Non-specificserine/threonineproteinkinase.; 2.4.1.37_Fucosylgalactoside3-alpha-galactosyltransferase.;
MGC04769	1	730	Filamin-C	FILAMIN ; SPECTRIN-LIKE CELL STRUCTURE PROTEIN ; E set domains ; FILAMIN_REPEAT ; Filamin		K04437_04010_MAPK signaling pathway; K04437_04510_Focal adhesion;	
MGC04770	2	709	Uncharacterized protein C9orf93	coiled-coil			
MGC04773	1	688					
MGC04775	1	781	Fibrinogen C domain-containing protein 1	FIBRINOGEN AND FIBRONECTIN ; Fibrinogen C-terminal domain-like ; Fibrinogen_C		K06252_04510_Focal adhesion; K06252_04512_ECM-receptor interaction;	
MGC04779	2	746	Serine/threonine-protein kinase SRPK2	Protein kinase-like (PK-like) ; SRPK ; PROTEIN_KINASE_ST ; PROTEIN_KINASE_ATP ; Pkinase ; Q6V9W0_RAT_Q6V9W0; ; CDC2, MAP KINASE-RELATED ; PROTEIN_KINASE_DOM			2.7.11.1_Non-specificserine/threonineproteinkinase.;
MGC04782	7	841	Chymotrypsin-like serine proteinase	TRYPSIN_HIS ; SERINE PROTEASE-RELATED ; Trypsin ; TRYPSIN_SER ; CHYMOTRYPSIN ; SERINE PROTEASE-RELATED, INSECT ; TRYPSIN_DOM ; Trypsin-like serine proteases		K01312_04080_Neuroactive ligand-receptor interaction;	3.4.21.1_Chymotrypsin.; 3.4.21.4_Trypsin.;
MGC04783	2	1259	Alpha-protein kinase vwkA	ALPHA KINASE/ELONGATION FACTOR 2 KINASE ; VWFA ; HEAT SHOCK 70 KDA PROTEIN 12 ; vWA-like ; VWA			
MGC04785	2	866		Invertebrate chitin-binding proteins			
MGC04794	1	740	Protein BMH1	1433_2 ; 143B_HORVU_Q43470; ; 14-3-3 ; 1433ZETA ; 14-3-3 protein		K06630_04110_Cell cycle;	
MGC04795	2	588		Lipocalins			

MGC04800	1	378	Collagen alpha-1(X) chain	C1q ; GLIACOLIN-RELATED ; CEREBELLIN-RELATED ; TNF-like ; C1Q	GO:0005581_C_collagen; GO:0001501_P_skeletal development;	K03986_04610_Complement and coagulation cascades; K03986_05010_Alzheimers disease; K03987_04610_Complement and coagulation cascades; K03987_05010_Alzheimers disease;	
MGC04802	2	653	Protein strawberry notch homolog 1	STRAWBERRY NOTCH-RELATED			
MGC04808	2	567	EF-hand domain-containing protein D2	EF_HAND_1 ; SWIPROSIN ; EF_HAND_2 ; Q7Z2R5_HUMAN_Q7Z2R5; ; EF-hand ; efhand	GO:0005515_F_protein binding;		
MGC04809	1	485	Techylectin-5B	FIBRINOGEN AND FIBRONECTIN ; Fibrinogen C-terminal domain-like ; Fibrinogen_C ; FIBRIN_AG_C_DOMAIN		K06252_04510_Focal adhesion; K06252_04512_ECM-receptor interaction;	
MGC04813	9	1052	PC4 and SFRS1-interacting protein	Tudor/PWWP/MBT ; PWWP ; HEPATOMA-DERIVED GROWTH FACTOR-RELATED ; HEPATOMA DERIVED GROWTH FACTOR 2, 3 ; coiled-coil			
MGC04816	1	727	Ataxin-10	ATAXIN-10 ; ARM repeat			
MGC04820	3	1024	Papilin	Leech antihemostatic proteins ; BPTI-like			
MGC04822	1	511	Kinase D-interacting substrate of 220 kDa	ANK REPEAT-CONTAINING ; ANKYRIN ; Ank ; ANKYRIN REPEAT-CONTAINING ; ANK_REPEAT ; ANK_REP_REGION ; Ankyrin repeat			2.7.11.1_Non-specificserine/threonineproteinkinase.; 2.4.2.30_NAD(+)ADP-ribosyltransferase.; 2.1.1.43_Histone-lysineN-methyltransferase.;
MGC04833	2	643	Fucolectin-6	Galactose-binding domain-like ; F5_F8_type_C			
MGC04834	2	680	Synaptosomal-associated protein 29	T_SNARE ; SYNAPTOSOMAL ASSOCIATED PROTEIN ; coiled-coil	GO:0005515_F_protein binding;	K08509_04130_SNARE interactions in vesicular transport;	
MGC04843	1	671		C1q ; TNF-like ; coiled-coil			
MGC04850	1	392	Elongator complex protein 1	IKAPPAB KINASE COMPLEX-ASSOCIATED PROTEIN			
MGC04857	1	678	Equistatin	HLA CLASS II GAMMA CHAIN ; Thyroglobulin_1 ; MHC CLASS II-ASSOCIATED INVARIANT CHAIN-RELATED ; Thyroglobulin type-1 domain ; THYROGLOBULIN_1_1 ; THYROGLOBULIN_1_2		K10809_05320_Autoimmune thyroid disease;	
MGC04860	1	629	CD209 antigen	C-type lectin-like ; C-TYPE LECTIN SUPERFAMILY MEMBER ; Lectin_C ; CD209 ANTIGEN (DENDRITIC CELL-SPECIFIC ICAM-3-GRABBING NONINTEGRIN 1) (DC-SIGN1) ; ANTIFREEZEII ; C_TYPE_LECTIN_2			
MGC04868	1	626	Hepatic lectin	C-type lectin-like ; THIOL_PROTEASE_CYS ; C_TYPE_LECTIN_1 ; C-TYPE LECTIN SUPERFAMILY MEMBER ; Lectin_C ; GALACTOSE-SPECIFIC C-TYPE LECTIN ; coiled-coil ; C_TYPE_LECTIN_2			
MGC04872	1	483	Nucleolar protein 58				
MGC04873	1	638	Chymotrypsin BII	SERINE PROTEASE-RELATED ; Trypsin ; SERINE PROTEASE-RELATED, INSECT ; TRYPSIN_DOM ; Trypsin-like serine proteases		K01344_04610_Complement and coagulation cascades; K01312_04080_Neuroactive ligand-receptor interaction;	3.4.21.32_Brachyurin.; 3.4.21.1_Chymotrypsin.; 3.4.21.4_Trypsin.;
MGC04874	2	858	Teneurin-2				
MGC04875	1	664	Fibrinogen-like protein 1	FIBRINOGEN AND FIBRONECTIN ; Fibrinogen C-terminal domain-like ; Fibrinogen_C	GO:0007411_P_axon guidance;	K06252_04510_Focal adhesion; K06252_04512_ECM-receptor interaction;	
MGC04881	2	667	Complement C1q tumor necrosis factor-related protein 3	C1q ; C1Q-RELATED FACTOR ; COLLAGEN ALPHA CHAIN ; COMPLEMNTC1Q ; TNF-like ; C1Q			
MGC04886	2	597					

MGC04887	3	1049	Tankyrase-2	ANKYRIN ; ION CHANNEL NOMPC ; Ank ; ANKYRIN REPEAT-CONTAINING ; P-loop containing nucleoside triphosphate hydrolases ; ANK_REPEAT ; ANK_REP_REGION ; Ankyrin repeat		K08803_05219_Bladder cancer;	2.7.11.1_Non-specificserine/threonineprotein kinase.; 2.4.2.30_NAD(+)ADP-ribosyltransferase.; 2.1.1.43_Histone-lysineN-methyltransferase.;
MGC04890	2	797	Palladin	Immunoglobulin ; IG_LIKE ; I-set ; TITIN (NEXIN) ; TITIN	GO:0007498_P_mesoderm development; GO:0004674_F_protein serine/threonine kinase activity; GO:0006468_P_protein amino acid phosphorylation;	K00907_04020_Calcium signaling pathway; K00907_04510_Focal adhesion; K00907_04810_Regulation of actin cytoskeleton; K06765_04360_Axon guidance; K06765_05210_Colorectal cancer;	2.7.11.1_Non-specificserine/threonineprotein kinase.; 2.7.11.18_[Myosinlight-chain]kinase.;
MGC04908	5	913	Complement C1q-like protein 3	C1q ; COLLAGEN ALPHA 1(VIII) CHAIN ; COLLAGEN ALPHA CHAIN ; COMPLEMNTC1Q ; TNF-like ; coiled-coil ; C1Q	GO:0005515_F_protein binding;		
MGC04915	1	670	Prestalk protein				
MGC04918	1	485	C-type lectin domain family 7 member A	C-type lectin-like			
MGC04922	1	317	Ribosomal protein S6 kinase beta-1	Protein kinase-like (PK-like) ; PROTEIN_KINASE_ATP ; Pkinase ; Q8C3J7_MOUSE_Q8C3J7; ; SERINE/THREONINE PROTEIN KINASE ; RIBOSOMAL PROTEIN S6 KINASE BETA ; PROTEIN_KINASE_DOM		K04688_04012_ErbB signaling pathway; K04688_04150_mTOR signaling pathway; K04688_04350_TGF-beta signaling pathway; K04688_04910_Insulin signaling pathway; K04688_05221_Acute myeloid leukemia;	2.7.11.1_Non-specificserine/threonineprotein kinase.;
MGC04926	1	594					
MGC04927	10	853	Platelet-activating factor acetylhydrolase IB subunit alpha	Lissencephaly-1 protein (Lis-1, PAF-AH alpha) N-terminal domain ; LISH ; PLATELET-ACTIVATING FACTOR ACETYLHYDROLASE ISOFORM 1B ALPHA SUBUNIT ; WD_REPEATS_2 ; LisH ; GPROTEINBRPT ; Q8HXX0_MACFA_Q8HXX0; ; WD40 REPEAT PROTEIN ; WD40 repeat-like ; coiled-coil ; WD_REPEATS_1 ; WD_REPEATS_REGION ; WD40		K01062_00565_Ether lipid metabolism;	2.7.11.1_Non-specificserine/threonineprotein kinase.; 2.4.1.37_Fucosylgalactoside3-alpha-galactosyltransferase.; 2.3.1.48_Histoneacetyltransferase.; 2.7.11.7_[Myosinheavy-chain]kinase.;
MGC04931	2	862	Protein phosphatase 1 regulatory subunit 12A	ANKYRIN ; Ank ; ANKYRIN REPEAT-CONTAINING ; ANK_REPEAT ; ANK_REP_REGION ; coiled-coil ; PROTEIN PHOSPHATASE 1 REGULATORY SUBUNIT 12B (MYOSIN PHOSPHATASE TARGETING SUBUNIT 2) ; Ankyrin repeat	GO:0004871_F_signal transducer activity; GO:0005515_F_protein binding;	K06270_04510_Focal adhesion; K06270_04720_Long-term potentiation; K06270_04810_Regulation of actin cytoskeleton;	2.7.11.1_Non-specificserine/threonineprotein kinase.; 2.4.2.30_NAD(+)ADP-ribosyltransferase.;
MGC04933	1	707	E3 ubiquitin-protein ligase RING2	RING/U-box ; RING FINGER PROTEIN ; ZF_RING_1 ; zf-C3HC4 ; ZF_RING_2 ; POLYCOMB-M33 INTERACTING PROTEIN RING1B			
MGC04953	3	662					
MGC04964	15	883	Pathogenesis-related protein 5	Thaumatin ; Osmotin, thaumatin-like protein ; THAUMATIN	GO:0005515_F_protein binding; GO:0031225_C_anchored to membrane;		
MGC04967	1	324					
MGC04980	1	407	Acidic mammalian chitinase	CHITINASE ; BRAIN CHITINASE AND CHIA ; Q803B7_BRARE_Q803B7; ; (Trans)glycosidases ; Glyco_hydro_18		K01183_00530_Aminosugars metabolism;	3.2.1.14_Chitinase.;
MGC04987	4	1154	Pre-mRNA-splicing factor CWC25				
MGC04988	5	608	Cdc42 homolog	P-loop containing nucleoside triphosphate hydrolases ; CELL DIVISION CONTROL PROTEIN 42 ; RASTRNSFRMNG ; RAS-RELATED GTPASE ; small_GTP: small GTP-binding protein domain ; Ras			
MGC04997	5	701	Dedicator of cytokinesis protein 7	DOCK-7,8 ; DEDICATOR OF CYTOKINESIS (DOCK)			

MGC05000	2	538	Peptidyl-prolyl cis-trans isomerase-like 1	CSA_PPIASE_1 ; Cyclophilin-like ; CSAPPISMRASE ; CYCLOPHILIN ; PEPTIDYL-PROLYL CIS-TRANS ISOMERASE-LIKE 1, PPIL1 ; Pro_isomerase ; CSA_PPIASE_2			5.2.1.8_Peptidylprolylisomerase.;
MGC05004	1	606	Protein SET	SET ; NAP ; TESTIS-SPECIFIC Y-ENCODED PROTEIN ; coiled-coil			
MGC05008	7	1646	Chromodomain-helicase-DNA-binding protein Mi-2 homolog	DUF1086 ; DUF1087 ; HELICASE_CTER ; Helicase_C ; P-loop containing nucleoside triphosphate hydrolases ; CHROMODOMAIN HELICASE DNA BINDING PROTEIN ; ATP-DEPENDENT HELICASE SMARCA (SWI/SNF-RELATED MATRIX-ASSOCIATED ACTIN-DEPENDENT REGULATOR OF CHROMATIN A)-RELATED ; coiled-coil	GO:0008134_F_transcription factor binding;	K01509_00230_Purine metabolism;	
MGC05010	1	695	Xylosyltransferase oxt	WSC ; coiled-coil			
MGC05017	6	762		C-type lectin-like			
MGC05021	1	591		C-type lectin-like			
MGC05026	5	698					
MGC05043	2	645	F-box/LRR-repeat protein 5				
MGC05047	1	522	TNF receptor-associated factor 2	RING/U-box ; TRAF domain-like ; ZF_RING_1 ; zf-C3HC4 ; ZF_RING_2 ; TNF RECEPTOR ASSOCIATED FACTOR 2 ; TNF RECEPTOR ASSOCIATED FACTOR	GO:0007165_P_signal transduction; GO:0046328_P_regulation of JNK cascade; GO:0030163_P_protein catabolic process; GO:0051023_P_regulation of immunoglobulin secretion; GO:0005515_F_protein binding; GO:0006916_P_anti-apoptosis;		
MGC05053	1	543	E-selectin	coiled-coil			
MGC05057	2	657	Probable inactive serine/threonine-protein kinase slob2	EGF/Laminin ; EGF-LIKE-DOMAIN, MULTIPLE 7, 8 ; EGF ; EGF_3 ; EGF_1 ; EGF_2			
MGC05071	3	734	Sulfated glycoprotein 1	SapB_2 ; SAP_B ; SAPOSIN-RELATED ; SapB_1 ; Saposin ; SAPOSIN			
MGC05076	1	563	Calnexin	P-domain of calnexin/calreticulin ; CALRETICULIN_REPEAT ; CALRETICULIN AND CALNEXIN ; CALRETICULIN ; CALNEXIN ; Calreticulin		K08054_04612_Antigen processing and presentation;	
MGC05079	1	486	Leucine-rich repeat-containing protein LOC400891	LEUCINE RICH REPEAT-CONTAINING ; RNI-like ; NALP (NACHT, LEUCINE RICH REPEAT AND PYRIN DOMAIN CONTAINING)-RELATED		K08727_05120_Epithelial cell signaling in Helicobacter pylori infection;	
MGC05080	3	681	Protein disabled	PID ; DISABLED 1, 2 RELATED ; DISABLED/NUMB-RELATED ADAPTOR ; PH domain-like		K06057_04330_Notch signaling pathway;	
MGC05083	1	657	Wiskott-Aldrich syndrome protein family member 3	WASP-1 ; coiled-coil		K06083_04520_Adherens junction; K05753_04520_Adherens junction; K05753_04810_Regulation of actin cytoskeleton;	
MGC05087	5	1258	Collagen alpha-3(VI) chain	VWFA ; VON WILENBRAND FACTOR RELATED ; vWA-like ; VWA ; VWFADOMAIN	GO:0007517_P_muscle development; GO:0005589_C_collagen type VI;	K06238_04510_Focal adhesion; K06238_04512_ECM-receptor interaction; K03900_04510_Focal adhesion; K03900_04512_ECM-receptor interaction; K03900_04610_Complement and coagulation cascades;	
MGC05089	2	649	Trypsin beta	TRYPSIN_HIS ; SERINE PROTEASE-RELATED ; Trypsin ; CHYMOTRYPSIN ; SERINE PROTEASE-RELATED, INSECT ; TRYPSIN_DOM ; Trypsin-like serine proteases		K01312_04080_Neuroactive ligand-receptor interaction;	3.4.21.4_Trypsin.;

MGC05090	4	878	Tumor necrosis factor receptor superfamily member 26	TNF receptor-like			
MGC05095	1	586	Na(+)/H(+) exchange regulatory cofactor NHE-RF2	PDZ ; PDZ domain-like ; PDZ DOMAIN CONTAINING PROTEIN	GO:0045177_C_apical part of cell; GO:0016324_C_apical plasma membrane; GO:0030643_P_cellular phosphate ion homeostasis; GO:0005515_F_protein binding; GO:0008022_F_protein C-terminus binding;	K05631_04530_Tight junction; K05631_05050_tba;	
MGC05096	2	981	Putative leucine-rich repeat-containing protein DDB_G0290503	STRUCTURAL MAINTENANCE OF CHROMOSOMES SMC FAMILY MEMBER ; Prefoldin ; coiled-coil			
MGC05097	1	634		C1q ; TNF-like ; C1Q			
MGC05100	2	632	Syntaxin-8	SNARE ; T_SNARE ; SYNTAXIN 8 ; coiled-coil ; SYNTAXIN	GO:0005887_C_integral to plasma membrane; GO:0031201_C_SNARE complex; GO:0006810_P_transport; GO:0005783_C_endoplasmic reticulum; GO:0005515_F_protein binding;	K08503_04130_SNARE interactions in vesicular transport; K08501_04130_SNARE interactions in vesicular transport;	
MGC05102	1	527	Transcriptional regulator ATRX	TRANSCRIPTIONAL REGULATOR ATRX (X-LINKED HELICASE II) ; P-loop containing nucleoside triphosphate hydrolases ; SNF2_N ; HELICASE_ATP_BIND_1 ; ATP-DEPENDENT HELICASE SMARCA (SWI/SNF-RELATED MATRIX-ASSOCIATED ACTIN-DEPENDENT REGULATOR OF CHROMATIN A)-RELATED		K01509_00230_Purine metabolism;	
MGC05112	7	643	Complement C1q-like protein 4	TNF-like ; coiled-coil			
MGC05118	2	1210	Integumentary mucin C.1 (Fragment)	Chitin_bind_3 ; SUBFAMILY NOT NAMED			
MGC05119	1	592	WD repeat-containing protein 3	WD_REPEATS_2 ; Q6CDF6_EEEEE_Q6CDF6; ; G-PROTEIN BETA WD-40 REPEATS CONTAINING PROTEIN ; GPROTEINBRPT ; WD40 REPEAT PROTEIN ; WD40 repeat-like ; WD_REPEATS_1 ; WD_REPEATS_REGION ; WD40	GO:0005634_C_nucleus;	K10260_04120_Ubiquitin mediated proteolysis; K01062_00565_Ether lipid metabolism;	2.7.11.1_Non-specificserine/threonineproteinkinase.; 2.3.1.48_Histoneacetyltransferase.; 2.7.11.7_[Myosinheavy-chain]kinase.;
MGC05126	1	526	Low-density lipoprotein receptor-related protein	LDL receptor-like module ; LOW DENSITY LIPOPROTEIN RECEPTOR ; LDLRA_1 ; Ldl_recept_a ; LDLRECEPTOR ; LDLRA_2 ; LOW-DENSITY LIPOPROTEIN RECEPTOR (LDL)		K03068_04310_Wnt signaling pathway; K04550_05010_Alzheimers disease; K06233_04340_Hedgehog signaling pathway;	3.4.21.109_Matriptase.;
MGC05130	5	654	Neurocan core protein	C-type lectin-like ; C_TYPE_LECTIN_1 ; C-TYPE LECTIN SUPERFAMILY MEMBER ; Lectin_C ; CD209 ANTIGEN (DENDRITIC CELL-SPECIFIC ICAM-3-GRABBING NONINTEGRIN 1) (DC-SIGN1) ; ANTIFREEZEII ; C_TYPE_LECTIN_2			
MGC05132	2	581		C-type lectin-like			
MGC05134	2	716	Fibrinogen-like protein A	FIBRINOGEN AND FIBRONECTIN ; Fibrinogen C-terminal domain-like ; Fibrinogen_C		K06252_04510_Focal adhesion; K06252_04512_ECM-receptor interaction;	
MGC05135	1	483					
MGC05136	2	537	Oxidation resistance protein 1	NUCLEOLAR PROTEIN 7/ESTROGEN RECEPTOR COACTIVATOR-RELATED ; LysM ; LysM domain ; NUCLEOLAR PROTEIN C7B	GO:0005730_C_nucleolus;		3.5.1.28_N-acetylmuramoyl-L-alanineamidase.;
MGC05141	4	795	Protocadherin-9	Cadherin-like ; CADHERIN_2 ; Cadherin ; CADHERIN ; CADHERIN_1			

MGC05142	2	756	Transforming growth factor-beta-induced protein ig-h3	FAS1 ; PERIOSTIN-RELATED ; PERIOSTIN (PN) (OSTEOBLAST-SPECIFIC FACTOR 2) (OSF-2) ; Fasciclin ; FAS1 domain			
MGC05147	2	763	DDB1- and CUL4-associated factor 17				
MGC05148	2	579	Protein disulfide-isomerase	Protein disulphide-isomerase ; Thioredoxin ; pdi_dom: protein disulfide-isomerase domain ; PROTEIN DISULFIDE ISOMERASE ; Thioredoxin-like ; THIOREDOXIN	GO:0005783_C_endoplasmic reticulum; GO:0005811_C_lipid particle;		5.3.4.1_Proteindisulfide-isomerase.;
MGC05157	3	980	Abnormal pharyngeal pumping eat-20	EGF/Laminin ; EGF-LIKE DOMAIN PROTEIN ; EGF ; EGF_3 ; EGF_1 ; EGF_2 ; NEUROGENIC LOCUS DELTA PROTEIN		K02599_04320_Dorso-ventral axis formation; K02599_04330_Notch signaling pathway;	
MGC05167	2	678	ADP,ATP carrier protein 2	ADP,ATP CARRIER PROTEIN ; SOLCAR ; ADPTRNSLCASE ; MITOCARRIER ; MITOCHONDRIAL CARRIER PROTEIN RELATED ; CARBOXYPEPT_ZN_2 ; Mitochondrial carrier ; Mito_carr		K05863_04020_Calcium signaling pathway;	
MGC05169	2	701	Proprotein convertase subtilisin/kexin type 5	Growth factor receptor domain			
MGC05171	1	459					
MGC05173	3	702	Microfibril-associated glycoprotein 4	FIBRINOGEN AND FIBRONECTIN ; Fibrinogen C-terminal domain-like ; Fibrinogen_C	GO:0001527_C_microfibril; GO:0007155_P_cell adhesion;		
MGC05175	1	457	CD9 antigen	TM4_1 ; TMFOUR ; Tetraspannin			
MGC05184	1	543	Serine/threonine-protein kinase PLK4	Protein kinase-like (PK-like) ; Pkinase ; POLO-LIKE KINASE 4 ; CALCIUM/CALMODULIN-DEPENDENT PROTEIN KINASE-RELATED ; PLK4_MOUSE_Q64702; ; PROTEIN_KINASE_DOM		K06631_04110_Cell cycle; K06631_04914_Progesterone-mediated oocyte maturation;	2.7.11.21_Polokinase.;
MGC05191	6	783	Protein LITAF homolog	LIPOPOLYSACCHARIDE-INDUCED TRANSCRIPTION FACTOR REGULATING TUMOR NECROSIS FACTOR ALPHA			
MGC05207	4	647	Collectin-12	C-type lectin-like ; C_TYPE_LECTIN_1 ; C-TYPE LECTIN SUPERFAMILY MEMBER ; Lectin_C ; GALACTOSE-SPECIFIC C-TYPE LECTIN ; coiled-coil ; C_TYPE_LECTIN_2	GO:0005515_F_protein binding;		
MGC05208	1	586	Collagen alpha-2(VIII) chain	C1q ; COLLAGEN ALPHA 1(VIII) CHAIN ; COLLAGEN ALPHA CHAIN ; COMPLEMNTC1Q ; TNF-like ; C1Q			
MGC05209	1	571	Fibrinogen C domain-containing protein 1-B	FIBRINOGEN AND FIBRONECTIN ; Fibrinogen C-terminal domain-like ; Fibrinogen_C			
MGC05213	1	500		coiled-coil			
MGC05220	1	564	Low affinity immunoglobulin epsilon Fc receptor	REGENERATING GENE TYPE IV-RELATED ; C-type lectin-like ; LITHOSTATHINE ; Lectin_C ; coiled-coil ; C_TYPE_LECTIN_2		K06468_04640_Hematopoietic cell lineage;	
MGC05228	1	644	Phospholipase A-2-activating protein	WD_REPEATS_2 ; GPROTEINBRPT ; WD40 repeat-like ; PHOSPHOLIPASE A-2-ACTIVATING PROTEIN ; Q8C6C4_MOUSE_Q8C6C4; ; WD_REPEATS_REGION ; WD40	GO:0007165_P_signal transduction; GO:0016005_F_phospholipase A2 activator activity; GO:0006954_P_inflammatory response; GO:0005515_F_protein binding; GO:0006644_P_phospholipid metabolic process;	K01062_00565_Ether lipid metabolism;	2.7.11.1_Non-specificserine/threonineproteinkinase.; 2.4.1.37_Fucosylgalactoside3-alpha-galactosyltransferase.; 2.3.1.48_Histoneacetyltransferase.; 2.7.11.7_[Myosinheavy-chain]kinase.;
MGC05238	2	654	Stress response protein NST1	coiled-coil			

MGC05239	1	533	C-type lectin domain family 4 member M	C-type lectin-like ; C_TYPE_LECTIN_1 ; C-TYPE LECTIN SUPERFAMILY MEMBER ; Lectin_C ; CD209 ANTIGEN (DENDRITIC CELL-SPECIFIC ICAM-3-GRABBING NONINTEGRIN 1) (DC-SIGN1) ; C_TYPE_LECTIN_2			
MGC05241	1	578	CAP-Gly domain-containing linker protein 1	Cap-Gly domain ; CAP_GLY ; RESTIN (CYTOPLASMIC LINKER PROTEIN-170) (CLIP-170) ; CAP_GLY_2 ; coiled-coil ; DYNACTIN 1-RELATED MICROTUBULE-BINDING			
MGC05242	9	1343	Cathepsin C	Cysteine proteinases ; Dipeptidyl peptidase I (cathepsin C), exclusion domain ; THIOL_PROTEASE_CYS ; CATHEPSIN C ; Peptidase_C1 ; PAPAIN ; Q8BQL3_MOUSE_Q8BQL3; ; THIOL_PROTEASE_HIS ; CathepsinC_exc ; CYSTEINE PROTEASE FAMILY C1-RELATED ; THIOL_PROTEASE_ASN	GO:0005764_C_lysosome; GO:0006955_P_immune response;	K01363_04612_Antigen processing and presentation;	3.4.14.1_Dipeptidyl-peptidaseI.;
MGC05254	1	539	SNF-related serine/threonine-protein kinase	Protein kinase-like (PK-like) ; PROTEIN_KINASE_ATP ; Pkinase ; SNF-1 RELATED KINASE (SNRK) ; CALCIUM/CALMODULIN-DEPENDENT PROTEIN KINASE-RELATED ; Q6IQ46_HUMAN_Q6IQ46; ; PROTEIN_KINASE_DOM			2.7.11.1_Non-specificserine/threonineproteinkinase.;
MGC05264	1	548	Protein NEL	CYR61/CCN1 ; Fibronectin type I module ; VWFC_2 ; CONNECTIVE TISSUE GROWTH FACTOR-RELATED ; VWFC_1			
MGC05265	2	676	WNT1-inducible-signaling pathway protein 1	CYR61/CCN1 ; ShK ; VWFC_2 ; CONNECTIVE TISSUE GROWTH FACTOR-RELATED ; VWFC_1			
MGC05266	2	729	Cardioacceleratory peptide receptor	G_PROTEIN_RECEP_F1_2 ; G-PROTEIN COUPLED RECEPTOR ; GPCRRHODOPSN ; 7tm_1 ; Family A G protein-coupled receptor-like		K04226_04020_Calcium signaling pathway; K04226_04080_Neuroactive ligand-receptor interaction;	
MGC05268	2	607	Calmodulin	EF_HAND_1 ; CALMODULIN ; RECOVERIN ; EF_HAND_2 ; EF-hand ; Q7R9F4_PLAYO_Q7R9F4; ; CALCIUM BINDING PROTEIN ; efhand			2.7.11.1_Non-specificserine/threonineproteinkinase.;
MGC05275	1	545	SH3 domain-containing protein C23A1.17				
MGC05285	1	584	Perlucin	C-type lectin-like ; ASIALOGLYCOPROTEIN RECEPTOR ; C_TYPE_LECTIN_1 ; C-TYPE LECTIN SUPERFAMILY MEMBER ; Lectin_C ; ANTIFREEZEII ; C_TYPE_LECTIN_2			
MGC05290	1	641	RING finger protein ETP1				
MGC05292	7	817	Proteasome subunit beta type-4	N-terminal nucleophile aminohydrolases (Ntn hydrolases) ; Proteasome ; PROTEASOME_B ; PROTEASOME SUBUNIT BETA TYPE 4 ; PROTEASOME SUBUNIT ALPHA/BETA		K02736_03050_Proteasome;	3.4.25.1_Proteasomeendopeptidasecomplex.;
MGC05294	1	565	Mammalian ependymin-related protein 1	EPENDYMIN			
MGC05297	1	597	Tetratricopeptide repeat protein 32	TPR_1 ; TPR ; TPR-like ; TPR_REGION			3.1.3.16_Phosphoproteinphosphatase.;
MGC05302	6	752	RNA polymerase sigma-E factor	RseA_N ; N-terminal, cytoplasmic domain of anti-sigmaE factor RseA			
MGC05305	6	704	Ubiquitin-conjugating enzyme E2-17 kDa	UBIQUITIN_CONJUGAT_2 ; UBIQUITIN-CONJUGATING ENZYME E2 ; UBC-like ; Q6PPH5_EEEEE_Q6PPH5; ; UQ_con ; UBIQUITIN_CONJUGAT_1	GO:0005515_F_protein binding;	K06689_04120_Ubiquitin mediated proteolysis;	6.3.2.19_Ubiquitin--proteinligase.;

MGC05307	1	653	Peroxinectin A	PROSTAGLANDIN G/H SYNTHASE ; Heme-dependent peroxidases ; PEROXIDASE_3 ; An_peroxidase ; FEEBLY-LIKE PROTEIN			
MGC05308	4	775	GTPase IMAP family member 4	AIG1 ; P-loop containing nucleoside triphosphate hydrolases ; coiled-coil			
MGC05314	1	655	Putative ankyrin repeat protein MM_0045	ANK REPEAT-CONTAINING ; ANKYRIN ; Ank ; ANKYRIN REPEAT-CONTAINING ; ANK_REPEAT ; ANK_REP_REGION ; Ankyrin repeat	GO:0048675_P_axon extension;	K08803_05219_Bladder cancer;	2.7.11.1_Non-specificserine/threonineprotein kinase.;
MGC05315	1	626	Mucosa-associated lymphoid tissue lymphoma translocation protein 1 homolog	Caspase-like ; PARACASPASE ; MUCOSA ASSOCIATED LYMPHOID TISSUE LYMPHOMA TRANSLOCATION PROTEIN 1/PARACASPASE		K07369_04660_T cell receptor signaling pathway; K07369_04662_B cell receptor signaling pathway;	
MGC05317	5	596	Phosphatidylethanolamine-binding protein 4	PHOSPHATIDYLETHANOLAMINE-BINDING PROTEIN ; PBP ; PEBP-like ; PROKAR_LIPOPROTEIN	GO:0048572_P_short-day photoperiodism; GO:0048510_P_regulation of timing of transition from vegetative to reproductive phase; GO:0010229_P_inflorescence development;		
MGC05318	1	612	Kinase D-interacting substrate of 220 kDa	ANK REPEAT-CONTAINING ; ANKYRIN ; Ank ; ANKYRIN REPEAT-CONTAINING ; ANK_REPEAT ; ANK_REP_REGION ; DNA_POLYMERASE_B ; Ankyrin repeat	GO:0019901_F_protein kinase binding; GO:0019887_F_protein kinase regulator activity;		2.7.11.1_Non-specificserine/threonineprotein kinase.; 2.4.2.30_NAD(+)ADP-ribosyltransferase.; 2.1.1.43_Histone-lysineN-methyltransferase.;
MGC05320	6	752	Protein Red	RED PROTEIN (IK FACTOR) (CYTOKINE IK) ; RED_N ; coiled-coil	GO:0005625_C_soluble fraction; GO:0007267_P_cell-cell signaling; GO:0005615_C_extracellular space; GO:0006955_P_immune response;		
MGC05336	2	635	Hsc70-interacting protein	TPR_1 ; TPR ; HSC70-INTERACTING PROTEIN ; TPR-like ; TPR REPEAT CONTAINING PROTEIN ; TPR_REGION			
MGC05350	1	629	Interleukin enhancer-binding factor 2	INTERLEUKIN ENHANCER BINDING FACTOR 2 ; 25A_SYNTH_3 ; EUKARYOTE SPECIFIC DSRNA BINDING PROTEIN ; Nucleotidyltransferase ; DZF			
MGC05361	1	593	Lachesin	Immunoglobulin ; IG_LIKE ; ig ; NEUROTRACTING/LSAMP/NEUROTRIMIN/OBCAM RELATED CELL ADHESION MOLECULE ; LACHESIN	GO:0005515_F_protein binding;	K06775_04514_Cell adhesion molecules (CAMs);	2.7.10.1_Receptorprotein-tyrosinekinase.; 1.11.1.7_Peroxidase.;
MGC05362	1	683	Uncharacterized protein PF11_0207	coiled-coil			
MGC05365	1	580	Collectin-12	C-type lectin-like ; C-TYPE LECTIN SUPERFAMILY MEMBER ; Lectin_C ; LOW AFFINITY IMMUNOGLOBULIN EPSILON FC RECEPTOR (CD23 ANTIGEN) ; ANTIFREEZEII ; C_TYPE_LECTIN_2			
MGC05366	1	595	Sorting nexin-2	SORTING NEXIN 2 ; SORTING NEXIN ; PX ; PX domain			
MGC05373	1	659	Titin	MYOSIN-BINDING PROTEIN-RELATED ; Fibronectin type III ; FN3 ; TITIN ; fn3 ; FNTYPEIII	GO:0040011_P_locomotion;	K06765_04360_Axon guidance; K06765_05210_Colorectal cancer; K06766_04514_Cell adhesion molecules (CAMs); K05695_04514_Cell adhesion molecules (CAMs); K05695_04520_Adherens junction; K05695_04910_Insulin signaling pathway;	2.7.11.1_Non-specificserine/threonineprotein kinase.; 3.1.3.48_Protein-tyrosine-phosphatase.;

MGC05376	2	678		Aerolisin/ETX pore-forming domain	GO:0040026_P_positive regulation of vulval development; GO:0005515_F_protein binding; GO:0008219_P_cell death;		
MGC05380	3	621	Collagen alpha-2(VIII) chain	C1q ; GLIACOLIN-RELATED ; CEREBELLIN-RELATED ; COMPLEMNTC1Q ; TNF-like ; C1Q			
MGC05381	2	605	PHD finger and CXXC domain-containing protein CG17446	FYVE/PHD zinc finger ; ZF_PHD_2 ; ZF_PHD_1 ; PHD ; CPG BINDING PROTEIN ; ZF_CXXC ; zf-CXXC	GO:0001568_P_blood vessel development; GO:0032776_P_DNA methylation on cytosine; GO:0030097_P_hemopoiesis;		1.14.11.27_[HistoneH3]-lysine-36demethylase.; 2.1.1.37_DNA(cytosine-5-)-methyltransferase.;
MGC05384	3	580	Caprin-2	TNF-like			
MGC05386	3	705					
MGC05393	1	618		Thymosin ; TETRA THYMOSIN			
MGC05394	1	585	Myosin heavy chain, striated muscle	MYOSIN HEAVY CHAIN, SKELETAL MUSCLE OR CARDIAC MUSCLE ; MYOSIN ; Spectrin repeat ; Prefoldin ; coiled-coil ; Myosin_tail_1		K10352_04530_Tight junction;	
MGC05399	3	681	Insulin receptor	INSULIN RECEPTOR ; Recep_L_domain ; Fibronectin type III ; L domain-like ; TYROSINE PROTEIN KINASE			2.7.10.1_Receptorprotein-tyrosinekinase.;
MGC05400	1	425	Stress-activated protein kinase JNK	Protein kinase-like (PK-like) ; Pkinase ; Q7Z085_APLCA_Q7Z085; ; CDC2, MAP KINASE-RELATED ; JNK ; PROTEIN_KINASE_DOM			2.7.11.24_Mitogen-activatedproteinkinase.;
MGC05402	1	612	Perlucin	C-type lectin-like ; ASIALOGLYCOPROTEIN RECEPTOR ; C_TYPE_LECTIN_1 ; C-TYPE LECTIN SUPERFAMILY MEMBER ; Lectin_C ; C_TYPE_LECTIN_2			
MGC05403	2	731	HEAT repeat-containing protein 3	ARM repeat			
MGC05412	4	550	Complement C1q-like protein 2	C1q ; C1Q-RELATED FACTOR ; COLLAGEN ALPHA CHAIN ; COMPLEMNTC1Q ; TNF-like ; C1Q	GO:0005515_F_protein binding;		
MGC05413	4	754	Drebrin-like protein	CORTACTIN ; Actin depolymerizing proteins ; DREBRIN-RELATED ; coiled-coil ; Cofilin_ADF			
MGC05420	2	684	IgLON family member 5	MYB_1			
MGC05421	2	645					
MGC05428	2	528	Thyroglobulin	INSULIN-LIKE GROWTH FACTOR BINDING PROTEIN ; Thyroglobulin_1 ; Thyroglobulin type-1 domain ; INSULIN-LIKE GROWTH FACTOR BINDING PROTEIN 3 ; THYROGLOBULIN_1_2	GO:0007165_P_signal transduction; GO:0005576_C_extracellular region;	K10809_05320_Autoimmune thyroid disease;	
MGC05436	2	969	Lipopolysaccharide-induced tumor necrosis factor-alpha factor homolog	LIPOPOLYSACCHARIDE-INDUCED TRANSCRIPTION FACTOR REGULATING TUMOR NECROSIS FACTOR ALPHA			
MGC05446	1	562	Chitinase domain-containing protein 1	CHITINASE ; (Trans)glycosidases			
MGC05448	1	688	Protein transport protein sec31				
MGC05453	1	575	Cyclin-dependent kinase-like 2	Protein kinase-like (PK-like) ; KKIAMRE ; PROTEIN_KINASE_ST ; Pkinase ; Q6TXH3_RAT_Q6TXH3; ; CDC2, MAP KINASE-RELATED ; PROTEIN_KINASE_DOM			2.7.11.22_Cyclin-dependentkinase.;
MGC05469	4	1058	Adenosine deaminase	ADENOSINE DEAMINASE ; Metallo-dependent hydrolases ; aden_deam: adenosine deaminase ; A_DEAMINASE ; A_deaminase		K01488_00230_Purine metabolism;	3.5.4.4_Adenosinedeaminase.;

MGC05472	1	649	Cathepsin S	Cysteine proteinases ; THIOL_PROTEASE_CYS ; Inhibitor_I29 ; Peptidase_C1 ; Q8MNZ7_EEEEE_Q8MNZ7; ; CYSTEINE PROTEASE FAMILY C1-RELATED ; CATHEPSIN L		K01368_04612_Antigen processing and presentation;	3.4.22.27_CathepsinS.; 3.4.22.15_CathepsinL.;
MGC05476	11	786	Collagen alpha-1(XII) chain	VWFA ; INTEGRIN ALPHA-RELATED ; VON WILENBRAND FACTOR RELATED ; vWA-like ; VWA ; VWFADOMAIN	GO:0030199_P_collagen fibril organization; GO:0030020_F_extracellular matrix structural constituent conferring tensile strength; GO:0001501_P_skeletal development; GO:0005595_C_collagen type XII;	K06238_04510_Focal adhesion; K06238_04512_ECM-receptor interaction;	3.4.21.43_Classical-complement-pathwayC3/C5convertase.;
MGC05480	1	677	Tyrosine-protein phosphatase non-receptor type 11	PROTEIN TYROSINE PHOSPHATASE N11 (SHP2) ; PROTEIN-TYROSINE PHOSPHATASE ; TYR_PHOSPHATASE_PTP ; PRTYPHPHTASE ; SH2 domain ; (Phosphotyrosine protein) phosphatases II ; Y_phosphatase ; SH2 ; Q6GP31_XENLA_Q6GP31;	GO:0005515_F_protein binding;		3.1.3.48_Protein-tyrosine-phosphatase.;
MGC05492	1	629	Neurogenic locus notch homolog protein 1	EGF/Laminin			
MGC05497	1	571	Lectoxin-Lio2	REGENERATING GENE TYPE IV-RELATED ; C-type lectin-like ; LITHOSTATHINE ; Lectin_C ; C_TYPE_LECTIN_2 ; PROKAR_LIPOPROTEIN		K06468_04640_Hematopoietic cell lineage;	
MGC05498	2	764		TNF-like			
MGC05509	3	793	Complement C1q-like protein 4	C1q ; GLIACOLIN-RELATED ; CEREBELLIN-RELATED ; TNF-like ; coiled-coil			
MGC05514	1	545	Delta-like protein A				
MGC05515	2	796	Band 4.1-like protein 3	4.1 G PROTEIN ; FERM_3 ; FA ; coiled-coil ; PH domain-like		K06107_04530_Tight junction;	3.1.3.48_Protein-tyrosine-phosphatase.;
MGC05517	2	629	Sushi, nidogen and EGF-like domain-containing protein 1	EGF/Laminin ; EGF-LIKE DOMAIN PROTEIN ; EGF ; EGF_3 ; EGF_1 ; SNED1 PROTEIN ; EGF_2		K02599_04320_Dorso-ventral axis formation; K02599_04330_Notch signaling pathway; K06255_04512_ECM-receptor interaction;	
MGC05521	3	396	Serine protease inhibitor Cvsi-2	EGF_1			
MGC05527	1	612	Complement C1q subcomponent subunit A	C1q ; COLLAGEN ALPHA 1(VIII) CHAIN ; COLLAGEN ALPHA CHAIN ; COMPLEMNTC1Q ; TNF-like ; C1Q		K03986_04610_Complement and coagulation cascades; K03986_05010_Alzheimers disease; K03988_04610_Complement and coagulation cascades; K03988_05010_Alzheimers disease;	
MGC05528	1	660	Ras association domain-containing protein 1	gb def: Hypothetical protein BE0003N10.2 ; Cysteine-rich domain ; CHIMERIN-RELATED RHO- GTPASE-ACTIVATING PROTEIN ; ZF_DAG_PE_2 ; C1_1 ; DAGPEDOMAIN			2.7.11.13_ProteinkinaseC.;
MGC05532	1	567	Perlucin	C-type lectin-like ; C_TYPE_LECTIN_1 ; C-TYPE LECTIN SUPERFAMILY MEMBER ; Lectin_C ; GALACTOSE-SPECIFIC C-TYPE LECTIN ; ANTIFREEZEII ; C_TYPE_LECTIN_2			
MGC05535	1	546	Complement C1q tumor necrosis factor-related protein 3	C1q ; GLIACOLIN-RELATED ; CEREBELLIN-RELATED ; COMPLEMNTC1Q ; TNF-like ; C1Q			
MGC05545	5	600	Heavy metal-binding protein HIP	C1q ; CEREBELLIN-RELATED ; COMPLEMNTC1Q ; TNF-like ; coiled-coil ; C1Q			
MGC05549	1	577	Big defensin				
MGC05550	1	576	Aggrecan core protein	C-type lectin-like ; ASIALOGLYCOPROTEIN RECEPTOR ; C-TYPE LECTIN SUPERFAMILY MEMBER ; coiled-coil			

MGC05579	1	657	Complement C1q tumor necrosis factor-related protein 4	C1q ; COLLAGEN ALPHA 1(VIII) CHAIN ; COLLAGEN ALPHA CHAIN ; COMPLEMNTC1Q ; TNF-like ; C1Q		K03986_04610_Complement and coagulation cascades; K03986_05010_Alzheimers disease;	
MGC05586	1	622	Filamin-C	FILAMIN ; SPECTRIN-LIKE CELL STRUCTURE PROTEIN ; E set domains ; FILAMIN_REPEAT ; Filamin		K04437_04010_MAPK signaling pathway; K04437_04510_Focal adhesion;	
MGC05594	5	1190	Radial spoke head 1 homolog	MORN ; PHOSPHATIDYLINOSITOL-4-PHOSPHATE 5-KINASE RELATED ; MORN PROTEIN ; Histone H3 K4-specific methyltransferase SET7/9 N-terminal domain ; coiled-coil	GO:0005737_C_cytoplasm; GO:0005634_C_nucleus;	K04575_05030_tba; K00889_00562_Inositol phosphate metabolism; K00889_04070_Phosphatidylinositol signaling system; K00889_04810_Regulation of actin cytoskeleton;	2.7.1.68_1-phosphatidylinositol-4-phosphate5-kinase.;
MGC05603	2	668	Elongation factor 1-alpha	TRANSLATION FACTOR ; P-loop containing nucleoside triphosphate hydrolases ; ELONGATNFCT ; ELONGATION FACTOR 1-ALPHA (EF-1-ALPHA) ; EFACTOR_GTP ; GTP_EFTU	GO:0005515_F_protein binding; GO:0005811_C_lipid particle;		2.7.7.4_Sulfateadenylyltransferase.;
MGC05604	3	803		C1q ; CEREBELLIN-RELATED ; CEREBELLIN 3 ; TNF-like ; C1Q			
MGC05606	1	605	PAB-dependent poly(A)-specific ribonuclease subunit 2	UBIQUITIN SPECIFIC PROTEASE 52 / POLY(A) RIBONUCLEASE SUBUNIT PAN2 ; Exonuc_X-T ; Ribonuclease H-like			3.1.13.4_Poly(A)-specificribonuclease.;
MGC05609	3	704		Leech antihemostatic proteins			
MGC05610	1	551		CEREBELLIN-RELATED ; TNF-like ; coiled-coil ; CEREBELLIN 4			
MGC05612	1	610	Harmonin	PDZK7 ; PDZ ; PDZ DOMAIN CONTAINING WHIRLIN AND HARMONIN-RELATED ; PDZ domain-like			
MGC05614	1	517	Transcription factor p65	C-REL PROTO-ONCOGENE ; REL_2 ; ANKYRIN REPEAT-CONTAINING ; p53-like transcription factors ; REL_1 ; RHD			
MGC05619	2	704	Calmodulin	EF_HAND_1 ; CALMODULIN ; EF_HAND_2 ; Q76LB7_STRIE_Q76LB7; ; EF-hand ; CALCIUM BINDING PROTEIN ; efhand			2.7.11.1_Non-specificserine/threonineproteinkinase.;
MGC05624	1	533	Hepatic lectin	C-type lectin-like ; C_TYPE_LECTIN_1 ; C-TYPE LECTIN SUPERFAMILY MEMBER ; Lectin_C ; GALACTOSE-SPECIFIC C-TYPE LECTIN ; coiled-coil ; ANTIFREEZEII ; C_TYPE_LECTIN_2			
MGC05635	1	602	Src substrate cortactin	CORTACTIN ; HS1_rep ; SRC SUBSTRATE CORTACTIN		K06106_04530_Tight junction;	
MGC05638	1	665	Synaptotagmin-15	SYNAPTOTAGMIN-15 ; C2DOMAIN ; SYNAPTOTAGMIN ; C2 domain (Calcium/lipid-binding domain, CaLB) ; SYNAPTOTAGMN ; C2	GO:0000299_C_integral to membrane of membrane fraction;		2.7.1.154_Phosphatidylinositol-4-phosphate3-kinase.;
MGC05642	1	726	Probable global transcription activator SNF2L2	HSA ; SMARCA4 ; ATP-DEPENDENT HELICASE SMARCA (SWI/SNF-RELATED MATRIX-ASSOCIATED ACTIN-DEPENDENT REGULATOR OF CHROMATIN A)-RELATED ; coiled-coil			
MGC05646	1	624	Slit homolog 1 protein	LRR_1 ; LRRNT ; SLIT ; L domain-like ; LEURICHRPT ; LEUCINE-RICH TRANSMEMBRANE PROTEINS		K06838_04360_Axon guidance; K06850_04360_Axon guidance;	1.11.1.7_Peroxidase.;
MGC05647	1	652		ShK			

MGC05648	1	544	Plasminogen	KRINGLE_1 ; Q6PBA6_BRARE_Q6PBA6; ; SERINE PROTEASE-RELATED ; Kringle-like ; KRINGLE_2 ; KRINGLE ; PROTHROMBIN (COAGULATION FACTOR II) ; Kringle		K05460_04060_Cytokine-cytokine receptor interaction; K05460_04510_Focal adhesion; K05460_05211_Renal cell carcinoma; K05460_05218_Melanoma; K01315_04080_Neuroactive ligand-receptor interaction; K01315_04610_Complement and coagulation cascades;	2.7.10.1_Receptorprotein-tyrosinekinase.; 3.4.21.7_Plasmin.;
MGC05649	1	590	Proteasomal ubiquitin receptor ADRM1	ARM_1 ; ADHESION REGULATING MOLECULE 1 (110 KDA CELL MEMBRANE GLYCOPROTEIN)	GO:0000502_C_proteasome complex; GO:0043248_P_proteasome assembly; GO:0008538_F_proteasome activator activity;		
MGC05650	2	560	Leukocyte surface antigen CD53	LEUKOCYTE SURFACE ANTIGEN CD53 ; TETRASPANIN ; Tetraspannin			
MGC05656	3	708	Hepatic lectin	C-type lectin-like ; C-TYPE LECTIN SUPERFAMILY MEMBER ; Lectin_C ; CD209 ANTIGEN (DENDRITIC CELL-SPECIFIC ICAM-3-GRABBING NONINTEGRIN 1) (DC-SIGN1) ; Q8RQ77_STRPN_Q8RQ77; ; C_TYPE_LECTIN_2			
MGC05676	3	619	Mammalian ependymin-related protein 1				
MGC05677	6	938	Fibrinogen C domain-containing protein 1	FIBRINOGEN AND FIBRONECTIN ; Fibrinogen C-terminal domain-like ; Fibrinogen_C ; FIBRIN_AG_C_DOMAIN		K06252_04510_Focal adhesion; K06252_04512_ECM-receptor interaction;	
MGC05691	1	180		PDZ ; PDZ domain-like			
MGC05711	1	606	Heat shock protein HSP 90-beta	HEAT SHOCK PROTEIN 90 ; HEATSHOCK90 ; HATPase_c ; HSP90 ; ATPase domain of HSP90 chaperone/DNA topoisomerase II/histidine kinase	GO:0005515_F_protein binding;	K04079_04612_Antigen processing and presentation; K04079_04914_Progesterone-mediated oocyte maturation; K04079_05215_Prostate cancer;	
MGC05729	1	649	BTB/POZ domain-containing protein 17	POZ domain ; KELCH-RELATED PROTEIN ; BTB ; BACK			
MGC05730	4	884	Tyrosine-protein phosphatase non-receptor type 1	PROTEIN-TYROSINE PHOSPHATASE ; TYR_PHOSPHATASE_2 ; TYR_PHOSPHATASE_PTP ; TYR_PHOSPHATASE_1 ; PRTYPHPHTASE ; PROTEIN TYROSINE PHOSPHATASE, NON-RECEPTOR TYPE NT1 ; (Phosphotyrosine protein) phosphatases II ; Y_phosphatase	GO:0007165_P_signal transduction; GO:0004725_F_protein tyrosine phosphatase activity; GO:0005515_F_protein binding;	K05696_04520_Adherens junction; K05696_04910_Insulin signaling pathway;	3.1.3.48_Protein-tyrosine-phosphatase.;
MGC05735	1	476		C1q ; CEREBELLIN-RELATED ; COMPLEMNTC1Q ; TNF-like ; coiled-coil ; C1Q			
MGC05741	1	635	Coronin-7	DUF1900 ; CORONIN ; WD40 repeat-like	GO:0005625_C_soluble fraction; GO:0000299_C_integral to membrane of membrane fraction; GO:0005794_C_Golgi apparatus;		
MGC05747	1	470					
MGC05748	1	498	Complement C3	A2M_recep ; Alpha-macroglobulin receptor domain ; MACROGLOBULIN/COMPLEMENT		K03990_04610_Complement and coagulation cascades; K03994_04610_Complement and coagulation cascades;	
MGC05753	2	664	Caspase-3	Caspase-like ; IL1BCENZYME ; CASPASE_P20 ; Peptidase_C14 ; CASPASE RELATED			3.4.22.56_Caspase-3.;
MGC05754	3	793	Calmodulin	EF-Hand_type ; EF_HAND_1 ; RECOVERIN ; PTHR23050 ; EF_HAND_2 ; SSF47473 ; EFh ; EF-hand ; PTHR23050:SF20 ; efhand			2.7.11.1_Non-specificserine/threonineproteinkinase.;

MGC05779	1	629		beta-Roll ; PROKAR_LIPOPROTEIN			
MGC05785	1	620	Integrin beta-1-binding protein 1	PID ; PH domain-like			
MGC05793	1	592	Caspase-7	Caspase-like ; IL1BCENZYME ; CASPASE_P20 ; Peptidase_C14 ; CASPASE RELATED	GO:0043065_P_positive regulation of apoptosis;	K04396_04210_Apoptosis; K04396_05040_tba; K04397_04210_Apoptosis; K04397_05010_Alzheimers disease; K04397_05050_tba;	3.4.22.59_Caspase-6.; 3.4.22.57_Caspase-4.; 3.4.22.58_Caspase-5.; 3.4.22.60_Caspase-7.;
MGC05807	1	598	Probable G-protein coupled receptor 157	G_PROTEIN_RECEP_F2_4 ; Family A G protein-coupled receptor-like ; G PROTEIN-COUPLED RECEPTOR 157-RELATED			
MGC05815	1	289	Proline-serine-threonine phosphatase-interacting protein 1	PROLINE-SERINE-THREONINE PHOSPHATASE INTERACTING PROTEIN 1 ; FCH	GO:0019903_F_protein phosphatase binding; GO:0003779_F_actin binding; GO:0005826_C_contractile ring; GO:0005515_F_protein binding; GO:0000910_P_cytokinesis; GO:0001725_C_stress fiber;		
MGC05817	1	508	Cell division protein kinase 9	Protein kinase-like (PK-like) ; Pkinase ; Q71FK1_EEEEE_Q71FK1; ; CDC2, MAP KINASE-RELATED ; CDK9 ; PROTEIN_KINASE_DOM			2.7.11.23_[RNA-polymerase]-subunitkinase.; 2.7.11.22_Cyclin-dependentkinase.;
MGC05818	1	654	Leucine-rich repeat-containing protein 1	LEUCINE-RICH REPEAT CONTAINING PROTEIN ; LRR_1 ; LEUCINE-RICH REPEAT-CONTAINING PROTEIN ; L domain-like ; LEURICHRPT		K04424_04010_MAPK signaling pathway; K04424_04530_Tight junction; K01768_00230_Purine metabolism; K10130_04115_p53 signaling pathway;	3.1.3.16_Phosphoproteinphosphatase.; 3.1.3.48_Protein-tyrosine-phosphatase.; 3.1.13.4_Poly(A)-specificribonuclease.;
MGC05819	1	610	Calreticulin	P-domain of calnexin/calreticulin ; CALRETICULIN AND CALNEXIN ; CALRETICULIN ; Calreticulin		K08057_04612_Antigen processing and presentation;	
MGC05820	1	643	Ankyrin repeat domain-containing protein 17	ANK REPEAT-CONTAINING ; ANKYRIN ; Ank ; ANKYRIN REPEAT-CONTAINING ; ANK_REPEAT ; ANK_REP_REGION ; Ankyrin repeat	GO:0007492_P_endoderm development;		2.7.11.1_Non-specificserine/threonineproteinkinase.; 2.4.2.30_NAD(+)ADP-ribosyltransferase.;
MGC05831	6	646	Collagen alpha-1(VIII) chain	C1q ; COLLAGEN ALPHA 1(VIII) CHAIN ; COLLAGEN ALPHA CHAIN ; COMPLEMNTC1Q ; TNF-like ; C1Q			
MGC05838	1	570					
MGC05839	1	629	Nucleolar protein 58	coiled-coil			
MGC05845	1	579	Peptidyl-prolyl cis-trans isomerase B	PEPTIDYL-PROLYL CIS-TRANS ISOMERASE B, PPIB ; Cyclophilin-like ; CSAPPISMRASE ; CYCLOPHILIN ; Pro_isomerase ; CSA_PPIASE_2	GO:0051082_F_unfolded protein binding; GO:0005783_C_endoplasmic reticulum; GO:0005515_F_protein binding; GO:0005788_C_endoplasmic reticulum lumen; GO:0003755_F_peptidyl-prolyl cis-trans isomerase activity;		5.2.1.8_Peptidylprolylisomerase.;
MGC05850	1	572					
MGC05853	1	552					
MGC05854	3	740	Splicing factor 3A subunit 2	ZF_MATRIN ; C2H2 and C2HC zinc fingers ; SPLICING FACTOR 3A SUBUNIT 2			
MGC05856	1	508	Complement C1q-like protein 4	C1q ; GLIACOLIN-RELATED ; CEREBELLIN-RELATED ; TNF-like ; C1Q		K03987_04610_Complement and coagulation cascades; K03987_05010_Alzheimers disease; K03988_04610_Complement and coagulation cascades; K03988_05010_Alzheimers disease;	
MGC05865	2	726		TNF-like			

MGC05869	1	446	Collagen alpha-1(XII) chain	VWFA ; INTEGRIN ALPHA-RELATED ; VON WILENBRAND FACTOR RELATED ; vWA-like ; VWA ; VWFADOMAIN	GO:0030199_P_collagen fibril organization; GO:0030020_F_extracellular matrix structural constituent conferring tensile strength; GO:0001501_P_skeletal development; GO:0005595_C_collagen type XII;		
MGC05870	2	661	Neurocalcin homolog	HIPPOCALCIN ; EF_HAND_1 ; RECOVERIN ; EF_HAND_2 ; NCAH_DROME_P42325; ; EF-hand ; CALCIUM BINDING PROTEINS ; efhand	GO:0005509_F_calcium ion binding; GO:0005515_F_protein binding;	K08328_04740_Olfactory transduction;	1.6.3.1_NAD(P)Hoxidase.; 3.1.2.15_Ubiquitinthiolesterase.; 2.7.11.17_Calcium/calmodulin-dependentproteinkinase.;
MGC05871	4	725	Peroxidasin homolog	Immunoglobulin ; Glucocorticoid receptor-like (DNA-binding domain)			
MGC05878	1	553	Mytimycin (Fragment)	EF_HAND_1 ; EF-hand ; efhand			
MGC05881	1	567	Low affinity immunoglobulin epsilon Fc receptor	REGENERATING GENE TYPE IV-RELATED ; C-type lectin-like ; C_TYPE_LECTIN_1 ; LITHOSTATHINE ; Lectin_C ; ANTIFREEZEII ; C_TYPE_LECTIN_2	GO:0009897_C_external side of plasma membrane; GO:0002925_P_positive regulation of humoral immune response mediated by circulating immunoglobulin;	K06468_04640_Hematopoietic cell lineage;	
MGC05888	1	574	Hepatic leukemia factor	bZIP_2 ; THYROTROPH EMBRYONIC FACTOR ; THYROTROPH EMBRYONIC FACTOR RELATED	GO:0003677_F_DNA binding; GO:0003690_F_double-stranded DNA binding; GO:0007275_P_multicellular organismal development; GO:0005634_C_nucleus; GO:0006366_P_transcription from RNA polymerase II promoter;		
MGC05889	2	627	Cysteine and histidine-rich domain-containing protein 1	CHORD ; CYSTEINE AND HISTIDINE-RICH DOMAIN (CHORD)-CONTAINING, ZINC BINDING PROTEIN 1 ; CYSTEINE AND HISTIDINE-RICH DOMAIN (CHORD)-CONTAINING PROTEIN ; coiled-coil	GO:0005509_F_calcium ion binding; GO:0005515_F_protein binding; GO:0008270_F_zinc ion binding;		
MGC05891	1	666	Metalloproteinase inhibitor 4	TIMP-like			
MGC05905	5	793	CD82 antigen				
MGC05908	1	346	Leucine-rich repeat serine/threonine-protein kinase 1	P-loop containing nucleoside triphosphate hydrolases			
MGC05916	1	610	Heavy metal-binding protein HIP	C1q ; GLIACOLIN-RELATED ; CEREBELLIN-RELATED ; COMPLEMNTC1Q ; TNF-like ; C1Q			
MGC05918	1	630	THO complex subunit 6 homolog	WD_REPEATS_2 ; GPROTEINBRPT ; Q7NJ67_GLOVI_Q7NJ67; ; WD40 REPEAT PROTEIN ; U5 SNRNP-SPECIFIC PROTEIN-RELATED ; WD40 repeat-like ; WD_REPEATS_1 ; WD_REPEATS_REGION ; WD40	GO:0009408_P_response to heat; GO:0005515_F_protein binding; GO:0008283_P_cell proliferation; GO:0000347_C_THO complex;	K08266_04150_mTOR signaling pathway; K06666_04111_Cell cycle - yeast; K01062_00565_Ether lipid metabolism;	2.7.11.1_Non-specificserine/threonineproteinkinase.; 2.7.11.7_[Myosinheavy-chain]kinase.;
MGC05919	1	610	Perlucin	C-type lectin-like ; C_TYPE_LECTIN_1 ; C-TYPE LECTIN SUPERFAMILY MEMBER ; Lectin_C ; GALACTOSE-SPECIFIC C-TYPE LECTIN ; EGF_2 ; C_TYPE_LECTIN_2			
MGC05922	3	692	Gamma-interferon-inducible lysosomal thiol reductase	SAP_A ; GILT ; GAMMA-INTERFERON INDUCIBLE LYSOSOMAL THIOL REDUCTASE (GILT)-RELATED	GO:0005764_C_lysosome; GO:0005576_C_extracellular region; GO:0019886_P_antigen processing and presentation of exogenous peptide antigen via MHC class II; GO:0015036_F_disulfide oxidoreductase activity;		

MGC05924	4	594		C-type lectin-like ; ASIALOGLYCOPROTEIN RECEPTOR ; C-TYPE LECTIN SUPERFAMILY MEMBER			
MGC05931	2	714	Ankyrin repeat domain-containing protein 16	ANKYRIN ; Ank ; ANKYRIN REPEAT-CONTAINING ; ANK_REPEAT ; ANK_REP_REGION ; Ankyrin repeat		K08803_05219_Bladder cancer;	2.7.11.1_Non-specificserine/threonineproteinkinase.;
MGC05935	2	659	Cysteine and tyrosine-rich protein 1				
MGC05939	1	622	Fos-related antigen 2	FOS-RELATED ANTIGEN 2 ; LEUZIPPRFOS ; FOS TRANSCRIPTION FACTOR-RELATED ; coiled-coil			
MGC05940	4	794	Putative surface protein SACOL0050	coiled-coil			
MGC05957	1	624	Tripartite motif-containing protein 45	RING/U-box ; TRIM56 PROTEIN ; 2FE2S_FER_1 ; ZINC_FINGER_C2H2_1 ; Glucocorticoid receptor-like (DNA-binding domain) ; ZF_BBOX ; zf-B_box ; RING FINGER-CONTAINING PROTEIN-RELATED		K10054_04120_Ubiquitin mediated proteolysis; K10054_05221_Acute myeloid leukemia;	
MGC05963	8	640	UBX domain-containing protein 1	UBX6(YEAST)-RELATED ; UBIQUITIN-ASSOCIATED UBA/UBX DOMAIN-CONTAINING ; UBA-like ; ZINC_FINGER_C2H2_1 ; coiled-coil ; UBA			3.1.2.15_Ubiquitinthiolesterase.;
MGC05973	1	597	Tripartite motif-containing protein 56	FYVE/PHD zinc finger ; RING/U-box ; ZF_RING_1 ; zf-C3HC4 ; ZF_RING_2 ; ZF_BBOX ; RING FINGER-CONTAINING ; RING FINGER-CONTAINING PROTEIN-RELATED			
MGC05975	3	550	Agrin	Kazal_2 ; Kazal-type serine protease inhibitors		K06254_04512_ECM-receptor interaction;	
MGC05979	1	602	Ubiquitin-conjugating enzyme E2 8	UBIQUITIN_CONJUGAT_2 ; UBIQUITIN-CONJUGATING ENZYME E2 ; UBC-like ; UQ_con ; WD_REPEATS_1 ; Q84K93_GOSHI_Q84K93;		K06689_04120_Ubiquitin mediated proteolysis;	6.3.2.19_Ubiquitin--proteinligase.;
MGC05980	1	502	Histone acetyltransferase MYST4	MYST4 ; MYST-RELATED PROTEINS			2.3.1.48_Histoneacetyltransferase.;
MGC05982	2	590	Caprin-2	C1q ; GPI-ANCHORED PROTEIN P137 ; COMPLEMNTC1Q ; TNF-like ; C1Q	GO:0005737_C_cytoplasm; GO:0005739_C_mitochondrion; GO:0030308_P_negative regulation of cell growth;		
MGC05984	2	1206	Probable histone acetyltransferase MYST1	MYST HISTONE ACETYLTRANSFERASE 1 ; MOZ_SAS ; Acyl-CoA N-acyltransferases (Nat) ; MYST-RELATED PROTEINS ; Chromo domain-like			2.3.1.48_Histoneacetyltransferase.;
MGC05987	1	682	Complement C1q tumor necrosis factor-related protein 9	C1q ; COLLAGEN ALPHA CHAIN ; COMPLEMNTC1Q ; TNF-like ; C1Q			
MGC06001	1	529	Muscle M-line assembly protein unc-89	Immunoglobulin ; IG_LIKE ; I-set ; ig ; TITIN	GO:0040011_P_locomotion; GO:0030018_C_Z disc;	K00907_04020_Calcium signaling pathway; K00907_04510_Focal adhesion; K00907_04810_Regulation of actin cytoskeleton;	2.7.11.1_Non-specificserine/threonineproteinkinase.; 2.7.11.18_[Myosinlight-chain]kinase.;
MGC06004	2	1034	Glyoxylate reductase/hydroxypyruvate reductase	2-Hacid_dh ; GLYOXYLATE/HYDROXYPYRUVATE REDUCTASE ; Formate/glycerate dehydrogenase catalytic domain-like ; NAD(P)-binding Rossmann-fold domains ; 2-Hacid_dh_C ; 2-HYDROXYACID DEHYDROGENASE		K00049_00620_Pyruvate metabolism; K00049_00630_Glyoxylate and dicarboxylate metabolism; K00015_00630_Glyoxylate and dicarboxylate metabolism;	1.1.1.79_Glyoxylatereductase(NADP(+)).; 1.1.1.26_Glyoxylatereductase.;
MGC06006	1	699	Fibrinolytic enzyme, isozyme C	TRYPSIN_HIS ; SERINE PROTEASE-RELATED ; Trypsin ; CHYMOTRYPSIN ; SERINE PROTEASE-RELATED, INSECT ; TRYPSIN_DOM ; Trypsin-like serine proteases			3.4.21.109_Matriptase.; 3.4.21.1_Chymotrypsin.; 3.4.21.4_Trypsin.;

MGC06008	2	644	Hepatic lectin	C-type lectin-like ; C-TYPE LECTIN SUPERFAMILY MEMBER ; Lectin_C ; LOW AFFINITY IMMUNOGLOBULIN EPSILON FC RECEPTOR (CD23 ANTIGEN) ; C_TYPE_LECTIN_2			
MGC06011	1	671					
MGC06020	2	668	Tubby protein homolog	coiled-coil			
MGC06024	1	602	Perlucin	C-type lectin-like ; C_TYPE_LECTIN_1 ; C-TYPE LECTIN SUPERFAMILY MEMBER ; Lectin_C ; GALACTOSE-SPECIFIC C-TYPE LECTIN ; ANTIFREEZEII ; C_TYPE_LECTIN_2			
MGC06040	1	623	Perlucin	REGENERATING GENE TYPE IV-RELATED ; C-type lectin-like ; C_TYPE_LECTIN_1 ; LITHOSTATHINE ; Lectin_C ; C_TYPE_LECTIN_2			
MGC06041	2	1200	Stress-induced-phosphoprotein 1	TPR_1 ; TPR ; STRESS-INDUCED-PHOSPHOPROTEIN 1 (STI1) (HSC70/HSP90-ORGANIZING PROTEIN) ; TPR_2 ; TPR-like ; TPR REPEAT CONTAINING PROTEIN ; TPR_REGION			3.1.3.16_Phosphoproteinphosphatase.;
MGC06058	2	762	Ficolin-2	FIBRINOGEN AND FIBRONECTIN ; Fibrinogen C-terminal domain-like ; Fibrinogen_C ; FIBRIN_AG_C_DOMAIN ; FICOLIN			
MGC06061	1	650	BTB/POZ domain-containing protein KCTD7	POZ domain ; POTASSIUM CHANNEL TETRAMERIZATION DOMAIN-CONTAINING ; POTASSIUM CHANNEL TETRAMERISATION DOMAIN CONTAINING ; K_tetra			
MGC06064	2	654	C-type lectin domain family 4 member E	C-type lectin-like ; C_TYPE_LECTIN_1 ; C-TYPE LECTIN SUPERFAMILY MEMBER ; Lectin_C ; GALACTOSE-SPECIFIC C-TYPE LECTIN ; ANTIFREEZEII ; C_TYPE_LECTIN_2	GO:0005529_F_sugar binding; GO:0005537_F_mannose binding; GO:0030139_C_endocytic vesicle;		
MGC06068	1	605	Prostaglandin E2 receptor EP4 subtype	G_PROTEIN_RECEP_F1_2 ; PROSTANOIDR ; PROSTAGLANDIN RECEPTOR ; 7tm_1 ; PRSTNOIDEP4R ; Family A G protein-coupled receptor-like	GO:0007188_P_G-protein signaling, coupled to cAMP nucleotide second messenger; GO:0004957_F_prostaglandin E receptor activity; GO:0016021_C_integral to membrane; GO:0006955_P_immune response;	K04261_04080_Neuroactive ligand-receptor interaction; K04263_04080_Neuroactive ligand-receptor interaction;	
MGC06074	1	641	Leucine-rich repeat-containing protein C10orf92	LEUCINE RICH REPEAT-CONTAINING ; RNI-like ; NALP (NACHT, LEUCINE RICH REPEAT AND PYRIN DOMAIN CONTAINING)-RELATED			
MGC06076	1	623	Kinase D-interacting substrate of 220 kDa	ANK REPEAT-CONTAINING ; ANKYRIN ; Ank ; ANKYRIN REPEAT-CONTAINING ; ANK_REPEAT ; ANK_REP_REGION ; Ankyrin repeat		K08803_05219_Bladder cancer;	2.7.11.1_Non-specificserine/threonineproteinkinase.; 2.1.1.43_Histone-lysineN-methyltransferase.;
MGC06080	1	646	Aldehyde dehydrogenase family 8 member A1	ALDEHYDE DEHYDROGENASE-RELATED ; ALDEHYDE DEHYDROGENASE ; ALDH-like ; Aldedh	GO:0005622_C_intracellular; GO:0042573_P_retinoic acid metabolic process; GO:0001758_F_retinal dehydrogenase activity; GO:0042574_P_retinal metabolic process;		1.2.1.47_4-trimethylammoniobutyraldehydedehydrogenase.; 1.2.1.3_Aldehydedehydrogenase(NAD(+)).; 1.2.1.36_Retinaldehydrogenase.;

MGC06089	1	590	Protein lap1	LEUCINE-RICH REPEAT CONTAINING PROTEIN ; LRR_1 ; LEUCINE-RICH REPEAT-CONTAINING PROTEIN ; L domain-like ; LEURICHRPT		K01768_00230_Purine metabolism; K10130_04115_p53 signaling pathway;	3.1.13.4_Poly(A)-specificribonuclease.;
MGC06090	1	651	Protein sel-1 homolog 1	SEL-1-LIKE PROTEIN, SEL-1L ; Sel1 ; HCP-like ; SEL-1-LIKE PROTEIN	GO:0016021_C_integral to membrane;		
MGC06095	6	770	Plancitoxin-1	DNase_II ; DEOXYRIBONUCLEASE II	GO:0006308_P_DNA catabolic process; GO:0005764_C_lysosome; GO:0003677_F_DNA binding; GO:0030218_P_erythrocyte differentiation; GO:0006259_P_DNA metabolic process; GO:0005515_F_protein binding; GO:0004531_F_deoxyribonuclease II activity;		3.1.22.1_DeoxyribonucleaseII.;
MGC06105	4	783	ADP-ribosylation factor 4	ADP-RIBOSYLATION FACTOR, ARF ; P-loop containing nucleoside triphosphate hydrolases ; RASTRNSFRMNG ; Arf ; small_GTP: small GTP-binding protein domain ; SAR1GTPBP ; ARF ; ADP RIBOSYLATION FACTOR-RELATED			
MGC06109	2	664	Golgi-associated plant pathogenesis-related protein 1	SCP ; CRISP_1 ; Q6DCW5_XENLA_Q6DCW5; ; GOLGI-ASSOCIATED PLANT PATHOGENESIS-RELATED PROTEIN 1 (GOLGI-ASSOCIATED PR-1 PROTEIN) ; CYSTEINE-RICH SECRETORY PROTEIN (CRISP/SCP/TPX1)-RELATED ; V5TPXLIKE ; PR-1-like			
MGC06110	2	1055	Aspartate aminotransferase, cytoplasmic	TRANSAMINASE ; Aminotran_1_2 ; coiled-coil ; ASPARTATE AMINOTRANSFERASE ; PLP-dependent transferases			2.6.1.1_Aspartatetransaminase.;
MGC06111	1	619	Semaphorin-1A	PSI ; Plexin repeat ; Sema domain ; SEMA ; Sema ; SEMAPHORIN		K06521_04360_Axon guidance; K06840_04360_Axon guidance;	
MGC06112	1	595	C-type mannose receptor 2	C-type lectin-like ; C-TYPE LECTIN SUPERFAMILY MEMBER ; Lectin_C ; gb def: Hypothetical protein F52E1.2 ; C_TYPE_LECTIN_2			
MGC06115	4	703	Proteasome subunit alpha type-4	N-terminal nucleophile aminohydrolases (Ntn hydrolases) ; Proteasome ; PROTEASOME_B ; PROTEASOME SUBUNIT ALPHA TYPE 4 ; PROTEASOME SUBUNIT ALPHA/BETA ; PROTEASOME_A	GO:0042802_F_identical protein binding; GO:0000502_C_proteasome complex; GO:0005515_F_protein binding;	K02728_03050_Proteasome;	3.4.25.1_Proteasomeendopeptidasecomplex.;
MGC06118	2	658		C1q ; TNF-like ; coiled-coil ; C1Q			
MGC06124	2	625	Putative L-amino-acid oxidase yobN	AMINE OXIDASE ; Amino_oxidase ; gb def: OTTMUSP00000005895 ; FAD/NAD(P)-binding domain			1.4.3.2_L-amino-acidoxidase.;
MGC06130	1	580					
MGC06132	5	690	DnaJ homolog subfamily C member 5B	DNAJ/HSP40 ; DNAJPROTEIN ; DNAJ_2 ; DnaJ ; CHAPERONE PROTEIN DNAJ ; Chaperone J-domain			
MGC06139	1	554	Complement C1q tumor necrosis factor-related protein 2	C1q ; CEREBELLIN-RELATED ; COMPLEMNTC1Q ; TNF-like ; C1Q		K03986_04610_Complement and coagulation cascades; K03986_05010_Alzheimers disease;	
MGC06141	2	1186					
MGC06145	2	677	Lysine-specific demethylase 5B	ARID-like ; JUMONJI/ARID DOMAIN-CONTAINING PROTEIN 1 ; JUMONJI DOMAIN CONTAINING PROTEIN ; JmjN ; JMJN ; ARID			
MGC06150	2	467					

MGC06154	2	667	Collagen alpha-1(X) chain	C1q ; PEROXIDASE_1 ; TNF-like ; coiled-coil			
MGC06156	1	634	Complement component 1 Q subcomponent-binding protein, mitochondrial	Acidic mitochondrial matrix protein p32 ; COMPLEMENT COMPONENT 1 ; MAM33	GO:0005739_C_mitochondrion; GO:0005515_F_protein binding;		
MGC06170	2	737	Complement C1q-like protein 4	C1q ; GLIACOLIN-RELATED ; CEREBELLIN-RELATED ; COMPLEMNTC1Q ; TNF-like ; C1Q	GO:0005737_C_cytoplasm;	K03987_04610_Complement and coagulation cascades; K03987_05010_Alzheimers disease;	
MGC06171	4	640	4F2 cell-surface antigen heavy chain	(Trans)glycosidases ; AMYLASE ; ALPHA-AMYLASE ; Alpha-amylase		K01226_00500_Starch and sucrose metabolism; K01182_00500_Starch and sucrose metabolism; K01187_00052_Galactose metabolism; K01187_00500_Starch and sucrose metabolism;	3.2.1.93_Alpha,alpha-phosphotrehalase.; 3.2.1.10_Oligo-1,6-glucosidase.; 3.2.1.20_Alpha-glucosidase.;
MGC06172	1	605	Echotoxin-2	Anemone pore-forming cytolysin			
MGC06173	1	632	14-3-3-like protein 2	Q6UFZ7_ONCMY_Q6UFZ7; ; 14-3-3 ; 1433ZETA ; 14-3-3 protein	GO:0040010_P_positive regulation of growth rate;	K06630_04110_Cell cycle;	
MGC06174	2	800	RNA-binding protein 25				
MGC06177	1	565	Protein scribble homolog	LEUCINE-RICH REPEAT-CONTAINING PROTEIN 1, LRRC1/LAP4 ; LRR_1 ; LEUCINE-RICH REPEAT-CONTAINING PROTEIN ; L domain-like ; LEURICHRPT		K10130_04115_p53 signaling pathway; K01768_00230_Purine metabolism;	4.6.1.1_Adenylatecyclase.; 3.1.3.16_Phosphoproteinphosphatase.;
MGC06178	1	626	Fibrinogen C domain-containing protein 1	FIBRINOGEN AND FIBRONECTIN ; Fibrinogen C-terminal domain-like ; Fibrinogen_C ; FIBRIN_AG_C_DOMAIN		K06252_04510_Focal adhesion; K06252_04512_ECM-receptor interaction;	
MGC06197	1	631	Collagen alpha-1(IX) chain	Immunoglobulin ; CELL ADHESION MOLECULE ; IMMUNOGLOBULIN DOMAIN SUPERFAMILY (SENSORY GUIDANCE PROTEIN) ; IG_LIKE ; ig ; Collagen ; CA26_CHICK_P15988;		K06491_04514_Cell adhesion molecules (CAMs); K06753_04360_Axon guidance;	1.11.1.7_Peroxidase.;
MGC06198	3	972	Tetraspanin-7	CD63 ANTIGEN (MELANOMA-ASSOCIATED ANTIGEN ME491) ; Tetraspanin ; TMFOUR ; TETRASPANIN ; Tetraspannin			
MGC06204	2	609	Complement C1q tumor necrosis factor-related protein 3	C1q ; COLLAGEN ALPHA CHAIN ; COMPLEMNTC1Q ; TNF-like ; COMPLEMENT C1Q TUMOR NECROSIS FACTOR-RELATED PROTEIN 3 ; C1Q			
MGC06205	2	676	E3 ubiquitin-protein ligase SIAH1	RING/U-box ; ZF_SIAH ; TRAF domain-like ; SEVEN IN ABSENTIA HOMOLOG 1 ; Sina ; ZF_RING_2 ; SEVEN IN ABSENTIA HOMOLOG		K04506_04115_p53 signaling pathway; K04506_04120_Ubiquitin mediated proteolysis; K04506_04310_Wnt signaling pathway;	
MGC06214	1	645	Protein FADD	DEATH domain ; DED			
MGC06219	4	1415	Probable endochitinase	CHITINASE ; Chitinase insertion domain ; BRAIN CHITINASE AND CHIA ; CBM_14 ; (Trans)glycosidases ; CHIT_BIND_II ; Glyco_hydro_18 ; Invertebrate chitin-binding proteins	GO:0006032_P_chitin catabolic process; GO:0008061_F_chitin binding; GO:0005615_C_extracellular space; GO:0004568_F_chitinase activity; GO:0009617_P_response to bacterium; GO:0006955_P_immune response;	K01183_00530_Aminosugars metabolism;	3.2.1.14_Chitinase.;
MGC06221	1	593	Digestive cysteine proteinase 2	Cysteine proteinases ; Peptidase_C1 ; CYSTEINE PROTEASE FAMILY C1-RELATED ; CATHEPSIN L ; THIOL_PROTEASE_ASN		K01368_04612_Antigen processing and presentation; K01365_04612_Antigen processing and presentation;	3.4.22.43_CathepsinV.; 3.4.22.15_CathepsinL.;
MGC06229	3	614	SH3 domain-binding protein 2	SH3-BINDING ; PH ; SUBPH_DOMAIN ; PH domain-like	GO:0007165_P_signal transduction; GO:0005070_F_SH3/SH2 adaptor activity;	K07984_04650_Natural killer cell mediated cytotoxicity;	
MGC06234	1	532					
MGC06239	1	280					

MGC06244	4	719	Elongation factor 1-alpha	EF-Tu/eEF-1alpha/eIF2-gamma C-terminal domain ; TRANSLATION FACTOR ; GTP_EFTU_D2 ; Translation proteins ; GTP_EFTU_D3 ; ELONGATION FACTOR 1-ALPHA (EF-1-ALPHA)			2.7.7.4_Sulfateadenylyltransferase.; 2.7.1.25_Adenylyl-sulfatekinase.;
MGC06252	1	583	Cathepsin L	Cysteine proteinases ; Q86GJ2_HYDAT_Q86GJ2; ; THIOL_PROTEASE_CYS ; Peptidase_C1 ; PAPAIN ; THIOL_PROTEASE_HIS ; CYSTEINE PROTEASE FAMILY C1-RELATED ; CATHEPSIN L		K01365_04612_Antigen processing and presentation;	3.4.22.15_CathepsinL.;
MGC06257	8	731	Proteasome subunit beta type-7	N-terminal nucleophile aminohydrolases (Ntn hydrolases) ; Proteasome ; PROTEASOME_B ; PROTEASOME ; PROTEASOME SUBUNIT BETA TYPE 7,10 ; PROTEASOME SUBUNIT ALPHA/BETA		K02739_03050_Proteasome;	3.4.25.1_Proteasomeendopeptidasecomplex.;
MGC06261	1	626	Calcium and integrin-binding protein 1	EF_HAND_1 ; EF_HAND_2 ; CALCIUM AND INTEGRIN-BINDING PROTEIN 1 (CALMYRIN) (DNA-PKCS INTERACTING PROTEIN) (KINASE INTERACTING PROTEIN) (KIP) (CIB) ; CALCINEURIN B ; EF-hand ; efhand ; CALB_NAEGR_P42322;			1.6.3.1_NAD(P)Hoxidase.;
MGC06262	1	623	Transcription intermediary factor 1-beta	TRIM56 PROTEIN ; B-box zinc-binding domain ; Glucocorticoid receptor-like (DNA-binding domain) ; ZF_BBOX ; zf-B_box ; BBOXZNFINGER ; RING FINGER-CONTAINING PROTEIN-RELATED			
MGC06263	1	643	Xylosyltransferase oxt	WSC			
MGC06267	1	597	Secretin receptor				
MGC06268	1	537	Transmembrane emp24 domain-containing protein 3	EMP24_GP25L ; COP-COATED VESICLE MEMBRANE PROTEIN P24 (EMP24/GP25L FAMILY) ; COPII-COATED VESICLE MEMBRANE PROTEIN ; Supernatant protein factor (SPF), C-terminal domain ; GOLD			
MGC06270	1	591	Uncharacterized protein KIAA1704	LIPOPOLYSACCHARIDE SPECIFIC RESPONSE-7-RELATED ; DNAJ HOMOLOG SUBFAMILY C MEMBER 8/LIPOPOLYSACCHARIDE SPECIFIC RESPONSE-7-RELATED ; coiled-coil			
MGC06272	2	800	Plasminogen	KRINGLE_1 ; Q6TCI0_MOUSE_Q6TCI0; ; SERINE PROTEASE-RELATED ; Kringle-like ; PLASMINOGEN ; KRINGLE_2 ; KRINGLE ; Kringle		K01315_04080_Neuroactive ligand-receptor interaction; K01315_04610_Complement and coagulation cascades;	3.4.21.7_Plasmin.;
MGC06290	10	592	C-type lectin domain family 4 member M	C-type lectin-like			
MGC06297	2	737	Caprin-2	C1q ; COLLAGEN ALPHA CHAIN ; COMPLEMNTC1Q ; TNF-like ; COMPLEMENT C1Q TUMOR NECROSIS FACTOR-RELATED PROTEIN 3 ; C1Q	GO:0005737_C_cytoplasm; GO:0005739_C_mitochondrion; GO:0030308_P_negative regulation of cell growth;		
MGC06299	2	680	Thyroglobulin	INSULIN-LIKE GROWTH FACTOR BINDING PROTEIN ; Thyroglobulin_1 ; Thyroglobulin type-1 domain ; INSULIN-LIKE GROWTH FACTOR BINDING PROTEIN 3 ; THYROGLOBULIN_1_2	GO:0007165_P_signal transduction; GO:0005576_C_extracellular region;	K10809_05320_Autoimmune thyroid disease;	
MGC06301	2	639	DPH3 homolog	zf-CSL ; SUBZF_DPH			
MGC06304	2	666	Mitochondrial import inner membrane translocase subunit Tim16	Pam16 ; MITOCHONDRIA ASSOCIATED GRANULOCYTE MACROPHAGE CSF SIGNALING MOLECULE ; Chaperone J-domain	GO:0001700_P_embryonic development via the syncytial blastoderm; GO:0002168_P_instar larval development;		

MGC06305	2	922	E3 ubiquitin-protein ligase MIB1	ANKYRIN ; MIND BOMB ; RING/U-box ; Ank ; ANKYRIN REPEAT-CONTAINING ; ZF_RING_2 ; ANK_REPEAT ; ANK_REP_REGION ; Ankyrin repeat			2.7.11.1_Non-specificserine/threonineprotеinkinase.;
MGC06307	2	785	RuvB-like 1	RUVB-RELATED REPTIN AND PONTIN ; P-loop containing nucleoside triphosphate hydrolases ; TIP49 ; AAA		K04499_04310_Wnt signaling pathway;	
MGC06333	1	544	Tetratricopeptide repeat protein 18	Protein prenylyltransferase ; TPR_2 ; TETRATRICOPEPTIDE REPEAT PROTEIN, TPR ; BARDET-BIEDL SYNDROME 4, BBS4 ; TPR_REGION			
MGC06347	1	529	Cerebellin-3	C1q ; CEREBELLIN-RELATED ; COMPLEMNTC1Q ; TNF-like ; TONB_DEPENDENT_REC_1 ; C1Q			
MGC06348	1	568	Cholecystokinin receptor type A	Family A G protein-coupled receptor-like			
MGC06352	1	536	Neurocan core protein	C-type lectin-like ; ASIALOGLYCOPROTEIN RECEPTOR ; C_TYPE_LECTIN_1 ; C-TYPE LECTIN SUPERFAMILY MEMBER ; Lectin_C ; ANTIFREEZEII ; C_TYPE_LECTIN_2			3.4.21.84_LimulusclottingfactorC.;
MGC06359	1	528	Fibroblast growth factor receptor	Immunoglobulin ; IG_LIKE ; ig ; FIBROBLAST GROWTH FACTOR RECEPTOR ; TYROSINE PROTEIN KINASE		K05093_04810_Regulation of actin cytoskeleton; K05093_05215_Prostate cancer; K04362_04010_MAPK signaling pathway; K04362_04520_Adherens junction; K04362_04810_Regulation of actin cytoskeleton; K04362_05215_Prostate cancer; K04362_05218_Melanoma;	2.7.10.1_Receptorprotein-tyrosinekinase.;
MGC06364	1	680	78 kDa glucose-regulated protein	Heat shock protein 70kD (HSP70), peptide-binding domain ; HEAT SHOCK PROTEIN 70KDA ; Heat shock protein 70kD (HSP70), C-terminal subdomain ; HEAT SHOCK PROTEIN 70 (HSP70) ; HSP70 ; ER_TARGET		K09490_05060_tba;	
MGC06366	1	485	Stromal membrane-associated protein 1	SMAP1 ; CENTAURIN/ARF ; ArfGap ; Pyk2-associated protein beta ARF-GAP domain ; ARFGAP ; REVINTRACTNG		K05737_04810_Regulation of actin cytoskeleton; K05737_05120_Epithelial cell signaling in Helicobacter pylori infection;	
MGC06372	3	698	Complement C1q tumor necrosis factor-related protein 3	C1q ; CEREBELLIN-RELATED ; COMPLEMNTC1Q ; TNF-like ; C1Q		K03987_04610_Complement and coagulation cascades; K03987_05010_Alzheimers disease;	
MGC06378	5	796	Transmembrane protease serine 6	TRYPSIN_HIS ; SERINE PROTEASE-RELATED ; Trypsin ; CHYMOTRYPSIN ; SERINE PROTEASE-RELATED, INSECT ; TRYPSIN_DOM ; Trypsin-like serine proteases	GO:0042730_P_fibrinolysis; GO:0005886_C_plasma membrane; GO:0006508_P_proteolysis; GO:0016021_C_integral to membrane; GO:0004252_F_serine-type endopeptidase activity;		3.4.21.34_Plasmakallikrein.; 3.4.21.9_Enteropeptidase.; 3.4.21.106_Hepsin.; 3.4.21.7_Plasmin.; 3.4.21.27_CoagulationfactorXIa.;
MGC06383	1	622	Fucolectin-6	Galactose-binding domain-like ; coiled-coil ; PROKAR_LIPOPROTEIN			
MGC06392	2	649	Protein LITAF homolog	LIPOPOLYSACCHARIDE-INDUCED TRANSCRIPTION FACTOR REGULATING TUMOR NECROSIS FACTOR ALPHA			

MGC06398	1	658	Ankyrin repeat domain-containing protein 46	ANKYRIN ; Ank ; ANKYRIN REPEAT SMALL PROTEIN ; ANKYRIN REPEAT-CONTAINING ; ANK_REPEAT ; ANK_REP_REGION ; Ankyrin repeat		K08803_05219_Bladder cancer;	2.7.11.1_Non-specificserine/threonineprotein kinase.; 2.4.2.30_NAD(+)ADP-ribosyltransferase.;
MGC06400	1	666	Major egg antigen	ACRYSTALLIN ; HEAT SHOCK PROTEIN 30 ; SMALL HEAT-SHOCK PROTEIN (HSP20) FAMILY ; HSP20 ; HSP20-like chaperones	GO:0005515_F_protein binding;		
MGC06401	1	647					
MGC06405	1	493		Snake toxin-like			
MGC06406	1	659	Flocculation protein FLO11				
MGC06411	1	433	Slit homolog 1 protein	LRR_1 ; CHAOPTIN ; L domain-like ; LEURICHRPT ; LEUCINE-RICH TRANSMEMBRANE PROTEINS	GO:0005886_C_plasma membrane; GO:0048749_P_compound eye development; GO:0007411_P_axon guidance; GO:0007185_P_transmembrane receptor protein tyrosine phosphatase signaling pathway;	K06838_04360_Axon guidance; K06839_04360_Axon guidance; K06850_04360_Axon guidance;	
MGC06419	1	651		PROSTAGLANDIN G/H SYNTHASE ; Heme-dependent peroxidases ; ABC_TRANSPORTER_1 ; PEROXIDASE_3 ; An_peroxidase ; FEEBLY-LIKE PROTEIN			1.14.99.1_Prostaglandin-endoperoxidesynthase.;
MGC06421	6	900	Probable cytosolic iron-sulfur protein assembly protein	WD_REPEATS_2 ; O80990_ARATH_O80990; ; WD40 PROTEIN CIAO1 ; GPROTEINBRPT ; WD40 repeat-like ; WD_REPEATS_1 ; WD_REPEATS_REGION ; WD40	GO:0005515_F_protein binding; GO:0003700_F_transcription factor activity; GO:0006357_P_regulation of transcription from RNA polymerase II promoter; GO:0008284_P_positive regulation of cell proliferation;	K04508_04310_Wnt signaling pathway; K01062_00565_Ether lipid metabolism;	2.7.11.1_Non-specificserine/threonineprotein kinase.; 2.3.1.48_Histoneacetyltransferase.; 2.7.11.7_[Myosinheavy-chain]kinase.;
MGC06432	1	646	Putative leucine-rich repeat-containing protein DDB_G0290503	STRUCTURAL MAINTENANCE OF CHROMOSOMES SMC FAMILY MEMBER ; coiled-coil			
MGC06436	2	690	Neurotrypsin	SRCR-like ; SRCR ; LYSYL OXIDASE-RELATED ; coiled-coil ; SRCR_2 ; SPERACTRCPTR			
MGC06446	2	766	Short-chain collagen C4 (Fragment)				
MGC06458	2	735	Tenascin	FIBRINOGEN AND FIBRONECTIN ; Fibrinogen C-terminal domain-like ; Fibrinogen_C	GO:0007528_P_neuromuscular junction development; GO:0005604_C_basement membrane; GO:0005576_C_extracellular region;	K06252_04510_Focal adhesion; K06252_04512_ECM-receptor interaction;	
MGC06468	1	566	Sorbitol dehydrogenase	ADH_N ; ALCOHOL DEHYDROGENASE RELATED ; ADH_ZINC ; ZINC-TYPE ALCOHOL DEHYDROGENASE-RELATED ; GroES-like	GO:0007601_P_visual perception; GO:0003939_F_L-iditol 2-dehydrogenase activity; GO:0005625_C_soluble fraction; GO:0006060_P_sorbitol metabolic process;	K00008_00051_Fructose and mannose metabolism;	1.1.1.14_L-iditol2-dehydrogenase.;
MGC06478	1	721					
MGC06482	1	633	Dipeptidyl peptidase 2	PROTEASE S28 PRO-X CARBOXYPEPTIDASE-RELATED ; DIPEPTIDYL-PEPTIDASE II ; Peptidase_S28	GO:0008236_F_serine-type peptidase activity; GO:0005515_F_protein binding;		3.4.16.2_LysosomalPro-Xaacarboxypeptidase.; 3.4.14.2_Dipeptidyl-peptidaseII.;
MGC06493	3	750	Cholecystokinin receptor type A	G_PROTEIN_RECEP_F1_2 ; G-PROTEIN COUPLED RECEPTOR ; GPCRRHODOPSN ; 7tm_1 ; Family A G protein-coupled receptor-like	GO:0004930_F_G-protein coupled receptor activity; GO:0016021_C_integral to membrane; GO:0007186_P_G-protein coupled receptor protein signaling pathway;	K04240_04080_Neuroactive ligand-receptor interaction; K04194_04020_Calcium signaling pathway; K04194_04080_Neuroactive ligand-receptor interaction;	
MGC06495	1	560	RING finger protein 170	RING/U-box ; ZF_RING_1 ; zf-C3HC4 ; ZF_RING_2			

MGC06496	2	523	U5 small nuclear ribonucleoprotein 40 kDa protein	WD_REPEATS_2 ; GPROTEINBRPT ; Q7ZXE3_XENLA_Q7ZXE3; ; WD40 REPEAT PROTEIN ; U5 SNRNP-SPECIFIC PROTEIN-RELATED ; WD40 repeat-like ; WD_REPEATS_1 ; WD_REPEATS_REGION ; WD40	GO:0031202_F_RNA splicing factor activity, transesterification mechanism; GO:0006396_P_RNA processing; GO:0005682_C_snRNP U5; GO:0005515_F_protein binding; GO:0008380_P_RNA splicing; GO:0005732_C_small nucleolar ribonucleoprotein complex;	K03130_03022_Basal transcription factors; K06666_04111_Cell cycle - yeast; K01852_00100_Biosynthesis of steroids;	2.7.11.1_Non-specificserine/threonineproteinkinase.; 2.3.1.48_Histoneacetyltransferase.;
MGC06503	1	601	Caprin-2	C1q ; GLIACOLIN-RELATED ; CEREBELLIN-RELATED ; COMPLEMNTC1Q ; TNF-like ; C1Q		K03987_04610_Complement and coagulation cascades; K03987_05010_Alzheimers disease;	
MGC06511	2	648	Formin-binding protein 1	FORMIN BINDING PROTEIN-RELATED, ARTHROPOD ; FCH ; FORMIN BINDING PROTEIN AND RELATED PROTEINS ; coiled-coil	GO:0007498_P_mesoderm development; GO:0005515_F_protein binding;	K07196_04910_Insulin signaling pathway;	
MGC06515	1	547	Nidogen-2	HLA CLASS II GAMMA CHAIN ; Thyroglobulin_1 ; MHC CLASS II-ASSOCIATED INVARIANT CHAIN-RELATED ; Thyroglobulin type-1 domain ; THYROGLOBULIN_1_1 ; THYROGLOBULIN_1_2			
MGC06523	1	643	Slit homolog 2 protein (Fragment)	LRR_1 ; LRRNT ; L domain-like ; LEURICHRPT ; LEUCINE-RICH TRANSMEMBRANE PROTEINS		K06838_04360_Axon guidance; K04308_04080_Neuroactive ligand-receptor interaction; K06839_04360_Axon guidance; K06850_04360_Axon guidance;	1.11.1.7_Peroxidase.;
MGC06530	5	1377	Multidrug resistance protein 1	ABC_TM1F ; MDR3_CAEEL_P34713; ; ABC_TRANSPORTER_2 ; ATP-BINDING CASSETTE TRANSPORTER ; P-loop containing nucleoside triphosphate hydrolases ; ABC_membrane ; MULTIDRUG RESISTANCE PROTEIN 1, 2, 3 (P GLYCOPROTEIN 1, 2, 3) ; ABC_TRANSPORTER_1 ; ABC_tran ; Multidrug resistance ABC transporter MsbA, N-terminal domain		K05659_02010_ABC transporters; K05664_02010_ABC transporters; K05658_02010_ABC transporters;	3.6.3.44_Xenobiotic-transportingATPase.;
MGC06543	1	662					
MGC06544	1	590	Heavy metal-binding protein HIP	C1q ; CEREBELLIN-RELATED ; COMPLEMNTC1Q ; TNF-like ; coiled-coil ; C1Q	GO:0007399_P_nervous system development; GO:0007268_P_synaptic transmission;		
MGC06547	1	717		CBM_14 ; CHIT_BIND_II ; CHITIN BINDING PERITROPHIN-A ; Invertebrate chitin-binding proteins			
MGC06549	1	682	ATP-dependent RNA helicase DDX39	HELICASE_CTER ; Helicase_C ; P-loop containing nucleoside triphosphate hydrolases ; DEAD (ASP-GLU-ALA-ASP) BOX POLYPEPTIDE 39 AND P47 ; DEAD BOX ATP-DEPENDENT RNA HELICASE		K01529_00500_Starch and sucrose metabolism; K01529_00790_Folate biosynthesis; K01509_00230_Purine metabolism;	
MGC06560	1	646	Charged multivesicular body protein 2a	SNF7-RELATED ; SNF7-LIKE PROTEIN (BC-2) (PUTATIVE BREAST ADENOCARCINOMA MARKER)			
MGC06561	2	641	Complement C1q-like protein 3	C1q ; C1Q-RELATED FACTOR ; COLLAGEN ALPHA CHAIN ; COMPLEMNTC1Q ; TNF-like ; C1Q	GO:0005515_F_protein binding;		
MGC06564	1	643		TNF receptor-like			
MGC06568	2	795		RING/U-box ; ZF_RING_2			

MGC06572	2	636	C3 and PZP-like alpha-2-macroglobulin domain-containing protein 8	A2M_recep ; Alpha-macroglobulin receptor domain ; MACROGLOBULIN/COMPLEMENT			
MGC06579	1	586	Complement C1q-like protein 2	C1q ; GLIACOLIN-RELATED ; CEREBELLIN-RELATED ; COMPLEMNTC1Q ; TNF-like ; C1Q			
MGC06584	2	627	Glutamate-rich WD repeat-containing protein 1	GLUTAMATE RICH WD REPEAT PROTEIN (GRWD) ; WD_REPEATS_2 ; GPROTEINBRPT ; WD40 REPEAT FAMILY ; WD40 repeat-like ; Q7S1P4_NEUCR_Q7S1P4; ; WD_REPEATS_1 ; WD_REPEATS_REGION ; WD40	GO:0009792_P_embryonic development ending in birth or egg hatching;		2.3.1.48_Histoneacetyltransferase.;
MGC06587	1	674	Counting factor associated protein D	Cysteine proteinases ; THIOL_PROTEASE_CYS ; Inhibitor_I29 ; Peptidase_C1 ; SUBCYSTEINE PROTEASE FAMILY C1-RELATED ; Q86GZ5_RHIAP_Q86GZ5;		K01365_04612_Antigen processing and presentation;	3.4.22.16_CathepsinH.;
MGC06588	2	539	H/ACA ribonucleoprotein complex subunit 4	PUA domain-like ; Pseudouridine synthase ; TruB_N ; CENTROMERE/MICROTUBULE BINDING PROTEIN CBF5			
MGC06601	1	696	Heat shock 70 kDa protein 14				
MGC06604	1	585	E3 ubiquitin-protein ligase mib1	MIB_HERC2		K10595_04120_Ubiquitin mediated proteolysis;	
MGC06606	3	728	RhoGEF domain-containing protein gxcJ				
MGC06607	1	636	WSC domain-containing protein 1	WSC		K00771_00532_Chondroitin sulfate biosynthesis; K00771_01030_tba;	2.4.2.26_Proteinxylosyltransferase.;
MGC06612	1	686	High mobility group protein B1	HMG_box ; HMG-box ; SWI/SNF-RELATED CHROMATIN BINDING PROTEIN ; coiled-coil ; HMG_BOX_2 ; HIGHMOBLTY12		K10802_03410_Base excision repair;	
MGC06615	1	597	Keratin-associated protein 10-7	ShK			
MGC06626	1	488	Sushi, nidogen and EGF-like domain-containing protein 1	EGF/Laminin ; SUSHI ; COMPLEMENT COMPONENT-RELATED SUSHI DOMAIN-CONTAINING ; CUB AND SUSHI MULTIPLE DOMAINS PROTEIN ; TSP-1 type 1 repeat ; EGF_1 ; Complement control module/SCR domain ; EGF_2 ; Sushi ; TSP1			
MGC06628	3	796	Ribosome biogenesis protein wdr12	WD40 REPEAT PROTEIN 12 ; Q8H594_EEEEE_Q8H594; ; WD_REPEATS_2 ; GPROTEINBRPT ; WD40 repeat-like ; WD40 REPEAT PROTEIN 12, 37 ; WD_REPEATS_REGION ; NLE ; WD40	GO:0007219_P_Notch signaling pathway; GO:0005515_F_protein binding; GO:0005634_C_nucleus;	K10260_04120_Ubiquitin mediated proteolysis; K10259_04111_Cell cycle - yeast; K10259_04120_Ubiquitin mediated proteolysis;	2.7.11.7_[Myosinheavy-chain]kinase.;
MGC06629	1	572		TNF-like			
MGC06631	1	647					
MGC06632	1	630		coiled-coil			
MGC06637	2	1049	AP-3 complex subunit delta-1	Adaptin_N ; LEUKEMIA VIRUS RECEPTOR BLVR ; ARM repeat ; BLVR ; DELTA ADAPTIN-RELATED			
MGC06648	1	646	Probable serine/threonine-protein kinase YOL100W	Protein kinase-like (PK-like) ; SERINE/THREONINE-PROTEIN KINASE CHK2 (CDS1) ; PROTEIN_KINASE_ST ; Pkinase ; Q753H7_ASHGO_Q753H7; ; CALCIUM/CALMODULIN-DEPENDENT PROTEIN KINASE-RELATED ; PROTEIN_KINASE_DOM		K05688_05020_tba;	2.7.11.1_Non-specificserine/threonineproteinkinase.;
MGC06658	1	168	Ficolin-2	FIBRINOGEN AND FIBRONECTIN ; Fibrinogen C-terminal domain-like ; Fibrinogen_C ; FIBRIN_AG_C_DOMAIN ; FICOLIN			

MGC06659	1	609	Tyrosine-protein kinase HTK16	HT16_HYDAT_P53356; ; TYROSINE-PROTEIN KINASE SHARK ; SH2DOMAIN ; SH2 domain ; TYROSINE PROTEIN KINASE ; SH2			2.7.10.2_Non-specificprotein-tyrosinekinase.;
MGC06660	1	611	Angiopoietin-related protein 7	FIBRINOGEN AND FIBRONECTIN ; Fibrinogen C-terminal domain-like ; Fibrinogen_C ; FIBRIN_AG_C_DOMAIN		K06252_04510_Focal adhesion; K06252_04512_ECM-receptor interaction;	
MGC06663	1	554	Protein LITAF homolog	LIPOPOLYSACCHARIDE-INDUCED TRANSCRIPTION FACTOR REGULATING TUMOR NECROSIS FACTOR ALPHA			
MGC06671	2	594	Serine/threonine-protein phosphatase 6 regulatory ankyrin repeat subunit B	ANKYRIN ; Ank ; CORTACTIN-BINDING PROTEIN 2 ; ANKYRIN REPEAT-CONTAINING ; ANK_REPEAT ; ANK_REP_REGION ; Ankyrin repeat	GO:0005515_F_protein binding; GO:0005200_F_structural constituent of cytoskeleton;	K06623_04110_Cell cycle;	2.4.2.30_NAD(+)ADP-ribosyltransferase.; 2.7.11.1_Non-specificserine/threonineproteinkinase.;
MGC06674	2	657					
MGC06678	2	963		BPTI-like			
MGC06679	1	602	Peptidyl-prolyl cis-trans isomerase B	Cyclophilin-like ; CSAPPISMRASE ; CYCLOPHILIN ; Pro_isomerase ; CSA_PPIASE_2			5.2.1.8_Peptidylprolylisomerase.;
MGC06681	2	675					
MGC06685	1	637		ZP_2			
MGC06686	1	680	Caprin-2	C1q ; C1Q-RELATED FACTOR ; COLLAGEN ALPHA CHAIN ; COMPLEMNTC1Q ; TNF-like ; C1Q	GO:0005515_F_protein binding;	K03987_04610_Complement and coagulation cascades; K03987_05010_Alzheimers disease;	
MGC06687	1	576	Wiskott-Aldrich syndrome protein	WISKOTT-ALDRICH SYNDROME PROTEIN ; coiled-coil ; PH domain-like ; WH1		K05747_04520_Adherens junction; K05747_04810_Regulation of actin cytoskeleton;	
MGC06696	1	523	Techylectin-5A	FIBRINOGEN AND FIBRONECTIN ; Fibrinogen C-terminal domain-like ; Fibrinogen_C	GO:0016337_P_cell-cell adhesion; GO:0005529_F_sugar binding;	K06252_04510_Focal adhesion; K06252_04512_ECM-receptor interaction;	
MGC06702	1	340	Ubiquitin	UBIQUITIN (RIBOSOMAL PROTEIN L40) ; UBIQUITIN_2 ; Ubiquitin-like ; ubiquitin ; UBIQUITIN_1 ; UBIQUITIN		K02977_03010_Ribosome; K08770_03320_PPAR signaling pathway; K02927_03010_Ribosome; K04551_05020_tba;	
MGC06703	1	659	DNA-binding protein inhibitor ID-2-B	DNA-BINDING PROTEIN INHIBITOR ; HLH ; HLH, helix-loop-helix DNA-binding domain		K04680_04350_TGF-beta signaling pathway;	
MGC06711	4	780	Big defensin				
MGC06734	1	590		Snake toxin-like			
MGC06742	1	539	Formin-like protein 6				
MGC06746	1	450	Myoferlin	FER-1-LIKE ; FER-1-LIKE 3 ; C2DOMAIN ; C2 domain (Calcium/lipid-binding domain, CaLB) ; C2			2.7.11.13_ProteinkinaseC.;
MGC06749	1	669	Death-associated protein kinase 1	ANKYRIN ; Ank ; ANKYRIN REPEAT-CONTAINING ; P-loop containing nucleoside triphosphate hydrolases ; ANK_REPEAT ; ANK_REP_REGION ; SERINE/THREONINE-PROTEIN KINASE RIPK4 ; Ankyrin repeat		K06622_04110_Cell cycle; K08803_05219_Bladder cancer;	2.7.11.1_Non-specificserine/threonineproteinkinase.; 3.6.1.22_NAD(+)diphosphatase.;
MGC06752	1	549	Presenilin-2	PRESENILIN ; PRESENILIN 2 ; Presenilin		K04522_04330_Notch signaling pathway; K04522_05010_Alzheimers disease; K04505_04310_Wnt signaling pathway; K04505_04330_Notch signaling pathway; K04505_05010_Alzheimers disease;	
MGC06756	1	562	Deleted in malignant brain tumors 1 protein	CUB ; Spermadhesin, CUB domain ; LYSYL OXIDASE-RELATED ; DELETED IN MALIGNANT BRAIN TUMORS 1			3.4.21.42_ComplementsubcomponentC1s.; 3.4.24.19_ProcollagenC-endopeptidase.; 3.4.24.21_Astacin.;

MGC06766	2	648	NF-kappa-B inhibitor-interacting Ras-like protein 2	Miro ; P-loop containing nucleoside triphosphate hydrolases ; RASTRNSFRMNG ; RAS-RELATED GTPASE ; small_GTP: small GTP-binding protein domain ; KAPPA B-RAS	GO:0003924_F_GTPase activity; GO:0007249_P_I-kappaB kinase/NF-kappaB cascade;		
MGC06772	2	697	Caprin-2	C1q ; GPI-ANCHORED PROTEIN P137 ; COMPLEMNTC1Q ; TNF-like ; coiled-coil ; C1Q	GO:0005737_C_cytoplasm; GO:0005739_C_mitochondrion; GO:0030308_P_negative regulation of cell growth;		
MGC06773	1	594	Histone deacetylase 1	HISTONE DEACETYLASE ; HISDACETLASE ; HISTONE DEACETYLASE 1, 2 ,3 ; Arginase/deacetylase ; Hist_deacetyl ; HDASUPER	GO:0005737_C_cytoplasm; GO:0016568_P_chromatin modification; GO:0008134_F_transcription factor binding; GO:0000118_C_histone deacetylase complex; GO:0004407_F_histone deacetylase activity; GO:0005634_C_nucleus;	K06067_04110_Cell cycle; K06067_04330_Notch signaling pathway; K06067_05220_Chronic myeloid leukemia;	3.5.1.98_Histonedeacetylase.;
MGC06780	1	553	Cytochrome b-c1 complex subunit 7	UCR_14kD ; 14 kDa protein of cytochrome bc1 complex (Ubiquinol-cytochrome c reductase) ; UBIQUINOL-CYTOCHROME C REDUCTASE COMPLEX 14 KD PROTEIN		K00417_00190_Oxidative phosphorylation;	
MGC06783	1	506					
MGC06787	2	591	Transmembrane emp24 domain-containing protein eca	EMP24_GP25L ; COPII-COATED VESICLE MEMBRANE PROTEIN ; GLYCOPROTEIN 25L ; GOLD ; coiled-coil			
MGC06790	1	630	Thyroglobulin	Thyroglobulin_1 ; Thyroglobulin type-1 domain ; THYROGLOBULIN_1_2	GO:0007165_P_signal transduction; GO:0005576_C_extracellular region;	K10809_05320_Autoimmune thyroid disease;	
MGC06796	1	679	Baculoviral IAP repeat-containing protein 7-A	RING/U-box ; INHIBITOR OF APOPTOSIS ; ZF_RING_2 ; INHIBITOR OF APOPTOSIS 1, DIAP1		K04725_04120_Ubiquitin mediated proteolysis; K04725_04210_Apoptosis; K04725_04510_Focal adhesion; K04725_05222_Small cell lung cancer;	
MGC06800	1	564	Type-1B angiotensin II receptor				
MGC06809	1	604	Ribosomal protein S6 kinase 2 alpha	Protein kinase-like (PK-like) ; PROTEIN_KINASE_ST ; Pkinase ; K6A3_MOUSE_P18654; ; RIBOSOMAL PROTEIN S6 KINASE ALPHA 1,2,3 ; SERINE/THREONINE PROTEIN KINASE ; PROTEIN_KINASE_DOM	GO:0004672_F_protein kinase activity; GO:0007165_P_signal transduction; GO:0007417_P_central nervous system development; GO:0004674_F_protein serine/threonine kinase activity; GO:0001501_P_skeletal development;	K04373_04010_MAPK signaling pathway; K04373_04150_mTOR signaling pathway; K04373_04720_Long-term potentiation; K04373_04914_Progesterone-mediated oocyte maturation;	2.7.11.1_Non-specificserine/threonineproteinkinase.;
MGC06812	1	640	Cathepsin L1	Cysteine proteinases ; THIOL_PROTEASE_CYS ; Inhibitor_I29 ; Peptidase_C1 ; CATL_PIG_Q28944; ; CYSTEINE PROTEASE FAMILY C1-RELATED ; CATHEPSIN L		K01365_04612_Antigen processing and presentation;	3.4.22.15_CathepsinL.;
MGC06815	1	609		EGF/Laminin ; Cadherin-like			
MGC06822	2	683	Putative apoptosis inhibitor ORF87	BIR ; INHIBITOR OF APOPTOSIS ; Inhibitor of apoptosis (IAP) repeat ; INHIBITOR OF APOPTOSIS PROTEIN 1 AND 2, IAP1, IAP2 ; BIR_REPEAT_2		K04725_04120_Ubiquitin mediated proteolysis; K04725_04210_Apoptosis; K04725_04510_Focal adhesion; K04725_05222_Small cell lung cancer;	
MGC06828	1	605	Hypoxia up-regulated protein 1 (Fragment)	HEAT SHOCK PROTEIN 70KDA ; HSP70 ; Actin-like ATPase domain ; Q7ZUW2_BRARE_Q7ZUW2; ; HYPOXIA UPREGULATED 1 (HYOU1)-RELATED ; HEATSHOCK70		K09490_05060_tba;	

MGC06833	1	629	2-amino-3-ketobutyrate coenzyme A ligase, mitochondrial	CLASS II AMINOTRANSFERASE/8-AMINO-7-OXONONANOATE SYNTHASE ; 2-AMINO-3-KETOBUTYRATE COENZYME A LIGASE ; AA_TRANSFER_CLASS_2 ; Aminotran_1_2 ; PLP-dependent transferases	GO:0005743_C_mitochondrial inner membrane; GO:0005739_C_mitochondrion; GO:0007067_P_mitosis;	K00639_00260_Glycine, serine and threonine metabolism;	2.3.1.29_GlycineC-acetyltransferase.;
MGC06835	2	547	Centrin-2	EF_HAND_1 ; CENTRIN ; EF_HAND_2 ; O49999_MARVE_O49999; ; EF-hand ; CALCIUM BINDING PROTEIN ; efhand	GO:0005622_C_intracellular; GO:0005509_F_calcium ion binding; GO:0005515_F_protein binding; GO:0005813_C_centrosome;		2.7.11.1_Non-specificserine/threonineprotein kinase.;
MGC06843	1	619					
MGC06851	1	579	Ubiquinol-cytochrome c reductase complex chaperone CBP3 homolog	Ubiq_cyt_C_chap ; BASIC FGF-REPRESSED ZIC BINDING PROTEIN HOMOLOG (ZIC3-BINDING PROTEIN)	GO:0016023_C_cytoplasmic membrane-bounded vesicle; GO:0005515_F_protein binding;		
MGC06854	1	656	Fibrinogen C domain-containing protein 1	FIBRINOGEN AND FIBRONECTIN ; Fibrinogen C-terminal domain-like ; Fibrinogen_C	GO:0007528_P_neuromuscular junction development; GO:0005604_C_basement membrane; GO:0005576_C_extracellular region;	K06252_04510_Focal adhesion; K06252_04512_ECM-receptor interaction;	
MGC06855	1	458	TFIIH basal transcription factor complex helicase subunit	DNA REPAIR HELICASE RAD3/XP-D ; DNA REPAIR DEAD HELICASE RAD3/XP-D SUBFAMILY MEMBER	GO:0006917_P_induction of apoptosis; GO:0006283_P_transcription-coupled nucleotide-excision repair; GO:0005515_F_protein binding; GO:0043139_F_5{prime}-3{prime} DNA helicase activity;	K10844_03420_Nucleotide excision repair;	
MGC06859	1	426	Brain-specific angiogenesis inhibitor 1	TSP_1 ; THROMBOSPONDIN 2 ; TSP-1 type 1 repeat ; THROMBOSPONDIN ; TSP1REPEAT ; TSP1	GO:0018996_P_molting cycle, collagen and cuticulin-based cuticle; GO:0040011_P_locomotion; GO:0010171_P_body morphogenesis; GO:0002119_P_nematode larval development; GO:0005515_F_protein binding; GO:0040007_P_growth;	K04659_04350_TGF-beta signaling pathway; K04659_04510_Focal adhesion; K04659_04512_ECM-receptor interaction;	3.4.24.21_Astacin.;
MGC06860	4	1326	Caspase-3	Caspase-like ; IL1BCENZYME ; CASPASE_P10 ; CASPASE_P20 ; CASPASE_CYS ; Peptidase_C14 ; DEATH domain ; CASPASE RELATED			3.4.22.56_Caspase-3.;
MGC06866	1	588	Lactose-binding lectin l-2	C-type lectin-like ; C_TYPE_LECTIN_1 ; C-TYPE LECTIN SUPERFAMILY MEMBER ; Lectin_C ; GALACTOSE-SPECIFIC C-TYPE LECTIN ; C_TYPE_LECTIN_2			
MGC06868	1	615	DnaJ homolog subfamily A member 2	ZF_CR ; DnaJ/Hsp40 cysteine-rich domain ; HSP40, SUBFAMILY A, MEMBERS 1,2,4 ; DNAJ/HSP40 ; HSP40/DnaJ peptide-binding domain ; DNAJPROTEIN ; DNAJ_2 ; DnaJ ; DnaJ_CXXCXGXG ; DNAJ_1 ; Chaperone J-domain			

MGC06876	2	729	LIM domain-binding protein 3	LIM ; LIM DOMAIN CONTAINING PROTEIN ; LIM_DOMAIN_2 ; Q7PTE3_EEEEE_Q7PTE3; ; Glucocorticoid receptor-like (DNA-binding domain) ; LIM_DOMAIN_1		K05760_04370_VEGF signaling pathway; K05760_04510_Focal adhesion; K05760_04670_Leukocyte transendothelial migration; K05760_04810_Regulation of actin cytoskeleton; K04511_04310_Wnt signaling pathway;	2.7.11.1_Non-specificserine/threonineproteinkinase.;
MGC06892	1	666	Peroxisomal biogenesis factor 19	PEROXISOMAL FARNESYLATED PROTEIN ; Pex19			
MGC06894	1	629					
MGC06898	2	1138	Dopamine beta-hydroxylase	DOPAMINE BETA HYDROXYLASE RELATED ; PHM/PNGase F ; Scorpion toxin-like ; Cu2_monoox_C	GO:0005737_C_cytoplasm; GO:0003824_F_catalytic activity; GO:0005625_C_soluble fraction; GO:0005576_C_extracellular region; GO:0005624_C_membrane fraction; GO:0042583_C_chromaffin granule; GO:0007268_P_synaptic transmission;	K00503_00350_Tyrosine metabolism;	1.14.17.1_Dopaminebeta-monooxygenase.;
MGC06909	5	760	WASH complex subunit CCDC53	PROTEIN AD-016-RELATED ; coiled-coil	GO:0005515_F_protein binding;		
MGC06914	1	674		PH ; PH_DOMAIN ; PH domain-like			
MGC06918	1	627	Collagen alpha-1(XI) chain	CCDA_CAEEL_P35800; COLLAGEN ALPHA CHAIN ; Collagen ; COLLAGEN ALPHA CHAIN, TYPE IV		K06237_04510_Focal adhesion; K06237_04512_ECM-receptor interaction; K06237_05222_Small cell lung cancer; K06236_04510_Focal adhesion; K06236_04512_ECM-receptor interaction;	
MGC06920	2	670	Soma ferritin	FERRITIN_LIKE ; FERRITIN ; FERRITIN LIGHT CHAIN ; Ferritin ; Ferritin-like		K00522_00860_Porphyrin and chlorophyll metabolism;	1.16.3.1_Ferroxidase.;
MGC06927	2	657	Mitogen-activated protein kinase scaffold protein 1	Roadblock/LC7 domain ; MAPKK1_Int ; MITOGEN-ACTIVATED PROTEIN KINASE KINASE 1 INTERACTING PROTEIN 1	GO:0000186_P_activation of MAPKK activity; GO:0005770_C_late endosome; GO:0019209_F_kinase activator activity;	K04370_04010_MAPK signaling pathway;	
MGC06936	1	643	Serine/threonine-protein kinase 10	Protein kinase-like (PK-like) ; Pkinase ; Q8NC04_HUMAN_Q8NC04; ; MAPKK-RELATED SERINE/THREONINE PROTEIN KINASES ; POLO-LIKE KINASE KINASE 1 ; PROTEIN_KINASE_DOM	GO:0004674_F_protein serine/threonine kinase activity;		2.7.11.1_Non-specificserine/threonineproteinkinase.;
MGC06939	3	748	Fibrinogen C domain-containing protein 1	FIBRINOGEN AND FIBRONECTIN ; Fibrinogen C-terminal domain-like ; Fibrinogen_C ; FIBRIN_AG_C_DOMAIN ; FICOLIN	GO:0046625_F_sphingolipid binding; GO:0030198_P_extracellular matrix organization and biogenesis; GO:0045121_C_membrane raft; GO:0009986_C_cell surface;	K06252_04510_Focal adhesion; K06252_04512_ECM-receptor interaction;	
MGC06940	2	675	Complement C1q-like protein 3	C1q ; C1Q-RELATED FACTOR ; COLLAGEN ALPHA CHAIN ; COMPLEMNTC1Q ; TNF-like ; C1Q	GO:0005581_C_collagen; GO:0001501_P_skeletal development;	K03987_04610_Complement and coagulation cascades; K03987_05010_Alzheimers disease;	
MGC06941	1	526	WD repeat-containing protein 65	WD_REPEATS_2 ; WD40 PROTEIN-RELATED ; WD40 REPEAT PROTEIN ; WD40 repeat-like ; coiled-coil ; WD_REPEATS_REGION			
MGC06945	1	650	Collagen alpha-1(VIII) chain	C1q ; GLIACOLIN-RELATED ; CEREBELLIN-RELATED ; COMPLEMNTC1Q ; TNF-like ; C1Q			

MGC06955	3	775	Proteasome subunit beta type-3	N-terminal nucleophile aminohydrolases (Ntn hydrolases) ; Proteasome ; PROTEASOME_B ; PROTEASOME SUBUNIT ALPHA/BETA ; PROTEASOME SUBUNIT BETA TYPE 3		K02735_03050_Proteasome;	3.4.25.1_Proteasomeendopeptidasecomplex.;
MGC06962	2	748	Fucolectin-6	Galactose-binding domain-like			
MGC06967	1	524	C-type lectin domain family 4 member M	C-type lectin-like ; C-TYPE LECTIN SUPERFAMILY MEMBER ; Lectin_C ; GALACTOSE-SPECIFIC C-TYPE LECTIN ; C_TYPE_LECTIN_2			
MGC06972	1	639	Receptor-type tyrosine-protein phosphatase F	PROTEIN-TYROSINE PHOSPHATASE ; TYR_PHOSPHATASE_PTP ; PRTYPHPHTASE ; (Phosphotyrosine protein) phosphatases II ; Y_phosphatase		K05698_04520_Adherens junction; K05695_04514_Cell adhesion molecules (CAMs); K05695_04520_Adherens junction; K05695_04910_Insulin signaling pathway;	3.1.3.48_Protein-tyrosine-phosphatase.;
MGC06973	2	701	CD209 antigen-like protein E	C-type lectin-like ; C_TYPE_LECTIN_1 ; C-TYPE LECTIN SUPERFAMILY MEMBER ; Lectin_C ; GALACTOSE-SPECIFIC C-TYPE LECTIN ; ANTIFREEZEII ; C_TYPE_LECTIN_2	GO:0005537_F_mannose binding;		
MGC06976	1	608	Stress-induced-phosphoprotein 1	TPR_1 ; TPR ; STRESS-INDUCED-PHOSPHOPROTEIN 1 (STI1) (HSC70/HSP90-ORGANIZING PROTEIN) ; TPR-like ; coiled-coil ; TPR REPEAT CONTAINING PROTEIN ; TPR_REGION	GO:0005515_F_protein binding; GO:0006950_P_response to stress; GO:0005634_C_nucleus; GO:0005794_C_Golgi apparatus;		3.1.3.16_Phosphoproteinphosphatase.;
MGC06978	1	627	Leucine-rich repeat transmembrane neuronal protein 3	LRR_1 ; SLIT PROTEIN ; L domain-like ; LEURICHRPT ; LEUCINE-RICH TRANSMEMBRANE PROTEINS		K04306_04080_Neuroactive ligand-receptor interaction;	1.11.1.7_Peroxidase.;
MGC06979	4	1598	Mesenchyme-specific cell surface glycoprotein				
MGC06989	2	970	Dynein heavy chain 5, axonemal	DHC_N1 ; coiled-coil	GO:0005858_C_axonemal dynein complex; GO:0001539_P_ciliary or flagellar motility; GO:0003777_F_microtubule motor activity;		
MGC06994	4	1073					
MGC06995	1	534		C-type lectin-like ; Lectin_C ; C-TYPE LECTIN PROTEINS ; C_TYPE_LECTIN_2			
MGC06999	1	639	Transforming growth factor-beta-induced protein ig-h3	FAS1 ; PERIOSTIN-RELATED ; PERIOSTIN (PN) (OSTEOBLAST-SPECIFIC FACTOR 2) (OSF-2) ; Fasciclin ; FAS1 domain	GO:0007155_P_cell adhesion; GO:0005615_C_extracellular space; GO:0005578_C_proteinaceous extracellular matrix;		
MGC07002	1	654		TNF-like			
MGC07003	1	650	Complement C1q-like protein 4	C1q ; C1Q-RELATED FACTOR ; COLLAGEN ALPHA CHAIN ; COMPLEMNTC1Q ; TNF-like ; coiled-coil ; C1Q			
MGC07010	3	1007	Guanine nucleotide-binding protein subunit beta-2-like 1	WD_REPEATS_2 ; GPROTEINBRPT ; WD40 repeat-like ; Q8T6T3_SCHMA_Q8T6T3; ; RECEPTOR FOR ACTIVATED PROTEIN KINASE C (RACK1) ; WD_REPEATS_1 ; WD_REPEATS_REGION ; WD40	GO:0005737_C_cytoplasm; GO:0043025_C_cell soma;	K01062_00565_Ether lipid metabolism;	2.7.11.1_Non-specificserine/threonineproteinkinase.; 2.3.1.48_Histoneacetyltransferase.; 2.7.11.7_[Myosinheavy-chain]kinase.;
MGC07012	1	598	Mothers against decapentaplegic homolog 4	SMAD MH1 domain ; SMAD4 ; SMAD ; MH1			
MGC07013	1	581	60S ribosomal protein L23a	Ribosomal proteins L23 and L15e ; Ribosomal_L23 ; RIBOSOMAL_L23 ; Ribosomal_L23eN ; 60S RIBOSOMAL PROTEIN L23A		K02893_03010_Ribosome;	

MGC07014	1	541	Probable G-protein coupled receptor 33	G_PROTEIN_RECEP_F1_2 ; G-PROTEIN COUPLED RECEPTOR ; GPCRRHODOPSN ; 7tm_1 ; Family A G protein-coupled receptor-like	GO:0016500_F_protein-hormone receptor activity; GO:0016021_C_integral to membrane; GO:0007186_P_G-protein coupled receptor protein signaling pathway;	K05050_04060_Cytokine-cytokine receptor interaction; K05050_05120_Epithelial cell signaling in Helicobacter pylori infection; K04173_04080_Neuroactive ligand-receptor interaction; K04172_04080_Neuroactive ligand-receptor interaction;	
MGC07015	2	877	Protein TANC1	ANKYRIN ; Ank ; ANKYRIN REPEAT-CONTAINING ; P-loop containing nucleoside triphosphate hydrolases ; ANK_REPEAT ; ANK_REP_REGION ; HSPC200 ; Ankyrin repeat	GO:0005515_F_protein binding;	K08803_05219_Bladder cancer;	2.7.11.1_Non-specificserine/threonineproteinkinase.;
MGC07024	3	743	Protein dachsous	Cadherin-like ; CADHERIN_2 ; Cadherin ; CADHERIN-RELATED ; CADHERIN ; CADHERIN_1	GO:0005624_C_membrane fraction;		
MGC07026	1	618	Phosphoglycolate phosphatase	4-NITROPHENYLPHOSPHATASE-RELATED ; HAD-like ; PYRIDOXAL-5-PHOSPHATE PHOSPHATASE		K01101_00361_gamma-Hexachlorocyclohexane degradation; K01091_00630_Glyoxylate and dicarboxylate metabolism;	3.1.3.41_4-nitrophenylphosphatase.; 3.1.3.18_Phosphoglycolatephosphatase.;
MGC07032	2	609	Tetraspanin-9				
MGC07038	2	1019	Collectin-12	C-type lectin-like ; ASIALOGLYCOPROTEIN RECEPTOR ; C-TYPE LECTIN SUPERFAMILY MEMBER ; Lectin_C ; C_TYPE_LECTIN_2			
MGC07041	1	602	Peptidyl-prolyl cis-trans isomerase 6	CSA_PPIASE_1 ; Cyclophilin-like ; CSAPPISMRASE ; CYCLOPHILIN ; Pro_isomerase ; PEPTIDYL-PROLYL CIS-TRANS ISOMERASE (CYCLOPHILIN) ; CSA_PPIASE_2	GO:0005515_F_protein binding;		5.2.1.8_Peptidylprolylisomerase.;
MGC07055	2	599	Calmodulin	EF_HAND_1 ; CALMODULIN ; EF_HAND_2 ; PARVALBUMIN ; CALF_NAEGR_P53440; ; EF-hand ; CALCIUM BINDING PROTEIN ; efhand			2.7.11.1_Non-specificserine/threonineproteinkinase.;
MGC07056	5	672		C-type lectin-like			
MGC07069	1	576	Fibrinogen-like protein A	FIBRINOGEN AND FIBRONECTIN ; Fibrinogen C-terminal domain-like ; Fibrinogen_C	GO:0007528_P_neuromuscular junction development; GO:0005604_C_basement membrane; GO:0005576_C_extracellular region;	K06252_04510_Focal adhesion; K06252_04512_ECM-receptor interaction;	
MGC07072	1	688	Malignant T cell-amplified sequence 1	PUA ; PUA domain-like ; unchar_dom_2: domain ; MCT-1 PROTEIN	GO:0005515_F_protein binding;		
MGC07073	1	546	Complement C4 (Fragments)	COMPLEMENT C3 ; Terpenoid cyclases/Protein prenyltransferases ; MACROGLOBULIN/COMPLEMENT ; A2M_comp			
MGC07080	1	648	Peptidyl-prolyl cis-trans isomerase CWC27 homolog	CSA_PPIASE_1 ; Cyclophilin-like ; CSAPPISMRASE ; CYCLOPHILIN ; Pro_isomerase ; PEPTIDYL-PROLYL CIS-TRANS ISOMERASE (CYCLOPHILIN) ; CSA_PPIASE_2	GO:0005634_C_nucleus;		5.2.1.8_Peptidylprolylisomerase.;
MGC07082	1	573	Interferon alpha-inducible protein 27-like protein 2	INTERFERON-INDUCED 6-16/INTERFERON STIMULATED GENE 12 ; Ifi-6-16 ; INTERFERON STIMULATED GENE 12			
MGC07083	2	1020	Homeobox protein cut-like 1	CCAAT DISPLACEMENT PROTEIN-RELATED ; CCAAT DISPLACEMENT PROTEIN (CDP) (CUT-LIKE 1) ; coiled-coil			
MGC07085	1	566	Uncharacterized protein ZC395.10	TELOMERASE-BINDING PROTEIN P23 (HSP90 CO-CHAPERONE) ; HSP20-like chaperones			5.3.99.3_Prostaglandin-Esynthase.;

MGC07088	2	812	Kielin/chordin-like protein	Pacifastin_I ; VWFC_2 ; PMP inhibitors	GO:0030513_P_positive regulation of BMP signaling pathway; GO:0005615_C_extracellular space; GO:0005515_F_protein binding;		
MGC07093	1	566	Peptidase inhibitor 16	SCP ; CRISP_1 ; V5ALLERGEN ; CYSTEINE-RICH SECRETORY PROTEIN (CRISP/SCP/TPX1)-RELATED ; V5TPXLIKE ; PR-1-like ; Q40597_TOBAC_Q40597; ; CRISP_2 ; CRISP SUBFAMILY GLIOMA PATHOGENESIS-RELATED PROTEIN-RELATED			
MGC07095	2	723	Proprotein convertase subtilisin/kexin type 5 (Fragment)	SUBTILISIN/KEXIN-RELATED SERINE PROTEASE ; Peptidase_S8 ; Subtilisin-like ; PROPROTEIN CONVERTASE SUBTILISIN/KEXIN TYPE 4, FURIN ; P_proprotein ; Galactose-binding domain-like	GO:0030141_C_secretory granule; GO:0005794_C_Golgi apparatus;		3.4.21.75_Furin.;
MGC07097	1	530	Collagen alpha-1(XII) chain	VWFA ; VON WILENBRAND FACTOR RELATED ; vWA-like ; VWA			
MGC07108	1	632		TRANSCRIPTION FACTOR L2 ; LIM ; LIM DOMAIN CONTAINING PROTEIN ; LIM_DOMAIN_2 ; Glucocorticoid receptor-like (DNA-binding domain) ; LIM_DOMAIN_1 ; Q6ICK4_HUMAN_Q6ICK4;			
MGC07111	1	628	Rootletin	CENTROSOMAL PROTEIN 2 ; coiled-coil ; CILIARY ROOTLET COILED-COIL, ROOTLETIN			
MGC07116	2	897	Cathepsin D	ASPARTIC PROTEASE - RELATED ; Acid proteases ; ASPARTYL PROTEASES ; Asp			3.4.23.5_CathepsinD.;
MGC07121	1	587	Protein sidekick-1	Immunoglobulin ; IG_LIKE ; I-set ; TITIN ; coiled-coil	GO:0008307_F_structural constituent of muscle; GO:0040011_P_locomotion; GO:0005515_F_protein binding; GO:0006936_P_muscle contraction; GO:0015629_C_actin cytoskeleton;	K00907_04020_Calcium signaling pathway; K00907_04510_Focal adhesion; K00907_04810_Regulation of actin cytoskeleton; K06759_04514_Cell adhesion molecules (CAMs);	2.7.11.1_Non-specificserine/threonineproteinkinase.; 2.7.11.18_[Myosinlight-chain]kinase.;
MGC07122	1	573	Lamin-L(II)	INTERMEDIATE FILAMENT ; LAMIN ; Filament ; coiled-coil			
MGC07126	2	776	Cartilage matrix protein	VWFA ; VON WILENBRAND FACTOR RELATED ; vWA-like ; VWA ; VWFADOMAIN			
MGC07136	1	731	Ataxin-2				
MGC07148	1	626	Kinase D-interacting substrate of 220 kDa	ANK REPEAT-CONTAINING ; ANKYRIN ; Ank ; ANKYRIN REPEAT-CONTAINING ; ANK_REPEAT ; ANK_REP_REGION ; Ankyrin repeat		K06653_04111_Cell cycle - yeast;	2.4.2.30_NAD(+)ADP-ribosyltransferase.; 3.1.1.5_Lysophospholipase.; 2.1.1.43_Histone-lysineN-methyltransferase.; 3.5.1.1_Asparaginase.; 3.1.1.47_1-alkyl-2-acetylglycerophosphocholineesterase.;
MGC07150	1	588	Protein lyl-1	T-CELL ACUTE LYMPHOCYTIC LEUKEMIA 1/STEM CELL LEUKEMIA PROTEIN (TAL-1/SCL) ; T-CELL ACUTE LYMPHOCYTIC LEUKEMIA/STEM CELL LEUKEMIA-RELATED ; HLH ; HLH, helix-loop-helix DNA-binding domain	GO:0003677_F_DNA binding; GO:0005515_F_protein binding; GO:0008283_P_cell proliferation; GO:0005634_C_nucleus;		
MGC07153	1	612	Lysosomal Pro-X carboxypeptidase	PROTEASE S28 PRO-X CARBOXYPEPTIDASE-RELATED ; Peptidase_S28 ; alpha/beta-Hydrolases ; PROLYLCARBOXYPEPTIDASE/PRCP	GO:0004185_F_serine carboxypeptidase activity;		3.4.14.2_Dipeptidyl-peptidaseII.; 3.4.16.2_LysosomalPro-Xaacarboxypeptidase.;
MGC07154	1	518	Angiopoietin-2	FIBRINOGEN AND FIBRONECTIN ; Fibrinogen C-terminal domain-like ; Fibrinogen_C		K06252_04510_Focal adhesion; K06252_04512_ECM-receptor interaction;	

MGC07157	1	574	Plasminogen	EGF/Laminin ; KRINGLE_1 ; Q6PBA6_BRARE_Q6PBA6; ; SERINE PROTEASE-RELATED ; Kringle-like ; PLASMINOGEN ; KRINGLE_2 ; KRINGLE ; EGF_2 ; Kringle		K01315_04080_Neuroactive ligand-receptor interaction; K01315_04610_Complement and coagulation cascades;	3.4.21.7_Plasmin.;
MGC07158	1	610	Putative fungistatic metabolite	WSC		K00771_00532_Chondroitin sulfate biosynthesis; K00771_01030_tba;	2.7.10.1_Receptorprotein-tyrosinekinase.; 2.4.2.26_Proteinxylosyltransferase.;
MGC07160	2	727	ADP-ribosylation factor	ADP-RIBOSYLATION FACTOR, ARF ; P-loop containing nucleoside triphosphate hydrolases ; RASTRNSFRMNG ; Arf ; small_GTP: small GTP-binding protein domain ; SAR1GTPBP ; ADP RIBOSYLATION FACTOR-RELATED			
MGC07164	1	549	BTB/POZ domain-containing protein 2	POZ domain ; KELCH-RELATED PROTEIN ; BTB	GO:0005515_F_protein binding;		
MGC07165	2	749		TNF-like ; coiled-coil			
MGC07166	1	606	Ubiquitin carboxyl-terminal hydrolase 32	UBIQUITIN SPECIFIC PROTEASE 32 ; UBIQUITIN SPECIFIC PROTEASE FAMILY C19-RELATED ; EF-hand			1.6.3.1_NAD(P)Hoxidase.; 3.1.2.15_Ubiquitinthiolesterase.;
MGC07173	1	528	Cathepsin D	Acid proteases ; A1_Propeptide ; CATHEPSIN D ; ASPARTYL PROTEASES ; ASP_PROTEASE ; Asp			3.4.23.5_CathepsinD.;
MGC07178	1	594	Ligand of Numb protein X 2	gb def: Novel protein similar to vertebrate ligand of numb-protein X (LNX) ; RING/U-box ; TRAF domain-like ; ZF_RING_1 ; ZF_TRAF ; zf-C3HC4 ; ZF_RING_2 ; MULTIPLE PDZ DOMAIN PROTEIN	GO:0051260_P_protein homooligomerization; GO:0005515_F_protein binding; GO:0030165_F_PDZ domain binding;		
MGC07183	1	528	Tetratricopeptide repeat protein 26	TPR-like			
MGC07184	2	601	EMILIN-2				
MGC07193	1	599	Protein SEC13 homolog	PROTEIN TRANSPORT PROTEIN SEC13 ; WD_REPEATS_2 ; GPROTEINBRPT ; WD40 repeat-like ; Q7T2E1_BRARE_Q7T2E1; ; PROTEIN TRANSPORT PROTEIN SEC13-RELATED ; WD_REPEATS_REGION ; WD40	GO:0035293_P_chitin-based larval cuticle pattern formation; GO:0006886_P_intracellular protein transport; GO:0006888_P_ER to Golgi vesicle-mediated transport; GO:0008363_P_larval chitin-based cuticle development;	K04508_04310_Wnt signaling pathway; K02084_04115_p53 signaling pathway; K02084_04210_Apoptosis; K02084_05222_Small cell lung cancer;	2.3.1.48_Histoneacetyltransferase.;
MGC07197	1	635	RING finger protein 151	RING/U-box ; zf-C3HC4 ; SUBZF_RING_2			
MGC07198	1	552	Serine/threonine-protein kinase PLK2	Protein kinase-like (PK-like) ; Q8N7M6_HUMAN_Q8N7M6; ; PROTEIN_KINASE_ATP ; Pkinase ; POLO-LIKE KINASE RELATED ; CALCIUM/CALMODULIN-DEPENDENT PROTEIN KINASE-RELATED ; PROTEIN_KINASE_DOM		K06631_04110_Cell cycle; K06631_04914_Progesterone-mediated oocyte maturation;	2.7.11.21_Polokinase.;
MGC07206	1	673	Anosmin-1	TSP_1 ; ADAMTS-17, 19 ; 4_DISULFIDE_CORE ; ADAMTS (A DISINTEGRIN AND METALLOPROTEASE WITH THROMBOSPONDIN MOTIFS) PROTEASE M12B-RELATED ; TSP-1 type 1 repeat ; WAP ; 4DISULPHCORE ; Galactose-binding domain-like ; TSP1REPEAT ; TSP1 ; Elafin-like		K04596_04115_p53 signaling pathway;	
MGC07220	1	557	Collagen-like protein 2	COLLAGEN ALPHA CHAIN ; BCLA PROTEIN ; Q74HW0_LACJO_Q74HW0;			
MGC07224	1	677	Thrombospondin-1	TSP_1 ; TSP-1 type 1 repeat ; THROMBOSPONDIN ; THROMBOSPONDIN 1 ; TSP1REPEAT ; TSP1		K04659_04350_TGF-beta signaling pathway; K04659_04510_Focal adhesion; K04659_04512_ECM-receptor interaction; K07521_04360_Axon guidance;	

MGC07226	3	828	Vascular endothelial growth factor D	Cystine-knot cytokines ; PDGF ; PDGF_2 ; PLATELET-DERIVED GROWTH FACTOR	GO:0005737_C_cytoplasm; GO:0008201_F_heparin binding; GO:0007298_P_border follicle cell migration; GO:0005102_F_receptor binding; GO:0005515_F_protein binding; GO:0035099_P_hemocyte migration; GO:0030031_P_cell projection biogenesis;		
MGC07228	3	541	Perlucin	C-type lectin-like ; ASIALOGLYCOPROTEIN RECEPTOR ; C_TYPE_LECTIN_1 ; C-TYPE LECTIN SUPERFAMILY MEMBER ; Lectin_C ; ANTIFREEZEII ; C_TYPE_LECTIN_2			
MGC07229	1	476	von Willebrand factor C domain-containing protein 2-like	VWC ; VWFC_2 ; EXTRACELLULAR MATRIX GLYCOPROTEIN RELATED ; VWFC_1		K06236_04510_Focal adhesion; K06236_04512_ECM-receptor interaction;	
MGC07233	4	736	Proteasome subunit alpha type-3	N-terminal nucleophile aminohydrolases (Ntn hydrolases) ; PROTEASOME SUBUNIT ALPHA TYPE 3 ; Proteasome ; PROTEASOME SUBUNIT ALPHA/BETA ; PROTEASOME_A	GO:0005737_C_cytoplasm; GO:0000502_C_proteasome complex; GO:0005515_F_protein binding; GO:0005634_C_nucleus;	K02727_03050_Proteasome;	3.4.25.1_Proteasomeendopeptidasecomplex.;
MGC07242	1	519	Nuclear factor NF-kappa-B p105 subunit	REL_2 ; ANKYRIN REPEAT-CONTAINING ; p53-like transcription factors ; REL_1 ; RHD ; NUCLEAR FACTOR NF-KAPPA-B P105 SUBUNIT			
MGC07251	1	473	Tetratricopeptide repeat protein 12	ARM repeat ; TETRATRICOPEPTIDE REPEAT DOMAIN 12 ; TPR REPEAT CONTAINING PROTEIN			
MGC07254	1	733	Sprouty-related, EVH1 domain-containing protein 2	SPROUTY PROTEIN EVH1 DOMAIN CONTAINING PROTEIN 3 ; VASODILATOR-STIMULATED PHOSPHOPROTEIN/SPROUTY PROTEIN EVH1 DOMAIN CONTAINING PROTEIN 3 ; PH domain-like ; WH1		K04703_04630_Jak-STAT signaling pathway;	
MGC07264	1	767	Probable aminopeptidase NPEPL1	Zn-dependent exopeptidases ; Peptidase_M17 ; CYTOSOL_AP ; LEUCINE AMINOPEPTIDASE-RELATED			3.4.11.1_Leucylaminopeptidase.;
MGC07267	1	735	SLIT-ROBO Rho GTPase-activating protein 3	C-terminal domain of RNA polymerase alpha subunit ; tRNA-binding arm ; FCH ; coiled-coil ; SLIT-ROBO RHO GTPASE ACTIVATING PROTEIN RELATED		K07526_04360_Axon guidance;	
MGC07272	1	749	Midline-1	RING/U-box ; ZF_RING_1 ; zf-C3HC4 ; ZF_RING_2 ; ZF_BBOX ; RING FINGER-CONTAINING ; zf-B_box ; RING FINGER-CONTAINING PROTEIN-RELATED		K08285_04120_Ubiquitin mediated proteolysis;	
MGC07274	2	799	Uncharacterized protein sll1388	UNVRSLSTRESS ; Adenine nucleotide alpha hydrolases-like ; Usp			
MGC07276	1	693	Macrophage mannose receptor 1	C-type lectin-like ; C-TYPE LECTIN SUPERFAMILY MEMBER ; Lectin_C ; GALACTOSE-SPECIFIC C-TYPE LECTIN ; C_TYPE_LECTIN_2			
MGC07277	2	1206	Death-associated protein kinase 1	ANKYRIN ; Ank ; ANKYRIN REPEAT-CONTAINING ; P-loop containing nucleoside triphosphate hydrolases ; ANK_REPEAT ; ANK_REP_REGION ; Ankyrin repeat		K08803_05219_Bladder cancer;	2.4.2.30_NAD(+)ADP-ribosyltransferase.; 2.7.11.1_Non-specificserine/threonineproteinkinase.; 2.1.1.43_Histone-lysineN-methyltransferase.;
MGC07284	1	638		G_PROTEIN_RECEP_F1_2 ; G-PROTEIN COUPLED RECEPTOR ; GPCRRHODOPSN ; 7tm_1 ; Family A G protein-coupled receptor-like			

MGC07294	1	686	Thioredoxin domain-containing protein C2F3.12c	Thioredoxin-like ; PHOSDUCIN-RELATED ; THIOREDOXIN DOMAIN CONTAINING PROTEIN 9-RELATED ; coiled-coil	GO:0005829_C_cytosol ; GO:0005634_C_nucleus;		
MGC07296	1	783	Tyrosine aminotransferase	SUBGROUP I AMINOTRANSFERASE RELATED ; TYROSINE AMINOTRANSFERASE ; ACCSYNTHASE ; Aminotran_1_2 ; PLP-dependent transferases		K00815_00271_Methionine metabolism; K00815_00350_Tyrosine metabolism; K00815_00360_Phenylalanine metabolism; K00815_00400_Phenylalanine, tyrosine and tryptophan biosynthesis; K00815_00401_Novobiocin biosynthesis; K00815_00950_Alkaloid biosynthesis I;	2.6.1.5_Tyrosinetransaminase.;
MGC07297	2	596	Aggrecan core protein	C-type lectin-like ; FRAS1 RELATED EXTRACELLULAR MATRIX PROTEIN 1 ; C-TYPE LECTIN SUPERFAMILY MEMBER ; Lectin_C ; C_TYPE_LECTIN_2			
MGC07298	1	701	Heavy metal-binding protein HIP				
MGC07300	1	769	Translocation protein SEC63 homolog	DNAJ/HSP40 ; DNAJ_2 ; DnaJ ; DNAJ-RELATED ; Chaperone J-domain			
MGC07305	2	761	Ras-related C3 botulinum toxin substrate 1	P-loop containing nucleoside triphosphate hydrolases ; RASTRNSFRMNG ; RAS-RELATED PROTEIN RAC ; RAS-RELATED GTPASE ; small_GTP: small GTP-binding protein domain ; Ras			2.7.11.1_Non-specificserine/threonineproteinkinase.;
MGC07308	1	789	Probable E3 ubiquitin-protein ligase MYCBP2	HIGHWIRE ; Galactose-binding domain-like			
MGC07312	1	801	MAP kinase-interacting serine/threonine-protein kinase 1	Protein kinase-like (PK-like) ; Pkinase ; MAP KINASE-INTERACTING SERINE/THREONINE KINASE ; CALCIUM/CALMODULIN-DEPENDENT PROTEIN KINASE-RELATED ; O82107_MAIZE_O82107;		K04372_04010_MAPK signaling pathway; K04372_04910_Insulin signaling pathway;	2.7.11.1_Non-specificserine/threonineproteinkinase.;
MGC07322	1	712	Baculoviral IAP repeat-containing protein 4	BIR_REPEAT_1 ; BIR ; INHIBITOR OF APOPTOSIS ; Inhibitor of apoptosis (IAP) repeat ; INHIBITOR OF APOPTOSIS PROTEIN 1 AND 2, IAP1, IAP2 ; BIR_REPEAT_2		K04725_04120_Ubiquitin mediated proteolysis; K04725_04210_Apoptosis; K04725_04510_Focal adhesion; K04725_05222_Small cell lung cancer;	
MGC07337	1	736	Serine protease inhibitor dipetalogastin (Fragment)	Kazal_2 ; KAZAL ; SERINE PROTEASE INHIBITOR ; Kazal-type serine protease inhibitors		K06254_04512_ECM-receptor interaction;	
MGC07340	1	724	FCH domain only protein 2	PROLINE-SERINE-THREONINE PHOSPHATASE INTERACTING PROTEIN 1 ; FCH ; coiled-coil			
MGC07341	1	720	Nuclear pore complex protein Nup85	Nucleopor_Nup85 ; FROUNT PROTEIN-RELATED ; FROUNT PROTEIN (PERICENTRIN 1)	GO:0019903_F_protein phosphatase binding; GO:0007165_P_signal transduction; GO:0007155_P_cell adhesion; GO:0003779_F_actin binding; GO:0005826_C_contractile ring; GO:0005515_F_protein binding; GO:0000910_P_cytokinesis; GO:0001725_C_stress fiber; GO:0048246_P_macrophage chemotaxis; GO:0030032_P_lamellipodium biogenesis; GO:0006935_P_chemotaxis;		
MGC07346	1	594		INTERLEUKIN_10			
MGC07348	1	717	Fibrinogen-like protein A	FIBRINOGEN AND FIBRONECTIN ; Fibrinogen C-terminal domain-like ; Fibrinogen_C		K06252_04510_Focal adhesion; K06252_04512_ECM-receptor interaction;	
MGC07351	1	613	Heavy metal-binding protein HIP	C1q ; CEREBELLIN-RELATED ; COMPLEMNTC1Q ; TNF-like ; coiled-coil ; C1Q			

MGC07352	1	710	Uncharacterized protein yxiE	UNVRSLSTRESS ; Adenine nucleotide alpha hydrolases-like ; coiled-coil ; Usp			
MGC07361	1	759		coiled-coil			
MGC07362	1	717	Sorting nexin-2	SORTING NEXIN 2 ; SORTING NEXIN ; PX ; PX domain			
MGC07367	1	744	Fibropellin-1	EGF/Laminin ; Immunoglobulin ; EGF-LIKE DOMAIN PROTEIN ; IG_LIKE ; EGF ; 2FE2S_FER_1 ; EGF_3 ; ASX_HYDROXYL ; CRUMBS(D.MELANOGASTER) RELATED ; EGF_1 ; EGF_2 ; EGFBLOOD ; EGF_CA		K06051_04330_Notch signaling pathway; K02599_04320_Dorso-ventral axis formation; K02599_04330_Notch signaling pathway;	3.4.21.22_Coagulationfactor IXa.; 2.7.10.1_Receptorprotein-tyrosinekinase.;
MGC07368	1	672	Peripheral-type benzodiazepine receptor-associated protein 1	coiled-coil	GO:0005737_C_cytoplasm; GO:0005739_C_mitochondrion; GO:0030156_F_benzodiazepine receptor binding;		
MGC07369	1	696	Macrophage asialoglycoprotein-binding protein 1	C-type lectin-like ; C-TYPE LECTIN SUPERFAMILY MEMBER ; Lectin_C ; GALACTOSE-SPECIFIC C-TYPE LECTIN ; C_TYPE_LECTIN_2		K06468_04640_Hematopoietic cell lineage;	
MGC07370	1	723	Alpha-2-macroglobulin-P	Terpenoid cyclases/Protein prenyltransferases ; ALPHA-MACROGLOBULIN ; MACROGLOBULIN/COMPLEMENT ; A2M_comp		K03910_04610_Complement and coagulation cascades; K03910_05010_Alzheimers disease;	
MGC07376	1	673	Translation initiation factor IF-2				
MGC07380	2	784	UV excision repair protein RAD23 homolog B	UBIQUITIN_2 ; Ubiquitin-like ; UBA-like ; ubiquitin ; UV EXCISION REPAIR PROTEIN RAD23 ; UBA	GO:0007283_P_spermatogenesis; GO:0006974_P_response to DNA damage stimulus;	K10839_03420_Nucleotide excision repair;	
MGC07386	1	606					
MGC07387	1	722	Probable ATP-dependent RNA helicase DDX41	Q_MOTIF ; DEAD (ASP-GLU-ALA-ASP) BOX POLYPEPTIDE 41 ; P-loop containing nucleoside triphosphate hydrolases ; DEAD BOX ATP-DEPENDENT RNA HELICASE ; coiled-coil	GO:0005515_F_protein binding;	K01529_00500_Starch and sucrose metabolism; K01529_00790_Folate biosynthesis;	
MGC07396	1	754	Prolyl 4-hydroxylase subunit alpha-2	PROLYL 4-HYDROXYLASE ALPHA SUBUNIT ; PROLYL 4-HYDROXYLASE ALPHA SUBUNIT 1, 2 ; coiled-coil ; P4Ha_N		K00472_00330_Arginine and proline metabolism;	1.14.11.2_Procollagen-prolinedioxygenase.;
MGC07397	1	655			GO:0042802_F_identical protein binding; GO:0005515_F_protein binding;		
MGC07398	1	684		C1q ; TNF-like ; coiled-coil ; C1Q			
MGC07399	2	829	Slit homolog 3 protein	LRR_1 ; L domain-like ; LEURICHRPT ; LEUCINE-RICH TRANSMEMBRANE PROTEINS		K06839_04360_Axon guidance;	
MGC07410	1	715	Alpha-(1,3)-fucosyltransferase	Glyco_transf_10 ; ALPHA-(1,3)-FUCOSYLTRANSFERASE		K07633_00602_tba; K07633_01031_tba; K07634_00602_tba; K07634_01031_tba; K00716_00601_Glycosphingolipid biosynthesis - lacto and neolacto series; K00716_00602_tba; K00716_01031_tba; K07635_00602_tba; K07635_01031_tba;	2.4.1.65_3-galactosyl-N-acetylglucosaminide4-alpha-L-fucosyltransferase.;
MGC07411	3	755	Ras-related protein Rab-5A	RAS-RELATED GTPASE ; small_GTP: small GTP-binding protein domain ; SMALL GTPASE RABF AND G, ENTAMOEBA HISTOLYTICA ; Ras ; P-loop containing nucleoside triphosphate hydrolases ; RASTRNSFRMNG		K07887_05030_tba;	
MGC07413	5	772	MAP/microtubule affinity-regulating kinase 3	Protein kinase-like (PK-like) ; PROTEIN_KINASE_ATP ; Pkinase ; SERINE/THREONINE PROTEIN KINASE ; Q8MLJ7_DROME_Q8MLJ7; ; PROTEIN_KINASE_DOM	GO:0005515_F_protein binding;		2.7.11.1_Non-specificserine/threonineproteinkinase.;

MGC07415	1	709	Angiopoietin-4	FIBRINOGEN AND FIBRONECTIN ; Fibrinogen C-terminal domain-like ; Fibrinogen_C	GO:0007492_P_endoderm development; GO:0005172_F_vascular endothelial growth factor receptor binding;		
MGC07418	2	1104	Eukaryotic translation initiation factor 3 subunit I	Q7ZV55_BRARE_Q7ZV55; ; WD_REPEATS_2 ; GPROTEINBRPT ; EUKARYOTIC TRANSLATION INITIATION FACTOR 3 SUBUNIT 2 ; WD40 REPEAT PROTEIN ; WD40 repeat-like ; WD_REPEATS_1 ; WD_REPEATS_REGION ; WD40	GO:0008135_F_translation factor activity, nucleic acid binding; GO:0005852_C_eukaryotic translation initiation factor 3 complex; GO:0005515_F_protein binding; GO:0006446_P_regulation of translational initiation;		2.7.11.1_Non-specificserine/threonineproteinkinase.; 2.3.1.48_Histoneacetyltransferase.;
MGC07424	1	493		C-type lectin-like			
MGC07430	2	724	Collectin-12	C-type lectin-like ; C-TYPE LECTIN SUPERFAMILY MEMBER ; Lectin_C ; GALACTOSE-SPECIFIC C-TYPE LECTIN ; C_TYPE_LECTIN_2			
MGC07433	1	428	Soluble NSF attachment protein	NSF ; TPR-like ; SOLUBLE NSF ATTACHMENT PROTEIN (SNAP) ; ALPHA-SOLUBLE NSF ATTACHMENT PROTEIN (SNAP-ALPHA) ; NSFATTACHMNT			
MGC07434	1	583	Complement C1q-like protein 3	C1q ; GLIACOLIN-RELATED ; CEREBELLIN-RELATED ; COMPLEMNTC1Q ; TNF-like ; C1Q		K03987_04610_Complement and coagulation cascades; K03987_05010_Alzheimers disease;	
MGC07452	1	638	Fibrinogen-like protein 1	FIBRINOGEN AND FIBRONECTIN ; Fibrinogen C-terminal domain-like ; Fibrinogen_C	GO:0007492_P_endoderm development; GO:0005172_F_vascular endothelial growth factor receptor binding;	K08767_03320_PPAR signaling pathway;	
MGC07458	1	772	Uncharacterized protein C6orf168	FAILED AXON CONNECTIONS RELATED ; METAXIN RELATED ; Glutathione S-transferase (GST), C-terminal domain ; Thioredoxin-like			
MGC07459	1	621		G_PROTEIN_RECEP_F1_1			
MGC07461	1	735	26S proteasome non-ATPase regulatory subunit 10				
MGC07462	1	743	Autophagy-related protein 12	Ubiquitin-like ; AUTOPHAGY PROTEIN 12 ; APG12	GO:0005737_C_cytoplasm; GO:0006914_P_autophagy; GO:0006915_P_apoptosis; GO:0006464_P_protein modification process; GO:0005515_F_protein binding;	K08336_04140_Regulation of autophagy;	
MGC07469	1	514	SH3 domain-containing kinase-binding protein 1	SH3-domain ; Q86PF3_DROME_Q86PF3; ; P67PHOX ; SH3 DOMAIN-CONTAINING ; SH3 ; SH3DOMAIN ; SH3_1 ; DAB2-INTERACTING PROTEIN 2		K05729_04810_Regulation of actin cytoskeleton; K05729_05212_Pancreatic cancer;	2.7.11.25_Mitogen-activatedproteinkinasekinasekinase.;
MGC07471	1	332		LECTIN_LEGUME_BETA ; Insulin-like			
MGC07472	1	749					
MGC07477	1	666	3-beta-hydroxysteroid-Delta(8),Delta(7)-isomerase	STEROL ISOMERASE ; EBP	GO:0005789_C_endoplasmic reticulum membrane; GO:0016126_P_sterol biosynthetic process; GO:0000247_F_C-8 sterol isomerase activity; GO:0030097_P_hemopoiesis;	K01824_00100_Biosynthesis of steroids;	5.3.3.5_CholestenolDelta-isomerase.;
MGC07484	2	709	Ficolin-1	FIBRINOGEN AND FIBRONECTIN ; Fibrinogen C-terminal domain-like ; Fibrinogen_C	GO:0008228_P_opsonization; GO:0005529_F_sugar binding; GO:0005509_F_calcium ion binding; GO:0003823_F_antigen binding;	K06252_04510_Focal adhesion; K06252_04512_ECM-receptor interaction;	
MGC07487	1	557					

MGC07490	1	652	Zinc finger homeobox protein 4				
MGC07491	1	744	Aggrecan core protein	C-type lectin-like ; ENDOCYTIC RECEPTOR ENDO180 ; PNCREATITSAP ; C_TYPE_LECTIN_1 ; Lectin_C ; MANNOSE, PHOSPHOLIPASE, LECTIN RECEPTOR RELATED ; ANTIFREEZEII ; C_TYPE_LECTIN_2			
MGC07497	1	672	Aldehyde dehydrogenase, mitochondrial	ALDEHYDE DEHYDROGENASE-RELATED ; ALDEHYDE DEHYDROGENASE ; ALDH-like ; ALDEHYDE_DEHYDR_CYS ; Aldedh			1.2.1.3_Aldehydedehydrogenase(NAD(+)).;
MGC07503	2	589	Fucolectin-6	Galactose-binding domain-like ; coiled-coil ; F5_F8_type_C			
MGC07508	2	965	Nuclear factor 7, brain				
MGC07516	2	827					
MGC07524	1	487	Toll-like receptor 6				
MGC07527	1	622	Histidine-rich protein PFHRP-II				
MGC07535	1	672	Protein toll	TIR ; Toll/Interleukin receptor TIR domain ; TOLL ; LEUCINE-RICH TRANSMEMBRANE PROTEINS		K10159_04620_Toll-like receptor signaling pathway;	
MGC07536	1	754	Low affinity immunoglobulin epsilon Fc receptor	C-type lectin-like ; C-TYPE LECTIN SUPERFAMILY MEMBER ; Lectin_C ; tRNA-binding arm ; coiled-coil ; ANTIFREEZEII ; C_TYPE_LECTIN_2			
MGC07541	1	680	Hepatic lectin	C-type lectin-like ; FRAS1 RELATED EXTRACELLULAR MATRIX PROTEIN 1 ; C_TYPE_LECTIN_1 ; C-TYPE LECTIN SUPERFAMILY MEMBER ; Lectin_C ; C_TYPE_LECTIN_2			
MGC07542	1	543	Chondroitin proteoglycan 2				
MGC07550	1	613	C-type lectin domain family 10 member A	C-type lectin-like ; ASIALOGLYCOPROTEIN RECEPTOR ; C-TYPE LECTIN SUPERFAMILY MEMBER ; Lectin_C ; ANTIFREEZEII ; C_TYPE_LECTIN_2	GO:0008329_F_pattern recognition receptor activity; GO:0006910_P_phagocytosis, recognition; GO:0005886_C_plasma membrane; GO:0005529_F_sugar binding; GO:0030169_F_low-density lipoprotein binding; GO:0006955_P_immune response; GO:0005576_C_extracellular region; GO:0030023_F_extracellular matrix constituent conferring elasticity; GO:0005515_F_protein binding;	K06468_04640_Hematopoietic cell lineage;	
MGC07551	4	625	EMILIN-2	C1q ; CEREBELLIN-RELATED ; COMPLEMNTC1Q ; TNF-like ; C1Q			
MGC07554	1	704					
MGC07555	1	671	Complement C1q tumor necrosis factor-related protein 3	C1q ; C1Q-RELATED FACTOR ; COLLAGEN ALPHA CHAIN ; TNF-like			
MGC07556	1	701	Centrosomal protein of 120 kDa	C2 domain (Calcium/lipid-binding domain, CaLB)			
MGC07564	1	733	PHD finger protein 10	FYVE/PHD zinc finger ; PHD FINGER PROTEIN 10 ; ZF_PHD_2 ; PHD ; MYST-RELATED PROTEINS			2.3.1.48_Histoneacetyltransferase.;
MGC07566	1	761	T-complex protein 1 subunit delta	GroEL equatorial domain-like ; GroEL-intermediate domain like ; Cpn60_TCP1 ; TCP1_1 ; TCOMPLEXTCP1 ; TCP1_3 ; CHAPERONIN CONTAINING T-COMPLEX PROTEIN 1, DELTA SUBUNIT, TCPD ; TCP1_2 ; CHAPERONIN ; CHAPERONIN60	GO:0005832_C_chaperonin-containing T-complex;		
MGC07567	1	674	Caspase-2	CASPASE-2 ; CARD ; DEATH domain ; CASPASE RELATED	GO:0043065_P_positive regulation of apoptosis;		

MGC07568	1	737	Stromelysin-1	PGBD-like ; PG_binding_1 ; MATRIXIN ; Metalloproteases ('zincins'), catalytic domain ; MATRIX METALLOPROTEINASE ; Peptidase_M10		K01398_04670_Leukocyte transendothelial migration; K01398_04912_GnRH signaling pathway; K01398_05219_Bladder cancer;	3.4.24.22_Stromelysin2.; 3.4.24.17_Stromelysin1.;
MGC07571	1	750		TNF-like			
MGC07572	1	774	Kelch-like protein 28	POZ domain			
MGC07574	1	787	Transaldolase	TRANSALDOLASE_1 ; Transaldolase ; TRANSALDOLASE ; TRANSALDOLASE_2 ; Aldolase ; TRANSALDOLASE 1	GO:0005737_C_cytoplasm; GO:0005975_P_carbohydrate metabolic process; GO:0005515_F_protein binding; GO:0004801_F_transaldolase activity;	K00616_00030_Pentose phosphate pathway;	2.2.1.2_Transaldolase.;
MGC07586	1	348	26S proteasome complex subunit DSS1	Proteasome			
MGC07587	1	378	Collagen alpha-4(VI) chain	VWFA ; vWA-like ; VWA			
MGC07590	1	538					
MGC07594	1	714					
MGC07596	1	712	DNA replication licensing factor MCM3	DNA REPLICATION LICENSING FACTOR ; P-loop containing nucleoside triphosphate hydrolases ; DNA REPLICATION LICENSING FACTOR MCM3 ; MCM	GO:0042555_C_MCM complex;	K02541_03030_DNA replication; K02541_04110_Cell cycle; K02541_04111_Cell cycle - yeast;	
MGC07604	1	723	Bactericidal permeability-increasing protein	LBP_BPI_CETP ; Bactericidal permeability-increasing protein, BPI			
MGC07609	1	772	Complement C1q tumor necrosis factor-related protein 8	C1q ; CEREBELLIN-RELATED ; COMPLEMNTC1Q ; TNF-like ; C1Q		K03987_04610_Complement and coagulation cascades; K03987_05010_Alzheimers disease;	
MGC07620	3	754	Differentially expressed in FDCP 6 homolog				
MGC07623	1	640	T-complex protein 1 subunit gamma	GroEL apical domain-like ; GroEL equatorial domain-like ; Cpn60_TCP1 ; CHAPERONIN CONTAINING T-COMPLEX PROTEIN 1, GAMMA SUBUNIT, TCPG ; TCOMPLEXTCP1 ; coiled-coil ; CHAPERONIN			
MGC07629	1	682	Tripartite motif-containing protein 56	RING/U-box ; TRIM56 PROTEIN ; ZF_RING_1 ; zf-C3HC4 ; B-box zinc-binding domain ; ZF_RING_2 ; ZF_BBOX ; zf-B_box ; RING FINGER-CONTAINING PROTEIN-RELATED			
MGC07630	1	633	Aggrecan core protein	C-type lectin-like ; C-TYPE LECTIN SUPERFAMILY MEMBER ; Lectin_C ; GALACTOSE-SPECIFIC C-TYPE LECTIN ; coiled-coil ; C_TYPE_LECTIN_2			
MGC07633	1	651	Tyrosine-protein kinase-like 7				
MGC07639	1	618	Ubiquitin carboxyl-terminal hydrolase 8	Cysteine proteinases ; UCH_2_3 ; UCH_2_2 ; UBIQUITIN SPECIFIC PROTEASE FAMILY C19-RELATED ; UBIQUITIN SPECIFIC PROTEASE 8 ; UCH	GO:0005622_C_intracellular; GO:0007265_P_Ras protein signal transduction; GO:0005515_F_protein binding; GO:0004843_F_ubiquitin-specific protease activity;		3.1.2.15_Ubiquitinthiolesterase.;
MGC07654	1	670	Epididymal secretory protein E1	E set domains ; E1_DerP2_DerF2 ; NIEMANN PICK TYPE C2 PROTEIN NPC2-RELATED			
MGC07655	1	395					
MGC07656	2	1218		ZINC FINGER PROTEIN 646 ; ZINC_FINGER_C2H2_1 ; C2H2 and C2HC zinc fingers ; ZINC FINGER PROTEINS ; ZINC_FINGER_C2H2_2			
MGC07660	1	696	Acetyl-CoA acetyltransferase, mitochondrial	ACETYL-COA ACETYLTRANSFERASE, MITOCHONDRIAL (ACETOACETYL-COA THIOLASE) ; THIOLASE_3 ; Thiolase-like ; ACETYL-COA C-ACYLTRANSFERASE ; Thiolase_C ; THIOLASE_2	GO:0005743_C_mitochondrial inner membrane; GO:0003985_F_acetyl-CoA C-acetyltransferase activity; GO:0005739_C_mitochondrion;		2.3.1.9_Acetyl-CoAC-acetyltransferase.;
MGC07662	1	787	Protein chibby homolog 1	LEUCINE-RICH PROTEIN ; coiled-coil			

MGC07666	1	732	Nuclear receptor ROR-beta	Nuclear receptor ligand-binding domain			
MGC07667	2	712	Centaurin-gamma-1A	CENTAURIN/ARF ; Miro ; P-loop containing nucleoside triphosphate hydrolases ; RASTRNSFRMNG ; CENTAURIN/ARF-RELATED	GO:0007264_P_small GTPase mediated signal transduction; GO:0005515_F_protein binding; GO:0005634_C_nucleus;		
MGC07669	1	774	Trans-acting transcriptional protein ICP0				
MGC07670	1	615	Serine/threonine-protein kinase PINK1, mitochondrial	Protein kinase-like (PK-like) ; PIK1_HUMAN_Q9BXM7; ; PROTEIN_KINASE_ST ; Pkinase ; SERINE/THREONINE PROTEIN KINASE ; PROTEIN_KINASE_DOM	GO:0033605_P_positive regulation of catecholamine secretion; GO:0033603_P_positive regulation of dopamine secretion; GO:0032226_P_positive regulation of synaptic transmission, dopaminergic; GO:0016301_F_kinase activity; GO:0005737_C_cytoplasm;	K05688_05020_tba;	2.7.11.1_Non-specificserine/threonineproteinkinase.; 2.7.10.2_Non-specificprotein-tyrosinekinase.;
MGC07684	2	989	Baculoviral IAP repeat-containing protein 4	BIR ; INHIBITOR OF APOPTOSIS ; Inhibitor of apoptosis (IAP) repeat ; ZF_RING_2 ; INHIBITOR OF APOPTOSIS PROTEIN 1 AND 2, IAP1, IAP2 ; BIR_REPEAT_2	GO:0043154_P_negative regulation of caspase activity; GO:0005515_F_protein binding; GO:0043066_P_negative regulation of apoptosis; GO:0006916_P_anti-apoptosis;	K04725_04120_Ubiquitin mediated proteolysis; K04725_04210_Apoptosis; K04725_04510_Focal adhesion; K04725_05222_Small cell lung cancer;	
MGC07688	3	555	Interferon alpha-inducible protein 27-like protein 2	INTERFERON-INDUCED 6-16/INTERFERON STIMULATED GENE 12			
MGC07691	1	568	C-type lectin domain family 4 member E	C-type lectin-like ; C_TYPE_LECTIN_1 ; C-TYPE LECTIN SUPERFAMILY MEMBER ; Lectin_C ; EGF_2 ; LOW AFFINITY IMMUNOGLOBULIN EPSILON FC RECEPTOR (CD23 ANTIGEN) ; ANTIFREEZEII ; C_TYPE_LECTIN_2		K06468_04640_Hematopoietic cell lineage;	
MGC07694	1	612	AN1-type zinc finger and ubiquitin domain-containing protein 1	UBIQUITIN_2 ; Ubiquitin-like ; ubiquitin ; AN1, UBIQUITIN-RELATED ; UBIQUITIN		K02977_03010_Ribosome; K08770_03320_PPAR signaling pathway; K02927_03010_Ribosome; K04551_05020_tba;	
MGC07698	1	722	Histone acetyltransferase KAT2B	GCN5 GENERAL CONTROL OF AMINO-ACID SYNTHESIS 5-LIKE 2, GCNL2 ; Bromodomain ; BROMODOMAIN_2 ; FALZ-RELATED BROMODOMAIN-CONTAINING PROTEINS ; BROMODOMAIN		K06062_04330_Notch signaling pathway;	2.3.1.48_Histoneacetyltransferase.;
MGC07700	1	776	Xaa-Pro dipeptidase	XAA-PRO DIPEPTIDASE PEPD/PEPQ(E.COLI) ; Creatinase/aminopeptidase ; PROTEASE FAMILY M24 (METHIONYL AMINOPEPTIDASE, AMINOPEPTIDASE P) ; Peptidase_M24			3.4.13.9_Xaa-Prodipeptidase.;
MGC07701	1	460	Deleted in malignant brain tumors 1 protein	SCAVENGER RECEPTOR-RELATED ; SRCR_1 ; SRCR-like ; SRCR ; LYSYL OXIDASE-RELATED ; SRCR_2 ; SPERACTRCPTR			
MGC07704	1	581		Ankyrin repeat			
MGC07705	1	764	C3 and PZP-like alpha-2-macroglobulin domain-containing protein 8		GO:0005515_F_protein binding;		
MGC07714	3	1597	Uncharacterized protein C12orf41	Snake toxin-like ; Rad51 N-terminal domain-like			
MGC07723	1	523	Neuroguidin	SOMETHING ABOUT SILENCING PROTEIN 10-RELATED			
MGC07726	1	551	12 kDa FK506-binding protein	FKBP-like ; FK506 BINDING PROTEIN ; FK506-BINDING PROTEIN 1 ; FKBP_PPIASE ; FKBP_C			5.2.1.8_Peptidylprolylisomerase.;

MGC07731	1	707	Coiled-coil domain-containing protein 147	tRNA-binding arm ; coiled-coil			
MGC07734	2	875	Fibrinogen-like protein A	FIBRINOGEN AND FIBRONECTIN ; Fibrinogen C-terminal domain-like ; Fibrinogen_C	GO:0007155_P_cell adhesion; GO:0007411_P_axon guidance;	K06252_04510_Focal adhesion; K06252_04512_ECM-receptor interaction;	
MGC07738	1	402	Peritrophin-1	CBM_14 ; CHIT_BIND_II ; CHITIN BINDING PERITROPHIN-A ; Invertebrate chitin-binding proteins	GO:0006508_P_proteolysis; GO:0007616_P_long-term memory; GO:0004295_F_trypsin activity;		
MGC07739	1	696	Calmodulin	EF_HAND_1 ; CALMODULIN ; EF_HAND_2 ; CALFLAGIN ; CALM_CHLRE_P04352; ; EF-hand ; coiled-coil ; CALCIUM BINDING PROTEIN ; efhand			2.7.11.1_Non-specificserine/threonineproteinkinase.;
MGC07746	2	602		C-type lectin-like			
MGC07759	1	574	Stress-induced-phosphoprotein 1	TPR_1 ; TPR ; STRESS-INDUCED-PHOSPHOPROTEIN 1 (STI1) (HSC70/HSP90-ORGANIZING PROTEIN) ; TPR-like ; TPR REPEAT CONTAINING PROTEIN ; TPR_REGION			
MGC07760	3	773	26S proteasome non-ATPase regulatory subunit 14	JAB1/MPN domain ; 26S PROTEASOME REGULATORY ATPASE SUBUNIT ; EUKARYOTIC TRANSLATION INITIATION FACTOR 3 - RELATED ; Mov34	GO:0004175_F_endopeptidase activity; GO:0005838_C_proteasome regulatory particle; GO:0005515_F_protein binding; GO:0006508_P_proteolysis; GO:0009987_P_cellular process;	K03030_03050_Proteasome;	3.1.2.15_Ubiquitinthiolesterase.;
MGC07764	1	781	BTB/POZ domain-containing protein 2	POZ domain ; KELCH-RELATED PROTEIN ; BACK ; BTB	GO:0005515_F_protein binding;		
MGC07770	2	707	Myeloid differentiation primary response protein MyD88	TIR ; Toll/Interleukin receptor TIR domain ; MYD88		K04729_04210_Apoptosis; K04729_04620_Toll-like receptor signaling pathway;	
MGC07779	1	601	Cysteine-rich motor neuron 1 protein	CYR61/CCN1 ; ShK ; VWC ; VWFC_2 ; CONNECTIVE TISSUE GROWTH FACTOR-RELATED ; VWFC_1	GO:0009653_P_anatomical structure morphogenesis; GO:0008283_P_cell proliferation;		
MGC07792	1	749	Myosin regulatory light chain B, smooth adductor muscle	EF_HAND_1 ; EF_HAND_2 ; EF-hand ; MYOSIN REGULATORY LIGHT CHAIN 2 ; Q26069_PLAMG_Q26069;		K10351_04510_Focal adhesion; K10351_04530_Tight junction; K10351_04670_Leukocyte transendothelial migration; K10351_04810_Regulation of actin cytoskeleton;	
MGC07794	1	442	von Willebrand factor C domain-containing protein 2-like	VWC ; VWFC_2 ; EXTRACELLULAR MATRIX GLYCOPROTEIN RELATED ; VWFC_1		K06236_04510_Focal adhesion; K06236_04512_ECM-receptor interaction;	
MGC07799	2	542	Thyroglobulin	INSULIN-LIKE GROWTH FACTOR BINDING PROTEIN ; Thyroglobulin_1 ; Thyroglobulin type-1 domain ; INSULIN-LIKE GROWTH FACTOR BINDING PROTEIN 3 ; THYROGLOBULIN_1_2		K10809_05320_Autoimmune thyroid disease;	
MGC07816	1	674	Acyl-CoA-binding domain-containing protein 6	ANKYRIN ; Ank ; ACYL-COENZYME A BINDING DOMAIN CONTAINING 6 ; ANKYRIN REPEAT-CONTAINING ; ANK_REPEAT ; ANK_REP_REGION ; Ankyrin repeat			2.7.11.1_Non-specificserine/threonineproteinkinase.; 2.4.2.30_NAD(+)ADP-ribosyltransferase.;
MGC07819	1	720	RING finger protein 13	RING FINGER AND PROTEASE ASSOCIATED DOMAIN-CONTAINING ; Transferrin receptor ectodomain, apical domain ; SPERIZIN, RING ZINC FINGER PROTEIN 4 ; PA			
MGC07821	1	500	Protein kinase C delta type	C2DOMAIN ; C2 domain (Calcium/lipid-binding domain, CaLB)		K06068_04530_Tight junction;	2.7.11.13_ProteinkinaseC.;

MGC07822	1	779	Ficolin-1	FIBRINOGEN AND FIBRONECTIN ; Fibrinogen C-terminal domain-like ; Fibrinogen_C ; FIBRIN_AG_C_DOMAIN			
MGC07825	1	747	Trichohyalin	coiled-coil			
MGC07828	1	719	CAP-Gly domain-containing linker protein 3	ANKYRIN ; Ank ; RESTIN-RELATED ; ANK_REP_REGION ; Ankyrin repeat ; DYNACTIN 1-RELATED MICROTUBULE-BINDING			
MGC07833	1	723	Cathepsin L	Cysteine proteinases ; THIOL_PROTEASE_CYS ; Inhibitor_I29 ; Peptidase_C1 ; CYSTEINE PROTEASE FAMILY C1-RELATED ; CATL_HUMAN_P07711; ; CATHEPSIN L		K01365_04612_Antigen processing and presentation;	3.4.22.15_CathepsinL.;
MGC07835	1	631	Uncharacterized transmembrane protein DDB_G0289901				
MGC07839	1	691	Heat shock cognate 70 kDa protein	Heat shock protein 70kD (HSP70), peptide-binding domain ; HEAT SHOCK PROTEIN 70KDA ; Heat shock protein 70kD (HSP70), C-terminal subdomain ; HEAT SHOCK PROTEIN 70 (HSP70) ; HSP70		K03283_04010_MAPK signaling pathway; K03283_04612_Antigen processing and presentation;	
MGC07840	1	732	Buccalin				
MGC07845	1	659		IG_MHC			
MGC07852	3	1245	Complement C1q-like protein 4	C1q ; GLIACOLIN-RELATED ; CEREBELLIN-RELATED ; COMPLEMNTC1Q ; TNF-like ; C1Q		K03987_04610_Complement and coagulation cascades; K03987_05010_Alzheimers disease;	
MGC07856	2	777	Methylated-DNA--protein-cysteine methyltransferase	Methylated DNA-protein cysteine methyltransferase, C-terminal domain ; MGMT ; DNA_binding_1 ; ogt: methylated-DNA-[protein]-cysteine S-met ; METHYLATED-DNA--PROTEIN-CYSTEINE METHYLTRANSFERASE ; Methylated DNA-protein cysteine methyltransferase domain	GO:0006307_P_DNA dealkylation; GO:0008168_F_methyltransferase activity; GO:0043281_P_regulation of caspase activity; GO:0006974_P_response to DNA damage stimulus; GO:0006281_P_DNA repair;		2.1.1.63_Methylated-DNA--[protein]-cysteineS-methyltransferase.;
MGC07865	1	597	Baculoviral IAP repeat-containing protein 7	INHIBITOR OF APOPTOSIS ; Inhibitor of apoptosis (IAP) repeat ; INHIBITOR OF APOPTOSIS PROTEIN 1 AND 2, IAP1, IAP2 ; BIR_REPEAT_2		K04725_04120_Ubiquitin mediated proteolysis; K04725_04210_Apoptosis; K04725_04510_Focal adhesion; K04725_05222_Small cell lung cancer;	
MGC07873	1	749	Ficolin-1	FIBRINOGEN AND FIBRONECTIN ; Fibrinogen C-terminal domain-like ; Fibrinogen_C			
MGC07877	1	511					
MGC07880	1	582	E3 ubiquitin-protein ligase UBR5	RCC1/BLIP-II		K10593_04120_Ubiquitin mediated proteolysis;	
MGC07881	2	692					
MGC07888	1	730	Fibrinogen C domain-containing protein 1	FIBRINOGEN AND FIBRONECTIN ; Fibrinogen C-terminal domain-like ; Fibrinogen_C ; FIBRIN_AG_C_DOMAIN		K06252_04510_Focal adhesion; K06252_04512_ECM-receptor interaction;	
MGC07901	1	735	Protein TSSC1	TUMOR SUPPRESSING SUBTRANSFERABLE CANDIDATE 1 ; WD-REPEAT PROTEIN ; WD40 repeat-like ; WD_REPEATS_1 ; WD_REPEATS_REGION	GO:0005515_F_protein binding;		
MGC07911	1	673	RISC-loading complex subunit tarbp2				
MGC07918	1	712					
MGC07926	1	715	N,N'-diacetylchitobiase	E set domains ; BETA-HEXOSAMINIDASE ; (Trans)glycosidases ; Glyco_hydro_20 ; CHB_HEX_C			3.2.1.52_Beta-N-acetylhexosaminidase.;
MGC07927	1	595	E3 ubiquitin-protein ligase NRDP1	RING/U-box ; 2FE2S_FER_1 ; TRAF domain-like ; ZF_RING_1 ; zf-C3HC4 ; USP8_interact ; SUBZF_RING_2 ; coiled-coil	GO:0005515_F_protein binding;	K09848_05222_Small cell lung cancer;	

MGC07947	2	660	Complement C1q-like protein 2	C1q ; C1Q-RELATED FACTOR ; COLLAGEN ALPHA CHAIN ; COMPLEMNTC1Q ; TNF-like ; coiled-coil ; C1Q	GO:0005515_F_protein binding;		
MGC07963	1	557	Complement C1q-like protein 4	C1q ; GLIACOLIN-RELATED ; CEREBELLIN-RELATED ; COMPLEMNTC1Q ; TNF-like ; C1Q			
MGC07964	3	570	Complement C1q-like protein 4	C1q ; CEREBELLIN-RELATED ; COMPLEMNTC1Q ; TNF-like ; C1Q		K03986_04610_Complement and coagulation cascades; K03986_05010_Alzheimers disease; K03987_04610_Complement and coagulation cascades; K03987_05010_Alzheimers disease; K03988_04610_Complement and coagulation cascades; K03988_05010_Alzheimers disease;	
MGC07973	1	808	Centrosomal protein of 290 kDa	SUBcoiled-coil			
MGC07976	1	569	U3 small nucleolar RNA-associated protein 15 homolog	WD_REPEATS_2 ; GPROTEINBRPT ; SUBO82266_ARATH_O82266; ; WD40 repeat-like ; WD_REPEATS_1 ; WD_REPEATS_REGION ; WD40	GO:0005737_C_cytoplasm;	K03130_03022_Basal transcription factors; K06666_04111_Cell cycle - yeast; K01852_00100_Biosynthesis of steroids;	2.7.11.1_Non-specificserine/threonineproteinkinase.; 2.3.1.48_Histoneacetyltransferase.; 2.7.11.7_[Myosinheavy-chain]kinase.;
MGC07982	1	740	Stromal cell-derived factor 2-like protein 1	MIR domain (Pfam 02815) ; ER_TARGET ; STROMAL CELL-DERIVED FACTOR 2 ; DOLICHYL-PHOSPHATE-MANNOSE--PROTEIN MANNOSYLTRANSFERASE ; MIR	GO:0005515_F_protein binding;		2.4.1.109_Dolichyl-phosphate-mannose-proteinmannosyltransferase.;
MGC07990	1	660	Sushi, nidogen and EGF-like domain-containing protein 1	EGF/Laminin ; EGF-LIKE DOMAIN PROTEIN ; EGF ; EGF_3 ; EGF_1 ; EGF_2 ; NEUROGENIC LOCUS DELTA PROTEIN		K06051_04330_Notch signaling pathway; K02599_04320_Dorso-ventral axis formation; K02599_04330_Notch signaling pathway; K06052_04330_Notch signaling pathway;	
MGC07991	1	409	Translin-associated protein X	Translin ; TRANSLIN AND TRANSLIN ASSOCIATED PROTEIN X ; TRANSLIN ASSOCIATED FACTOR X			
MGC07996	1	645	Fucolectin-6	Galactose-binding domain-like ; coiled-coil ; F5_F8_type_C			
MGC07998	1	792	Adhesive plaque matrix protein 2	EGF_2			
MGC08006	2	779	Zinc finger protein 227	SUBZINC_FINGER_C2H2_1 ; C2H2 and C2HC zinc fingers ; zf-C2H2 ; ZINC FINGER PROTEINS ; ZINC_FINGER_C2H2_2			
MGC08021	1	458	RNA polymerase-associated protein CTR9 homolog				
MGC08027	4	540	C-type lectin domain family 4 member G				
MGC08028	1	732	ERC protein 2	Eukaryotic DNA topoisomerase I, dispensable insert domain ; coiled-coil ; RAB6-INTERACTING PROTEIN 2/ELKS/ERC/CAST	GO:0030426_C_growth cone; GO:0005515_F_protein binding; GO:0042734_C_presynaptic membrane;		
MGC08029	2	790	N,N'-diacetylchitobiase	E set domains ; BETA-HEXOSAMINIDASE ; (Trans)glycosidases ; Glyco_hydro_20 ; CHB_HEX_C			3.2.1.52_Beta-N-acetylhexosaminidase.;
MGC08044	1	668	Equistatin	HLA CLASS II GAMMA CHAIN ; Thyroglobulin_1 ; MHC CLASS II-ASSOCIATED INVARIANT CHAIN-RELATED ; Thyroglobulin type-1 domain ; THYROGLOBULIN_1_1 ; THYROGLOBULIN_1_2		K10809_05320_Autoimmune thyroid disease;	
MGC08046	1	548	von Willebrand factor A domain-containing protein 5A	VON WILLEBRAND FACTOR, TYPE A DOMAIN CONTAINING ; VWFA ; BREAST CANCER SUPPRESSOR CANDIDATE 1,BCSC-1 ; vWA-like			

MGC08051	1	195	Zinc finger protein basonuclin-2	DISCONNECTED-RELATED	GO:0003677_F_DNA binding; GO:0005634_C_nucleus;		
MGC08052	1	550	C-type lectin domain family 4 member E	C-type lectin-like ; C_TYPE_LECTIN_1 ; C-TYPE LECTIN SUPERFAMILY MEMBER ; Lectin_C ; GALACTOSE-SPECIFIC C-TYPE LECTIN ; ANTIFREEZEII ; C_TYPE_LECTIN_2		K06468_04640_Hematopoietic cell lineage;	
MGC08061	1	485	Peroxisome proliferator-activated receptor gamma	NUCLEAR_REC_DBD_1 ; NUCLEAR_REC_DBD_2 ; STROIDFINGER ; NHR6_CAEEL_P41829; ; Glucocorticoid receptor-like (DNA-binding domain) ; NUCLEAR HORMONE RECEPTOR ; zf-C4 ; VITAMINDR ; PEROXISOME PROLIFERATOR ACTIVATED RECEPTOR ALPHA (PPAR-ALPHA)		K08530_03320_PPAR signaling pathway; K08530_05216_Thyroid cancer;	
MGC08063	1	609	Exosome complex exonuclease RRP45	Ribosomal protein S5 domain 2-like ; EXOSOME COMPLEX EXONUCLEASE RRP45 (POLYMYOSITIS/SCLERODERMA AUTOANTIGEN 1) (RIBOSOMAL RNA PROCESSING PROTEIN 45) ; RNase_PH ; EXOSOME COMPLEX EXONUCLEASE (RIBOSOMAL RNA PROCESSING PROTEIN)			2.7.7.56_tRNAnucleotidyltransferase.;
MGC08064	1	658	Fibrinogen C domain-containing protein 1	FIBRINOGEN AND FIBRONECTIN ; Fibrinogen C-terminal domain-like ; Fibrinogen_C		K06252_04510_Focal adhesion; K06252_04512_ECM-receptor interaction;	
MGC08068	1	687	Paramyosin (Fragment)	coiled-coil			
MGC08069	1	425	Acyl-CoA synthetase family member 2, mitochondrial	AMP-binding ; Acetyl-CoA synthetase-like ; ATP-DEPENDENT AMP-BINDING ENZYME FAMILY MEMBER ; LONG-CHAIN-FATTY-ACID--COA LIGASE		K01897_00071_Fatty acid metabolism; K01897_03320_PPAR signaling pathway; K01897_04920_Adipocytokine signaling pathway;	6.2.1.3_Long-chain-fatty-acid--CoAligase.;
MGC08071	1	690	Clumping factor B				
MGC08075	1	709	Putative GTP-binding protein tag-210	DUF933 ; P-loop containing nucleoside triphosphate hydrolases ; GTP-BINDING PROTEIN-RELATED ; TGS-like	GO:0005515_F_protein binding; GO:0009792_P_embryonic development ending in birth or egg hatching;		
MGC08077	1	711	Ankyrin repeat and KH domain-containing protein mask	ANKYRIN ; Ank ; ANKYRIN REPEAT-CONTAINING ; ANK_REPEAT ; ANK_REP_REGION ; Ankyrin repeat ; ASP_PROTEASE			2.7.11.1_Non-specificserine/threonineproteinkinase.; 2.4.2.30_NAD(+)ADP-ribosyltransferase.; 2.1.1.43_Histone-lysineN-methyltransferase.;
MGC08078	1	616	Complement C1q tumor necrosis factor-related protein 6	C1q ; GLIACOLIN-RELATED ; CEREBELLIN-RELATED ; COMPLEMNTC1Q ; TNF-like ; C1Q			
MGC08079	1	564	Serine/threonine-protein phosphatase 6 regulatory ankyrin repeat subunit C	ANK REPEAT-CONTAINING ; ANKYRIN ; Ank ; ANKYRIN REPEAT-CONTAINING ; ANK_REPEAT ; ANK_REP_REGION ; Ankyrin repeat	GO:0030424_C_axon; GO:0005515_F_protein binding; GO:0007411_P_axon guidance; GO:0050808_P_synapse organization and biogenesis; GO:0045202_C_synapse;	K03426_00760_Nicotinate and nicotinamide metabolism; K08803_05219_Bladder cancer;	2.7.11.1_Non-specificserine/threonineproteinkinase.; 3.6.1.22_NAD(+)diphosphatase.;
MGC08080	1	575	Caspase-3	Caspase-like ; IL1BCENZYME ; CASPASE_P10 ; CASPASE_P20 ; CASPASE_CYS ; Peptidase_C14 ; CASPASE-1 ; CASPASE RELATED			3.4.22.56_Caspase-3.;
MGC08083	1	277	Nucleolar RNA helicase 2	DEAD-BOX PROTEIN 21, 50 ; DEAD BOX ATP-DEPENDENT RNA HELICASE		K01529_00500_Starch and sucrose metabolism; K01529_00790_Folate biosynthesis;	
MGC08088	2	786					

MGC08097	1	715	Signal peptidase complex catalytic subunit SEC11A	PROTEASE FAMILY S26 MICROSOMAL SIGNAL PEPTIDASE SUBUNIT SPC18,21/SEC11(YEAST) ; LexA/Signal peptidase ; SIGNALPTASE ; SPASE_I_1 ; sigpep_I_arch: signal peptidase I ; Peptidase_S24 ; SPASE_I_3		K03100_03060_Protein export;	3.4.21.89_SignalpeptidaseI.;
MGC08098	1	716	Kielin/chordin-like protein	Pacifastin_I ; VWFC_2 ; PMP inhibitors	GO:0030513_P_positive regulation of BMP signaling pathway; GO:0005615_C_extracellular space; GO:0005515_F_protein binding;		
MGC08100	2	1110	Myosin heavy chain, non-muscle	MYOSIN HEAVY CHAIN, NONMUSCLE OR SMOOTH MUSCLE ; MYOSIN ; coiled-coil ; Myosin_tail_1	GO:0008307_F_structural constituent of muscle; GO:0030241_P_muscle thick filament assembly; GO:0006939_P_smooth muscle contraction; GO:0048739_P_cardiac muscle fiber development; GO:0048251_P_elastic fiber assembly; GO:0005515_F_protein binding; GO:0006461_P_protein complex assembly;	K10352_04530_Tight junction;	
MGC08107	1	640	Collagen alpha-4(VI) chain	MATRILIN 1 ; VWFA ; VON WILENBRAND FACTOR RELATED ; vWA-like ; VWA	GO:0005201_F_extracellular matrix structural constituent; GO:0005578_C_proteinaceous extracellular matrix;		
MGC08109	1	640	DNA mismatch repair protein Msh2	MutS_II ; MutS_I		K08735_03430_Mismatch repair; K08735_05210_Colorectal cancer;	
MGC08116	1	648	Complement C1q-like protein 3	C1q ; GLIACOLIN-RELATED ; CEREBELLIN-RELATED ; COMPLEMNTC1Q ; TNF-like ; C1Q	GO:0005515_F_protein binding;	K03987_04610_Complement and coagulation cascades; K03987_05010_Alzheimers disease;	
MGC08117	1	670	Lysozyme g	SLT ; LYSOZYMEG ; Lysozyme g ; Lysozyme-like			3.2.1.17_Lysozyme.;
MGC08119	1	623	Gamete and mating-type specific protein A	VWFA ; MATRILIN 2 ; VON WILENBRAND FACTOR RELATED ; vWA-like ; VWA			
MGC08124	1	559	Complement C1q tumor necrosis factor-related protein 3	C1q ; GLIACOLIN-RELATED ; CEREBELLIN-RELATED ; COMPLEMNTC1Q ; TNF-like ; C1Q	GO:0005576_C_extracellular region;		
MGC08125	1	740	Ankyrin repeat domain-containing protein 50	ANK REPEAT-CONTAINING ; ANKYRIN ; Ank ; ANKYRIN REPEAT-CONTAINING ; ANK_REPEAT ; ANK_REP_REGION ; Ankyrin repeat			2.7.11.1_Non-specificserine/threonineproteinkinase.; 2.1.1.43_Histone-lysineN-methyltransferase.;
MGC08128	1	550	DnaJ homolog subfamily C member 8	DNAJ HOMOLOG SUBFAMILY C MEMBER 8 ; DNAJ_2 ; DnaJ ; Chaperone J-domain ; DNAJ HOMOLOG SUBFAMILY C MEMBER 8/LIPOPOLYSACCHARIDE SPECIFIC RESPONSE-7-RELATED			
MGC08129	1	713	Low-density lipoprotein receptor-related protein 12	LDL receptor-like module ; LOW DENSITY LIPOPROTEIN RECEPTOR ; Ldl_recept_a ; CUB ; Spermadhesin, CUB domain ; LDLRA_2 ; LOW-DENSITY LIPOPROTEIN RECEPTOR (LDL)			
MGC08132	1	697	Transforming growth factor-beta-induced protein ig-h3	FAS1 ; PERIOSTIN-RELATED ; Fasciclin ; FAS1 domain ; TRANSFORMING GROWTH FACTOR-BETA INDUCED PROTEIN IG-H3	GO:0016203_P_muscle attachment; GO:0007155_P_cell adhesion; GO:0005615_C_extracellular space; GO:0005515_F_protein binding; GO:0005578_C_proteinaceous extracellular matrix;		
MGC08138	2	626	Coiled-coil domain-containing protein 147	coiled-coil			

MGC08144	1	724	Sarcoplasmic calcium-binding protein	EF_HAND_2 ; EF-hand ; efhand			
MGC08147	1	680	DEAD-box ATP-dependent RNA helicase 42	coiled-coil			
MGC08152	1	416		Kazal_1 ; Kazal-type serine protease inhibitors ; FOLLISTATIN			
MGC08154	1	584	Neuropilin and tolloid-like protein 1	LDL receptor-like module ; LOW DENSITY LIPOPROTEIN RECEPTOR ; Ldl_recept_a ; Spermadhesin, CUB domain ; LDLRA_2 ; LOW-DENSITY LIPOPROTEIN RECEPTOR (LDL)			
MGC08157	2	749		Concanavalin A-like lectins/glucanases			
MGC08158	2	726	Zinc metalloproteinase nas-4	ZINC METALLOPROTEINASE-RELATED ; DISCOIDIN, CUB, EGF, LAMININ , AND ZINC METALLOPROTEASE DOMAIN ; ZINC_PROTEASE ; Metalloproteases ('zincins'), catalytic domain ; Astacin ; ASTACIN			3.4.24.21_Astacin.; 3.4.24.18_MeprinA.;
MGC08162	1	528	Docking protein 3	IRS_PTB ; IRS ; DOCKING PROTEIN RELATED ; DOCKING PROTEIN 3 ; PH domain-like			
MGC08167	1	726	EMILIN-2	C1q ; GLIACOLIN-RELATED ; CEREBELLIN-RELATED ; TNF-like ; C1Q	GO:0005576_C_extracellular region; GO:0030023_F_extracellular matrix constituent conferring elasticity; GO:0005515_F_protein binding;		
MGC08174	1	664	Probable glutathione S-transferase 7	GST_C ; GST_N ; Glutathione S-transferase (GST), C-terminal domain ; Thioredoxin-like ; GLUTATHIONE S-TRANSFERASE	GO:0005515_F_protein binding;	K00799_00480_Glutathione metabolism; K00799_00980_Metabolism of xenobiotics by cytochrome P450; K01830_00590_Arachidonic acid metabolism;	2.5.1.18_Glutathionetransferase.; 5.3.99.2_Prostaglandin-Dsynthase.;
MGC08179	1	662	Protein hu-li tai shao	ADDUCIN ; ADDUCIN RELATED PROTEIN			
MGC08180	1	503	Perlucin	C-type lectin-like ; C_TYPE_LECTIN_1 ; C-TYPE LECTIN SUPERFAMILY MEMBER ; Lectin_C ; GALACTOSE-SPECIFIC C-TYPE LECTIN ; ANTIFREEZEII ; C_TYPE_LECTIN_2		K06468_04640_Hematopoietic cell lineage;	
MGC08186	1	665	Tumor necrosis factor receptor superfamily member 27				
MGC08195	1	728	Centromere protein F	tRNA-binding arm ; coiled-coil			
MGC08197	2	701	Microfibril-associated glycoprotein 4	FIBRINOGEN AND FIBRONECTIN ; Fibrinogen C-terminal domain-like ; Fibrinogen_C	GO:0030297_F_transmembrane receptor protein tyrosine kinase activator activity; GO:0001527_C_microfibril; GO:0007155_P_cell adhesion;		
MGC08200	1	715	Receptor-type tyrosine-protein phosphatase U	PROTEIN-TYROSINE PHOSPHATASE ; TYR_PHOSPHATASE_2 ; TYR_PHOSPHATASE_PTP ; TYR_PHOSPHATASE_1 ; PRTYPHPHTASE ; (Phosphotyrosine protein) phosphatases II ; Y_phosphatase		K05693_04514_Cell adhesion molecules (CAMs); K05693_04520_Adherens junction;	3.1.3.48_Protein-tyrosine-phosphatase.;
MGC08201	1	584	Interferon alpha-inducible protein 27-like protein 2	Immunoglobulin			
MGC08207	1	735	Protein jagged-1	EGF/Laminin ; Fz ; FZ ; EGF ; EGF_3 ; CADHERIN ; Frizzled cysteine-rich domain ; EGF_1 ; CADHERIN-RELATED TUMOR SUPPRESSOR-RELATED	GO:0016021_C_integral to membrane; GO:0001889_P_liver development; GO:0031016_P_pancreas development;	K06051_04330_Notch signaling pathway; K06052_04330_Notch signaling pathway;	
MGC08209	1	680	Ankyrin repeat domain-containing protein 50	ANK REPEAT-CONTAINING ; ANKYRIN ; Ank ; ANKYRIN REPEAT-CONTAINING ; ANK_REPEAT ; ANK_REP_REGION ; Ankyrin repeat	GO:0048675_P_axon extension;		2.7.11.1_Non-specificserine/threonineproteinkinase.;

MGC08210	2	771	Macrophage mannose receptor 1	C-type lectin-like ; C_TYPE_LECTIN_1 ; C-TYPE LECTIN SUPERFAMILY MEMBER ; Lectin_C ; GALACTOSE-SPECIFIC C-TYPE LECTIN ; C_TYPE_LECTIN_2			
MGC08213	1	772	YTH domain family protein 2	YTH DOMAIN PROTEIN 1 ; YTH (YT521-B HOMOLOGY) DOMAIN-CONTAINING ; YTH	GO:0005737_C_cytoplasm; GO:0005515_F_protein binding; GO:0006959_P_humoral immune response; GO:0005634_C_nucleus;		
MGC08214	1	599	Ubiquitin	UBIQUITIN (RIBOSOMAL PROTEIN L40) ; UBIQUITIN_2 ; Ubiquitin-like ; ubiquitin ; UBIQUITIN_1 ; UBIQUITIN		K02977_03010_Ribosome; K08770_03320_PPAR signaling pathway; K02927_03010_Ribosome; K04551_05020_tba;	
MGC08225	1	786	Ankyrin repeat domain-containing protein 6	ANKYRIN ; Ank ; ANKYRIN REPEAT-CONTAINING ; ANK_REPEAT ; ANK_REP_REGION ; SERINE/THREONINE-PROTEIN KINASE RIPK4 ; Ankyrin repeat		K08803_05219_Bladder cancer;	2.7.11.1_Non-specificserine/threonineproteinkinase.; 2.1.1.43_Histone-lysineN-methyltransferase.;
MGC08228	1	799	Receptor-type tyrosine-protein phosphatase epsilon	PROTEIN-TYROSINE PHOSPHATASE ; TYR_PHOSPHATASE_2 ; TYR_PHOSPHATASE_PTP ; PRTYPHPHTASE ; (Phosphotyrosine protein) phosphatases II ; Y_phosphatase	GO:0004725_F_protein tyrosine phosphatase activity; GO:0042803_F_protein homodimerization activity; GO:0007185_P_transmembrane receptor protein tyrosine phosphatase signaling pathway;	K05695_04514_Cell adhesion molecules (CAMs); K05695_04520_Adherens junction; K05695_04910_Insulin signaling pathway;	3.1.3.48_Protein-tyrosine-phosphatase.;
MGC08241	1	556	CD82 antigen	Tetraspannin			
MGC08246	1	540	Protein regulator of cytokinesis 1	PROTEIN REGULATOR OF CYTOKINESIS 1 PRC1-RELATED ; coiled-coil	GO:0005515_F_protein binding; GO:0000910_P_cytokinesis; GO:0005876_C_spindle microtubule; GO:0005634_C_nucleus; GO:0000022_P_mitotic spindle elongation;		
MGC08250	1	717	Endoplasmic reticulum-Golgi intermediate compartment protein 2	SEROLOGICALLY DEFINED BREAST CANCER ANTIGEN NY-BR-84-RELATED	GO:0005515_F_protein binding; GO:0005793_C_ER-Golgi intermediate compartment; GO:0006888_P_ER to Golgi vesicle-mediated transport;		
MGC08261	1	768	Neurobeachin				
MGC08268	2	1071					
MGC08276	1	494	Chromatin-remodeling complex ATPase chain Iswi	HAND domain of the nucleosome remodeling ATPase ISWI ; HAND ; HELICASE SMARCA1,5 ; Homeodomain-like ; ATP-DEPENDENT HELICASE SMARCA (SWI/SNF-RELATED MATRIX-ASSOCIATED ACTIN-DEPENDENT REGULATOR OF CHROMATIN A)-RELATED			
MGC08281	1	751	Neurogenic locus notch homolog protein 2	EGF/Laminin ; EGF-LIKE DOMAIN PROTEIN ; EGF ; EGF_3 ; ASX_HYDROXYL ; CRUMBS(D.MELANOGASTER) RELATED ; EGF_1 ; EGF_2 ; EGFBLOOD		K02599_04320_Dorso-ventral axis formation; K02599_04330_Notch signaling pathway;	2.7.10.1_Receptorprotein-tyrosinekinase.;
MGC08283	1	762	DnaJ homolog subfamily B member 1	DNAJ/HSP40 ; DNAJPROTEIN ; DNAJ_2 ; DNAJ HOMOLOG SUBFAMILY B MEMBER 4, 5 ; DnaJ ; Chaperone J-domain			

MGC08287	1	730	Sin3 histone deacetylase corepressor complex component SDS3	Sds3 ; BREAST CANCER METASTASIS-SUPPRESSOR 1 ; SUPPRESSOR OF DEFECTIVE SILENCING 3 ; coiled-coil	GO:0042802_F_identical protein binding; GO:0042826_F_histone deacetylase binding; GO:0016580_C_Sin3 complex; GO:0005515_F_protein binding; GO:0016481_P_negative regulation of transcription;		
MGC08291	1	764	NAD-dependent ADP-ribosyltransferase sirtuin-4	CHROMATIN REGULATORY PROTEIN SIR2 ; DHS-like NAD/FAD-binding domain ; SIRTUIN ; SIR2	GO:0005739_C_mitochondrion; GO:0005515_F_protein binding; GO:0046676_P_negative regulation of insulin secretion;	K01463_00760_Nicotinate and nicotinamide metabolism; K01463_00770_Pantothenate and CoA biosynthesis; K01463_00930_Caprolactam degradation;	2.4.2.31_NAD(P)(+)--protein-arginineADP-ribosyltransferase.;
MGC08295	1	713	Titin	MYOSIN-BINDING PROTEIN-RELATED ; Fibronectin type III ; FN3 ; TITIN ; fn3 ; FNTYPEIII		K06756_04514_Cell adhesion molecules (CAMs); K06766_04514_Cell adhesion molecules (CAMs);	2.7.11.1_Non-specificserine/threonineprotein kinase.; 3.1.3.48_Protein-tyrosine-phosphatase.;
MGC08298	1	707		HMG_box ; HMG-box ; coiled-coil ; HMG_BOX_2			
MGC08299	1	536	Fibrinogen-like protein A	FIBRINOGEN AND FIBRONECTIN ; Fibrinogen C-terminal domain-like ; Fibrinogen_C ; FICOLIN		K06252_04510_Focal adhesion; K06252_04512_ECM-receptor interaction;	
MGC08309	1	644	Titin	Immunoglobulin ; MYOSIN-BINDING PROTEIN-RELATED ; IG_LIKE ; I-set ; Fibronectin type III ; FN3 ; TITIN ; fn3 ; FNTYPEIII	GO:0040011_P_locomotion;	K00907_04020_Calcium signaling pathway; K00907_04510_Focal adhesion; K00907_04810_Regulation of actin cytoskeleton; K06491_04514_Cell adhesion molecules (CAMs);	2.7.11.1_Non-specificserine/threonineprotein kinase.; 2.7.11.18_[Myosinlight-chain]kinase.;
MGC08316	1	662	Matrix metalloproteinase-24	Hemopexin ; Hemopexin-like domain ; MATRIX METALLOPROTEASE 15, MMP-15 ; HEMOPEXIN ; MATRIX METALLOPROTEINASE			3.4.24.24_GelatinaseA.;
MGC08320	1	565	Calmodulin	EF_HAND_1 ; CALMODULIN ; EF_HAND_2 ; O96792_BRALA_O96792; ; EF-hand ; CALCIUM BINDING PROTEIN ; efhand			2.7.11.1_Non-specificserine/threonineprotein kinase.;
MGC08329	1	708	Thyroglobulin	INSULIN-LIKE GROWTH FACTOR BINDING PROTEIN ; Thyroglobulin_1 ; Thyroglobulin type-1 domain ; INSULIN-LIKE GROWTH FACTOR BINDING PROTEIN 3 ; THYROGLOBULIN_1_2		K10809_05320_Autoimmune thyroid disease;	
MGC08330	1	694	Titin	MYOSIN-BINDING PROTEIN-RELATED ; Fibronectin type III ; FN3 ; TITIN ; fn3 ; FNTYPEIII		K05106_04360_Axon guidance; K00907_04020_Calcium signaling pathway; K00907_04510_Focal adhesion; K00907_04810_Regulation of actin cytoskeleton; K06765_04360_Axon guidance; K06765_05210_Colorectal cancer;	2.7.11.1_Non-specificserine/threonineprotein kinase.; 2.7.10.1_Receptorprotein-tyrosinekinase.; 2.7.11.18_[Myosinlight-chain]kinase.;
MGC08333	1	547	Perlucin	C-type lectin-like ; PNCREATITSAP ; ASIALOGLYCOPROTEIN RECEPTOR ; C_TYPE_LECTIN_1 ; C-TYPE LECTIN SUPERFAMILY MEMBER ; Lectin_C ; C_TYPE_LECTIN_2	GO:0008329_F_pattern recognition receptor activity; GO:0006910_P_phagocytosis, recognition; GO:0030169_F_low-density lipoprotein binding; GO:0006955_P_immune response;		
MGC08340	1	746	p53 and DNA damage-regulated protein 1	P53 AND DNA DAMAGE-REGULATED PROTEIN ; Prefoldin ; coiled-coil			
MGC08343	1	784	Neurofilament heavy polypeptide				
MGC08344	1	773	Apoptosis 1 inhibitor	Q7S3G2_NEUCR_Q7S3G2; Inhibitor of apoptosis (IAP) repeat ; coiled-coil			

MGC08346	1	641	Complement C1q-like protein 4	COMPLEMENT C1Q TUMOR NECROSIS FACTOR-RELATED PROTEIN 4 ; C1q ; COLLAGEN ALPHA CHAIN ; COMPLEMNTC1Q ; TNF-like ; C1Q		K03986_04610_Complement and coagulation cascades; K03986_05010_Alzheimers disease; K03987_04610_Complement and coagulation cascades; K03987_05010_Alzheimers disease;	
MGC08353	2	665	Thyroid receptor-interacting protein 11	coiled-coil	GO:0005515_F_protein binding; GO:0006366_P_transcription from RNA polymerase II promoter; GO:0003713_F_transcription coactivator activity;		
MGC08354	1	690	Tubulin-folding cofactor B	Cap-Gly domain ; Ubiquitin-like ; CAP_GLY ; TUBULIN-SPECIFIC CHAPERONE B (TUBULIN FOLDING COFACTOR B) ; CAP_GLY_2 ; coiled-coil ; DYNACTIN 1-RELATED MICROTUBULE-BINDING	GO:0005515_F_protein binding; GO:0015630_C_microtubule cytoskeleton;		
MGC08355	1	524	Tissue factor pathway inhibitor 2	BPTI-like			
MGC08359	1	595	Perlucin	C-type lectin-like ; ASIALOGLYCOPROTEIN RECEPTOR ; C-TYPE LECTIN SUPERFAMILY MEMBER ; Lectin_C ; EGF_2 ; ANTIFREEZEII ; C_TYPE_LECTIN_2			
MGC08366	1	555	Caprin-2	C1q ; COLLAGEN ALPHA 1(VIII) CHAIN ; COLLAGEN ALPHA CHAIN ; COMPLEMNTC1Q ; TNF-like ; C1Q		K03986_04610_Complement and coagulation cascades; K03986_05010_Alzheimers disease;	
MGC08368	1	652	Aminopeptidase B	PROTEASE M1 ZINC METALLOPROTEASE ; AMINOPEPTIDASE B ; Leukotriene A4 hydrolase N-terminal domain ; Peptidase_M1	GO:0005576_C_extracellular region; GO:0005886_C_plasma membrane; GO:0030601_F_aminopeptidase B activity;	K01254_00590_Arachidonic acid metabolism;	3.4.11.6_AminopeptidaseB.; 3.3.2.6_Leukotriene-A(4)hydrolase.;
MGC08380	1	676	Heavy metal-binding protein HIP	C1q ; TNF-like			
MGC08386	1	565					
MGC08392	1	546	AP-1 complex subunit sigma-2	CLATHRIN ASSEMBLY PROTEIN AP19 ; SNARE-like ; Clat_adaptor_s ; CLATHRIN COAT ASSEMBLY PROTEIN ; CLAT_ADAPTOR_S			
MGC08393	4	750					
MGC08402	1	609	Complement C1q-like protein 3	coiled-coil			
MGC08404	1	366	Poly [ADP-ribose] polymerase 4	VON WILLEBRAND FACTOR, TYPE A DOMAIN CONTAINING ; VWFA ; vWA-like ; VWA		K10798_03410_Base excision repair;	2.4.2.30_NAD(+)ADP-ribosyltransferase.;
MGC08416	1	642	Follistatin	Kazal_1 ; TSP-1 type 1 repeat ; Kazal-type serine protease inhibitors			
MGC08417	1	571	Angiotensin-converting enzyme	Peptidase_M2 ; ANGIOTENSIN-CONVERTING ENZYME (DIPEPTIDYL CARBOXYPEPTIDASE) ; Metalloproteases ('zincins'), catalytic domain ; ANGIOTENSIN-CONVERTING-RELATEDENZYME	GO:0005515_F_protein binding;	K01283_04614_Renin-angiotensin system;	3.4.15.1_Peptidyl-dipeptidaseA.;
MGC08420	2	494	Aggrecan core protein	C-type lectin-like ; C_TYPE_LECTIN_1 ; C-TYPE LECTIN SUPERFAMILY MEMBER ; Lectin_C ; GALACTOSE-SPECIFIC C-TYPE LECTIN ; C_TYPE_LECTIN_2			
MGC08425	1	686		HTHFIS			
MGC08449	1	451	Dual serine/threonine and tyrosine protein kinase	Protein kinase-like (PK-like) ; Q6V8K6_CHLRE_Q6V8K6; ; PROTEIN_KINASE_ST ; Pkinase ; CALCIUM/CALMODULIN-DEPENDENT PROTEIN KINASE-RELATED ; PROTEIN_KINASE_DOM			2.7.11.1_Non-specificserine/threonineproteinkinase.;

MGC08451	1	557	Versican core protein (Fragments)	C-type lectin-like ; C-TYPE LECTIN SUPERFAMILY MEMBER ; Lectin_C ; GALACTOSE-SPECIFIC C-TYPE LECTIN ; ANTIFREEZEII ; C_TYPE_LECTIN_2			
MGC08457	1	660	Sarcoplasmic calcium-binding protein	EF_HAND_2 ; EF-hand ; efhand			
MGC08461	1	592	Myosin-10	MYOSIN HEAVY CHAIN, NONMUSCLE OR SMOOTH MUSCLE ; MYOSIN ; Prefoldin ; coiled-coil ; Myosin_tail_1		K10352_04530_Tight junction;	
MGC08466	1	655	Cell division protein kinase 10	Protein kinase-like (PK-like) ; CDK2_HUMAN_P24941; ; Pkinase ; CDC2, MAP KINASE-RELATED ; CDK10/11			2.7.11.22_Cyclin-dependentkinase.;
MGC08470	1	638	Fibrinogen-like protein A	FIBRINOGEN AND FIBRONECTIN ; Fibrinogen C-terminal domain-like ; Fibrinogen_C	GO:0007155_P_cell adhesion; GO:0007411_P_axon guidance;	K06252_04510_Focal adhesion; K06252_04512_ECM-receptor interaction;	
MGC08472	1	647	Tetratricopeptide repeat protein 30A	TPR-like ; coiled-coil			
MGC08476	1	651	Scavenger receptor class A member 5	COLLAGEN ALPHA CHAIN ; COLLAGEN ALPHA 3(IV)-RELATED ; Spermadhesin, CUB domain ; SRCR-like ; SRCR ; SRCR_2 ; SPERACTRCPTR			
MGC08481	1	693		C1q ; GLIACOLIN-RELATED ; CEREBELLIN-RELATED ; TNF-like ; coiled-coil			
MGC08487	1	728					
MGC08503	1	613	Angiopoietin-2	FIBRINOGEN AND FIBRONECTIN ; Fibrinogen C-terminal domain-like ; Fibrinogen_C			
MGC08506	1	564	Hepatic lectin	C-type lectin-like ; C-TYPE LECTIN SUPERFAMILY MEMBER ; Lectin_C ; CD209 ANTIGEN (DENDRITIC CELL-SPECIFIC ICAM-3-GRABBING NONINTEGRIN 1) (DC-SIGN1) ; C_TYPE_LECTIN_2		K06468_04640_Hematopoietic cell lineage;	
MGC08510	1	661	CD209 antigen-like protein E	C-type lectin-like ; ASIALOGLYCOPROTEIN RECEPTOR ; C_TYPE_LECTIN_1 ; C-TYPE LECTIN SUPERFAMILY MEMBER ; Lectin_C ; ANTIFREEZEII ; C_TYPE_LECTIN_2			
MGC08511	1	642		C-type lectin-like ; C-TYPE LECTIN SUPERFAMILY MEMBER ; gb def: Hypothetical protein F52E1.2 ; C_TYPE_LECTIN_2			
MGC08512	1	727	Low-density lipoprotein receptor-related protein 1B	ShK ;			
MGC08514	1	675					
MGC08517	1	716	Bullous pemphigoid antigen 1	Spectrin ; SPECTRIN-LIKE CELL STRUCTURE PROTEIN ; Spectrin repeat ; MICROTUBULE ACTIN CROSSLINKING FACTOR			
MGC08522	1	700	Peptidase inhibitor 16	SCP ; V5ALLERGEN ; CYSTEINE-RICH SECRETORY PROTEIN (CRISP/SCP/TPX1)-RELATED ; V5TPXLIKE ; PR-1-like ; Q6FSU9_EEEEE_Q6FSU9; ; ADH_ZINC ; CRISP SUBFAMILY GLIOMA PATHOGENESIS-RELATED PROTEIN-RELATED	GO:0030133_C_transport vesicle;		
MGC08536	1	714					
MGC08537	1	713	Kelch-like protein 28	POZ domain ; KELCH-RELATED PROTEIN ; BTB			
MGC08549	1	570	Death-associated protein 1	DEATH-ASSOCIATED PROTEIN 1	GO:0008624_P_induction of apoptosis by extracellular signals;		
MGC08555	1	474	Perlucin	REGENERATING GENE TYPE IV-RELATED ; C-type lectin-like ; C_TYPE_LECTIN_1 ; LITHOSTATHINE ; Lectin_C ; C_TYPE_LECTIN_2			

MGC08561	1	658	Sulfated surface glycoprotein 185	BASICPTASE ; IVB3_VIPAA_P00992; ; Kunitz_BPTI ; BASIC PROTEASE INHIBITOR (BPI) (BPTI)-RELATED ; BPTI_KUNITZ_1 ; SERINE-TYPE PROTEASE INHIBITOR-RELATED ; BPTI-like ; BPTI_KUNITZ_2	GO:0030414_F_protease inhibitor activity;	K06238_04510_Focal adhesion; K06238_04512_ECM-receptor interaction; K03909_04610_Complement and coagulation cascades;	
MGC08577	1	743	C-type lectin domain family 4 member M	C-type lectin-like ; C-TYPE LECTIN SUPERFAMILY MEMBER ; Lectin_C ; GALACTOSE-SPECIFIC C-TYPE LECTIN ; C_TYPE_LECTIN_2			
MGC08581	1	715		coiled-coil			
MGC08586	2	670	Rho-related GTP-binding protein RhoQ	P-loop containing nucleoside triphosphate hydrolases ; RAS-RELATED PROTEIN RAC ; RAS-RELATED GTPASE ; Ras	GO:0030036_P_actin cytoskeleton organization and biogenesis; GO:0008360_P_regulation of cell shape; GO:0003924_F_GTPase activity; GO:0005886_C_plasma membrane; GO:0005515_F_protein binding;		
MGC08587	1	532	Deleted in malignant brain tumors 1 protein	SRCR_1 ; SRCR-like ; SRCR ; LYSYL OXIDASE-RELATED ; SRCR_2 ; SPERACTRCPTR ; DELETED IN MALIGNANT BRAIN TUMORS 1			
MGC08592	1	466	Ficolin-1	FIBRINOGEN AND FIBRONECTIN ; Fibrinogen C-terminal domain-like ; Fibrinogen_C ; FICOLIN		K06252_04510_Focal adhesion; K06252_04512_ECM-receptor interaction;	
MGC08602	1	254	Dedicator of cytokinesis protein 1	DEDICATOR OF CYTOKINESIS (DOCK) ; DOCK-1,2,5			
MGC08603	1	678	Protein LITAF homolog	LIPOPOLYSACCHARIDE-INDUCED TRANSCRIPTION FACTOR REGULATING TUMOR NECROSIS FACTOR ALPHA	GO:0005515_F_protein binding;		
MGC08605	1	740	Complement C1q subcomponent subunit B	C1q ; CEREBELLIN-RELATED ; COMPLEMNTC1Q ; TNF-like ; C1Q			
MGC08611	1	654		C1q ; TNF-like			
MGC08613	1	696	Caspase-3	Caspase-like ; IL1BCENZYME ; CASPASE_P20 ; Peptidase_C14 ; CASPASE RELATED ; coiled-coil	GO:0030693_F_caspase activity; GO:0042221_P_response to chemical stimulus; GO:0043065_P_positive regulation of apoptosis;		3.4.22.56_Caspase-3.; 3.4.22.59_Caspase-6.;
MGC08614	1	209		G-PROTEIN COUPLED RECEPTOR-RELATED ; G_PROTEIN_RECEP_F1_2 ; G-PROTEIN COUPLED RECEPTOR ; Family A G protein-coupled receptor-like			
MGC08621	1	794		PROKAR_LIPOPROTEIN			
MGC08626	1	699	Plexin-B	Plexin_cytopl ; PLEXIN ; PLEXIN B	GO:0035021_P_negative regulation of Rac protein signal transduction; GO:0005515_F_protein binding; GO:0007411_P_axon guidance; GO:0008045_P_motor axon guidance; GO:0035025_P_positive regulation of Rho protein signal transduction;	K06820_04360_Axon guidance;	
MGC08640	1	665	Angio-associated migratory cell protein	WD_REPEATS_2 ; SUBWD40 repeat-like ; WD_REPEATS_REGION ; WD40	GO:0008201_F_heparin binding; GO:0005515_F_protein binding; GO:0005829_C_cytosol; GO:0005634_C_nucleus;	K10259_04111_Cell cycle - yeast; K10259_04120_Ubiquitin mediated proteolysis; K03130_03022_Basal transcription factors; K02084_04115_p53 signaling pathway; K02084_04210_Apoptosis; K02084_05222_Small cell lung cancer;	2.7.11.1_Non-specificserine/threonineproteinkinase.;

MGC08652	1	709	Kinase D-interacting substrate of 220 kDa	ANK REPEAT-CONTAINING ; ANKYRIN ; Ank ; ANKYRIN REPEAT-CONTAINING ; ANK_REPEAT ; ANK_REP_REGION ; Ankyrin repeat		K08803_05219_Bladder cancer;	2.7.11.1_Non-specificserine/threonineprotein kinase.;
MGC08655	1	644	Mitochondrial fission 1 protein	TETRATRICOPEPTIDE REPEAT PROTEIN 11 (TPR REPEAT PROTEIN 11) ; TPR ; TPR_2 ; TPR-like ; TPR_REGION			
MGC08661	1	741	Interferon-induced protein 44-like	P-loop containing nucleoside triphosphate hydrolases	GO:0009615_P_response to virus; GO:0006955_P_immune response;		
MGC08662	1	700	Ras-related and estrogen-regulated growth inhibitor	P-loop containing nucleoside triphosphate hydrolases ; RASTRNSFRMNG ; RAS-RELATED GTPASE ; RAS-LIKE, ESTROGEN-REGULATED, GROWTH INHIBITOR ; Ras			
MGC08675	1	468	Perlucin	C-type lectin-like ; C_TYPE_LECTIN_1 ; C-TYPE LECTIN SUPERFAMILY MEMBER ; Lectin_C ; GALACTOSE-SPECIFIC C-TYPE LECTIN ; C_TYPE_LECTIN_2			
MGC08683	1	687	SWI/SNF-related matrix-associated actin-dependent regulator of chromatin subfamily A containing DEAD/H box 1	P-loop containing nucleoside triphosphate hydrolases ; HELICASE SWR1(YEAST)-RELATED ; SNF2_N ; HELICASE_ATP_BIND_1 ; ATP-DEPENDENT HELICASE SMARCA (SWI/SNF-RELATED MATRIX-ASSOCIATED ACTIN-DEPENDENT REGULATOR OF CHROMATIN A)-RELATED			
MGC08688	1	751					
MGC08699	1	501	Perlucin	REGENERATING GENE TYPE IV-RELATED ; C-type lectin-like ; LITHOSTATHINE ; Lectin_C ; C_TYPE_LECTIN_2		K06468_04640_Hematopoietic cell lineage;	
MGC08705	1	721	von Willebrand factor D and EGF domain-containing protein	PROKAR_LIPOPROTEIN			
MGC08717	1	642					
MGC08719	1	633	EF-hand calcium-binding domain-containing protein 4B	coiled-coil ; Domain of the SRP/SRP receptor G-proteins			
MGC08725	1	752	Aquaporin-4	Aquaporin ; AQUAPORIN TRANSPORTER ; MIP: MIP family channel proteins ; Aquaporin-like ; Q6AZD2_BRARE_Q6AZD2; ; MIP ; MINTRINSICP			
MGC08734	1	459	von Willebrand factor D and EGF domain-containing protein				
MGC08737	1	784	Putative ankyrin repeat protein L88	ANKYRIN ; Ank ; ANKYRIN REPEAT-CONTAINING ; ANK_REPEAT ; ANK_REP_REGION ; Ankyrin repeat	GO:0030424_C_axon; GO:0005515_F_protein binding; GO:0007411_P_axon guidance; GO:0050808_P_synapse organization and biogenesis; GO:0045202_C_synapse;	K08803_05219_Bladder cancer;	2.7.11.1_Non-specificserine/threonineprotein kinase.; 2.1.1.43_Histone-lysineN-methyltransferase.;
MGC08754	1	594	Hemicentin-1	TSP_1 ; TSP-1 type 1 repeat ; TITIN ; HEMICENTIN ; TSP1		K06841_04360_Axon guidance; K04659_04350_TGF-beta signaling pathway; K04659_04510_Focal adhesion; K04659_04512_ECM-receptor interaction;	3.4.24.14_ProcollagenN-endopeptidase.;
MGC08757	1	648	Tetratricopeptide repeat protein 35	Protein prenylyltransferase ; TETRATRICOPEPTIDE REPEAT PROTEIN ; coiled-coil ; TPR_REGION			

MGC08762	1	577	Myosin-XV	P-loop containing nucleoside triphosphate hydrolases ; MYOSIN ; MYOSIN XV ; Myosin_head	GO:0046847_P_filopodium formation; GO:0007391_P_dorsal closure; GO:0043025_C_cell soma; GO:0030175_C_filopodium; GO:0005515_F_protein binding; GO:0006886_P_intracellular protein transport;		2.7.11.1_Non-specificserine/threonineproteinkinase.;
MGC08765	1	662		C1q ; TNF-like ; coiled-coil ; C1Q			
MGC08766	1	484	Collectin-12	C-type lectin-like ; C-TYPE LECTIN SUPERFAMILY MEMBER ; Lectin_C ; GALACTOSE-SPECIFIC C-TYPE LECTIN ; ANTIFREEZEII ; C_TYPE_LECTIN_2			
MGC08770	1	502	MIF-like protein mif-2	D-DOPACHROME TAUTOMERASE ; MACROPHAGE MIGRATION INHIBITORY FACTOR RELATED ; Tautomerase/MIF ; MIF	GO:0009792_P_embryonic development ending in birth or egg hatching;	K07253_00350_Tyrosine metabolism; K07253_00360_Phenylalanine metabolism;	5.3.2.1_Phenylpyruvatetautomerase.; 4.1.1.84_D-dopachromedecarboxylase.;
MGC08774	1	672	Calmodulin	EF_HAND_1 ; CALMODULIN ; EF_HAND_2 ; Q39708_DUNSA_Q39708; ; EF-hand ; CALCIUM BINDING PROTEIN ; efhand			2.7.11.1_Non-specificserine/threonineproteinkinase.;
MGC08783	1	633	Proline-rich protein 2				
MGC08785	1	681	Macrophage migration inhibitory factor	MACROPHAGE MIGRATION INHIBITORY FACTOR RELATED ; Tautomerase/MIF ; MIF		K07253_00350_Tyrosine metabolism; K07253_00360_Phenylalanine metabolism;	5.3.2.1_Phenylpyruvatetautomerase.;
MGC08787	1	603	Calmodulin	EF_HAND_1 ; CALMODULIN ; EF_HAND_2 ; Q76LB7_STRIE_Q76LB7; ; EF-hand ; CALCIUM BINDING PROTEIN ; efhand			2.7.11.1_Non-specificserine/threonineproteinkinase.;
MGC08789	1	486	Heavy metal-binding protein HIP				
MGC08803	1	710					
MGC08805	2	769	DnaJ homolog dnj-10	DNAJ/HSP40 ; DNAJPROTEIN ; DNAJ_2 ; DnaJ ; CHAPERONE PROTEIN DNAJ ; Chaperone J-domain ; coiled-coil			
MGC08806	1	477	Tubulin alpha chain	ALPHATUBULIN ; TUBULIN ALPHA-1 CHAIN ; Tubulin C-terminal domain-like ; coiled-coil ; TUBULIN		K07374_04540_Gap junction;	
MGC08815	1	635	Inhibitor of apoptosis protein	BIR ; INHIBITOR OF APOPTOSIS ; Inhibitor of apoptosis (IAP) repeat ; INHIBITOR OF APOPTOSIS PROTEIN 1 AND 2, IAP1, IAP2 ; BIR_REPEAT_2	GO:0005737_C_cytoplasm; GO:0043027_F_caspase inhibitor activity; GO:0005515_F_protein binding; GO:0005829_C_cytosol ; GO:0006916_P_anti-apoptosis;	K04725_04120_Ubiquitin mediated proteolysis; K04725_04210_Apoptosis; K04725_04510_Focal adhesion; K04725_05222_Small cell lung cancer;	
MGC08819	1	697	Peptidylprolyl isomerase domain and WD repeat-containing protein 1	PEPTIDYL-PROLYL CIS-TRANS ISOMERASE-LIKE ; Cyclophilin-like ; CSAPPISMRASE ; CYCLOPHILIN ; Pro_isomerase ; CSA_PPIASE_2			5.2.1.8_Peptidylprolylisomerase.;
MGC08821	1	707	Complement C1q tumor necrosis factor-related protein 3	C1q ; C1Q-RELATED FACTOR ; COLLAGEN ALPHA CHAIN ; COMPLEMNTC1Q ; TNF-like ; coiled-coil ; C1Q			
MGC08822	1	514	Glutathione peroxidase 6	GSHPx ; Thioredoxin-like ; GLUTATHIONE PEROXIDASE		K00432_00480_Glutathione metabolism; K00432_00590_Arachidonic acid metabolism;	1.11.1.9_Glutathioneperoxidase.;
MGC08823	1	686	SH3 domain-binding glutamic acid-rich protein	SH3 DOMAIN-BINDING GLUTAMIC ACID-RICH-LIKE PROTEIN ; Thioredoxin-like ; SH3BGR	GO:0005737_C_cytoplasm; GO:0005515_F_protein binding; GO:0006461_P_protein complex assembly; GO:0005634_C_nucleus; GO:0005070_F_SH3/SH2 adaptor activity;		
MGC08831	1	630	Metalloproteinase inhibitor 1	TIMP-like			

MGC08848	1	734	Ubiquitin-conjugating enzyme E2 D2	UBIQUITIN_CONJUGAT_2 ; UBIQUITIN-CONJUGATING ENZYME E2 ; UBC-like ; Q7ZUK1_BRARE_Q7ZUK1; ; UQ_con ; UBIQUITIN_CONJUGAT_1	GO:0005515_F_protein binding;	K06689_04120_Ubiquitin mediated proteolysis;	6.3.2.19_Ubiquitin--proteinligase.;
MGC08860	2	896	Solute carrier family 25 member 38-B	MITOCHONDRIAL CARRIER PROTEIN ; Mitochondrial carrier ; SOLCAR ; MITOCARRIER ; MITOCHONDRIAL CARRIER PROTEIN RELATED ; Mito_carr			
MGC08864	1	175	DBH-like monooxygenase protein 1	Cu2_monooxygen ; DOPAMINE BETA HYDROXYLASE RELATED ; PHM/PNGase F ; DBMONOXGNASE		K00503_00350_Tyrosine metabolism;	1.14.17.1_Dopaminebeta-monooxygenase.;
MGC08875	1	687	Mammalian ependymin-related protein 1				
MGC08883	1	723	Cathepsin L	Cysteine proteinases ; Peptidase_C1 ; Q80X23_MOUSE_Q80X23; ; CYSTEINE PROTEASE FAMILY C1-RELATED ; CATHEPSIN L ; THIOL_PROTEASE_ASN		K01365_04612_Antigen processing and presentation;	3.4.22.38_CathepsinK.; 3.4.22.15_CathepsinL.;
MGC08884	1	633	Max-like protein X	BASIC HELIX-LOOP-HELIX ZIP TRANSCRIPTION FACTOR ; HLH ; BHLHZIP TRANSCRIPTION FACTOR BIGMAX ; HLH, helix-loop-helix DNA-binding domain ; coiled-coil	GO:0005737_C_cytoplasm; GO:0006913_P_nucleocytoplasmic transport; GO:0005515_F_protein binding; GO:0005634_C_nucleus;		
MGC08887	1	690	Galectin-9	Concanavalin A-like lectins/glucanases ; GALECTIN ; Gal-bind_lectin			3.1.1.5_Lysophospholipase.;
MGC08888	1	746	Regulator of G-protein signaling 12	Q6T9C3_BRARE_Q6T9C3; ; REGULATOR OF G-PROTEIN SIGNALING 12 ; RGSPROTEIN ; REGULATOR OF G PROTEIN SIGNALING, RGS ; RGS ; Regulator of G-protein signaling, RGS		K07524_04360_Axon guidance;	
MGC08897	1	628	PDZ domain-containing protein 11	PDZ ; SUBPDZ domain-like	GO:0016323_C_basolateral plasma membrane; GO:0005515_F_protein binding; GO:0008022_F_protein C-terminus binding; GO:0005829_C_cytosol ;	K08018_04010_MAPK signaling pathway;	3.1.3.48_Protein-tyrosine-phosphatase.;
MGC08898	1	694	Methyl-CpG-binding domain protein 3	MBD ; DNA-binding domain ; METHYL-CPG BINDING PROTEIN, MBD	GO:0003677_F_DNA binding; GO:0005515_F_protein binding;		
MGC08899	1	634	Adapter molecule Crk	CRK_DROME_Q9XYM0; ; SH2-SH3 ADAPTOR PROTEIN-RELATED ; SH3 ; SH2DOMAIN ; SH3DOMAIN ; SH3_1 ; SH2 domain ; CRK ; SH2			2.7.10.2_Non-specificprotein-tyrosinekinase.;
MGC08910	2	743	MAP kinase-activated protein kinase 2	Protein kinase-like (PK-like) ; PROTEIN_KINASE_ST ; PROTEIN_KINASE_ATP ; Pkinase ; Q80ZF4_RAT_Q80ZF4; ; CALCIUM/CALMODULIN-DEPENDENT PROTEIN KINASE-RELATED ; MAP KINASE-ACTIVATED PROTEIN KINASE (MAPKAPK) ; PROTEIN_KINASE_DOM		K04443_04010_MAPK signaling pathway; K04443_04370_VEGF signaling pathway; K04444_04010_MAPK signaling pathway; K04444_04370_VEGF signaling pathway;	2.7.11.1_Non-specificserine/threonineproteinkinase.;
MGC08911	1	695	von Willebrand factor D and EGF domain-containing protein	EGF_3 ; ASX_HYDROXYL ; EGF_1 ; EGF_2			
MGC08916	1	686	ADP-ribosylation factor-like protein 2	ADP-RIBOSYLATION FACTOR-LIKE 2, ARL2 ; P-loop containing nucleoside triphosphate hydrolases ; RASTRNSFRMNG ; Arf ; small_GTP: small GTP-binding protein domain ; SAR1GTPBP ; ARF ; ADP RIBOSYLATION FACTOR-RELATED	GO:0007021_P_tubulin folding; GO:0005515_F_protein binding; GO:0005095_F_GTPase inhibitor activity;		

MGC08918	1	642	Apoptotic chromatin condensation inducer in the nucleus	coiled-coil				
MGC08931	1	729	STE20-like serine/threonine-protein kinase	Protein kinase-like (PK-like) ; PROTEIN_KINASE_ST ; PROTEIN_KINASE_ATP ; POLO KINASE KINASE 1 ; Pkinase ; MAPKK-RELATED SERINE/THREONINE PROTEIN KINASES ; Q6YHU9_SCHMA_Q6YHU9; ; PROTEIN_KINASE_DOM		K04414_04010_MAPK signaling pathway;	2.7.11.1_Non-specificserine/threonineproteinkinase.;	
MGC08934	1	415	Eukaryotic initiation factor 4A-III	Q_MOTIF ; P-loop containing nucleoside triphosphate hydrolases ; HELICASE_ATP_BIND_1 ; EUKARYOTIC INITIATION FACTOR 4A ; DEAD BOX ATP-DEPENDENT RNA HELICASE ; DEAD	GO:0005737_C_cytoplasm; GO:0005524_F_ATP binding; GO:0017148_P_negative regulation of translation; GO:0005515_F_protein binding; GO:0004004_F_ATP-dependent RNA helicase activity; GO:0008143_F_poly(A) binding;			
MGC08941	1	655	Prominin-1	PROMININ (PROM) PROTEIN	GO:0005887_C_integral to plasma membrane; GO:0005902_C_microvillus; GO:0005615_C_extracellular space; GO:0005903_C_brush border;			
MGC08946	1	555	Angiomotin	ANGIOMOTIN ; coiled-coil		K06104_04530_Tight junction;		
MGC08956	1	686		G_PROTEIN_RECEP_F1_1				
MGC08964	1	653	Latrophilin-2	EGF, LATROPHILIN AND SEVEN TRANSMEMBRANE DOMAIN CONTAINING 1 ; G-PROTEIN COUPLED RECEPTOR ; GPS	GO:0004930_F_G-protein coupled receptor activity; GO:0016021_C_integral to membrane; GO:0007186_P_G-protein coupled receptor protein signaling pathway;			
MGC08969	1	698	Complement C1q-like protein 3	C1q ; C1Q-RELATED FACTOR ; COLLAGEN ALPHA CHAIN ; COMPLEMNTC1Q ; TNF-like ; C1Q	GO:0005515_F_protein binding;	K07296_03320_PPAR signaling pathway; K07296_04920_Adipocytokine signaling pathway; K07296_04930_Type II diabetes mellitus; K03987_04610_Complement and coagulation cascades; K03987_05010_Alzheimers disease;		
MGC08970	1	603						
MGC08974	1	726	Heavy metal-binding protein HIP	C1q ; TNF-like				
MGC08975	1	664	ADP-ribosylation factor 3	ADP-RIBOSYLATION FACTOR, ARF ; P-loop containing nucleoside triphosphate hydrolases ; RASTRNSFRMNG ; Arf ; small_GTP: small GTP-binding protein domain ; SAR1GTPBP ; ARF ; ADP RIBOSYLATION FACTOR-RELATED				
MGC08984	1	754	Discoidin, CUB and LCCL domain-containing protein 2	LACTADHERIN/MFG-E8 ; DISCOIDIN, CUB, EGF, LAMININ , AND ZINC METALLOPROTEASE DOMAIN ; FA58C_3 ; Galactose-binding domain-like ; F5_F8_type_C		K03899_04610_Complement and coagulation cascades; K03902_04610_Complement and coagulation cascades;		
MGC08985	1	735	Probable G-protein coupled receptor 157					
MGC08987	1	761	Golgi-associated plant pathogenesis-related protein 1	SCP ; GOLGI-ASSOCIATED PLANT PATHOGENESIS-RELATED PROTEIN 1 (GOLGI-ASSOCIATED PR-1 PROTEIN) ; CYSTEINE-RICH SECRETORY PROTEIN (CRISP/SCP/TPX1)-RELATED ; PR-1-like				
MGC08988	1	598	Dynactin subunit 2	DYNACTIN SUBUNIT ; Dynamitin	GO:0040023_P_establishment of nucleus localization;			

MGC09001	1	694	Neuronal calcium sensor 2	EF_HAND_1 ; Q8HZK3_PIG_Q8HZK3; ; RECOVERIN ; EF_HAND_2 ; EF-hand ; CALCIUM BINDING PROTEINS ; efhand			1.6.3.1_NAD(P)Hoxidase.;
MGC09039	1	571					
MGC09042	1	664	C-type lectin domain family 4 member E	C-type lectin-like ; C_TYPE_LECTIN_1 ; C-TYPE LECTIN SUPERFAMILY MEMBER ; Lectin_C ; CD209 ANTIGEN (DENDRITIC CELL-SPECIFIC ICAM-3-GRABBING NONINTEGRIN 1) (DC-SIGN1) ; ANTIFREEZEII ; C_TYPE_LECTIN_2	GO:0008329_F_pattern recognition receptor activity; GO:0006910_P_phagocytosis, recognition; GO:0030169_F_low-density lipoprotein binding; GO:0006955_P_immune response;		
MGC09044	1	736	Complement C1q-like protein 2	C1q ; CEREBELLIN-RELATED ; TNF-like ; coiled-coil ; C1Q			
MGC09046	1	727	Tyrosine-protein phosphatase non-receptor type 11	PROTEIN TYROSINE PHOSPHATASE N11 (SHP2) ; PROTEIN-TYROSINE PHOSPHATASE ; TYR_PHOSPHATASE_PTP ; PRTYPHPHTASE ; (Phosphotyrosine protein) phosphatases II ; Y_phosphatase	GO:0007165_P_signal transduction; GO:0004726_F_non-membrane spanning protein tyrosine phosphatase activity; GO:0004725_F_protein tyrosine phosphatase activity; GO:0005515_F_protein binding;		3.1.3.48_Protein-tyrosine-phosphatase.;
MGC09053	1	674		CBM_14 ; CHIT_BIND_II ; Invertebrate chitin-binding proteins			
MGC09056	1	767	RCC1 domain-containing protein 1	REGULATOR OF CHROMOSOME CONDENSATION ; RCC1_3 ; RCC1_2 ; REGULATOR OF CHROMOSOME CONDENSATION-RELATED ; RCCNDNSATION ; RCC1/BLIP-II	GO:0005515_F_protein binding;	K10614_04120_Ubiquitin mediated proteolysis; K10615_04120_Ubiquitin mediated proteolysis;	2.7.11.1_Non-specificserine/threonineproteinkinase.;
MGC09063	1	610	Ankyrin repeat domain-containing protein 57	ANK REPEAT-CONTAINING ; ANKYRIN ; Ank ; ANKYRIN REPEAT-CONTAINING ; ANK_REPEAT ; ANK_REP_REGION ; Ankyrin repeat		K06272_03320_PPAR signaling pathway; K06272_04510_Focal adhesion; K06272_05213_Endometrial cancer;	2.7.11.1_Non-specificserine/threonineproteinkinase.;
MGC09072	1	672		Immunoglobulin			
MGC09080	1	257	Plasminogen	KRINGLE_1 ; HGFL_HUMAN_P26927; ; SERINE PROTEASE-RELATED ; Kringle-like ; PLASMINOGEN ; KRINGLE_2 ; KRINGLE ; Kringle	GO:0007566_P_embryo implantation;	K05460_04060_Cytokine-cytokine receptor interaction; K05460_04510_Focal adhesion; K05460_05211_Renal cell carcinoma; K05460_05218_Melanoma; K01315_04080_Neuroactive ligand-receptor interaction; K01315_04610_Complement and coagulation cascades;	3.4.21.7_Plasmin.;
MGC09082	2	670	Thioredoxin reductase 3 (Fragment)	Pyr_redox_2 ; Glutaredoxin ; GLUTAREDOXIN ; GLUTAREDOXIN-1, GRX1 ; Thioredoxin-like ; GRX_euk: Glutaredoxin ; PYRIDINE_REDOX_1 ; FAD/NAD(P)-binding domain ; PNDRDTASEI		K00384_00240_Pyrimidine metabolism;	1.8.1.9_Thioredoxin-disulfidereductase.;
MGC09083	1	757	Fucolectin-3	Galactose-binding domain-like			
MGC09085	1	638	Guanine nucleotide-binding protein G(i) subunit alpha	Transducin (alpha subunit), insertion domain ; GTP-BINDING PROTEIN ALPHA SUBUNIT ; G-alpha ; GPROTEINA ; P-loop containing nucleoside triphosphate hydrolases ; GTP-BINDING PROTEIN (I) ALPHA-1 AND ALPHA-3 SUBUNIT (GNAI1,3) ; GPROTEINAI		K04630_04360_Axon guidance; K04630_04530_Tight junction; K04630_04540_Gap junction; K04630_04670_Leukocyte transendothelial migration; K04630_04730_Long-term depression; K04630_04914_Progesterone-mediated oocyte maturation; K04630_04916_Melanogenesis;	

MGC09088	1	421	Structural maintenance of chromosomes protein 4	SMC_N ; P-loop containing nucleoside triphosphate hydrolases ; STRUCTURAL MAINTENANCE OF CHROMOSOMES SMC4 ; STRUCTURAL MAINTENANCE OF CHROMOSOMES SMC FAMILY MEMBER		K06675_04111_Cell cycle - yeast;	
MGC09089	1	605		Immunoglobulin			
MGC09090	2	809	Casein kinase II subunit beta	CASEIN KINASE II BETA CHAIN ; Casein kinase II beta subunit ; CK2_BETA ; CK_II_beta ; CASNKINASEII	GO:0004682_F_protein kinase CK2 activity; GO:0030177_P_positive regulation of Wnt receptor signaling pathway; GO:0007249_P_I-kappaB kinase/NF-kappaB cascade;	K03115_04310_Wnt signaling pathway; K03115_04520_Adherens junction; K03115_04530_Tight junction;	
MGC09091	1	680	Pre-B-cell leukemia transcription factor 2	HOMEOBOX PROTEIN TRANSCRIPTION FACTORS ; PRE-B-CELL LEUKEMIA TRANSCRIPTION FACTOR 1, 2, 3, 4 (PBX) ; PBC			
MGC09093	1	673	Neurogenic locus notch homolog protein 1	EGF/Laminin ; NOTCH ; EGF-LIKE DOMAIN PROTEIN ; EGF ; EGF_3 ; ASX_HYDROXYL ; C_TYPE_LECTIN_1 ; EGF_1 ; EGF_2	GO:0001840_P_neural plate development; GO:0048793_P_pronephros development; GO:0001889_P_liver development;	K02599_04320_Dorso-ventral axis formation; K02599_04330_Notch signaling pathway; K06052_04330_Notch signaling pathway;	2.7.10.1_Receptorprotein-tyrosinekinase.; 3.4.21.6_Coagulationfactor Xa.;
MGC09097	1	630	Complement C1q tumor necrosis factor-related protein 3	C1q ; CEREBELLIN-RELATED ; COMPLEMNTC1Q ; TNF-like ; C1Q			
MGC09100	1	623	Protein TANC2	TPR_1 ; ANKYRIN REPEAT-CONTAINING ; TPR DOMAIN, ANKYRIN-REPEAT AND COILED-COIL-CONTAINING ; TPR_2 ; TPR-like ; ANK_REP_REGION ; Ankyrin repeat ; TPR_REGION			
MGC09101	1	705	Leucine-rich PPR motif-containing protein, mitochondrial	PPR ; PENTATRICOPEPTIDE REPEAT-CONTAINING PROTEIN ; SNF1-LIKE PROTEIN KINASE/SALT INDUCIBLE PROTEIN/LEUCINE-RICH PPR MOTIF ; PPR: pentatricopeptide repeat domain			
MGC09106	1	726	Fibrinogen-like protein A	FIBRINOGEN AND FIBRONECTIN ; Fibrinogen C-terminal domain-like ; Fibrinogen_C		K06252_04510_Focal adhesion; K06252_04512_ECM-receptor interaction;	
MGC09118	1	621	Chromatin accessibility complex protein 1	Histone-fold ; HISTONE-LIKE TRANSCRIPTION FACTOR CCAAT-RELATED ; CBFD_NFYB_HMF ; DNA POLYMERASE EPSILON SUBUNIT	GO:0005515_F_protein binding;	K03506_03030_DNA replication; K03506_03410_Base excision repair; K03506_03420_Nucleotide excision repair;	2.7.7.7_DNA-directedDNApolymerase.;
MGC09123	2	716	Ocs element-binding factor 1	CAMP-RESPONSE ELEMENT BINDING PROTEIN-RELATED ; CYCLIC-AMP-DEPENDENT TRANSCRIPTION FACTOR ATF-6 ; bZIP_1 ; BZIP_BASIC ; BZIP ; coiled-coil			
MGC09124	1	761	Hepatic lectin	C-type lectin-like ; C_TYPE_LECTIN_1 ; C-TYPE LECTIN SUPERFAMILY MEMBER ; Lectin_C ; GALACTOSE-SPECIFIC C-TYPE LECTIN ; C_TYPE_LECTIN_2	GO:0030246_F_carbohydrate binding;	K06468_04640_Hematopoietic cell lineage;	
MGC09127	1	728					
MGC09128	1	782	Arylsulfatase B	SULFATASE_2 ; Alkaline phosphatase-like ; Sulfatase ; SULFATASE ; ARYLSULFATASE B	GO:0004065_F_arylsulfatase activity;	K01135_00531_Glycosaminoglycan degradation; K01135_01032_tba; K01131_00150_Androgen and estrogen metabolism; K01130_00150_Androgen and estrogen metabolism; K01130_00600_Sphingolipid metabolism;	3.1.6.1_Arylsulfatase.; 3.1.6.12_N-acetylgalactosamine-4-sulfatase.;

MGC09136	1	725	Methionyl-tRNA synthetase, cytoplasmic	TRBD ; tRNA_bind ; Nucleic acid-binding proteins ; ENDOTHELIAL-MONOCYTE ACTIVATING POLYPEPTIDE II-RELATED (EMAP-II-RELATED)UBFAMILY E MEMBER 1) ; ISOLEUCYL, LEUCYL, TYROSYL, VALYL AND METHIONYL-TRNA SYNTHETASES	GO:0040010_P_positive regulation of growth rate; GO:0002119_P_nematode larval development; GO:0000003_P_reproduction; GO:0009792_P_embryonic development ending in birth or egg hatching; GO:0040007_P_growth;	K01866_00400_Phenylalanine, tyrosine and tryptophan biosynthesis; K01866_00970_Aminoacyl-tRNA biosynthesis; K01874_00271_Methionine metabolism; K01874_00450_Selenoamino acid metabolism; K01874_00970_Aminoacyl-tRNA biosynthesis;	6.1.1.10_Methionine--tRNAligase.; 6.1.1.1_Tyrosine--tRNAligase.;
MGC09137	1	578	Collagen alpha-2(VIII) chain	C1q ; COLLAGEN ALPHA 1(VIII) CHAIN ; COLLAGEN ALPHA CHAIN ; COMPLEMNTC1Q ; TNF-like ; C1Q		K03986_04610_Complement and coagulation cascades; K03986_05010_Alzheimers disease; K03987_04610_Complement and coagulation cascades; K03987_05010_Alzheimers disease;	
MGC09138	1	664	Putative ankyrin repeat protein RF_0381	ANKYRIN ; Ank ; ANKYRIN REPEAT-CONTAINING ; ANK_REPEAT ; ANKYRIN REPEAT DOMAIN PROTEIN 28 ; ANK_REP_REGION ; Ankyrin repeat			2.4.2.30_NAD(+)ADP-ribosyltransferase.; 2.7.11.1_Non-specificserine/threonineproteinkinase.; 2.1.1.43_Histone-lysineN-methyltransferase.;
MGC09141	1	573	Zinc finger and BTB domain-containing protein 41	ZINC_FINGER_C2H2_1 ; C2H2 and C2HC zinc fingers ; gb def: Zinc finger protein 450 (Brain specific protein 1) ; zf-C2H2 ; ZINC FINGER PROTEINS ; ZINC_FINGER_C2H2_2			
MGC09145	1	679	Toxin CaTX-A				
MGC09148	1	642	Complement C1q-like protein 2	C1q ; TNF-like ; C1Q			
MGC09150	1	768	Probable E3 ubiquitin-protein ligase HERC3	RCC1_3 ; RCC1_2 ; HECT DOMAIN AND RCC1-LIKE DOMAIN PROTEIN 3,4 (HERC3, HERC4) ; HECT DOMAIN UBIQUITIN-PROTEIN LIGASE ; RCC1 ; RCC1/BLIP-II		K10614_04120_Ubiquitin mediated proteolysis; K10595_04120_Ubiquitin mediated proteolysis; K10615_04120_Ubiquitin mediated proteolysis; K04575_05030_tba;	2.7.11.1_Non-specificserine/threonineproteinkinase.;
MGC09152	2	1175	Apoptosis regulator BAX	Bcl2 related apoptosis regulator ; BCL2FAMILY ; BCL2_FAMILY ; APOPTOSIS REGULATOR BAX ; Bcl-2 inhibitors of programmed cell death ; Bcl-2 ; BCL-2 RELATED			
MGC09153	1	735					
MGC09155	1	630	Low affinity immunoglobulin epsilon Fc receptor	REGENERATING GENE TYPE IV-RELATED ; C-type lectin-like ; LITHOSTATHINE ; Lectin_C ; C_TYPE_LECTIN_2			
MGC09157	1	710	Heat shock 70 kDa protein 12A	Actin-like ATPase domain	GO:0048514_P_blood vessel morphogenesis; GO:0002040_P_sprouting angiogenesis;		
MGC09158	1	598	Microfibril-associated glycoprotein 4	Snake toxin-like ; FIBRINOGEN AND FIBRONECTIN ; Fibrinogen C-terminal domain-like ; Fibrinogen_C	GO:0001527_C_microfibril; GO:0007155_P_cell adhesion;	K06252_04510_Focal adhesion; K06252_04512_ECM-receptor interaction;	
MGC09162	1	655	Fibrinogen-like protein 1	FIBRINOGEN AND FIBRONECTIN ; Fibrinogen C-terminal domain-like ; Fibrinogen_C ; coiled-coil		K06252_04510_Focal adhesion; K06252_04512_ECM-receptor interaction;	
MGC09172	1	709		TNF-like			
MGC09184	1	658	WD repeat-containing protein 33	WD40 REPEAT PROTEIN	GO:0005515_F_protein binding; GO:0006301_P_postreplication repair; GO:0007283_P_spermatogenesis; GO:0005634_C_nucleus;		

MGC09185	1	661	Phospholipid scramblase 2	Scramblase ; PHOSPHOLIPID SCRAMBLASE 1, 2 ; PHOSPHOLIPID SCRAMBLASE-RELATED	GO:0017128_F_phospholipid scramblase activity; GO:0030168_P_platelet activation; GO:0005886_C_plasma membrane; GO:0017121_P_phospholipid scrambling; GO:0005509_F_calcium ion binding; GO:0005515_F_protein binding; GO:0007268_P_synaptic transmission;		
MGC09186	1	733	Doublecortin domain-containing protein 2				
MGC09193	1	711	Arylsulfatase B	Alkaline phosphatase-like ; Sulfatase ; SULFATASE ; ARYLSULFATASE B	GO:0004065_F_arylsulfatase activity;	K01134_00600_Sphingolipid metabolism; K01135_00531_Glycosaminoglycan degradation; K01135_01032_tba; K01130_00150_Androgen and estrogen metabolism; K01130_00600_Sphingolipid metabolism;	3.1.6.1_Arylsulfatase.; 3.1.6.12_N-acetylgalactosamine-4-sulfatase.;
MGC09209	1	707					
MGC09212	1	599	Collagen alpha-1(VIII) chain	C1q ; GLIACOLIN-RELATED ; CEREBELLIN-RELATED ; COMPLEMNTC1Q ; TNF-like ; C1Q	GO:0005581_C_collagen; GO:0001501_P_skeletal development;	K07296_03320_PPAR signaling pathway; K07296_04920_Adipocytokine signaling pathway; K07296_04930_Type II diabetes mellitus;	
MGC09215	1	715	Serine-rich adhesin for platelets				
MGC09223	1	424	Complement C1q-like protein 3	C1q ; C1Q-RELATED FACTOR ; COLLAGEN ALPHA CHAIN ; COMPLEMNTC1Q ; TNF-like ; C1Q	GO:0005515_F_protein binding;		
MGC09231	1	596	Heterogeneous nuclear ribonucleoprotein A1	RRM ; RRM_1 ; HETEROGENEOUS NUCLEAR RIBONUCLEOPROTEIN ; RNA-binding domain, RBD ; RNA-BINDING PROTEIN		K03102_04320_Dorso-ventral axis formation;	5.2.1.8_Peptidylprolylisomerase.;
MGC09233	1	725	Mediator of RNA polymerase II transcription subunit 1.1				
MGC09238	1	710					
MGC09239	1	638	Dermal papilla-derived protein 6 homolog	ShK ; LECTIN_LEGUME_BETA ; IG_MHC			
MGC09243	1	588		Toll/Interleukin receptor TIR domain			
MGC09244	1	599	Titin	Immunoglobulin ; IG_LIKE ; I-set ; TITIN ; KETTIN/TITIN-RELATED PROTEIN		K00907_04020_Calcium signaling pathway; K00907_04510_Focal adhesion; K00907_04810_Regulation of actin cytoskeleton; K06765_04360_Axon guidance; K06765_05210_Colorectal cancer; K06765_04360_Axon guidance;	2.7.11.1_Non-specificserine/threonineproteinkinase.; 2.7.11.18_[Myosinlight-chain]kinase.;
MGC09246	1	630	Aminoglycoside phosphotransferase domain-containing protein 1	Protein kinase-like (PK-like) ; APH ; RCC1_2		K02204_00260_Glycine, serine and threonine metabolism;	2.7.1.39_Homoserinekinase.;
MGC09254	1	613	Leukocyte elastase inhibitor	Serpins ; SERINE PROTEASE INHIBITOR, SERPIN			
MGC09257	1	688	NAD-dependent deacetylase 2	CHROMATIN REGULATORY PROTEIN SIR2 ; DHS-like NAD/FAD-binding domain ; SIRTUIN ; SIR2		K01463_00760_Nicotinate and nicotinamide metabolism; K01463_00770_Pantothenate and CoA biosynthesis; K01463_00930_Caprolactam degradation;	

MGC09267	1	736	Putative inhibitor of apoptosis	BIR_REPEAT_1 ; BIR ; INHIBITOR OF APOPTOSIS ; Inhibitor of apoptosis (IAP) repeat ; INHIBITOR OF APOPTOSIS PROTEIN 1 AND 2, IAP1, IAP2 ; BIR_REPEAT_2	GO:0007166_P_cell surface receptor linked signal transduction; GO:0005737_C_cytoplasm; GO:0004842_F_ubiquitin-protein ligase activity; GO:0005515_F_protein binding; GO:0005634_C_nucleus; GO:0006916_P_anti-apoptosis;	K04725_04120_Ubiquitin mediated proteolysis; K04725_04210_Apoptosis; K04725_04510_Focal adhesion; K04725_05222_Small cell lung cancer;	
MGC09268	1	774	Coactosin-like protein	CORTACTIN ; Actin depolymerizing proteins ; DREBRIN-RELATED ; Cofilin_ADF			
MGC09277	1	708	von Willebrand factor D and EGF domain-containing protein	PROKAR_LIPOPROTEIN			
MGC09283	2	1082	Vigilin	Eukaryotic type KH-domain (KH-domain type I) ; KH_TYPE_1 ; KH_1 ; HIGH DENSITY LIPOPROTIEN BINDING PROTEIN / VIGILIN ; SCP160 ; coiled-coil			
MGC09284	1	723	Collagen alpha-1(X) chain	C1q ; COLLAGEN ALPHA 1(VIII) CHAIN ; COLLAGEN ALPHA CHAIN ; COMPLEMNTC1Q ; TNF-like ; C1Q	GO:0005581_C_collagen; GO:0001501_P_skeletal development; GO:0005938_C_cell cortex;		
MGC09286	1	630	Fucolectin-4	Galactose-binding domain-like			
MGC09294	1	542	BRCA1-associated RING domain protein 1	ANKYRIN ; Ank ; ANKYRIN REPEAT-CONTAINING ; ANK_REPEAT ; ANK_REP_REGION ; SERINE/THREONINE-PROTEIN KINASE RIPK4 ; Ankyrin repeat			2.4.2.30_NAD(+)ADP-ribosyltransferase.; 2.7.11.1_Non-specificserine/threonineproteinkinase.;
MGC09295	1	640	Baculoviral IAP repeat-containing protein 3	RING/U-box ; INHIBITOR OF APOPTOSIS ; zf-C3HC4 ; ZF_RING_2 ; INHIBITOR OF APOPTOSIS PROTEIN 1 AND 2, IAP1, IAP2	GO:0007166_P_cell surface receptor linked signal transduction; GO:0005737_C_cytoplasm; GO:0004842_F_ubiquitin-protein ligase activity; GO:0005515_F_protein binding; GO:0005634_C_nucleus; GO:0006916_P_anti-apoptosis;	K04725_04120_Ubiquitin mediated proteolysis; K04725_04210_Apoptosis; K04725_04510_Focal adhesion; K04725_05222_Small cell lung cancer;	
MGC09297	1	563	Phospholipid scramblase 1	Scramblase ; PHOSPHOLIPID SCRAMBLASE 1, 2 ; PHOSPHOLIPID SCRAMBLASE-RELATED	GO:0030099_P_myeloid cell differentiation; GO:0007268_P_synaptic transmission;		
MGC09302	1	696	Ribonuclease Oy	Ribonuclease Rh-like ; RIBONUCLEASE T2 ; Ribonuclease_T2 ; RNASE_T2_2 ; RNASE_T2_1			3.1.27.1_RibonucleaseT(2).;
MGC09305	1	714	Collagen alpha-1(XII) chain	VWFA ; VON WILENBRAND FACTOR RELATED ; vWA-like ; VWA ; VWFADOMAIN	GO:0030199_P_collagen fibril organization; GO:0030020_F_extracellular matrix structural constituent conferring tensile strength; GO:0001501_P_skeletal development; GO:0005595_C_collagen type XII;	K06238_04510_Focal adhesion; K06238_04512_ECM-receptor interaction;	
MGC09309	1	633	DnaJ homolog subfamily A member 1	ZF_CR ; DnaJ/Hsp40 cysteine-rich domain ; HSP40, SUBFAMILY A, MEMBERS 1,2,4 ; DNAJ/HSP40 ; DNAJPROTEIN ; DNAJ_2 ; DnaJ ; DnaJ_CXXCXGXG ; DNAJ_1 ; Chaperone J-domain			
MGC09317	1	696	Neurotrypsin	GALECTIN-3 BINDING PROTEIN ; SRCR_1 ; SRCR-like ; SRCR ; LYSYL OXIDASE-RELATED ; SRCR_2 ; SPERACTRCPTR			
MGC09318	1	703					

MGC09325	1	620		SAM/Pointed domain			
MGC09326	1	641					
MGC09337	1	543	Uncharacterized protein ZK673.1	ShK			
MGC09341	1	733					
MGC09345	1	664		HSP70_3			
MGC09348	1	729	Kelch-like protein 13	Galactose oxidase, central domain ; KELCH-RELATED PROTEIN ; Kelch_1		K10447_04120_Ubiquitin mediated proteolysis;	
MGC09350	1	657	Neurocan core protein	C-type lectin-like ; C_TYPE_LECTIN_1 ; C-TYPE LECTIN SUPERFAMILY MEMBER ; Lectin_C ; LOW AFFINITY IMMUNOGLOBULIN EPSILON FC RECEPTOR (CD23 ANTIGEN) ; ANTIFREEZEII ; C_TYPE_LECTIN_2			
MGC09355	1	633	Acetylcholine receptor subunit alpha-type acr-16	NICOTINIC ACETYLCHOLINE RECEPTOR ALPHA 7 ; Neur_chan_LBD ; NEUROTRANSMITTER GATED ION CHANNEL ; Nicotinic receptor ligand binding domain-like		K04809_04020_Calcium signaling pathway;	
MGC09361	1	557	Perlucin	C-type lectin-like ; C_TYPE_LECTIN_1 ; C-TYPE LECTIN SUPERFAMILY MEMBER ; Lectin_C ; LOW AFFINITY IMMUNOGLOBULIN EPSILON FC RECEPTOR (CD23 ANTIGEN) ; ANTIFREEZEII ; C_TYPE_LECTIN_2			
MGC09382	1	559	Uncharacterized protein sll1388	UNVRSLSTRESS ; Adenine nucleotide alpha hydrolases-like ; Usp			
MGC09385	1	669	Vesicle-trafficking protein SEC22b-B	SNARE PROTEINS ; SNARE-like ; SNARE PROTEIN SEC22 ; V_SNARE ; LONGIN ; Synaptobrevin		K08517_04130_SNARE interactions in vesicular transport;	
MGC09394	1	634	MORN repeat-containing protein 2	MORN ; PHOSPHATIDYLINOSITOL-4-PHOSPHATE 5-KINASE-RELATED ; PHOSPHATIDYLINOSITOL-4-PHOSPHATE 5-KINASE RELATED ; Histone H3 K4-specific methyltransferase SET7/9 N-terminal domain		K00889_00562_Inositol phosphate metabolism; K00889_04070_Phosphatidylinositol signaling system; K00889_04810_Regulation of actin cytoskeleton;	2.7.1.68_1-phosphatidylinositol-4-phosphate5-kinase.;
MGC09401	1	755	Inactive phospholipase C-like protein 2	PI-PLC-Y ; PLC-like phosphodiesterases ; PIPLC_Y_DOMAIN ; PHPHLIPASEC ; Q15111_HUMAN_Q15111; ; C2DOMAIN ; PHOSPHOLIPASE C ; PHOSPHOLIPASE C-LIKE PROTEIN 2, PLC-L2 ; C2 domain (Calcium/lipid-binding domain, CaLB) ; C2	GO:0007242_P_intracellular signaling cascade; GO:0004629_F_phospholipase C activity;	K05857_00562_Inositol phosphate metabolism; K05857_04020_Calcium signaling pathway; K05857_04070_Phosphatidylinositol signaling system;	3.1.4.11_PhosphoinositidephospholipaseC.;
MGC09404	1	588	Complement C1q tumor necrosis factor-related protein 2	C1q ; CEREBELLIN-RELATED ; COMPLEMNTC1Q ; TNF-like ; C1Q			
MGC09406	2	774	Viral protein TPX	PRICHEXTENSN ; VWFC_2 ; VWFC_1			
MGC09408	1	691	Growth arrest-specific protein 8	coiled-coil	GO:0008285_P_negative regulation of cell proliferation; GO:0005874_C_microtubule; GO:0005515_F_protein binding; GO:0030317_P_sperm motility; GO:0009434_C_microtubule-based flagellum;		
MGC09413	1	695	HCLS1-binding protein 3	SORTING NEXIN ; SORTING NEXIN 7-RELATED ; PX ; PX domain	GO:0007166_P_cell surface receptor linked signal transduction; GO:0005515_F_protein binding; GO:0030217_P_T cell differentiation;		
MGC09415	1	699	Cingulin-like protein 1	coiled-coil			

MGC09416	1	621	Cholecystokinin receptor type A	G_PROTEIN_RECEP_F1_2 ; G-PROTEIN COUPLED RECEPTOR ; G_PROTEIN_RECEP_F1_1 ; GPCRRHODOPSN ; 7tm_1 ; Family A G protein-coupled receptor-like		K04205_04080_Neuroactive ligand-receptor interaction; K04238_04080_Neuroactive ligand-receptor interaction; K04195_04020_Calcium signaling pathway; K04195_04080_Neuroactive ligand-receptor interaction;	
MGC09419	1	747	Proteasome subunit alpha type-1	N-terminal nucleophile aminohydrolases (Ntn hydrolases) ; Proteasome ; PROTEASOME SUBUNIT ALPHA TYPE 1 ; PROTEASOME SUBUNIT ALPHA/BETA ; PROTEASOME_A		K02725_03050_Proteasome;	3.4.25.1_Proteasomeendopeptidasecomplex.;
MGC09429	1	601	Chondroitin sulfate N-acetylgalactosaminyltransferase 2	Nucleotide-diphospho-sugar transferases ; CHONDROITIN SYNTHASE ; CHGN		K00746_00532_Chondroitin sulfate biosynthesis; K00746_01030_tba; K03419_00532_Chondroitin sulfate biosynthesis; K03419_01030_tba;	2.4.1.174_acetylgalactosaminyltransferase.; 2.4.1.226_N-acetylgalactosaminyl-proteoglycan3-beta-glucuronosyltransferase.; 2.4.1.175_acetylgalactosaminyltransferase.;
MGC09430	1	796	E3 ubiquitin-protein ligase MIB2	ANKYRIN ; SKELETROPHIN ; Ank ; ANKYRIN REPEAT-CONTAINING ; ANK_REPEAT ; ANK_REP_REGION ; Ankyrin repeat			2.7.11.1_Non-specificserine/threonineproteinkinase.; 3.1.1.4_PhospholipaseA(2).;
MGC09434	1	443	Uncharacterized protein PF11_0207	coiled-coil			
MGC09435	1	610	E3 ubiquitin-protein ligase TRIM33	RING/U-box ; TRIM56 PROTEIN ; ZF_RING_1 ; zf-C3HC4 ; ZINC_FINGER_C2H2_1 ; ZF_RING_2 ; ZF_BBOX ; zf-B_box ; RING FINGER-CONTAINING PROTEIN-RELATED			
MGC09439	1	767	Early growth response protein 1	EARLY GROWTH RESPONSE PROTEIN ; Q18250_CAEEL_Q18250; ; C2H2 and C2HC zinc fingers ; zf-C2H2 ; ZINC_FINGER_C2H2_2			
MGC09444	1	746	Nebulin-related-anchoring protein	NEBULIN-RELATED ANCHORING PROTEIN ; LIM ; LIM_DOMAIN_2 ; Q8N3R6_HUMAN_Q8N3R6; ; Glucocorticoid receptor-like (DNA-binding domain) ; LIM_DOMAIN_1 ; coiled-coil ; NEBULIN			
MGC09446	1	601	E3 ubiquitin-protein ligase TRIM33	TRIM56 PROTEIN ; ZF_BBOX ; zf-B_box ; RING FINGER-CONTAINING PROTEIN-RELATED ; coiled-coil			
MGC09451	1	708	Transmembrane and TPR repeat-containing protein 2	TPR_1 ; TPR ; O-GLCNAC TRANSFERASE, P110 SUBUNIT-RELATED ; TPR_2 ; TPR-like ; TETRATRICOPEPTIDE REPEAT PROTEIN, TPR ; TPR_REGION	GO:0005783_C_endoplasmic reticulum;	K09667_00512_O-Glycan biosynthesis; K09667_01030_tba;	4.6.1.1_Adenylatecyclase.;
MGC09453	1	570	Heat shock protein 75 kDa, mitochondrial	HSP90 C-terminal domain (C-terminal part of Pfam 00183) ; TUMOR NECROSIS FACTOR TYPE 1 RECEPTOR ASSOCIATED PROTEIN (TRAP-1) ; HEAT SHOCK PROTEIN 90 ; Ribosomal protein S5 domain 2-like ; HSP90 ; coiled-coil	GO:0005164_F_tumor necrosis factor receptor binding;		
MGC09454	1	745	Ras suppressor protein 1	LRR_1 ; LEUCINE-RICH REPEAT-CONTAINING PROTEIN ; RAS SUPPRESSOR PROTEIN 1, RSU1 ; L domain-like ; LEURICHRPT	GO:0007165_P_signal transduction; GO:0005515_F_protein binding;	K01768_00230_Purine metabolism;	3.1.13.4_Poly(A)-specificribonuclease.;
MGC09463	1	738	Digestive cysteine proteinase 1	Cysteine proteinases ; Q6F6A8_DAUCA_Q6F6A8; ; Peptidase_C1 ; THIOL_PROTEASE_HIS ; CYSTEINE PROTEASE FAMILY C1-RELATED ; THIOL_PROTEASE_ASN		K01365_04612_Antigen processing and presentation;	3.4.22.15_CathepsinL.;

MGC09469	1	630	Collagen alpha-1(XII) chain	VWFA ; INTEGRIN ALPHA-RELATED ; VON WILENBRAND FACTOR RELATED ; vWA-like ; VWA			
MGC09475	1	710	Versican core protein	C-type lectin-like ; C_TYPE_LECTIN_1 ; C-TYPE LECTIN SUPERFAMILY MEMBER ; Lectin_C ; GALACTOSE-SPECIFIC C-TYPE LECTIN ; C_TYPE_LECTIN_2			
MGC09476	1	740	Lipopolysaccharide-binding protein	BACTERICIDAL PERMEABILITY-INCREASING (BPI) PROTEIN ; LBP_BPI_CETP ; Bactericidal permeability-increasing protein, BPI		K08761_03320_PPAR signaling pathway; K05399_04620_Toll-like receptor signaling pathway;	
MGC09479	1	723	Serine/threonine-protein phosphatase 6 regulatory ankyrin repeat subunit C	ANKYRIN ; Ank ; ANKYRIN REPEAT-CONTAINING ; ANK_REPEAT ; ANK_REP_REGION ; Ankyrin repeat		K08803_05219_Bladder cancer;	2.4.2.30_NAD(+)ADP-ribosyltransferase.; 2.7.11.1_Non-specificserine/threonineprotei nkinase.;
MGC09480	1	508		OVARIAN CARCINOMA IMMUNOREACTIVE ANTIGEN ; OCIA			
MGC09484	1	602	Collagen alpha-5(VI) chain	VWFA ; VON WILENBRAND FACTOR RELATED ; vWA-like ; VWA ; VWFADOMAIN			
MGC09508	1	685	Matrix metalloproteinase-19	Hemopexin ; Hemopexin-like domain ; MATRIX METALLOPROTEASE 15, MMP-15 ; HEMOPEXIN ; MATRIX METALLOPROTEINASE		K01398_04670_Leukocyte transendothelial migration; K01398_04912_GnRH signaling pathway; K01398_05219_Bladder cancer;	3.4.24.24_GelatinaseA.;
MGC09541	1	495	SCO-spondin	Serine proterase inhibitors ; VWFC_2 ; CONNECTIVE TISSUE GROWTH FACTOR-RELATED ; CONNECTIVE TISSUE GROWTH FACTOR/CCN2 ; VWFC_1	GO:0005737_C_cytoplasm;		
