# Supplementary material for: Insights into the innate immunity of the Mediterranean mussel Mytilus galloprovincialis
Source: BMC Genomics. 2011 Jan 26;12:69. doi: 10.1186/1471-2164-12-69 (PMC3039611; doi:10.1186/1471-2164-12-69)
Supplement: Additional file 2 — Differentially expressed genes in mussel hemocytes at 3 h post-injection of live V. splendidus. Probe ID, sequence information and ordered expression values (log2 of normalized test/control values) are reported. Similarities resulting from InterproScan Analysis are reported in brackets (* annotation based on manual inspection of other relevant similarities) [file 1471-2164-12-69-S2.PDF]

Additional file 2

Differentially expressed genes in mussel hemocytes at 3 h post-injection of live *V. splendidus:* probe ID, annotation, expression values.
(first-hit similarity to sequences expressed in the deep sea vent mussel *Bathymodiolus azoricus* have been added during the manuscript revision)

Mytibase ID	Sequence similarity description	Expression Value	Best sequence similarity of each Mytibase MGC to DeepSeaVent *B. azoricus* contigs BLASTN e-V	ID (B. azoricus)	First BlastX Hit Description (B. azoricus)
**Over-expressed**					
MGO_05083	WAS protein family member 3	2.88	1.00E-13	mussel_c27127	actin binding protein, putative [Aedes aegypti]
MGO_04578	Heavy metal-binding protein HIP	2.83	6.00E-12	mussel_c4891	sialic acid binding lectin [Cepaea hortensis]
MGO_07351	Heavy metal-binding protein HIP	2.50	3.00E-16	mussel_rep_c43238	PREDICTED: similar to FLJ00201 protein [Gallus gallus]
MGO_00991	Allograft inflammatory factor 1, AIF	2.45	1.00E-71	mussel_c48503	allograft inflammatory factor 1-like [Mus musculus]
MGO_09295	Baculoviral IAP repeat-containing protein 3, IAP	2.34	3.00E-41	mussel_rep_c39920	baculoviral IAP repeat-containing 3 [Mus musculus]
MGO_05648	Plasminogen	2.30	1.00E-07	mussel_rep_c55483	PREDICTED: similar to plasminogen isoform 2 [Monodelphis domestica]
MGO_07503	Fucolectin-6	2.29	2.00E-04	mussel_rep_c56716	No hits
MGO_03385	Integrin beta-1-B	2.28	5.00E-12	mussel_c30926	integrin, beta 7 [Bos taurus]
MGO_04450	ETS homologous factor	2.22	5.00E-135	mussel_c250	ETS-family transcription factor [Chlamys farreri]
MGO_06141	Unknown	2.21	4.00E-43	mussel_c24611	No hits
MGO_05290	RING finger protein ETP1	2.09	-	No hits found	
MGO_03934	NF-kappa-B inhibitor alpha, IkBα	2.03	0.00E+00	mussel_c244	inhibitor of nuclear factor-kappaB protein [Pinctada fucata]
MGO_01658	Collagen alpha-1(XIV) chain	1.94	3.00E-03	mussel_c6461	PREDICTED: similar to mCG140660 [Danio rerio]
MGO_00161	Apolipophorin	1.90	3.00E-76	mussel_c27498	RecName: Full=Apolipophorin-2;
MGO_03879	Tribbles homolog 1	1.86	1.00E-87	mussel_c4187	PREDICTED: similar to TRB2 protein [Ornithorhynchus anatinus]
MGO_00425	Major egg antigen (sHSP20)	1.86	4.00E-22	mussel_c56049	No hits
MGO_00233	Alpha-protein kinase vwkA	1.80	7.00E-76	mussel_c1110	predicted protein [Nematostella vectensis]
MGO_04287	Noggin-2	1.80	-	No hits found	
MGO_05614	Transcription factor p65	1.80	-	No hits found	
MGO_04110	Superoxide dismutase [Cu-Zn], SOD	1.75	2.00E-58	mussel_c1241	superoxide dismutase [Cu-Zn] [Megathura crenulata]
MGO_00609	Low affinity immunoglobulin epsilon Fc receptor (*mannose receptor 1, MR1)	1.68	-	No hits found	
MGO_09439	Early growth response protein 1	1.62	1.00E-155	mussel_c25665	conserved hypothetical protein [Culex quinquefasciatus]
MGO_00060	Chitotriosidase-1	1.62	2.00E-18	mussel_c52715	Chain A, Structure Of Human Chitotriosidase
MGO_04039	CCAAT/enhancer-binding protein epsilon	1.61	3.00E-99	mussel_rep_c23380	CCAAT/enhancer binding protein [Aplysia kurodai]
MGO_07770	Myeloid differentiation primary response protein MyD88	1.60	5.00E-83	mussel_c1511	MyD88 adaptor [Crassostrea gigas]
MGO_07312	MAP kinase-interacting serine/threonine-protein kinase 1, MNK	1.58	6.00E-39	mussel_c46250	RecName: Full=MAP kinase-interacting serine/threonine-protein kinase 1;
MGO_06796	Baculoviral IAP repeat-containing protein 7-A, IAP	1.57	1.00E-03	mussel_c5979	hypothetical protein BRAFLDRAFT_224204 [Branchiostoma floridae]
MGO_06631	Unknown	1.57	-	No hits found	
MGO_08195	Centromere protein F	1.56	-	No hits found	
MGO_06401	Unknown	1.54	2.00E-06	mussel_c8742	Complement C1q-like protein 2 [Salmo salar]
MGO_06772	Caprin-2	1.52	-	No hits found	
MGO_08250	Endoplasmic reticulum-Golgi intermediate compartment protein 2	1.50	9.00E-55	mussel_c9316	PREDICTED: similar to MGC81917 protein [Strongylocentrotus purpuratus]
MGO_01777	G-protein coupled receptor GRL101	1.50	-	No hits found	
MGO_09361	Perlucin	1.50	-	No hits found	
MGO_07491	Aggrecan core protein	1.48	3.00E-03	mussel_c34984	No hits
MGO_03478	Ependymin-2	1.47	5.00E-03	mussel_c4370	hypothetical protein BRAFLDRAFT_127102[Branchiostoma floridae]
MGO_08186	Tumor necrosis factor receptor superfamily member 27, TNFR	1.42	-	No hits found	
MGO_00580	Transmembrane protein 205	1.42	6.00E-19	mussel_c41923	No hits
MGO_09404	Complement C1q tumor necrosis factor-related protein 2	1.41	4.00E-27	mussel_c18364	PREDICTED: similar to complement component 1, q subcomponent-like 2 [Danio rerio]
MGO_06398	Ankyrin repeat domain-containing protein 46	1.38	-	No hits found	
MGO_07746	Unknown (C-type lectin-like)	1.37	-	No hits found	
MGO_07369	Macrophage asialoglycoprotein-binding protein 1	1.36	4.00E-15	mussel_c17100	C-type lectin [Venerupis philippinarum]
MGO_07998	Adhesive plaque matrix protein 2	1.36	-	No hits found	
MGO_01089	Complement C1q tumor necrosis factor-related protein 3	1.35	1.00E-27	mussel_c2254	sialic acid binding lectin [Helix pomatia]
MGO_03696	Macrophage asialoglycoprotein-binding protein 1	1.34	1.00E-07	mussel_rep_c59520	C-type lectin, superfamily member 9 [Mus musculus]
MGO_02087	Myosin heavy chain, striated muscle	1.34	7.00E-140	mussel_c31887	myosin heavy chain [Mytilus galloprovincialis]
MGO_07609	Complement C1q tumor necrosis factor-related protein 8	1.34	2.00E-06	mussel_rep_c33855	sialic acid binding lectin [Haliotis discus discus]
MGO_07298	Heavy metal-binding protein HIP	1.33	-	No hits found	
MGO_00857	Suppressor of cytokine signaling 2	1.29	5.00E-74	mussel_c2213	suppressor of cytokine signaling 2 [Haliotis discus discus]
MGO_07835	Uncharacterized transmembrane protein DDB_G0289901	1.27	-	No hits found	
MGO_02425	Chitotriosidase-1	1.26	1.00E-16	mussel_c27937	midgut chitinase [Phlebotomus papatasi]
MGO_01735	Zinc metalloproteinase nas-13	1.25	-	No hits found	
MGO_01612	CD209 antigen-like protein E	1.25	-	No hits found	
MGO_00125	Fibrinogen C domain-containing protein 1	1.25	2.00E-16	mussel_c31141	hypothetical protein BRAFLDRAFT_59661 [Branchiostoma floridae]
MGO_05528	Ras association domain-containing protein 1	1.25	-	No hits found	
MGO_08052	C-type lectin domain family 4 member E	1.25	-	No hits found	
MGO_06909	WASH complex subunit CCDC53	1.24	3.00E-42	mussel_c2651	Coiled-coil domain-containing protein 53 [Salmo salar]
MGO_04809	Techylectin-5B	1.23	5.00E-24	mussel_c755	hypothetical protein BRAFLDRAFT_86061 [Branchiostoma floridae]
MGO_07535	Protein toll	1.22	9.00E-42	mussel_c39831	Toll receptor [Chlamys farreri]
MGO_07536	Low affinity immunoglobulin epsilon Fc receptor	1.22	1.00E-08	mussel_c12335	perlucin-like protein isoform A [Venerupis philippinarum]
MGO_04997	Dedicator of cytokinesis protein 7	1.22	-	No hits found	
MGO_04922	Ribosomal protein S6 kinase beta-1	1.21	-	No hits found	
MGO_01319	Actin, adductor muscle	1.21	0.00E+00	mussel_c325	RecName: Full=Actin, adductor muscle
MGO_06800	Type-1B angiotensin II receptor	1.20	-	No hits found	
MGO_01787	Unknown	1.19	-	No hits found	
MGO_03790	Ras-related protein Rab-32B	1.19	3.00E-30	mussel_rep_c40648	No hits
MGO_04063	Yolk ferritin	1.18	2.00E-89	mussel_c24591	RecName: Full=Yolk ferritin;
MGO_02371	Unknown (*hsc70 gene for heat shock cognate 70)	1.17	-	No hits found	
MGO_07274	Uncharacterized protein sll1388, (Adenine nucleotide alpha hydrolases-like ; Usp)	1.17	5.00E-52	mussel_c1664	predicted protein [Nematostella vectensis]
MGO_07926	N,N'-diacetylchitobiase	1.17	-	No hits found	
MGO_00278	Probable G-protein coupled receptor 83	1.17	1.00E-37	mussel_rep_c31932	GK13356 [Drosophila willistoni]
MGO_06262	Transcription intermediary factor 1-beta	1.16	-	No hits found	
MGO_00924	Delta and Notch-like epidermal growth factor-related receptor	1.16	-	No hits found	
MGO_07828	CAP-Gly domain-containing linker protein 3	1.16	-	No hits found	
MGO_06364	78 kDa glucose-regulated protein, GRP78	1.15	6.00E-126	mussel_c4463	78kDa glucose regulated protein [Crassostrea gigas]
MGO_04137	Thyrotropin-releasing hormone-degrading ectoenzyme	1.15	4.00E-03	mussel_c16473	No hits
MGO_04603	Endoplasmin, GRP94	1.15	1.00E-111	mussel_c10957	glucose-regulated protein 94 [Crassostrea gigas]
MGO_00488	WD repeat-containing protein 89	1.12	1.00E-179	mussel_c5230	PREDICTED: hypothetical protein [Strongylocentrotus purpuratus]
MGO_04135	Src substrate cortactin	1.10	4.00E-112	mussel_c55154	Cttn protein [Xenopus laevis]
MGO_04498	B(0,+)-type amino acid transporter 1	1.10	3.00E-78	mussel_rep_c27056	hypothetical protein TcasGA2_TC000583 [Tribolium castaneum]
MGO_07376	Translation initiation factor IF-2	1.09	1.00E-84	mussel_c3344	PREDICTED: similar to Nuclear receptor coactivator 5 [Strongylocentrotus purpuratus]
MGO_07691	C-type lectin domain family 4 member E	1.08	6.00E-11	mussel_c55188	codakine [Codakia orbicularis]
MGO_04209	Peptidoglycan recognition protein 1, PGRP	1.07	7.00E-81	mussel_c110	peptidoglycan recognition protein 2 precursor [Euprymna scolopes]
MGO_01452	Very low-density lipoprotein receptor, LDLR	1.06	5.00E-18	mussel_c13003	PREDICTED: similar to low density lipoprotein receptor-related protein 8 [Canis familiaris]
MGO_01376	Hepatic lectin (*Macrophage mannose receptor 1 precursor, MR1)	1.05	-	No hits found	
MGO_06198	Tetraspanin-7/ CD63 antigen	1.05	-	No hits found	
MGO_05318	Kinase D-interacting substrate of 220 kDa	1.05	-	No hits found	
MGO_01716	Slit homolog 3 protein	1.04	-	No hits found	
MGO_05135	Unknown	1.04	-	No hits found	
MGO_03472	Unknown	1.02	-	No hits found	
MGO_02711	Annulin	1.01	1.00E-18	mussel_c32988	protein-glutamine gamma-glutamyltransferase, putative [Ixodes scapularis]
MGO_07370	Alpha-2-macroglobulin-P	1.01	-	No hits found	
MGO_04820	Papilin	0.96	3.00E-37	mussel_c51088	No hits
MGO_04645	ATP-dependent RNA helicase DDX54	0.96	2.00E-69	mussel_c32700	hypothetical protein LOC495097 [Xenopus laevis]
MGO_07516	Unknown	0.93	4.00E-15	mussel_c55578	No hits
MGO_08064	Fibrinogen C domain-containing protein 1	0.93	2.00E-17	mussel_c31141	hypothetical protein BRAFLDRAFT_59661 [Branchiostoma floridae]
MGO_01455	Elongation factor 2	0.92	0.00E+00	mussel_c657	elongation factor-2 [Chaetopleura apiculata]
MGO_00659	Mytilin-B	0.92	-	No hits found	
MGO_00477	Interleukin-1 receptor-associated kinase 4, IRAK4	0.88	4.00E-06	mussel_c16476	No hits
MGO_01800	Optineurin	0.88	-	No hits found	
MGO_00400	Protein fosB	0.86	5.00E-127	mussel_c7288	protein fosB, putative [Ixodes scapularis]
MGO_06702	Ubiquitin	0.85	1.00E-67	mussel_c10233	polyubiquitin [Homo sapiens]
MGO_09415	Cingulin-like protein 1	0.85	2.00E-17	mussel_c36380	hypothetical protein jhp0050 [Helicobacter pylori J99]
MGO_08200	Receptor-type tyrosine-protein phosphatase U	0.84	6.00E-12	mussel_c4654	predicted protein [Nematostella vectensis]
MGO_09348	Kelch-like protein 13, KLHL	0.84	0.00E+00	mussel_c477	KLHL9 protein [Homo sapiens]
MGO_00786	Unknown (C-type lectin-like)	0.83	-	No hits found	
**Under-expressed**					
MGO_01219	Ubiquitin	-0.95	4.00E-119	mussel_rep_c23840	ubiquitin [Branchiostoma belcheri]
MGO_01145	ATP synthase-coupling factor 6, mitochondrial	-1.02	-	No hits found	
MGO_07586	26S proteasome complex subunit DSS1	-1.02	9.00E-69	mussel_rep_c70771	26S proteasome complex subunit DSS1, putative [Ixodes scapularis]
MGO_07799	Thyroglobulin	-1.03	-	No hits found	
MGO_00934	Fibrinogen C domain-containing protein 1	-1.04	1.00E-14	mussel_c4573	hypothetical protein BRAFLDRAFT_108089 [Branchiostoma floridae]
MGO_08561	Sulfated surface glycoprotein 185	-1.11	4.00E-07	mussel_c52964	Hypothetical protein CBG22657 [Caenorhabditis briggsae]
MGO_04517	Complement C1q tumor necrosis factor-related protein 3	-1.11	-	No hits found	
MGO_09294	BRCA1-associated RING domain protein 1	-1.12	3.00E-52	mussel_rep_c26319	ank repeat-containing [Schistosoma mansoni]
MGO_00413	Unknown	-1.15	-	No hits found	
MGO_04466	Prostaglandin E2 receptor EP4 subtype	-1.16	-	No hits found	
MGO_00779	WSC domain-containing protein 2	-1.21	-	No hits found	
MGO_05362	Uncharacterized protein PF11_0207 (coliled coil)	-1.23	-	No hits found	
MGO_00270	Myticin-A	-1.26	-	No hits found	
MGO_09090	Casein kinase II subunit beta	-1.29	-	No hits found	
MGO_03656	Ankyrin repeat domain-containing protein 49	-1.29	-	No hits found	
MGO_03559	Macrophage migration inhibitory factor, MIF	-1.29	2.00E-63	mussel_c10585	macrophage migration inhibitory factor [Ascaris suum]
MGO_00508	Complement C1q-like protein 3	-1.35	2.00E-11	mussel_rep_c67050	PREDICTED: similar to Collagen alpha-1(VIII) chain [Monodelphis domestica]
MGO_04267	Ficolin-2	-1.42	2.00E-12	mussel_c10289	hypothetical protein BRAFLDRAFT_86061 [Branchiostoma floridae]
MGO_00501	Integumentary mucin C.1	-1.43	-	No hits found	
MGO_00305	Complement C1q tumor necrosis factor-related protein 3	-1.44	-	No hits found	
MGO_03550	Proteasome assembly chaperone 2, PAC	-1.44	1.00E-148	mussel_c574	hypothetical protein BRAFLDRAFT_118820 [Branchiostoma floridae]
MGO_01080	Unknown	-1.52	-	No hits found	
MGO_04881	Complement C1q tumor necrosis factor-related protein 3	-1.57	-	No hits found	
MGO_02476	Stress-associated endoplasmic reticulum protein 2	-1.58	8.00E-78	mussel_c2242	predicted protein [Nematostella vectensis]
MGO_09001	Neuronal calcium sensor 2	-1.60	8.00E-78	mussel_c2242	predicted protein [Nematostella vectensis]

MGO_03647	Unknown (*MAC/perforin- and kringle-domains-containing protein)	-1.61	-	No hits found	
MGO_08987	Golgi-associated plant pathogenesis-related protein 1	-1.63	-	No hits found	
MGO_00517	Heavy metal-binding protein HIP	-1.66	-	No hits found	
MGO_06436	Neurotrypsin (scavenger receptor cysteine-rich protein precursor)	-1.69	8.00E-04	mussel_c59462	No hits
MGO_00791	WSC domain-containing protein 2	-1.82	-	No hits found	
MGO_01925	60S ribosomal protein L21	-1.91	-	No hits found	
MGO_00333	Collagen alpha-2(VIII) chain	-1.92	2.00E-11	mussel_c7198	unnamed protein product [Tetraodon nigroviridis]
MGO_00451	Defensin MGD-1	-2.24	-	No hits found	
MGO_00292	Unknown (*apextrin)	-2.43	1.00E-03	mussel_rep_c34433	No hits
MGO_00845	Unknown (*apextrin)	-2.56	-	No hits found	
MGO_06446	Short-chain collagen C4	-2.79	3.00E-47	mussel_c5763	hypothetical protein BRAFLDRAFT_80549 [Branchiostoma floridae]
MGO_06572	C3 and PZP-like alpha-2-macroglobulin domain-containing protein 8	-3.15	9.00E-28	mussel_c4665	thioester-containing protein [Chlamys farreri]

No hits found: no counterpart found in the DeepSeaVent *Bathymodiolus azoricus* database (e-value ≥ 0.001, BLASTN 2.2.23+)
