# Supplementary material for: Insights into the innate immunity of the Mediterranean mussel Mytilus galloprovincialis
Source: BMC Genomics. 2011 Jan 26;12:69. doi: 10.1186/1471-2164-12-69 (PMC3039611; doi:10.1186/1471-2164-12-69)
Supplement: Additional file 3 — Differentially expressed genes in mussel hemocytes at 48 h post-injection of live V. splendidus. Probe ID, sequence information and ordered expression values (log2 of normalized test/control values) are reported. Similarities resulting from InterproScan Analysis are reported in brackets (* annotation based on manual inspection of other relevant similarities) [file 1471-2164-12-69-S3.PDF]

Additional file 3
Differentially expressed genes in mussel hemocytes at 48 h post-injection of live *V. splendidus:* probe ID, annotation, expression values.
(first-hit similarity to sequences expressed in the deep sea vent mussel Bathymodiolus azoricus have been added during the manuscript revision)

**Mytibase ID**	**Sequence similarity description**	**Expression Value**	**Sequence similarity to *B. azoricus* transcript contigs (**454 GS-FLX reads)		
			**BLASTN e-V**	**ID (B. azoricus)**	**First BlastX Hit Description**
**Over-expressed**					
MGO_03478	Ependymin-2	2.93	5.00E-03	mussel_c4370	hypothetical protein BRAFLDRAFT_127102[Branchiostoma floridae]
MGO_00233	Alpha-protein kinase vwkA	2.82	7.00E-76	mussel_c1110	predicted protein [Nematostella vectensis]
MGO_05648	Plasminogen	2.45	1.00E-07	mussel_rep_c55483	PREDICTED: similar to plasminogen isoform 2 [Monodelphis domestica]
MGO_05118	Integumentary mucin C.1	2.44	7.00E-115	mussel_c2786	PREDICTED: similar to GH24606 [Ciona intestinalis]
MGO_03983	Peptidyl-prolyl cis-trans isomerase B	2.38	0.00E+00	mussel_c816	PREDICTED: similar to peptidylprolyl isomerase (EC 5.2.1.8) B, 20.3K - rat [Strongylocentrotus purpura
MGO_04110	Superoxide dismutase [Cu-Zn], SOD	2.29	2.00E-58	mussel_c1241	superoxide dismutase [Cu-Zn] [Megathura crenulata]
MGO_00991	Allograft inflammatory factor, AIF	2.29	1.00E-71	mussel_c48503	allograft inflammatory factor 1-like [Mus musculus]
MGO_00161	Apolipophorin	2.22	3.00E-76	mussel_c27498	RecName: Full=Apolipophorin-2;
MGO_07158	Putative fungistatic metabolite	1.99	-	No hits found	
MGO_07604	Bactericidal permeability-increasing protein	1.97	-	No hits found	
MGO_05436	Lipopolysaccharide-induced tumor necrosis factor-alpha factor homolog, LITAF	1.96	5.00E-08	mussel_rep_c23813	LPS-induced TNF-alpha factor [Chlamys farreri]
MGO_07055	Calmodulin	1.91	8.00E-60	mussel_c7250	RecName: Full=Calmodulin;
MGO_04373	Actin cytoskeleton-regulatory complex protein pan1	1.90	3.00E-66	mussel_c24741	No hits
MGO_02668	Unknown (Immunoglobulin like)	1.88	-	No hits found	
MGO_03168	14-3-3-like protein 2	1.87	1.00E-87	mussel_c191	RecName: Full=14-3-3 protein 2
MGO_05779	Unknown (PROKAR lipoprotein)	1.86	-	No hits found	
MGO_02854	Suppressor of tumorigenicity 14 protein (Serine protease inhibitors, LDL receptor-like module)	1.84	-	No hits found	
MGO_04450	ETS homologous factor	1.83	5.00E-135	mussel_c250	ETS-family transcription factor [Chlamys farreri]
MGO_01337	Heavy metal-binding protein HIP	1.82	-	No hits found	
MGO_04197	Unknown (TONB dependent REC1)	1.82	-	No hits found	
MGO_00580	Transmembrane protein 205	1.80	6.00E-19	mussel_c41923	No hits
MGO_05711	Heat shock protein HSP 90-beta	1.80	9.00E-85	mussel_rep_c23505	heat shock protein 90 [Mytilus alloprovincialis]
MGO_09508	Matrix metalloproteinase-19	1.78	1.00E-38	mussel_c25956	GG19900 [Drosophila erecta]
MGO_00317	Mytilin-D	1.74	-	No hits found	
MGO_07770	Myeloid differentiation primary response protein MyD88	1.73	5.00E-83	mussel_c1511	MyD88 adaptor [Crassostrea gigas]
MGO_02500	Whey acidic protein	1.72	-	No hits found	
MGO_04498	B(0,+)-type amino acid transporter 1	1.71	3.00E-78	mussel_rep_c27056	hypothetical protein TcasGA2_TC000583 [Tribolium castaneum]
MGO_06383	Fucolectin-6	1.66	-	No hits found	
MGO_05528	Ras association domain-containing protein 1	1.61	-	No hits found	
MGO_04146	Calmodulin	1.58	1.00E-146	mussel_rep_c66559	RecName: Full=Calmodulin;
MGO_04578	Heavy metal-binding protein HIP	1.57	6.00E-12	mussel_c4891	sialic acid binding lectin [Cepaea hortensis]
MGO_00258	LPS-induced TNF-alpha factor, LITAF	1.56	4.00E-52	mussel_c12008	LPS-induced TNF-alpha factor [Chlamys farreri]
MGO_03878	Putative tyrosinase-like protein tyr-3	1.53	-	No hits found	
MGO_08860	Solute carrier family 25 member 38-B	1.50	3.00E-105	mussel_c28372	PREDICTED: hypothetical protein [Taeniopygia guttata]
MGO_04809	Techylectin-5B	1.49	5.00E-24	mussel_c755	hypothetical protein BRAFLDRAFT_86061 [Branchiostoma floridae]
MGO_06401	Unknown	1.49	2.00E-06	mussel_c8742	Complement C1q-like protein 2 [Salmo salar]
MGO_00630	Astacin	1.46	-	No hits found	
MGO_00298	Heat shock 70 kDa protein, HSP70	1.44	6.00E-72	mussel_c38295	heat shock protein 70 [Mytilus galloprovincialis]
MGO_01031	Unknown (C-type lectin-like)	1.44	3.00E-17	mussel_c26036	RecName: Full=Tetranectin-like protein
MGO_03838	Metalloproteinase inhibitor 3, TIMP3	1.44	-	No hits found	
MGO_00924	Delta and Notch-like epidermal growth factor-related receptor	1.43	-	No hits found	
MGO_04209	Peptidoglycan recognition protein 1, PGRP	1.43	7.00E-81	mussel_c110	peptidoglycan recognition protein 2 precursor [Euprymna scolopes]
MGO_05083	WAS protein family member 3	1.42	1.00E-13	mussel_c27127	actin binding protein, putative [Aedes aegypti]
MGO_01385	SCO-spondin	1.40	4.00E-88	mussel_c751	SCO-spondin precursor [Gallus gallus]
MGO_06198	Tetraspanin-7/ CD63 antigen	1.34	-	No hits found	
MGO_03765	Calmodulin	1.33	3.00E-142	mussel_rep_c23919	PREDICTED: similar to calmodulin 2 [Strongylocentrotus purpuratus]
MGO_02825	Macrophage mannose receptor 1, MR1	1.33	1.00E-20	mussel_c1525	hypothetical protein BRAFLDRAFT_224211 [Branchiostoma floridae]
MGO_05498	Unknown (TNF-like)	1.31	-	No hits found	
MGO_02456	Cathepsin L1	1.30	0.00E+00	mussel_c3973	predicted protein [Nematostella vectensis]
MGO_05735	Unknown (C1Q, TNF-like)	1.30	-	No hits found	
MGO_00425	Major egg antigen (sHSP20)	1.29	4.00E-22	mussel_c56049	No hits
MGO_00516	Galectin-9	1.28	1.00E-85	mussel_c149	galectin [Pinctada fucata]
MGO_02559	Collagen alpha-1(III) chain	1.28	4.00E-149	mussel_c1150	large adhesin [Haemophilus somnus 129PT]
MGO_05285	Perlucin	1.28	-	No hits found	
MGO_01452	Very low-density lipoprotein receptor, LDLR	1.26	5.00E-18	mussel_c13003	PREDICTED: similar to low density lipoprotein receptor-related protein 8 [Canis familiaris]
MGO_02833	Heavy metal-binding protein HIP	1.25	-	No hits found	
MGO_03857	Unknown	1.24	3.00E-49	mussel_c964	No hits
MGO_05076	Calnexin	1.23	3.00E-21	mussel_c494	calreticulin [Crassostrea gigas]
MGO_00250	Caprin-2	1.22	-	No hits found	
MGO_04608	Collagen alpha-1(XII) chain	1.20	-	No hits found	
MGO_07567	Caspase-2	1.18	-	No hits found	
MGO_06800	Type-1B angiotensin II receptor	1.17	-	No hits found	
MGO_05112	Complement C1q-like protein 4	1.17	3.00E-66	mussel_c585	gliacolin-related [Schistosoma mansoni]
MGO_04478	Unknown (Chitin-bind3)	1.16	7.00E-58	mussel_c41606	conserved hypothetical protein [Ixodes scapularis]
MGO_00222	Collagen alpha-2(XI) chain	1.16	1.00E-168	mussel_c1150	large adhesin [Haemophilus somnus 129PT]
MGO_05132	Unknown (C-type lectin-like)	1.16	-	No hits found	
MGO_03800	Unknown (TNF-like)	1.14	-	No hits found	
MGO_05242	Cathepsin C	1.14	0.00E+00	mussel_c146	PREDICTED: similar to cathepsin C [Strongylocentrotus purpuratus]
MGO_02412	Cysteine-rich motor neuron 1 protein	1.11	-	No hits found	
MGO_05594	Radial spoke head 1 homolog	1.11	0.00E+00	mussel_c1881	hypothetical protein LOC100177304 [Ciona intestinalis]
MGO_04049	Metalloproteinase inhibitor 3, TIMP3	1.10	-	No hits found	
MGO_00786	Unknown (C-type lectin-like)	1.10	-	No hits found	
MGO_02744	Proteasome subunit alpha type-2	1.07	1.00E-101	mussel_c42067	proteasome alpha type 2 [Haliotis discus discus]
MGO_07056	Unknown (C-type lectin-like)	1.06	-	No hits found	
MGO_01319	Actin, adductor muscle	1.06	0.00E+00	mussel_c325	RecName: Full=Actin, adductor muscle
MGO_01042	Allene oxide synthase-lipoxygenase protein	1.06	-	No hits found	
MGO_07459	Unknown (*bactericidal permeability increasing protein LBP/BPI)	1.04	-	No hits found	
MGO_03064	Cerebellin-2	1.03	-	No hits found	
MGO_04275	Unknown (DEATH domain)	1.03	-	No hits found	
MGO_00636	Unknown (MACPF; MAC-perforin)	1.02	-	No hits found	
MGO_01771	Butyrate response factor 2	1.01	2.00E-72	mussel_c484	Tis11 family protein [Crassostrea virginica]
MGO_09243	Unknown (Toll/Interleukin receptor TIR domain)	1.00	-	No hits found	
MGO_09162	Fibrinogen-like protein 1	0.99	1.00E-07	mussel_c1346	hypothetical protein BRAFLDRAFT_86061 [Branchiostoma floridae]
MGO_07746	Unknown (C-type lectin-like)	0.98	-	No hits found	
MGO_09408	Growth arrest-specific protein 8	0.98	6.00E-151	mussel_c32059	predicted protein [Nematostella vectensis]
MGO_00616	Calreticulin	0.98	8.00E-170	mussel_c494	calreticulin [Crassostrea gigas]
MGO_07865	Baculoviral IAP repeat-containing protein 7, IAP	0.98	2.00E-09	mussel_c26844	baculoviral IAP repeat-containing 7 [Xenopus (Silurana) tropicalis]
MGO_00545	Angiopoietin-related protein 6	0.98	2.00E-89	mussel_c215	PREDICTED: similar to angiopoietin-like 2 a [Ciona intestinalis]
MGO_07566	T-complex protein 1 subunit delta	0.96	6.00E-06	mussel_c26932	hypothetical protein BRAFLDRAFT_279056 [Branchiostoma floridae]
MGO_08044	Equistatin	0.96	-	No hits found	
MGO_05603	Elongation factor 1-alpha	0.96	6.00E-138	mussel_c912	elongation factor 1 alpha [Mytilus galloprovincialis]
MGO_02087	Myosin heavy chain, striated muscle	0.95	7.00E-140	mussel_c31887	myosin heavy chain [Mytilus galloprovincialis]
MGO_06058	Ficolin-2	0.94	4.00E-52	mussel_rep_c32176	hypothetical protein BRAFLDRAFT_88596 [Branchiostoma floridae]
MGO_06855	TFIIH basal transcription factor complex helicase subunit	0.93	2.00E-71	mussel_c27596	hypothetical protein BRAFLDRAFT_286830 [Branchiostoma floridae]
MGO_00748	Brevican core protein	0.92	-	No hits found	
MGO_05292	Proteasome subunit beta type-4	0.91	1.00E-148	mussel_rep_c23683	proteasome subunit N3 [Haliotis discus discus]
MGO_08207	Protein jagged-1 (EGF/Laminin)	0.90	-	No hits found	
MGO_04926	Unknown	0.89	-	No hits found	
MGO_05290	RING finger protein ETP1	0.89	-	No hits found	
MGO_08762	Myosin-XV	0.89	-	No hits found	
MGO_04451	Collagen alpha-1(XII) chain	0.88	-	No hits found	
MGO_04159	Endoplasmin GRP94	0.88	0.00E+00	mussel_c568	glucose-regulated protein 94 [Crassostrea gigas]
MGO_01476	Collagen alpha-1(I) chain	0.87	6.00E-52	mussel_rep_c23498	PREDICTED: similar to alpha 1 type I collagen preproprotein isoform 2 [Monodelphis domestica]
MGO_07794	von Willebrand factor C domain-containing protein 2-like	0.87	-	No hits found	
MGO_04603	Endoplasmin GRP94	0.87	1.00E-111	mussel_c10957	glucose-regulated protein 94 [Crassostrea gigas]
MGO_07284	Unknown (G protein-coupled receptor-like)	0.86	3.00E-47	mussel_rep_c32803	hypothetical protein BRAFLDRAFT_77391 [Branchiostoma floridae]
MGO_02462	Uncharacterized protein DDB_G0271670	0.86	-	No hits found	
MGO_00225	Lipopolysaccharide-induced tumor necrosis factor-alpha factor homolog, LITAF	0.85	6.00E-57	mussel_c588	lipopolysaccharide-induced TNF-alpha factor [Meretrix meretrix]
MGO_03900	Muscle LIM protein Mlp84B	0.84	2.00E-126	mussel_c25842	LIM protein [Mytilus edulis]
MGO_05057	Probable inactive serine/threonine-protein kinase slob2	0.83	-	No hits found	
MGO_03647	Unknown (*MAC/perforin- and kringle-domains-containing protein)	0.83	-	No hits found	
MGO_03812	Cerebellin-2	0.83	-	No hits found	
MGO_06304	Mitochondrial import inner membrane translocase subunit Tim16	0.80	1.00E-46	mussel_c233	mitochondria-associated granulocyte macrophage CSF signaling molecule [Bombyx mori]
MGO_03231	Unknown	0.80	-	No hits found	
MGO_02060	Uncharacterized protein yxiE (Adenine nucleotide alpha hydrolases-like ; Usp)	0.79	6.00E-126	mussel_c94	predicted protein [Nematostella vectensis]
MGO_07116	Cathepsin D	0.78	1.00E-47	mussel_rep_c42203	cathepsin D [Chlamys farreri]
MGO_00554	Fucolectin-2	0.78	8.00E-04	mussel_c35505	No hits
MGO_00848	Proteasome subunit beta type-5	0.78	0.00E+00	mussel_c58	unknown [Branchiostoma floridae]
MGO_08549	Death-associated protein 1	0.76	5.00E-69	mussel_c485	death-associated protein [Crassostrea angulata]
MGO_03521	Uncharacterized protein ZC395.10 (HSP90co-chaperone)	0.75	2.00E-147	mussel_c7086	PREDICTED: similar to CG16817-PA [Apis mellifera]
MGO_02457	PDZ and LIM domain protein 4	0.74	5.00E-07	mussel_c32335	No hits
MGO_03551	von Willebrand factor A domain-containing protein 2	0.73	-	No hits found	
MGO_01586	Guanine nucleotide-binding protein subunit beta-2-like 1	0.71	0.00E+00	mussel_rep_c27146	Receptor of Activated Kinase C 1 [Mya arenaria]
MGO_03602	Gelsolin-like protein 1	0.69	3.00E-70	mussel_c9949	gelsolin [Suberites ficus]
**Under-expressed**					
MGO_00475	Unknown (C1q ; TNF-like)	-0.67	7.00E-12	mussel_c914	No hits
MGO_07620	Differentially expressed in FDCP 6 homolog	-0.71	-	No hits found	
MGO_01646	Nidogen-1	-0.71	4.00E-72	mussel_rep_c23375	AGAP005942-PB [Anopheles gambiae str. PEST]
MGO_01070	Myticin-A	-0.72	-	No hits found	
MGO_00501	Integumentary mucin C.1	-0.73	-	No hits found	
MGO_00685	Cell division control protein 42 homolog	-0.74	4.00E-40	mussel_c37505	Cell division control protein 42 homolog [Oncorhynchus mykiss]
MGO_00336	Unknown	-0.75	1.00E-09	mussel_c33691	hypothetical protein BRAFLDRAFT_92086 [Branchiostoma floridae]

MGO_03997	Disabled homolog 2-interacting protein	-0.77	1.00E-108	mussel_c33621	PREDICTED: similar to synaptic ras gtpase activating protein, syngap [Acyrthosiphon pisum]
MGO_07792	Myosin regulatory light chain B, smooth adductor muscle	-0.77	4.00E-78	mussel_c275	myosin:SUBUNIT=regulatory light chain
MGO_01021	Myticin-B	-0.77	-	No hits found	
MGO_02733	Unknown (HSP20-like chaperones)	-0.77	1.00E-32	mussel_rep_c23478	PREDICTED: similar to small heat shock protein [Hydra magnipapillata]
MGO_04088	SH3 domain-binding glutamic acid-rich-like protein 3	-0.77	4.00E-13	mussel_c26761	No hits
MGO_03972	Gastric intrinsic factor	-0.78	1.00E-36	mussel_c31004	hypothetical protein BRAFLDRAFT_121860 [Branchiostoma floridae]
MGO_09295	Baculoviral IAP repeat-containing protein 3	-0.78	3.00E-41	mussel_rep_c39920	baculoviral IAP repeat-containing 3 [Mus musculus]
MGO_09172	Unknown (TNF-like)	-0.79	3.00E-09	mussel_c3679	sialic acid-binding lectin [Venerupis philippinarum]
MGO_03559	Macrophage migration inhibitory factor	-0.79	2.00E-63	mussel_c10585	macrophage migration inhibitory factor [Ascaris suum]
MGO_01734	Keratin-associated protein 5-4	-0.79	-	No hits found	
MGO_00542	Unknown (*Mytilin B)	-0.79	-	No hits found	
MGO_09469	Collagen alpha-1(XII) chain	-0.81	-	No hits found	
MGO_09294	BRCA1-associated RING domain protein 1	-0.81	3.00E-52	mussel_rep_c26319	ank repeat-containing [Schistosoma mansoni]
MGO_00709	Double-strand-break repair protein rad21 homolog	-0.81	2.00E-18	mussel_c51605	No hits
MGO_02872	Nucleoprotein TPR	-0.82	-	No hits found	
MGO_06262	Transcription intermediary factor 1-beta	-0.82	-	No hits found	
MGO_00911	Uncharacterized serine-rich protein C215.13	-0.83	-	No hits found	
MGO_07704	Unknown (Ankyrin repeat)	-0.84	-	No hits found	
MGO_00860	Integumentary mucin C.1	-0.85	7.00E-08	mussel_rep_c23389	P-domain peptide precursor [Xenopus laevis]
MGO_06749	Death-associated protein kinase 1	-0.86	7.00E-04	mussel_c46485	No hits
MGO_00273	Heavy metal-binding protein HIP	-0.88	8.00E-35	mussel_c8742	Complement C1q-like protein 2 [Salmo salar]
MGO_06945	Collagen alpha-1(VIII) chain	-0.88	-	No hits found	
MGO_00301	Stress-induced protein 1	-0.90	3.00E-73	mussel_rep_c23478	PREDICTED: similar to small heat shock protein [Hydra magnipapillata]
MGO_03821	Uncharacterized protein PFB0145c	-0.90	-	No hits found	
MGO_01920	ATP-dependent RNA helicase DBP2	-0.91	2.00E-06	mussel_c43543	hypothetical protein TRIADDRAFT_26030 [Trichoplax adhaerens]
MGO_08652	Kinase D-interacting substrate of 220 kDa	-0.92	-	No hits found	
MGO_00522	Complement C1q tumor necrosis factor-related protein 3	-0.92	-	No hits found	
MGO_02605	Protein still life, isoforms C/SIF type 2	-0.93	-	No hits found	
MGO_00536	Macrophage mannose receptor 1, MR1	-0.93	2.00E-04	mussel_c63052	isoamyl acetate-hydrolyzing esterase, putative [Ixodes scapularis]
MGO_05656	Hepatic lectin	-0.93	-	No hits found	
MGO_05376	Unknown (Aerolisin/ETX pore-forming domain)	-0.95	-	No hits found	
MGO_08268	Unknown	-0.96	1.00E-23	mussel_c64562	predicted protein [Nematostella vectensis]
MGO_02703	Techylectin-5B	-0.96	4.00E-07	mussel_c7017	PREDICTED: similar to MGC107780 protein [Strongylocentrotus purpuratus]
MGO_03787	Lysozyme 3	-0.98	-	No hits found	
MGO_03710	Unknown (C1q ; Gliacolin-related; Cerebellin-related; TNF-like)	-0.98	-	No hits found	
MGO_07996	Fucolectin-6	-1.01	-	No hits found	
MGO_01140	Myticin-A	-1.02	-	No hits found	
MGO_06139	Complement C1q tumor necrosis factor-related protein 2	-1.02	-	No hits found	
MGO_09001	Neuronal calcium sensor 2	-1.02	8.00E-78	mussel_c2242	predicted protein [Nematostella vectensis]
MGO_03686	Myticin-B	-1.02	-	No hits found	
MGO_07688	Interferon alpha-inducible protein 27-like protein 2	-1.03	-	No hits found	
MGO_02030	Aminopeptidase N	-1.04	-	No hits found	
MGO_03915	Platelet endothelial aggregation receptor 1	-1.04	2.00E-11	mussel_c29542	PREDICTED: similar to MEGF6 [Gallus gallus]
MGO_04833	Fucolectin-6	-1.04	-	No hits found	
MGO_00286	Hepatic lectin	-1.05	-	No hits found	
MGO_00773	Defensin MGD-2	-1.05	-	No hits found	
MGO_07656	Unknown (C2H2 and C2HC zinc fingers)	-1.05	2.00E-39	mussel_c54081	No hits
MGO_00125	Fibrinogen C domain-containing protein 1	-1.06	2.00E-16	mussel_c31141	hypothetical protein BRAFLDRAFT_59661 [Branchiostoma floridae]
MGO_06796	Baculoviral IAP repeat-containing protein 7-A, IAP	-1.06	1.00E-03	mussel_c5979	hypothetical protein BRAFLDRAFT_224204 [Branchiostoma floridae]
MGO_00455	Unknown	-1.07	-	No hits found	
MGO_02324	DNA ligase 1	-1.08	-	No hits found	
MGO_04872	Nucleolar protein 58	-1.08	-	No hits found	
MGO_04820	Papilin	-1.08	3.00E-37	mussel_c51088	No hits
MGO_06436	Neurotrypsin (scavenger receptor cysteine-rich protein precursor, SRCR)	-1.08	8.00E-04	mussel_c59462	No hits
MGO_00299	Unknown	-1.10	-	No hits found	
MGO_04540	Complement C1q-like protein 4	-1.11	2.00E-12	mussel_c1219	PREDICTED: similar to C1q domain containing 1, partial [Ornithorhynchus anatinus]
MGO_07880	E3 ubiquitin-protein ligase, UBR5	-1.12	-	No hits found	
MGO_04610	Fibrinogen-like protein A	-1.13	3.00E-09	mussel_rep_c74228	hypothetical protein BRAFLDRAFT_86509 [Branchiostoma floridae]
MGO_04013	C1q-related factor	-1.13	-	No hits found	
MGO_04808	EF-hand domain-containing protein D2	-1.15	3.00E-135	mussel_c1455	PREDICTED: hypothetical protein [Strongylocentrotus purpuratus]
MGO_00639	Defensin MGD-2	-1.16	-	No hits found	
MGO_02419	Galectin-4	-1.19	9.00E-09	mussel_rep_c23964	galectin [Pinctada fucata]
MGO_05831	Collagen alpha-1(VIII) chain	-1.19	1.00E-19	mussel_c8661	gliacolin-related [Schistosoma mansoni]
MGO_05924	Unknown (Asialoglycoprotein receptor)	-1.21	2.00E-10	mussel_rep_c66303	hypothetical protein BRAFLDRAFT_224211 [Branchiostoma floridae]
MGO_03646	Unknown	-1.24	1.00E-20	mussel_rep_c27649	No hits
MGO_00274	Ficolin-2	-1.25	1.00E-14	mussel_c1346	hypothetical protein BRAFLDRAFT_86061 [Branchiostoma floridae]
MGO_04722	Perlucin	-1.25	-	No hits found	
MGO_00791	WSC domain-containing protein 2	-1.26	-	No hits found	
MGO_03747	Apoptosis regulator Bcl-2	-1.28	-	No hits found	
MGO_03984	Collagen alpha-1(XII) chain	-1.30	-	No hits found	
MGO_04518	Fibrinogen C domain-containing protein 1	-1.32	2.00E-32	mussel_c2013	hypothetical protein BRAFLDRAFT_86061 [Branchiostoma floridae]
MGO_00371	Fibrinogen C domain-containing protein 1	-1.32	2.00E-26	mussel_c5428	hypothetical protein BRAFLDRAFT_86061 [Branchiostoma floridae]
MGO_05878	Mytimycin	-1.33	2.00E-04	mussel_c2159	hypothetical protein MA2045 [Methanosarcina acetivorans C2A]
MGO_07415	Angiopoietin-4	-1.34	9.00E-10	mussel_c755	hypothetical protein BRAFLDRAFT_86061 [Branchiostoma floridae]
MGO_04026	Unknown (TNF-like)	-1.34	8.00E-10	mussel_c264	mantle gene 4 [Pinctada fucata]
MGO_00517	Heavy metal-binding protein HIP	-1.34	-	No hits found	
MGO_00302	Toxin CrTX-A	-1.35	-	No hits found	
MGO_08281	Neurogenic locus notch homolog protein 2	-1.37	-	No hits found	
MGO_09416	Cholecystokinin receptor type A	-1.40	-	No hits found	
MGO_00413	Unknown	-1.42	-	No hits found	
MGO_01206	ATP synthase subunits region ORF 7	-1.43	-	No hits found	
MGO_00292	Unknown (apextrin)	-1.44	1.00E-03	mussel_rep_c34433	No hits
MGO_08675	Perlucin	-1.44	-	No hits found	
MGO_00774	A-agglutinin anchorage subunit	-1.44	-	No hits found	
MGO_00508	Complement C1q-like protein 3	-1.45	2.00E-11	mussel_rep_c67050	PREDICTED: similar to Collagen alpha-1(VIII) chain [Monodelphis domestica]
MGO_04501	Fucolectin-6	-1.49	-	No hits found	
MGO_08029	N,N'-diacetylchitobiase	-1.50	4.00E-34	mussel_c46276	predicted protein [Nematostella vectensis]
MGO_04032	Collagen alpha-1(XII) chain	-1.53	-	No hits found	
MGO_05365	Collectin-12	-1.55	-	No hits found	
MGO_00348	Fibrinogen-like protein A	-1.56	7.00E-04	mussel_c1874	hypothetical protein BRAFLDRAFT_86061 [Branchiostoma floridae]
MGO_06511	Formin-binding protein 1, FNBP1	-1.56	-	No hits found	
MGO_00921	Hippocalcin-like protein 1	-1.58	4.00E-115	mussel_c2631	neuronal calcium sensor [Schistosoma mansoni]
MGO_04761	Pre-mRNA-processing factor 19	-1.64	1.00E-08	mussel_c34744	hypothetical protein BRAFLDRAFT_121774 [Branchiostoma floridae]
MGO_06940	Complement C1q-like protein 3	-1.67	1.00E-06	mussel_c16025	PREDICTED: similar to FLJ00201 protein [Gallus gallus]
MGO_00394	Unknown (C-type lectin domain)	-1.67	-	No hits found	
MGO_06703	DNA-binding protein inhibitor ID-2-B	-1.67	3.00E-117	mussel_rep_c23668	hypothetical protein TcasGA2_TC000024 [Tribolium castaneum]
MGO_00333	Collagen alpha-2(VIII) chain	-1.68	2.00E-11	mussel_c7198	unnamed protein product [Tetraodon nigroviridis]
MGO_01028	Collagen alpha-1(VIII) chain	-1.68	1.00E-21	mussel_c8661	gliacolin-related [Schistosoma mansoni]
MGO_00284	Unknown (C1Q, TNF-like)	-1.74	2.00E-18	mussel_c264	mantle gene 4 [Pinctada fucata]
MGO_08987	Golgi-associated plant pathogenesis-related protein 1	-1.82	-	No hits found	
MGO_04511	Serine/threonine-protein kinase 16	-1.85	-	No hits found	
MGO_00402	Complement C1q tumor necrosis factor-related protein 4	-1.86	1.00E-27	mussel_c27941	hypothetical protein BRAFLDRAFT_69125 [Branchiostoma floridae]
MGO_00270	Myticin-A	-1.89	-	No hits found	
MGO_00280	Defensin MGD-1	-1.90	5.00E-04	mussel_c29582	No hits
MGO_08974	Heavy metal-binding protein HIP	-1.94	1.00E-14	mussel_c264	mantle gene 4 [Pinctada fucata]
MGO_07080	Peptidyl-prolyl cis-trans isomerase CWC27 homolog	-1.95	7.00E-04	mussel_c2831	hypothetical protein BRAFLDRAFT_116129 [Branchiostoma floridae]
MGO_04044	Unknown	-1.97	-	No hits found	
MGO_09097	Complement C1q tumor necrosis factor-related protein 3	-1.98	4.00E-13	mussel_c2254	sialic acid binding lectin [Helix pomatia]
MGO_00365	Complement C1q tumor necrosis factor-related protein 4	-2.00	-	No hits found	
MGO_00441	Unknown (*apextrin)	-2.05	1.00E-03	mussel_rep_c34433	No hits
MGO_00623	Unknown	-2.06	-	No hits found	
MGO_05021	Unknown (C-type lectin-like)	-2.08	-	No hits found	
MGO_00358	Defensin MGD-2	-2.08	-	No hits found	
MGO_00322	Complement C1q tumor necrosis factor-related protein 4	-2.08	-	No hits found	
MGO_04638	Complement C1q-like protein 3	-2.08	3.00E-09	mussel_rep_c69103	PREDICTED: hypothetical protein [Danio rerio]
MGO_04631	Unknown (*C1q-like)	-2.10	-	No hits found	
MGO_01080	Unknown	-2.10	-	No hits found	
MGO_02691	Collagen alpha-1(V) chain	-2.11	-	No hits found	
MGO_00779	WSC domain-containing protein 2	-2.11	-	No hits found	
MGO_08621	Unknown (*apextrin)	-2.12	-	No hits found	
MGO_06290	C-type lectin domain family 4 member M	-2.14	-	No hits found	
MGO_08969	Complement C1q-like protein 3	-2.15	2.00E-05	mussel_c16025	PREDICTED: similar to FLJ00201 protein [Gallus gallus]
MGO_01089	Complement C1q tumor necrosis factor-related protein 3	-2.17	1.00E-27	mussel_c2254	sialic acid binding lectin [Helix pomatia]
MGO_04953	Unknown (*apextrin)	-2.20	-	No hits found	
MGO_00744	Unknown (C-type lectin-like)	-2.50	-	No hits found	
MGO_00451	Defensin MGD-1	-2.58	-	No hits found	
MGO_04267	Ficolin-2	-2.70	2.00E-12	mussel_c10289	hypothetical protein BRAFLDRAFT_86061 [Branchiostoma floridae]
MGO_00529	Skin secretory protein xP2	-2.73	2.00E-17	mussel_rep_c23389	P-domain peptide precursor [Xenopus laevis]
MGO_08088	Unknown (*apextrin)	-2.91	-	No hits found	
MGO_03823	Unknown (EF-hand)	-3.02	-	No hits found	
MGO_00845	Unknown (*apextrin)	-3.27	-	No hits found	

No hits found: no counterpart found in the DeepSeaVent *Bathymodiolus azoricus* database (e-value ≥ 0.001, BLASTN 2.2.23+)
