# Supplementary material for: Insights into the innate immunity of the Mediterranean mussel Mytilus galloprovincialis
Source: BMC Genomics. 2011 Jan 26;12:69. doi: 10.1186/1471-2164-12-69 (PMC3039611; doi:10.1186/1471-2164-12-69)
Supplement: Additional file 4 — Common differentially expressed genes in mussel hemocytes at 3 and 48 h post-injection of live V. splendidus. Probe ID, sequence information and ordered expression values (log2 of normalized test/control values). Similarities resulting from InterproScan Analysis are reported in brackets (* annotation based on manual inspection of other relevant similarities) [file 1471-2164-12-69-S4.PDF]

Additional file 4
Common differentially expressed genes in mussel hemocytes at 3 and 48 h post-injection of live *V. splendidus*

ID	Sequence similarity description	Expression Value	
		3 h	48 h
MGO_03478	Ependymin-2	1.47	2.93
MGO_00233	Alpha-protein kinase vwkA	1.80	2.82
MGO_05648	Plasminogen	2.30	2.45
MGO_04110	Superoxide dismutase [Cu-Zn], SOD	1.75	2.29
MGO_00991	Allograft inflammatory factor 1, AIF	2.45	2.29
MGO_00161	Apolipophorin	1.90	2.22
MGO_04450	ETS homologous factor	2.22	1.83
MGO_00580	Transmembrane protein 205	1.42	1.80
MGO_07770	Myeloid differentiation primary response protein MyD88	1.60	1.73
MGO_04498	B(0,+)-type amino acid transporter 1	1.10	1.71
MGO_05528	Ras association domain-containing protein 1	1.25	1.61
MGO_04578	Heavy metal-binding protein HIP	2.83	1.57
MGO_04809	Techylectin-5B	1.23	1.49
MGO_06401	Unknown	1.54	1.49
MGO_00924	Delta and Notch-like epidermal growth factor-related receptor	1.16	1.43
MGO_04209	Peptidoglycan recognition protein 1, PGRP	1.07	1.43
MGO_05083	WAS protein family member 3	2.88	1.42
MGO_06198	Tetraspanin-7/ CD63 antigen	1.05	1.34
MGO_00425	Major egg antigen (sHSP20)	1.86	1.29
MGO_01452	Very low-density lipoprotein receptor	1.06	1.26
MGO_06800	Type-1B angiotensin II receptor	1.20	1.17
MGO_00786	Unknown (C-type lectin-like)	0.83	1.10
MGO_01319	Actin, adductor muscle	1.21	1.06
MGO_07746	Unknown (C-type lectin-like)	1.37	0.98
MGO_02087	Myosin heavy chain, striated muscle	1.34	0.95
MGO_05290	RING finger protein ETP1	2.09	0.89
MGO_04603	Endoplasmin GRP94	1.15	0.87
MGO_03647	Unknown (*MAC/perforin- and kringle-domains-containing protein)	-1.61	0.83
MGO_00501	Integumentary mucin C.1	-1.43	-0.73
MGO_09295	Baculoviral IAP repeat-containing protein 3, IAP	2.34	-0.78
MGO_03559	Macrophage migration inhibitory factor, MIF	-1.29	-0.79
MGO_09294	BRCA1-associated RING domain protein 1	-1.12	-0.81
MGO_06262	Transcription intermediary factor 1-beta	1.16	-0.82
MGO_09001	Neuronal calcium sensor 2	-1.60	-1.02
MGO_00125	Fibrinogen C domain-containing protein 1	1.25	-1.06
MGO_06796	Baculoviral IAP repeat-containing protein 7-A, IAP	1.57	-1.06
MGO_04820	Papilin	0.96	-1.08
MGO_06436	Neurotrypsin (scavenger receptor cysteine-rich protein precursor, SRCR)	-1.69	-1.08
MGO_00791	WSC domain-containing protein 2	-1.82	-1.26
MGO_00517	Heavy metal-binding protein HIP	-1.66	-1.34
MGO_00413	Unknown	-1.15	-1.42
MGO_00292	Unknown (*apextrin)	-2.43	-1.44
MGO_00508	Complement C1q-like protein 3	-1.35	-1.45
MGO_00333	Collagen alpha-2(VIII) chain	-1.92	-1.68
MGO_08987	Golgi-associated plant pathogenesis-related protein 1	-1.63	-1.82
MGO_00270	Myticin-A	-1.26	-1.89
MGO_01080	Unknown	-1.52	-2.10
MGO_00779	WSC domain-containing protein 2	-1.21	-2.11
MGO_01089	Complement C1q tumor necrosis factor-related protein 3	1.35	-2.17
MGO_00451	Defensin MGD-1	-2.24	-2.58
MGO_04267	Ficolin-2	-1.42	-2.70
MGO_00845	Unknown (*apextrin)	-2.56	-3.27
